# Supplementary material for: Photochemical diazidation of alkenes enabled by ligand-to-metal charge transfer and radical ligand transfer
Source: Nat Commun. 2022 Dec 23;13:7881. doi: 10.1038/s41467-022-35560-3 (PMC9789121; doi:10.1038/s41467-022-35560-3)
Supplement: Supplementary file 1 — Supplementary Information [file 41467_2022_35560_MOESM1_ESM.pdf]

Supplementary Information

For

Photochemical Diazidation of Alkenes Enabled by Ligand-to-Metal Charge Transfer and Radical Ligand Transfer

Kang-Jie Bian,[†] Shih-Chieh Kao,[†] David Nemoto Jr., Xiao-Wei Chen, Julian G. West[*]

Department of Chemistry, Rice University, 6500 Main St, Houston, TX-77005, USA.

Contents

I. Supplementary Methods 3

1.1 General Information 3

1.2 General Procedures for Substrate Synthesis 3

General Procedure 1 for the Synthesis of Unactivated Alkenes 3

General Procedure 2 for the Synthesis of Unactivated Alkenes 4

Procedure for the Synthesis of N-(but-3-en-1-yl)benzenesulfonamide 4

Procedure for the Synthesis of N-(but-3-en-1-yl)-N-methylbenzenesulfonamide 4

Procedure for the Synthesis of 2-(but-3-en-1-yl)isoindoline-1,3-dione 5

1.3 General Procedures for Diazidation of Alkenes 6

1.4 General Procedures for Derivatization 6

II. Supplementary Discussion 8

2.1 Optimization of Photochemical Diazidation (Batch) 8

Supplementary Table 1. Preliminary Screening of Batch Diazidation (metal salt). 8

Supplementary Table 2. Screening of Batch Diazidation (solvent). 9

Supplementary Table 3. Screening of Batch Diazidation (concentration). 10

Supplementary Table 4. Screening of Batch Diazidation (control experiments). 10

Supplementary Table 5. Screening of Catalytic, Batch Diazidation (catalyst/reactants loading, concentration and atmosphere mixture). 11

Supplementary Table 6. Screening of Catalytic, Batch Dizidation (Oxidant). 12

2.2 Optimization of Photochemical Diazidation (Flow Reaction) 13

Supplementary Table 7. Screening of Catalytic, Flow Diazidation. 13

2.3 Scope of Photochemical Diazidation (Flow Reaction) 14

Supplementary Table 8. Substrate Scope of Diazidation of Alkenes (Flow Reaction). 14

2.4 Photochemical Diazidation Setup (Batch & Flow Reaction) 15

Supplementary Table 9. Photochemical Diazidation Setup. A. batch diazidation. B, C & D. flow diaziadation 15

2.5 Characterization of Corresponding Products 16

III. Supplementary Figures 31

IV. Supplementary Reference 158

I. Supplementary Methods

1.1 General Information

All reagents were purchased from commercially available sources and used without further purification. All reactions were monitored by either ^{1}H NMR or thin layer chromatography (TLC) carried out on 0.25 mm pre-coated silia plates (F-254) purchased from Silicycle, Quebec, Canada, using shortwave UV light as visualizing agent and KMnO4 or phosphomolybdic acid (PMA) as developing agents. Flash column chromatography was performed using SiliaFlash-P60 silica gel (40 – 63 µm) purchased from Silicycle, Quebec, Canada. ^{1}H, ^{13}C and ^{19}F NMR spectra were recorded on a Bruker DRX-600 spectrometers operating at 600 MHz for proton nuclei, 151 MHz for carbon nuclei and 565 MHz for fluorine nuclei were calibrated using residual undeuterated solvent as an internal reference (CDCl3: 7.26 ppm ^{1}H NMR and 77.00 ppm ^{13}C NMR). 25 W PR160L 427 nm LEDs from Kessil Lights were used as light source. For reporting NMR peak multiplicities, the following abbreviations were used: s = singlet, d = doublet, t = triplet, q = quartet, quin = quintet, hept = heptet, m = multiplet. High-resolution mass spectra (HRMS) were recorded on an Agilent UHPLC TOF mass spectrometer using electrospray ionization time-of-flight (ESI-TOF), chemical ionization time-of-flight (CI-TOF) or atmospheric pressure chemical ionization (APCI).

1.2 General Procedures for Substrate Synthesis

General Procedure 1 for the Synthesis of Unactivated Alkenes

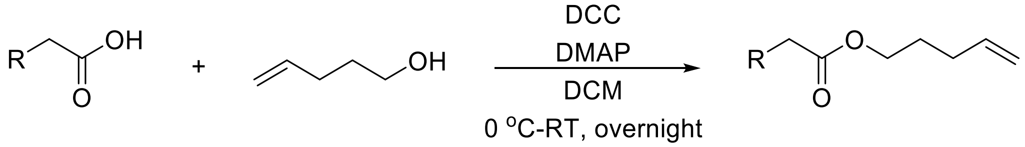

To an RB flask were added 5-pentenol (4.9 mmol, 1.96 equiv), carboxylic acid (2.5 mmol, 1.0 equiv), 4-dimethylamino pyridine (0.24 mmol, 0.097 equiv), and a stir bar. The RB flask was then evacuated and backfilled with nitrogen gas three times. Dry dichloromethane (0.225 M) was added via syringe to the RB flask, dissolving the solid components. The RB flask was then placed in an ice bath positioned on top of a stirring plate. Dicyclohexyl carbodiimide (4.85 mmol, 1.94 mmol) was added to the mixture via syringe dropwise over a period of 5 minutes. The ice bath was then removed, allowing the reaction to return to room temperature. The reaction was left to stir overnight. Following reaction, the mixture was concentrated through rotary evaporation.

Subsequent flash column chromatography (hexanes/EtOAc) allowed for isolation of the ester.[1-2]

General Procedure 2 for the Synthesis of Unactivated Alkenes

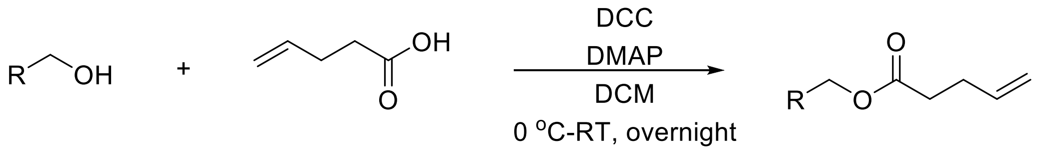

To an RB flask were added 5-pentenoic acid (2.5 mmol, 1.0 equiv), alcohol (4.9 mmol, 1.96 equiv), 4-dimethylamino pyridine (0.24 mmol, 0.097 equiv), and a stir bar. The RB flask was then evacuated and backfilled with nitrogen gas three times. Dry dichloromethane (0.225 M) was added via syringe to the RB flask, dissolving the solid components. The RB flask was then placed in an ice bath positioned on top of a stirring plate. Dicyclohexyl carbodiimide (4.85 mmol, 1.94 mmol) was added to the mixture via syringe dropwise over a period of 5 minutes. The ice bath was then removed, allowing the reaction to return to room temperature. The reaction was left to stir overnight. Following reaction, the mixture was concentrated through rotary evaporation. Subsequent flash column chromatography (hexanes/EtOAc) allowed for isolation of the ester.[1-2]

Procedure for the Synthesis of N-(but-3-en-1-yl)benzenesulfonamide

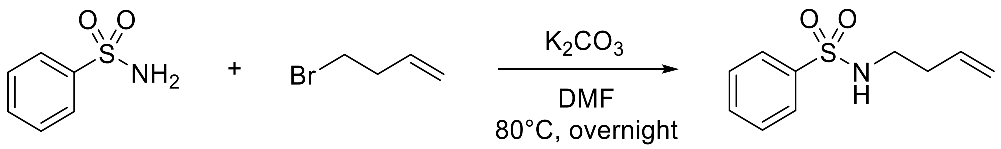

To an RB flask were added benzenesulfonamide (944 mg, 6.0 mmol), 4-bromobut-1-ene (0.8 mL, 6.0 mmol), dimethylformamide (30 mL), and a stir bar. Potassium carbonate (830 mg, 6.0 mmol) was added to the reaction mixture. After stirring overnight at 80 °C, the mixture was cooled to room temperature and quenched with water. The reaction mixture was then washed with brine and extracted with diethyl ether. The organic layer was concentrated through rotary evaporation. Subsequent flash column chromatography (hexanes/EtOAc) (3:1) allowed for isolation of *N*-(but-3-en-1-yl)benzenesulfonamide.[3]

Procedure for the Synthesis of N-(but-3-en-1-yl)-N-methylbenzenesulfonamide

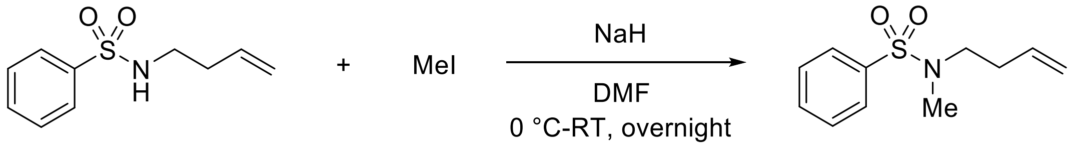

To an RB flask were added sodium hydride (60% in mineral oil, 240 mg, 6 mmol), dimethylformamide (25 mL), a solution of *N*-(but-3-en-1-yl)benzenesulfonamide (1.20 g, 5 mmol) in DMF (5 mL), and a stir bar in an ice bath at 0 °C. The reaction mixture was brought to room temperature and stirred for 30 minutes. The reaction mixture was cooled to 0 °C in an ice bath again, and a solution of methyl iodide (1.06 g, 7.5 mmol) in DMF (5 mL) was added dropwise over a period of 5 minutes by syringe. The reaction mixture was brought to room temperature and left to run overnight. The reaction mixture was quenched with a saturated aqueous solution of sodium bicarbonate. The mixture was then washed with brine and extracted with diethyl ether. The organic phase was dried over sodium sulfate and concentrated through rotary evaporation. Subsequent flash column chromatography (hexanes/EtOAc) (10:1) produced *N*-(but-3-en-1-yl)-*N*-methylbenzenesulfonamide.[4]

Procedure for the Synthesis of 2-(but-3-en-1-yl)isoindoline-1,3-dione

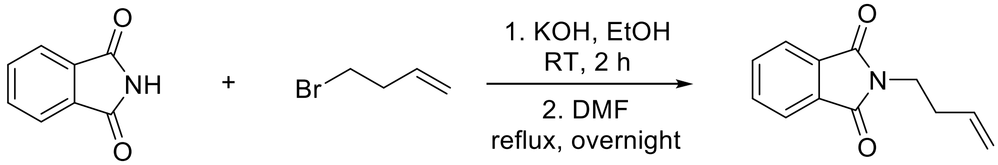

To an RB flask were added phthalimide (1.71 g, 11.6 mmol), potassium hydroxide (0.650 g, 11.6 mmol), ethyl alcohol (20 mL), and a stir bar. The reaction mixture was stirred at room temperature for 2 h and evaporated to remove EtOH. The resulting residue was then dissolved in dimethylformamide (15 mL) and 4-bromobut-1-ene (1.10 mL, 12.8 mmol) was added. The reaction mixture was stirred at reflux overnight. The reaction mixture was cooled, diluted with ethyl acetate, and quenched with saturated sodium bicarbonate. The mixture was then washed with brine. The extracted organic layer was dried over sodium sulfate and concentrated through rotary evaporation. Subsequent flash column chromatography (hexanes/EtOAc) (10:1) produced 2-(but-3-en-1-yl)isoindoline-1,3-dione.[5]

1.3 General Procedures for Diazidation of Alkenes

General Procedure A for batch diazidation of alkenes: Fe salt (0.15 mmol, 1.5 equiv.) was added in an oven-dried 8-mL test vial containing a Teflon®-coated magnetic stir bar. The vial was evacuated and backfilled with N2 (repeated for 4 times), followed by addition of alkenes (0.1 mmol, 1.0 equiv.) and TMSN3 (0.40 mmol, 4.0 equiv) in MeCN (1.0 mL, 0.1 M in regard to alkenes) via syringe under N2. The reaction mixture was placed under 25 W 427nm Kessil® light after sealing the punctured holes of the vial cap with vacuum grease and electric tape/parafilm for better air-tight protection and allowed to react at room temperature for 24 h. Following this, the reaction mixture was filtered through a pad of celite which was subsequently rinsed with DCM. The filtrate was concentrated, and the residue was then purified by flash column chromatography to give the corresponding diazidated products.

General Procedure B for 'continuous flow' diazidation of alkenes: Fe salt (0.035 mmol, 35 mol%), alkenes (0.10 mmol, 1.0 equiv.), TMSN3 (0.30 mmol, 3.0 equiv), MeCN (1.0 mL, 0.1 M in regard to alkenes) were added in an oven-dried 8-mL test vial containing a Teflon®-coated magnetic stir bar under N2. After the reaction mixture was withdrawn wtih a syringe, the syringe was connected to a FEP flow reactor and placed onto a syringe pump. the reaction mixture was pumped into a FEP flow reactor that was place under two 25 W 390nm Kessil® light at a rate of 0.06 mL/h. (Note: the length of reaction tube in the flow diazidation scope is 30 cm, see below for more information). The reaction mixture eluted from the outlet was discarded for the first 3h and the subsequent portion was collected for another 16h (on average ~1 mL). Following this, the collected portion was filtered through a pad of celite which was subsequently rinsed with DCM. The filtrate was concentrated, and the residue was then added dibromomethane as internal standard to determine the NMR yield of the corresponding diazidated products.

1.4 General Procedures for Derivatization

Derivatization Procedure A-Huisgen Cycloaddition: To a vial equipped with a stir bar was added the solution of diazide (0.23 mmol, 1.0 eq.) and phenylacetylene (0.057 mL, 0.51 mmol, 2.2 eq.) in t-BuOH:H_2O (1.5 mL, 2:1) at rt was added $CuSO_4{\cdot}5H_2O$ (5.7 mg, 0.023mmol, 0.1 eq.) and sodium ascorbate (9.1 mg, 0.046 mmol, 0.20 eq.) and the resulting reaction solution stirred at rt

for 24 h. The reaction was monitored via TLC analysis and was quenched with the addition of H_2O (5 mL) and the organic layer was extracted with CH_2Cl_2 (3 × 5 mL). The combined organic layers were further washed with H_2O and brine, dried (Na_2SO_4) and concentrated in vacuo. The crude product was purified via column chromatography (1:1 = hexane:ethyl acetate) to yield the title compound as a powdery white solid.[6]

Derivatization Procedure B-Reduction: To a vial equipped with a stir bar was added solution of diazide in anhydrous methanol followed by addition of Pd/C. The reaction was stirred under an atmosphere of H_2 (1 atm) overnight. The reaction mixture was filtered through a pad of silica and the filtrate was concentrated in vacuo. Residue was subsequently purified through column chromatography.[7]

Derivatization Procedure C-Reduction Followed by Boc-Protection: To a vial equipped with a stir bar were added the diazidation product (0.6 mmol, 1.0 equiv), H_2O (54 μL, 3.0 mmol, 5.0 equiv.) and THF (2 mL). After the vial was evacuated and backfilled twice with N_2, a solution of PPh_3 (346 mg, 1.32 mmol, 2.2 equiv) in THF (2 mL) was added drop-wise at 0 °C. The mixture was warmed up to room temperature and stirred for 8 h (monitored TLC). Subsequently, Boc_2O (393 mg, 1.8 mmol, 3.0 equiv) in THF (1.5 mL) was added to the above mixture dropwise at room temperature. The resulting mixture was stirred for additional 12 h until the diamine intermediates were fully consumed (monitored by TLC). After concentration in vacuo, the residue was subsequently purified through column chromatography.[8]

II. Supplementary Discussion

2.1 Optimization of Photochemical Diazidation (Batch)

Supplementary Table 1. Preliminary Screening of Batch Diazidation (metal salt).

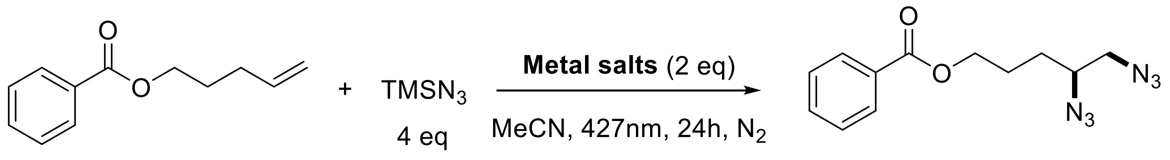

Entry	**Metal Salt**	yield
1	$CuBr_2$	~quant dibromo pdt
2	$Cu(OAc)_2$	NR
3	$Cu(acac)_2$	NR
4	$CoCl_2$ $6H_2O$	ND
5	$Co(acac)_2$	ND
6	$Mn(OAc)_3$	28
7	$Mn(OAc)_2$	NR

Supplementary Table 2. Screening of Batch Diazidation (solvent).

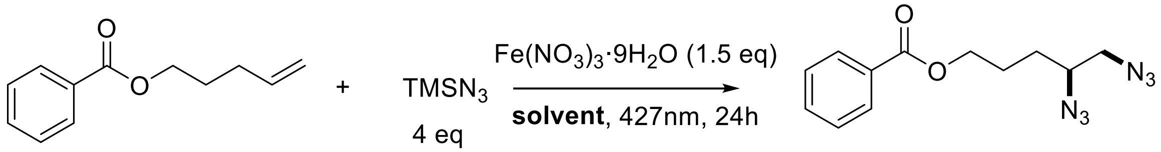

Entry	**solvent**	yield (%)
1	THF	ND
2	DCM	ND
3	Acetone	60
4	EA	64
5	MeCN	86
6	[a]DCM	trace

[a]4.5 equiv. of MeCN was added.

Supplementary Table 3. Screening of Batch Diazidation (concentration).

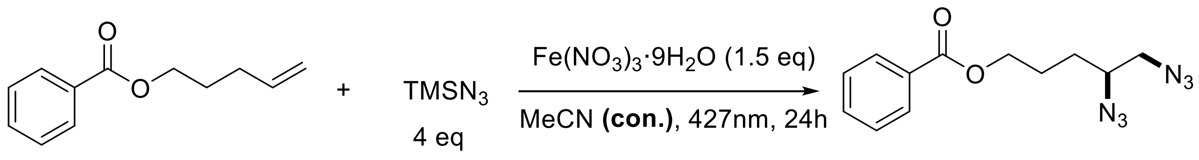

Entry	**con.**	yield (%)
1	0.05M	82
2	0.1M	86
3	0.2M	78

Supplementary Table 4. Screening of Batch Diazidation (control experiments).

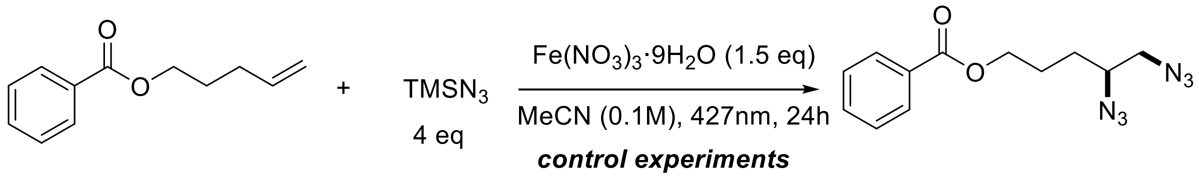

Entry	**Deviation from standard conditions**	yield (%)
1	none	86
2	dark (RT)	ND
3	dark (60 ^{o}C)	messy
4	no [Fe]	ND
5	under air	72
6	under air+ 20 mol% [Fe]	48

Supplementary Table 5. Screening of Catalytic, Batch Diazidation (catalyst/reactants loading, concentration and atmosphere mixture).

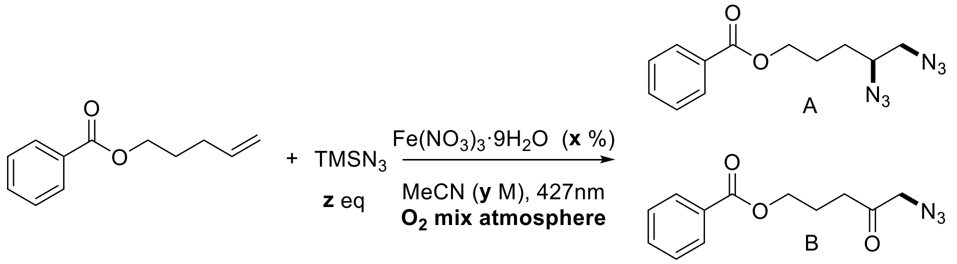

Entry	**x**	**y**	**z**	**atmosphere**	yield (%) (A/B)
1	10%	0.2M	6	1/4 N_2/O_2	44/8
2	10%	0.2M	6	1/8 N_2/O_2	52/10
3	20%	0.1M	6	1/4 N_2/O_2	48/8
4	20%	0.1M	6	1/8 N_2/O_2	56/8
5	30%	0.1M	6	1/4 N_2/O_2	52/12
6	30%	0.1M	6	1/8 N_2/O_2	56/16
7	40%	0.1M	4	air	52/10
8	40%	0.1M	5	air	52/6
9	40%	0.1M	6	air	52/8

Supplementary Table 6. Screening of Catalytic, Batch Dizidation (Oxidant).

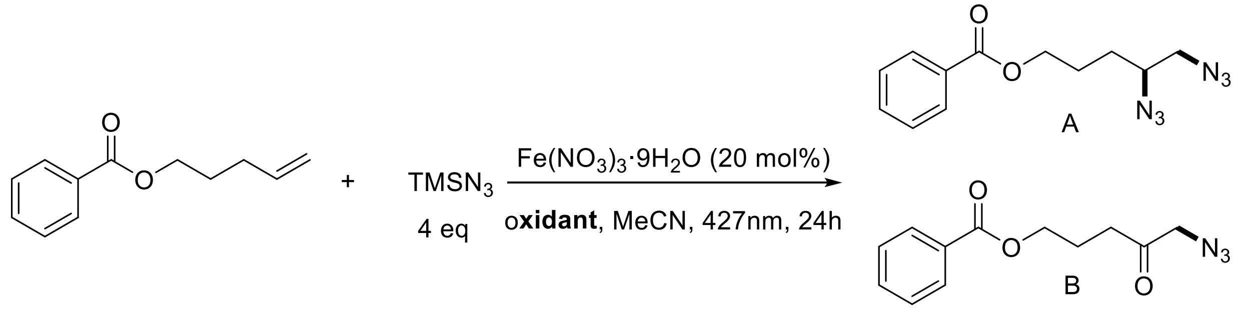

Entry	**Oxidant**	yield (%) (A/B)
1	O_2	24/14
2	$Cu(OAc)_2$	16/trace
3	$Mn(OAc)_2$	16/ND
4	$Cu(OTf)_2$	24/ND
5	disulfide	20/ND

2.2 Optimization of Photochemical Diazidation (Flow Reaction)

Supplementary Table 7. Screening of Catalytic, Flow Diazidation.

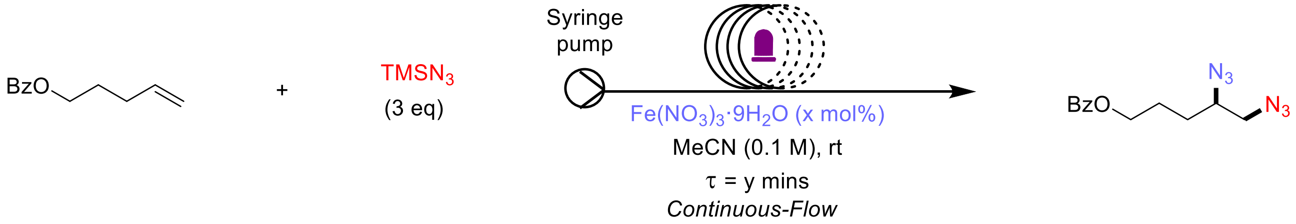

Entry	x (iron loading)	y (duration in reaction tubes)	tube length/velocity	yield (absolute) (%)
1	50 mol%	45 mins	10 cm/0.060 ml/h	62
2	35 mol%	68 mins	10 cm/0.040 ml/h	66
3	35 mol%	80 mins	10 cm/ 0.035 ml/h	64
4	35 mol%	135 mins	10 cm/0.030 ml/h	69
5	35 mol%	135 mins	30 cm/0.060 ml/h	72
6	30 mol%	135 mins	30 cm/0.060 ml/h	62

2.3 Scope of Photochemical Diazidation (Flow Reaction)

Supplementary Table 8. Substrate Scope of Diazidation of Alkenes (Flow Reaction).

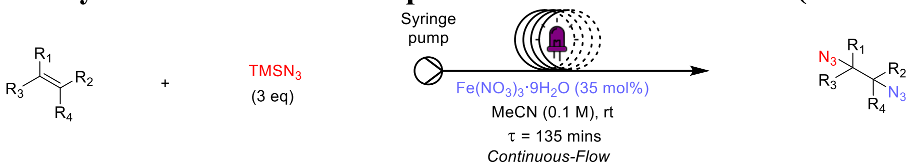

Product	Yield (absolute)	Yield (relative)	Product	Yield (absolute)	Yield (relative)
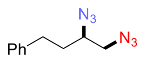	70%	92%	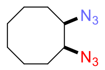	69% (4.2:1)	69%
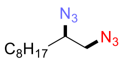	63%	76%	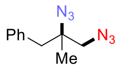	67%	67%
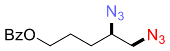	72% (63%)[a]	85% (89%)[a]	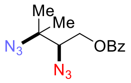	64%	64%
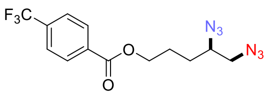	52%	69%	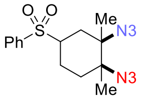	44%	44%
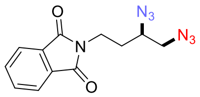	64%	94%	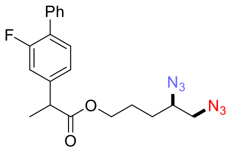	40%	43%
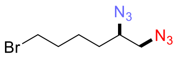	62%	73%	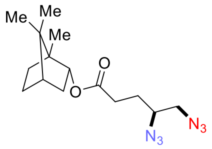	60%	78%

[a] 0.5 mmol scale. Isolated absolute yield.

2.4 Photochemical Diazidation Setup (Batch & Flow Reaction)

Supplementary Table 9. Photochemical Diazidation Setup. A. batch diazidation. B, C & D. flow diaziadation

A

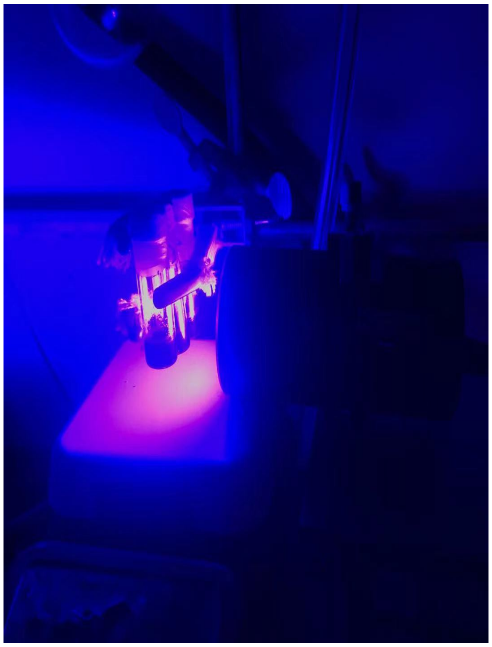

B

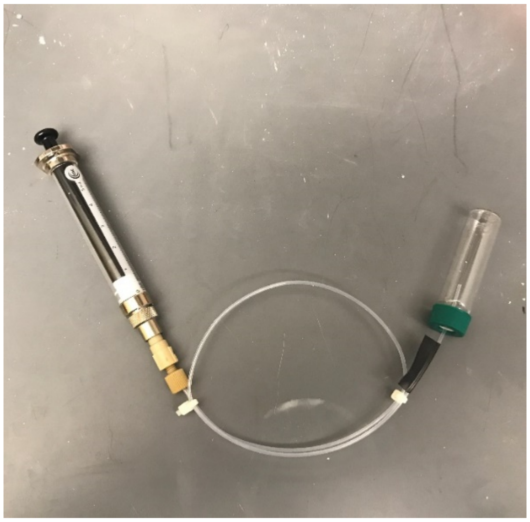

C

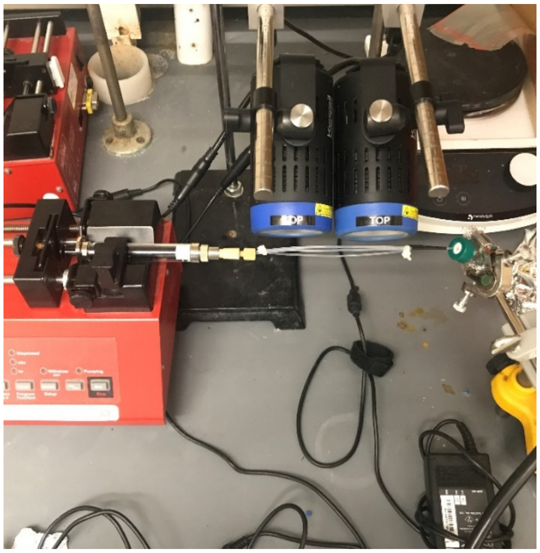

D

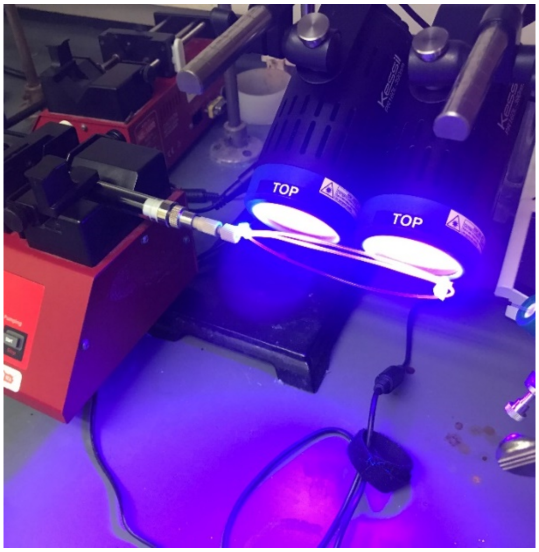

2.5 Characterization of Corresponding Products

General Procedure A for Batch Diazidation of Alkenes: Fe salt (0.15 mmol, 1.5 equiv.) was added in an oven-dried 8-mL test vial containing a Teflon®-coated magnetic stir bar. The vial was evacuated and backfilled with N2 (repeated for 4 times), followed by addition of alkenes (0.1 mmol, 1.0 equiv.) and TMSN3 (0.40 mmol, 4.0 equiv) in MeCN (1.0 mL, 0.1 M in regard to alkenes) via syringe under N2. The reaction mixture was placed under 25 W 427nm Kessil® light after sealing the punctured holes of the vial cap with vacuum grease and electric tape/parafilm for better air-tight protection and allowed to react at room temperature for 24 h. Following this, the reaction mixture was filtered through a pad of celite which was subsequently rinsed with DCM. The filtrate was concentrated, and the residue was then purified by flash column chromatography to give the corresponding diazidated products.

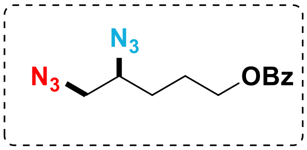

Prepared according to General Procedure A and obtained as colorless oil. ^{1}H NMR (600 MHz, Chloroform-*d*) δ 8.03 – 7.93 (m, 2H), 7.55 – 7.46 (m, 1H), 7.43 – 7.33 (m, 2H), 4.34 – 4.25 (m, 2H), 3.49 (ddt, *J* = 8.8, 7.2, 4.4 Hz, 1H), 3.38 (dd, *J* = 12.7, 4.2 Hz, 1H), 3.31 (dd, *J* = 12.7, 7.3 Hz, 1H), 1.97 – 1.86 (m, 1H), 1.85 – 1.76 (m, 1H), 1.71 – 1.55 (m, 2H). ^{13}C NMR (151 MHz, $CDCl_3$) δ 166.56, 133.11, 130.05, 129.58, 128.46, 64.15, 61.62, 54.87, 28.55, 25.33. HRMS APCI: $[M-N2+H]^+$ calcd. for C12H15N4O2: 247.1190; Found 247.1187

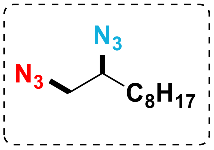

Prepared according to General Procedure A and obtained as colorless oil. ^{1}H NMR (600 MHz, Chloroform-*d*) δ 3.43 – 3.36 (m, 1H), 3.32 (dd, *J* = 12.7, 4.0 Hz, 1H), 3.24 (dd, *J* = 12.7, 7.5 Hz, 1H), 1.50-1.45 (m, 2H), 1.41-1.35 (m, 1H), 1.32 – 1.18 (m, 11H), 0.82 (t, *J* = 7.0 Hz, 3H).^{13}C NMR (151 MHz, $CDCl_3$) δ 62.10, 54.85, 31.83, 31.78, 29.39, 29.31, 29.19, 25.90, 22.67, 14.13. HRMS APCI: $[M-N2+H]^+$ calcd. for C10H21N4: 197.1761; Found 197.1759

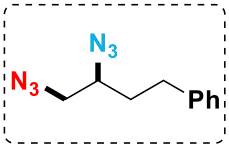

Prepared according to General Procedure A and obtained as colorless oil. ^{1}H NMR (600 MHz, Chloroform-*d*) δ 7.26-7.22 (m, 2H), 7.17 – 7.10 (m, 3H), 3.41 – 3.31 (m, 2H), 3.28 (dd, *J* = 12.6, 7.3 Hz, 1H), 2.75 (dt, *J* = 14.2, 7.2 Hz, 1H), 2.63 (dt, *J* = 13.8, 8.1 Hz, 1H), 1.82 – 1.72 (m, 2H).^{13}C NMR (151 MHz, $CDCl_3$) δ 140.39, 128.70, 128.43, 126.40, 61.14, 54.98, 33.43, 32.04. HRMS APCI: $[M-N2+H]^+$ calcd. for C10H13N4: 189.1135; Found 189.1133

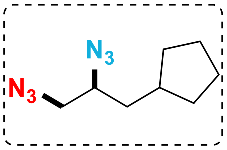

Prepared according to General Procedure A and obtained as colorless oil. ^{1}H NMR (600 MHz, Chloroform-*d*) δ 3.53 – 3.45 (m, 1H), 3.40 (dd, J = 12.7, 3.9 Hz, 1H), 3.32 (dd, J = 12.7, 7.4 Hz, 1H), 1.98 – 1.89 (m, 1H), 1.87-1.78 (m, 2H), 1.68-1.58 (m, 3H), 1.58-1.53 (m, 2H), 1.52-1.42 (m, 1H), 1.18 – 1.01 (m, 2H).^{13}C NMR (151 MHz, $CDCl_3$) δ 61.56, 55.13, 37.87, 36.76, 32.87, 32.38, 25.03, 24.95. HRMS APCI: $[M-N2+H]^+$ calcd. for C8H15N4: 167.1291; Found 167.1288

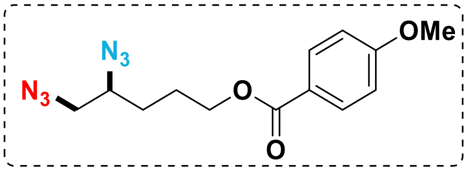

Prepared according to General Procedure A and obtained as colorless oil. ^{1}H NMR (600 MHz, Chloroform-*d*) δ 8.04 – 7.92 (m, 2H), 6.93 (d, J = 8.9 Hz, 2H), 4.38 – 4.27 (m, 2H), 3.87 (s, 3H), 3.59-3.52 (m, 1H), 3.45 (dd, J = 12.8, 4.2 Hz, 1H), 3.38 (dd, J = 12.7, 7.3 Hz, 1H), 2.00-1.91 (m, 1H), 1.91-1.81 (m, 1H), 1.79 – 1.64 (m, 2H). ^{13}C NMR (151 MHz, $CDCl_3$) δ 166.29, 163.43, 131.59, 122.42, 113.65, 63.81, 61.61, 55.45, 54.84, 28.54, 25.34. HRMS APCI: $[M-N2+H]^+$ calcd. for C13H17N4O3: 277.1295; Found 277.1290

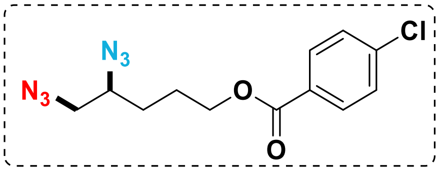

Prepared according to General Procedure A and obtained as colorless oil. ^{1}H NMR (600 MHz, Chloroform-*d*) δ 8.04 – 7.92 (m, 2H), 7.48 – 7.36 (m, 2H), 4.40 – 4.30 (m, 2H), 3.55 (ddt, J = 8.8, 7.2, 4.5 Hz, 1H), 3.45 (dd, J = 12.7, 4.2 Hz, 1H), 3.39 (dd, J = 12.7, 7.2 Hz, 1H), 2.02 – 1.92 (m, 1H), 1.90 – 1.81 (m, 1H), 1.74 – 1.63 (m, 2H).^{13}C NMR (151 MHz, $CDCl_3$) δ 165.67, 139.54, 130.95, 128.79, 128.45, 64.37, 61.54, 54.82, 28.47, 25.26. HRMS APCI: $[M-N2+H]^+$ calcd. for C12H14ClN4O2: 281.0800; Found 281.0799

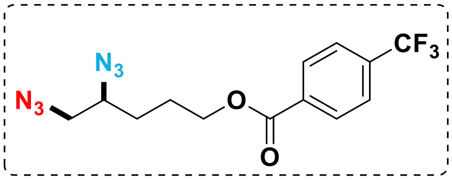

Prepared according to General Procedure A and obtained as colorless oil. ^{1}H NMR (600 MHz, Chloroform-*d*) δ 8.13 – 8.01 (m, 2H), 7.71 – 7.59 (m, 2H), 4.38 – 4.28 (m, 2H), 3.49 (ddt, J = 8.8, 7.1, 4.5 Hz, 1H), 3.39 (dd, J = 12.7, 4.3 Hz, 1H), 3.33 (dd, J = 12.7, 7.2 Hz, 1H), 1.98 – 1.89 (m, 1H), 1.86 – 1.77 (m, 1H), 1.71 – 1.55 (m, 2H). ^{13}C NMR (151 MHz, $CDCl_3$) δ 165.34, 134.57 (q, J = 32.7 Hz), 133.25, 130.00, 125.51 (q, J = 3.8 Hz), 123.61 (q, J = 272.9 Hz), 64.73, 61.54, 54.83, 28.47, 25.26. ^{19}F NMR (565 MHz, Chloroform-*d*) δ -63.09. HRMS APCI: $[M-N2+H]^+$ calcd. for C13H14F3N4O2: 315.1063; Found 315.1060

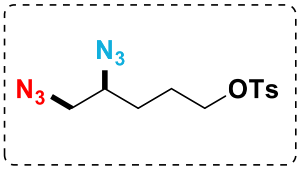

Prepared according to General Procedure A and obtained as colorless oil. ^{1}H NMR (600 MHz, Chloroform-*d*) δ 7.77 – 7.68 (m, 2H), 7.35 – 7.27 (m, 2H), 4.03-3.95 (m, 2H), 3.39 – 3.30 (m, 2H), 3.24 (dd, *J* = 12.5, 7.1 Hz, 1H), 2.39 (s, 3H), 1.80-1.73 (m, 1H), 1.71 – 1.62 (m, 1H), 1.58-1.52 (m, 1H), 1.47-1.40 (m, 1H). ^{13}C NMR (151 MHz, $CDCl_3$) δ 145.07, 132.80, 129.98, 127.92, 69.56, 61.28, 54.83, 27.94, 25.41, 21.70. HRMS APCI: $[M-N2+H]^+$ calcd. for C12H17N4O3S: 297.1016; Found 297.1014

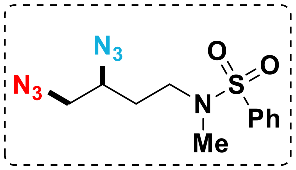

Prepared according to General Procedure A and obtained as colorless oil. ^{1}H NMR (600 MHz, Chloroform-*d*) δ 7.76 – 7.71 (m, 2H), 7.58 – 7.52 (m, 1H), 7.51-7.48 (m, 2H), 3.65 (ddt, *J* = 8.8, 6.8, 4.4 Hz, 1H), 3.46 (dd, *J* = 12.8, 4.1 Hz, 1H), 3.39 (dd, *J* = 12.8, 6.7 Hz, 1H), 3.18-3.12 (m, 1H), 2.96-2.92 (m, 1H), 2.69 (s, 3H), 1.78-1.71 (m, 1H), 1.63-1.57 (m, 1H).^{13}C NMR (151 MHz, $CDCl_3$) δ 136.88, 132.90, 129.25, 127.41, 58.96, 54.69, 47.03, 35.40, 29.89. HRMS APCI: $[M-N2+H]^+$ calcd. for C11H16N5O2S: 282.1019; Found 282.1016

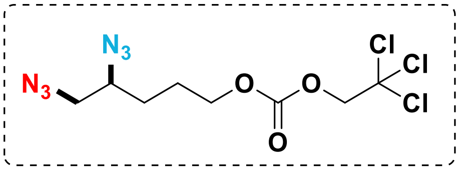

Prepared according to General Procedure A and obtained as colorless oil. ^{1}H NMR (600 MHz, Chloroform-*d*) δ 4.71 (s, 2H), 4.24-4.17 (m, 2H), 3.45 (ddt, *J* = 8.7, 7.1, 4.3 Hz, 1H), 3.37 (dd, *J* = 12.7, 4.2 Hz, 1H), 3.30 (dd, *J* = 12.7, 7.3 Hz, 1H), 1.90 – 1.81 (m, 1H), 1.79 – 1.69 (m, 1H), 1.65 – 1.58 (m, 1H), 1.57-1.50 (m, 1H). ^{13}C NMR (151 MHz, $CDCl_3$) δ 152.89, 93.32, 75.74, 67.30, 60.47, 53.81, 27.16, 24.10. HRMS APCI: $[M-N2+H]^+$ calcd. for C8H12Cl3N4O3: 316.9969; Found 316.9961

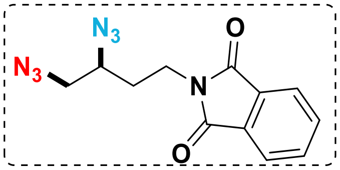

Prepared according to General Procedure A and obtained as white powder. ^{1}H NMR (600 MHz, Chloroform-*d*) δ 7.84 – 7.78 (m, 2H), 7.71 – 7.64 (m, 2H), 3.86 – 3.71 (m, 2H), 3.50 – 3.44 (m, 1H), 3.41 (dd, *J* = 12.7, 4.0 Hz, 1H), 3.35 (dd, *J* = 12.7, 7.4 Hz, 1H), 1.84 (ddt, *J* = 14.1, 7.0, 4.2 Hz, 1H), 1.74 (ddt, *J* = 14.2, 9.3, 6.3 Hz, 1H). ^{13}C NMR (151 MHz, $CDCl_3$) δ 168.31, 134.22, 123.46, 59.77, 34.69, 30.78. HRMS APCI: $[M-N2+H]^+$ calcd. for C12H12N5O2: 258.0986; Found 258.0985

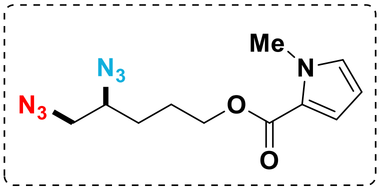

Prepared according to General Procedure A and obtained as colorless oil. ^{1}H NMR (600 MHz, Chloroform-*d*) δ 6.87 (dd, *J* = 4.0, 1.8 Hz, 1H), 6.73 (t, *J* = 2.2 Hz, 1H), 6.05 (dd, *J* = 4.0, 2.5 Hz, 1H), 4.23 – 4.14 (m, 2H), 3.86 (s, 3H), 3.51 – 3.44 (m, 1H), 3.37 (dd, *J* = 12.7, 4.1 Hz, 1H), 3.30 (dd, *J* = 12.7, 7.3 Hz, 1H), 1.89 – 1.80 (m, 1H), 1.78 – 1.70 (m, 1H), 1.68 – 1.57 (m, 2H).^{13}C NMR (151 MHz, $CDCl_3$) δ 161.19, 129.75,

122.22, 117.88, 107.93, 62.91, 61.63, 54.88, 36.88, 28.58, 25.40. HRMS APCI: [M-N2+H]$^+$ calcd. for C11H16N5O2: 250.1299; Found 250.1296

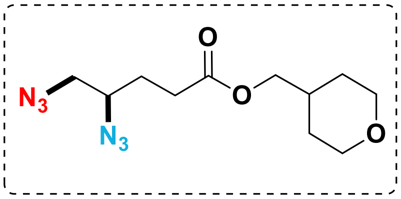

Prepared according to General Procedure A and obtained as colorless oil. ^{1}H NMR (600 MHz, Chloroform-*d*) δ 4.01 – 3.94 (m, 4H), 3.61-3.56 (m, 1H), 3.50 – 3.34 (m, 4H), 2.52-2.46 (m, 2H), 1.95-1.86 (m, 2H), 1.80-1.72 (m, 1H), 1.66 – 1.61 (m, 2H), 1.42-1.34(m, 2H). ^{13}C NMR (151 MHz, $CDCl_3$) δ 172.50, 69.01, 67.46, 61.11, 54.88, 34.48, 30.27, 29.49, 27.00. HRMS APCI: [M-N2+H]$^+$ calcd. for C11H19N4O3: 255.1452; Found 255.1440

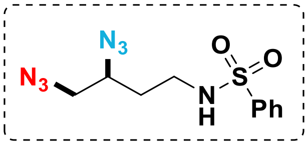

Prepared according to General Procedure A and obtained as colorless oil. ^{1}H NMR (600 MHz, Chloroform-*d*) δ 7.87 – 7.78 (m, 2H), 7.58 – 7.52 (m, 1H), 7.51-7.46 (m, 2H), 4.85 (t, *J* = 6.3 Hz, 1H), 3.61 – 3.54 (m, 1H), 3.37 (dd, *J* = 12.7, 4.1 Hz, 1H), 3.28 (dd, *J* = 12.8, 7.0 Hz, 1H), 3.07-2.97 (m, 2H), 1.70-1.64 (m, 1H), 1.56-1.50 (m, 1H). ^{13}C NMR (151 MHz, $CDCl_3$) δ 139.45, 133.01, 129.35, 127.05, 59.09, 54.78, 39.92, 31.71. HRMS APCI: [M-N2+H]$^+$ calcd. for C10H14N5O2S: 268.0683; Found 268.0680

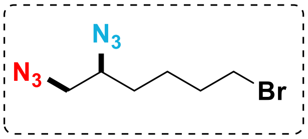

Prepared according to General Procedure A and obtained as colorless oil. ^{1}H NMR (600 MHz, Chloroform-*d*) δ 3.44-3.39 (m, 1H), 3.38 – 3.32 (m, 3H), 3.28 (dd, *J* = 12.7, 7.3 Hz, 1H), 1.88 – 1.77 (m, 2H), 1.61 – 1.45 (m, 4H). ^{13}C NMR (151 MHz, $CDCl_3$) δ 61.82, 54.78, 33.20, 32.23, 30.94, 24.54. HRMS APCI: [M-N2+H]$^+$ calcd. for C6H12BrN4: 219.0240; Found 219.0233

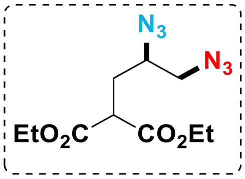

Prepared according to General Procedure A and obtained as colorless oil. ^{1}H NMR (600 MHz, Chloroform-*d*) δ 4.34 – 4.13 (m, 4H), 3.63 – 3.54 (m, 2H), 3.49 (ddd, *J* = 12.7, 4.1, 1.2 Hz, 1H), 3.39 (ddd, *J* = 12.7, 7.4, 1.2 Hz, 1H), 2.18 – 2.10 (m, 1H), 2.01-1.90(m, 1H), 1.35-1.23 (m, 6H). ^{13}C NMR (151 MHz, $CDCl_3$) δ 168.69, 168.66, 61.91, 61.87, 59.93, 55.06, 48.68, 30.93, 14.06, 14.01. HRMS APCI: [M-N2+H]$^+$ calcd. for C10H17N4O4: 257.1244; Found 257.1234

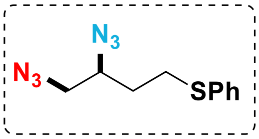

Prepared according to General Procedure A and obtained as colorless oil. ^{1}H NMR (600 MHz, Chloroform-*d*) δ 7.38-7.33 (m, 2H), 7.33-7.28 (m, 2H), 7.25 – 7.20 (m, 1H), 3.77 – 3.68 (m, 1H), 3.42 (dd, *J* = 12.7, 4.1 Hz, 1H), 3.35 (dd, *J* = 12.7, 7.3 Hz, 1H), 3.09 (dt, *J* = 13.1, 6.4 Hz, 1H), 2.98 (dt, *J* = 13.5, 7.7 Hz, 1H), 1.83-1.75 (m, 2H). ^{13}C NMR (151 MHz, $CDCl_3$) δ 135.14, 129.74, 129.13, 126.56, 60.38, 54.83, 31.22, 30.15. HRMS APCI: [M-N2+H]$^+$ calcd. for C10H13N4S: 221.0855; Found 221.0851

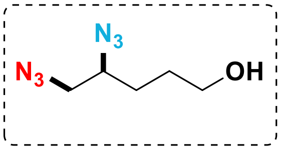

Prepared according to General Procedure A and obtained as colorless oil. ^{1}H NMR (600 MHz, Chloroform-*d*) δ 3.69 – 3.59 (m, 2H), 3.50 – 3.43 (m, 1H), 3.36 (dd, *J* = 12.7, 4.1 Hz, 1H), 3.29 (dd, *J* = 12.7, 7.4 Hz, 1H), 1.71 – 1.53 (m, 5H). ^{13}C NMR (151 MHz, $CDCl_3$) δ 62.20, 61.88, 54.92, 28.83, 28.35. HRMS APCI: $[M-N2+H]^+$ calcd. for C5H11N4O: 143.0927; Found 143.0923

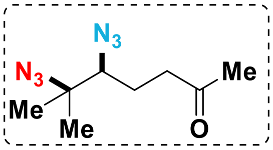

Prepared according to General Procedure A and obtained as colorless oil. ^{1}H NMR (600 MHz, Chloroform-*d*) δ 3.16 (dd, *J* = 11.4, 2.5 Hz, 1H), 2.75-2.66 (m, 1H), 2.63-2.55 (m, 1H), 2.19 (s, 3H), 2.02-1.94 (m, 1H), 1.59-1.50 (m, 1H), 1.34 (d, *J* = 6.6 Hz, 6H). ^{13}C NMR (151 MHz, $CDCl_3$) δ 207.58, 69.76, 64.76, 40.31, 30.12, 23.44, 23.31, 22.44. HRMS APCI: $[M-N2+H]^+$ calcd. for C8H15N4O: 183.1240; Found 183.1237

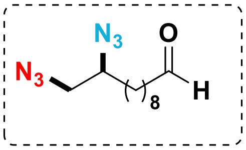

Prepared according to General Procedure A and obtained as colorless oil. ^{1}H NMR (600 MHz, Chloroform-*d*) δ 9.77 (t, *J* = 1.8 Hz, 1H), 3.50-3.41 (m, 1H), 3.39 (dd, *J* = 12.7, 4.0 Hz, 1H), 3.31 (dd, *J* = 12.7, 7.4 Hz, 1H), 2.43 (td, *J* = 7.3, 1.8 Hz, 2H), 1.68-1.59 (m, 2H), 1.56 – 1.51 (m, 2H), 1.48 – 1.31 (m, H). ^{13}C NMR (151 MHz, $CDCl_3$) δ 202.87, 62.04, 54.83, 43.88, 31.74, 29.19, 29.16, 29.07, 25.84, 22.01. HRMS APCI: $[M-N2+H]^+$ calcd. for C11H21N4O: 225.1710; Found 225.1706

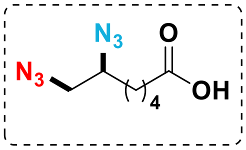

Prepared according to General Procedure A and obtained as colorless oil. ^{1}H NMR (600 MHz, Chloroform-*d*) δ 3.51-3.44 (m, 1H), 3.41 (dd, *J* = 12.7, 4.1 Hz, 1H), 3.33 (dd, *J* = 12.7, 7.3 Hz, 1H), 2.40 (t, *J* = 7.3 Hz, 2H), 1.76 – 1.62 (m, 2H), 1.60 – 1.50 (m, 3H), 1.49 – 1.40 (m, 1H). ^{13}C NMR (151 MHz, $CDCl_3$) δ 179.35, 61.78, 54.79, 33.67, 31.44, 25.33, 24.23. HRMS APCI: $[M-N2+H]^+$ calcd. for C7H13N4O2: 185.1033; Found 185.1030

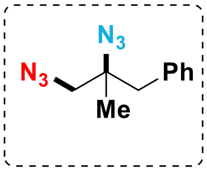

Prepared according to General Procedure A and obtained as colorless oil. ^{1}H NMR (600 MHz, Chloroform-*d*) δ 7.31 – 7.20 (m, 3H), 7.17 – 7.13 (m, 2H), 3.16 (s, 2H), 2.81 (d, *J* = 13.6 Hz, 1H), 2.75 (d, *J* = 13.6 Hz, 1H), 1.22 (s, 3H). ^{13}C NMR (151 MHz, $CDCl_3$) δ 135.36, 130.50, 128.44, 127.18, 63.97, 58.39, 43.24, 21.13. HRMS APCI: $[M-N2+H]^+$ calcd. for C10H13N4: 189.1135; Found 189.1134

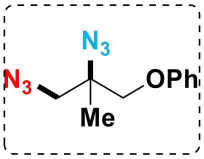

Prepared according to General Procedure A and obtained as colorless oil. ^{1}H NMR (600 MHz, Chloroform-*d*) δ 7.34 – 7.27 (m, 2H), 7.00 (tt, *J* = 7.4, 1.1 Hz, 1H), 6.95 – 6.87 (m, 2H), 4.04 – 3.87 (m, 2H), 3.49 (q, *J* = 12.5 Hz, 2H), 1.45 (s, 3H). ^{13}C NMR (151 MHz, $CDCl_3$) δ 158.03, 129.60, 121.60, 114.56, 71.24, 62.85, 56.20, 19.25. HRMS APCI: [M-N2+H]$^+$ calcd. for C10H13N4O: 205.1084; Found 205.1078

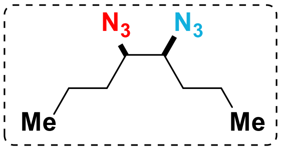

Prepared according to General Procedure A and obtained as colorless oil. ^{1}H NMR (600 MHz, Chloroform-*d*) δ 3.29 (dt, *J* = 5.8, 4.1 Hz, 1.11H), 3.21 (dt, *J* = 6.5, 4.1 Hz, 0.89H), 1.62 – 1.56 (m, 1H), 1.55 – 1.45 (m, 5H), 1.39 – 1.29 (m, 2H), 0.91 (td, *J* = 7.3, 2.3 Hz, 6H).^{13}C NMR (151 MHz, $CDCl_3$) δ 65.65, 65.05, 33.38, 32.43, 19.62, 19.50, 13.84. HRMS APCI: [M-N2+H]$^+$ calcd. for C8H17N4: 169.1448; Found 169.1447

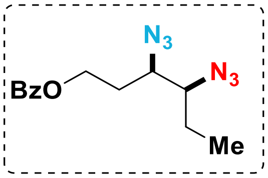

Prepared according to General Procedure A and obtained as colorless oil. ^{1}H NMR (600 MHz, Chloroform-*d*) δ 7.97 (dt, *J* = 8.4, 1.5 Hz, 2H), 7.56 – 7.47 (m, 1H), 7.43-7.36 (m, 2H), 4.51 – 4.42 (m, 1.02H), 4.41 – 4.33 (m, 0.98H), 3.54 (ddd, J = 10.4, 4.9, 3.1 Hz, 0.56H), 3.49 (dt, J = 9.1, 4.4 Hz, 0.44H), 3.34 (dt, J = 9.1, 4.5 Hz, 0.55H), 3.23 (ddd, J = 7.6, 5.9, 4.4 Hz, 0.45H), 2.07 – 1.96 (m, 1.46H), 1.85 (ddt, J = 15.0, 10.0, 4.8 Hz, 0.54H), 1.73-1.65 (m, 1H), 1.65-1.55 (m, 1H), 1.05-0.96 (m, 3H). ^{13}C NMR (151 MHz, $CDCl_3$) δ 166.40, 133.25, 133.22, 129.86, 129.82, 129.61, 128.52, 128.50, 67.32, 66.88, 62.44, 62.03, 61.54, 61.47, 30.68, 29.48, 24.42, 23.93, 10.82, 10.66. HRMS APCI: [M-N2+H]$^+$ calcd. for C13H17N4O2: 261.1346; Found 261.1338

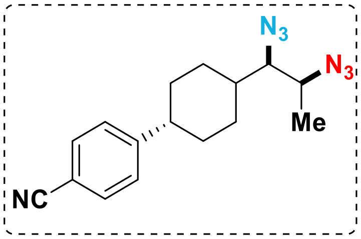

Prepared according to General Procedure A and obtained as colorless oil. ^{1}H NMR (600 MHz, Chloroform-*d*) δ 7.53-7.50 (m, 2H), 7.24-7.21 (m, 2H), 3.63 (qd, *J* = 6.6, 4.9 Hz, 0.38H), 3.57 (p, *J* = 6.4 Hz, 0.62H), 3.10 (t, *J* = 6.2 Hz, 0.62H), 2.88 (dd, *J* = 6.8, 5.0 Hz, 0.38H), 2.52-2.46 (m, 1H), 2.00 – 1.85 (m, 3H), 1.82 – 1.53 (m, 2H), 1.44 – 1.21 (m, 7H). ^{13}C NMR (151 MHz, $CDCl_3$) δ 152.27, 132.33, 127.64, 119.11, 109.94, 72.12, 72.02, 58.04, 57.61, 44.15, 38.85, 38.61, 33.26, 33.16, 33.05, 32.98, 30.07, 30.04, 28.39, 28.27, 16.85, 14.59. HRMS APCI: [M-N2+H]$^+$ calcd. for C16H20N5: 282.1713; Found 282.1701

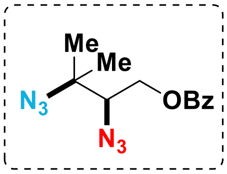

Prepared according to General Procedure A and obtained as colorless oil. ^{1}H NMR (600 MHz, Chloroform-*d*) δ 8.12 – 7.96 (m, 2H), 7.58 – 7.49 (m, 1H), 7.45 – 7.34 (m, 2H), 4.68 (dd, *J* = 11.5, 2.8 Hz, 1H), 4.23

(dd, J = 11.5, 9.5 Hz, 1H), 3.62 (dd, J = 9.4, 2.8 Hz, 1H), 1.35 (s, 3H), 1.32 (s, 3H). ^{13}C NMR (151 MHz, $CDCl_3$) δ 166.27, 133.46, 129.81, 129.33, 128.58, 68.32, 64.65, 62.11, 23.83, 22.96. HRMS APCI: $[M-N2+H]^+$ calcd. for C12H15N4O2: 247.1190; Found 247.1189

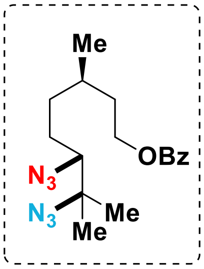

Prepared according to General Procedure A and obtained as colorless oil. ^{1}H NMR (600 MHz, Chloroform-*d*) δ 8.09 – 8.00 (m, 2H), 7.59 – 7.53 (m, 1H), 7.45 (t, J = 7.8 Hz, 2H), 4.45-4.28 (m, 2H), 3.09-2.99 (m, 1H), 1.91-1.76 (m, 1H), 1.74 – 1.54 (m, 4H), 1.51 – 1.37 (m, 2H), 1.34 – 1.29 (m, 6H), 1.01 (t, J = 6.9 Hz, 3H). ^{13}C NMR (151 MHz, $CDCl_3$) δ 166.65, 132.91, 130.34, 129.53, 128.36, 71.19, 70.91, 64.63, 64.61, 63.23, 63.19, 35.74, 35.17, 34.48, 34.25, 30.01, 29.83, 27.01, 26.98, 22.96, 22.94, 22.87, 19.62, 19.22. HRMS APCI: $[M-N2+H]^+$ calcd. for C17H25N4O2: 317.1972; Found 317.1956

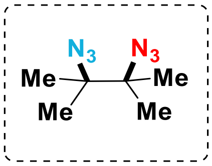

Prepared according to General Procedure A and obtained as colorless oil. ^{1}H NMR (600 MHz, Chloroform-*d*) δ 1.32 (s, 12H). ^{13}C NMR (151 MHz, $CDCl_3$) δ 66.55, 21.99. HRMS APCI: $[M-N2+H]^+$ calcd. for C6H13N4: 141.1135; Found 141.1134

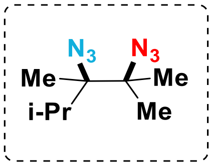

Prepared according to General Procedure A and obtained as colorless oil. ^{1}H NMR (600 MHz, Chloroform-*d*) δ 2.01 (hept, J = 6.8 Hz, 1H), 1.40 (s, 3H), 1.36 (s, 3H), 1.26 (s, 3H), 1.02 (dd, J = 10.5, 6.8 Hz, 6H). ^{13}C NMR (151 MHz, $CDCl_3$) δ 71.46, 67.73, 34.11, 23.18, 22.77, 19.75, 19.25, 13.02. HRMS APCI: $[M-N2+H]^+$ calcd. for C8H17N4: 169.1448; Found 169,1447

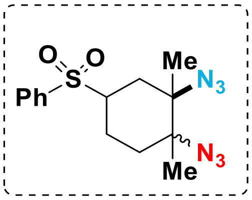

Prepared according to General Procedure A and obtained as colorless oil. ^{1}H NMR (600 MHz, Chloroform-*d*) δ 7.92 – 7.83 (m, 2H), 7.73 – 7.65 (m, 1H), 7.60 (t, J = 7.8 Hz, 2H), 3.23 (ddt, J = 12.3, 9.9, 4.1 Hz, 1H), 2.07 – 1.97 (m, 1H), 1.92 (ddd, J = 13.6, 3.7, 1.7 Hz, 1H), 1.83 – 1.68 (m, 4H), 1.37 (s, 3H), 1.32 (s, 3H). ^{13}C NMR (151 MHz, $CDCl_3$) δ 136.82, 133.96, 129.28, 128.88, 64.96, 64.35, 58.76, 31.96, 31.53, 20.72, 19.73, 19.64. HRMS APCI: $[M-N2+H]^+$ calcd. for C14H19N4O2S: 307.1223; Found 307.1222

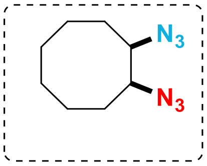
Prepared according to General Procedure A and obtained as colorless oil. ^{1}H NMR (600 MHz, Chloroform-*d*) δ 3.70-3.66 (m, 0.36H), 3.49 – 3.40 (m, 1.64H), 1.89-1.83 (m, 2H), 1.81 – 1.66 (m, 4H), 1.64 – 1.55 (m, 2H), 1.51 – 1.45 (m, 2H), 1.41 – 1.29 (m, 2H). ^{13}C NMR (151 MHz, $CDCl_3$) δ 66.60, 63.39, 29.27, 28.12, 26.46, 25.57, 24.76, 23.48. HRMS APCI: $[M-N2+H]^+$ calcd. for C8H15N4: 167.1291; Found 167.1286

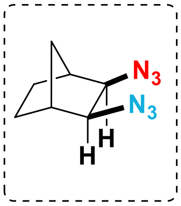
Prepared according to General Procedure A and obtained as colorless oil. ^{1}H NMR (600 MHz, Chloroform-*d*) δ 3.70-3.63 (m, 0.27H), 3.62-3.57 (m, 1.72H), 3.20-3.17 (m, 0.28H), 2.48-2.43 (m, 0.33H), 2.38-2.34 (m, 1.72H), 2.33-2.30 (m, 0.33H), 1.78-1.74 (m, 0.88H), 1.68 – 1.63 (m, 0.95H), 1.61-1.57 (m, 1.89H), 1.40-1.35 (m, 0.64H), 1.27-1.25 (m, 0.35H), 1.23 – 1.15 (m, 2.58H). ^{13}C NMR (151 MHz, $CDCl_3$) δ 70.45, 69.99, 67.49, 41.96, 41.53, 40.51, 35.00, 33.36, 26.27, 25.79, 20.85. HRMS APCI: $[M-N2+H]^+$ calcd. for C7H11N4: 151.0978; Found 151.0973

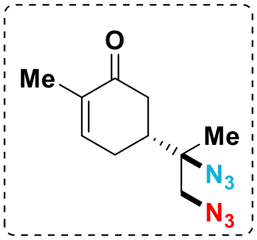
Prepared according to General Procedure A and obtained as colorless oil. ^{1}H NMR (600 MHz, Chloroform-*d*) δ 6.77-6.67 (m, 1H), 3.46 – 3.33 (m, 2H), 2.59 – 2.49 (m, 1H), 2.42-2.34 (m, 1H), 2.33 – 2.22 (m, 3H), 1.78 (s, 3H), 1.42-1.33 (m, 3H). ^{13}C NMR (151 MHz, $CDCl_3$) δ 198.43, 198.31, 143.99, 143.58, 135.67, 135.53, 64.74, 64.68, 57.62, 57.50, 41.29, 41.25, 39.06, 38.83, 26.98, 26.69, 18.65, 18.49, 15.57, 15.56. HRMS APCI: $[M-N2+H]^+$ calcd. for C0H15N4O: 207.1240; Found 207.1232

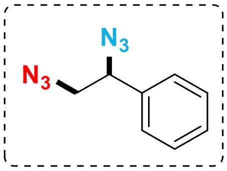
Prepared according to General Procedure A and obtained as colorless oil. ^{1}H NMR (600 MHz, Chloroform-*d*) δ 7.40 – 7.29 (m, 3H), 7.29 – 7.24 (m, 2H), 4.61 (dd, *J* = 8.4, 4.8 Hz, 1H), 3.44 (dd, *J* = 12.8, 8.5 Hz, 1H), 3.37 (dd, *J* = 12.8, 4.8 Hz, 1H). ^{13}C NMR (151 MHz, $CDCl_3$) δ ^{13}C NMR (151 MHz, $CDCl_3$) δ 136.33, 129.14, 129.10, 126.98, 65.56, 55.98.HRMS APCI: $[M-N2+H]^+$ calcd. for C8H9N4: 161.0822; Found 161.0821

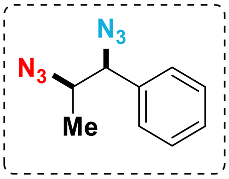
Prepared according to General Procedure A and obtained as colorless oil. ^{1}H NMR (600 MHz, Chloroform-*d*) δ 7.44 – 7.29 (m, 5H), 4.52 (d, *J* = 5.8 Hz, 0.66H), 4.37 (d, *J* = 7.7 Hz, 0.34H), 3.74 – 3.62 (m, 1H), 1.26 (dd, *J* = 6.6, 0.7 Hz, 2H), 1.10 (dd, *J* = 6.7, 0.7 Hz, 1H). ^{13}C NMR (151 MHz, $CDCl_3$) δ 136.27, 136.04, 129.00,

128.85, 128.81, 127.56, 70.77, 69.63, 61.51, 61.02, 16.76, 15.03. HRMS APCI: $[M-N2+H]^+$ calcd. for C9H11N4: 175.0978; Found 175.0977

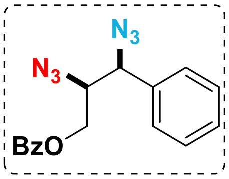

Prepared according to General Procedure A and obtained as colorless oil. ^{1}H NMR (600 MHz, Chloroform-*d*) δ 8.12-7.97 (m, 2H), 7.63 – 7.56 (m, 1H), 7.49 – 7.34 (m, 7H), 4.73-4.65 (m, 1H), 4.62-4.54 (m, 0.62H), 4.42 – 4.31 (m, 1H), 4.13-4.08 (m, 0.45H), 4.03-3.98 (m, 0.59H), 3.97-3.91 (m, 0.41H). ^{13}C NMR (151 MHz, $CDCl_3$) δ 166.02, 165.90, 135.27, 135.09, 133.49, 133.43, 129.77, 129.73, 129.45, 129.33, 129.29, 129.17, 129.14, 128.57, 128.54, 127.74, 127.41, 66.71, 65.69, 64.59, 64.30, 64.13, 63.98. HRMS APCI: $[M-N2+H]^+$ calcd. for C16H15N4O2: 295.1190; Found 295.1186

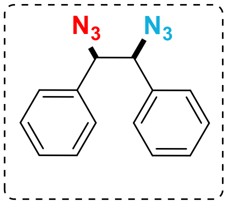

Prepared according to General Procedure A and obtained as colorless oil. ^{1}H NMR (600 MHz, Chloroform-*d*) δ 7.36 – 7.28 (m, 2H), 7.21-7.16 (m, 5H), 7.03 – 6.96 (m, 3H), 4.62 (s, 0.56H), 4.57 (s, 1.44H). ^{13}C NMR (151 MHz, $CDCl_3$) δ 135.85, 135.76, 129.00, 128.74, 128.70, 128.59, 127.97, 127.68, 70.74, 69.65. HRMS APCI: $[M-N2+H]^+$ calcd. for C14H13N4: 237.1135; Found 237.1133

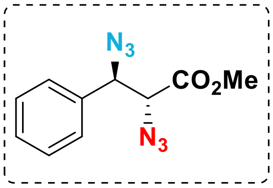

Prepared according to General Procedure A and obtained as colorless oil. ^{1}H NMR (600 MHz, Chloroform-*d*) δ 7.51 – 7.34 (m, 5H), 5.06 (d, *J* = 5.8 Hz, 1H), 4.02 (d, *J* = 5.7 Hz, 1H), 3.75 (s, 3H). ^{13}C NMR (151 MHz, $CDCl_3$) δ 168.05, 134.89, 129.32, 129.04, 127.43, 66.31, 66.26, 53.05. HRMS APCI: $[M-N2+H]^+$ calcd. for C10H11N4O2: 219.0877; Found 219.0868

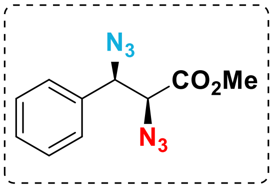

Prepared according to General Procedure A and obtained as colorless oil. ^{1}H NMR (600 MHz, Chloroform-*d*) δ 7.47 – 7.36 (m, 5H), 4.91 (d, *J* = 8.1 Hz, 1H), 4.10 (d, *J* = 8.1 Hz, 1H), 3.83 (s, 3H). ^{13}C NMR (151 MHz, $CDCl_3$) δ 168.25, 134.43, 129.54, 129.09, 127.83, 65.54, 65.46, 53.05. HRMS APCI: $[M-N2+H]^+$ calcd. for C10H11N4O2: 219.0877; Found 219.0868

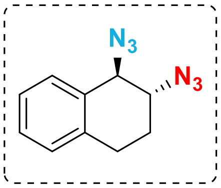

Prepared according to General Procedure A and obtained as colorless oil. ^{1}H NMR (600 MHz, Chloroform-*d*) δ 7.37 (dd, *J* = 5.5, 3.6 Hz, 1H), 7.32 – 7.21 (m, 2H), 7.15 (dd, *J* = 5.4, 3.6 Hz, 1H), 4.43 (d, *J* = 6.6 Hz, 1H), 3.93-3.80 (m, 1H), 3.01 – 2.78 (m, 2H), 2.37-2.16 (m, 1H), 2.09-1.88 (m, 1H). ^{13}C NMR (151 MHz, $CDCl_3$)

δ 135.66, 131.51, 129.01, 128.99, 128.55, 126.73, 63.49, 61.54, 25.98, 25.11. HRMS APCI: $[M-N2+H]^+$ calcd. for C10H11N4: 187.0978; Found 187.0974

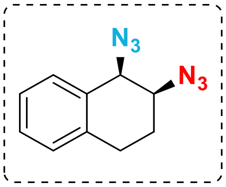

Prepared according to General Procedure A and obtained as colorless oil. ^{1}H NMR (600 MHz, Chloroform-*d*) δ 7.32 – 7.19 (m, 4H), 4.65 (d, *J* = 3.7 Hz, 1H), 3.81 (dt, *J* = 11.5, 3.5 Hz, 1H), 3.13-3.01 (m, 1H), 2.96-2.82 (m, 1H), 2.31-2.16 (m, 1H), 2.15 – 2.01 (m, 1H). ^{13}C NMR (151 MHz, $CDCl_3$) δ 135.35, 131.64, 129.61, 129.33, 129.14, 126.59, 62.47, 59.68, 27.37, 22.80. HRMS APCI: $[M-N2+H]^+$ calcd. for C10H11N4: 187.0978; Found 187.0974

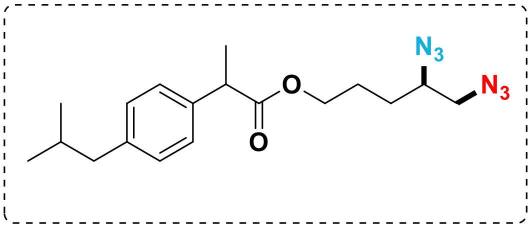

Prepared according to General Procedure A and obtained as colorless oil. ^{1}H NMR (600 MHz, Chloroform-*d*) δ 7.19 (d, *J* = 7.9 Hz, 2H), 7.10 (d, *J* = 7.9 Hz, 2H), 4.18 – 4.01 (m, 2H), 3.69 (q, *J* = 7.2 Hz, 1H), 3.42 – 3.33 (m, 1H), 3.29 (dd, *J* = 12.7, 4.0 Hz, 1H), 3.23 (dd, *J* = 12.7, 7.3 Hz, 1H), 2.45 (d, *J* = 7.2 Hz, 2H), 1.88-1.81 (m, 1H), 1.74 (ddt, *J* = 14.7, 9.5, 8.5, 6.5 Hz, 1H), 1.65 (ddt, *J* = 14.6, 7.6, 4.2 Hz, 1H), 1.49 (d, *J* = 7.2 Hz, 3H), 1.46 – 1.39 (m, 2H), 0.90 (d, *J* = 6.6 Hz, 6H). ^{13}C NMR (151 MHz, $CDCl_3$) δ 174.68, 140.65, 137.68, 137.66, 129.36, 127.13, 63.78, 63.65, 61.42, 61.39, 54.75, 54.73, 45.11, 45.10, 44.98, 30.20, 28.27, 28.14, 25.01, 24.97, 22.37, 18.32, 18.28. HRMS APCI: $[M-N2+H]^+$ calcd. for C18H27N4O2: 331.2129; Found 331.2126

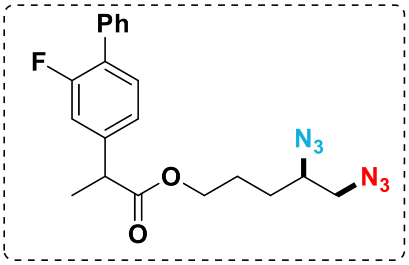

Prepared according to General Procedure A and obtained as colorless oil. ^{1}H NMR (600 MHz, Chloroform-*d*) δ 7.55-7.52 (m, 2H), 7.48 – 7.34 (m, 4H), 7.17 – 7.09 (m, 2H), 4.19 – 4.09 (m, 2H), 3.76 (q, *J* = 7.2 Hz, 1H), 3.45 – 3.38 (m, 1H), 3.33 (dd, *J* = 12.6, 4.1 Hz, 1H), 3.27 (dd, *J* = 12.7, 7.2 Hz, 1H), 1.85 – 1.74 (m, 1H), 1.73-1.65 (m, 1H), 1.54 (d, *J* = 7.2 Hz, 3H), 1.52-1.46 (m, 2H). ^{13}C NMR (151 MHz, $CDCl_3$) δ 173.91, 159.66 (d, *J* = 248.5 Hz), 141.70 (d, *J* = 7.9 Hz), 135.35, 130.84 (d, *J* = 3.9 Hz), 128.90 (d, *J* = 3.0 Hz), 128.49, 127.88 (d, *J* = 13.5 Hz), 127.73, 123.53 (d, *J* = 3.3 Hz), 115.20 (d, *J* = 23.5 Hz), 64.11 (d, *J* = 14.2 Hz), 61.41 (d, *J* = 3.7 Hz), 54.75, 44.99 (d, *J* = 1.8 Hz), 28.26 (d, *J* = 13.6 Hz), 25.03 (d, *J* = 5.4 Hz), 18.22 (d, *J* = 4.7 Hz). ^{19}F NMR (565 MHz, Chloroform-*d*) δ -117.52. HRMS APCI: $[M-N2+H]^+$ calcd. for C20H22FN4O2: 369.1721; Found 369.1719

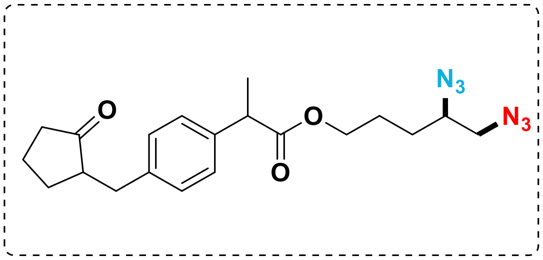

Prepared according to General Procedure A and obtained as colorless oil. ^{1}H NMR (600 MHz, Chloroform-*d*) δ 7.21 (d, *J* = 7.7 Hz, 2H), 7.13 (d, *J* = 7.7 Hz, 2H), 4.15-4.05 (m, 2H), 3.69 (q, *J* = 7.2 Hz, 1H), 3.43-3.37 (m, 1H), 3.31 (dd, *J* = 12.7, 4.0 Hz, 1H), 3.25 (dd, *J* = 12.7, 7.2 Hz, 1H), 3.13 (dd, *J* = 13.9, 4.2 Hz, 1H), 2.51 (dd, *J* = 13.9, 9.5 Hz, 1H), 2.42 – 2.26 (m, 2H), 2.15-2.04 (m, 2H), 2.00 – 1.93 (m, 1H), 1.79 – 1.41 (m, 9H). ^{13}C NMR (151 MHz, $CDCl_3$) δ 220.27, 174.58, 139.01, 138.28, 129.18, 127.52, 63.84 (d, *J* = 15.6 Hz), 61.44 (d, *J* = 3.4 Hz), 54.74, 51.01, 45.11, 38.20, 35.20, 29.29, 28.25 (d, *J* = 14.9 Hz), 25.03 (d, *J* = 4.8 Hz), 20.56, 18.38 (d, *J* = 4.5 Hz). HRMS APCI: $[M\text{-}N2+H]^+$ calcd. for C20H27N4O3: 371.2078; Found 371.2075

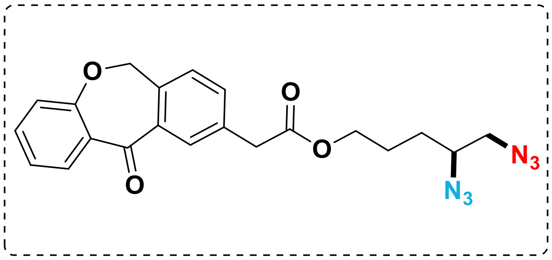

Prepared according to General Procedure A and obtained as colorless oil. ^{1}H NMR (600 MHz, Chloroform-*d*) δ 8.05 (d, *J* = 2.4 Hz, 1H), 7.82 (dd, *J* = 7.7, 1.3 Hz, 1H), 7.50 (td, *J* = 7.4, 1.4 Hz, 1H), 7.41 (td, *J* = 7.6, 1.2 Hz, 1H), 7.35 (dd, *J* = 8.4, 2.5 Hz, 1H), 7.30 (dd, *J* = 7.5, 1.2 Hz, 1H), 6.97 (d, *J* = 8.4 Hz, 1H), 5.12 (s, 2H), 4.11-4.03 (m, 2H), 3.58 (s, 2H), 3.42-3.37 (m, 1H), 3.33 (dd, *J* = 12.7, 4.0 Hz, 1H), 3.25 (dd, *J* = 12.7, 7.3 Hz, 1H), 1.79-1.72 (m, 1H), 1.70 – 1.61 (m, 1H), 1.56 – 1.42 (m, 2H). ^{13}C NMR (151 MHz, $CDCl_3$) δ 190.90, 171.38, 160.53, 140.41, 136.35, 135.53, 132.88, 132.41, 129.48, 129.33, 127.89, 127.70, 125.16, 121.15, 73.65, 64.19, 61.55, 54.83, 40.28, 28.40, 25.11. HRMS APCI: $[M +H]^+$ calcd. for C21H21N6O4: 421.1619; Found 421.1615

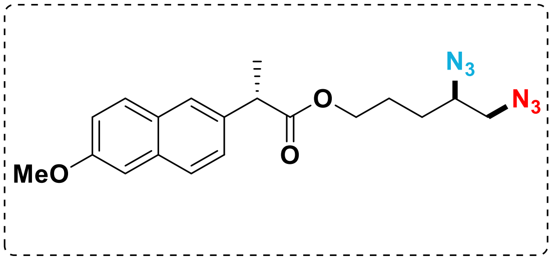

Prepared according to General Procedure A and obtained as colorless oil. ^{1}H NMR (600 MHz, Chloroform-*d*) δ 7.75 – 7.60 (m, 3H), 7.42-7.37 (m, 1H), 7.21 – 7.07 (m, 2H), 4.20 – 4.00 (m, 2H), 3.91 (s, 3H), 3.85 (q, *J* = 7.1 Hz, 1H), 3.30-3.22 (m, 1H), 3.15 – 2.97 (m, 2H), 1.75-1.68 (m, 1H), 1.62-1.54 (d, *J* = 7.1 Hz, 4H), 1.35-1.28 (m, 2H). ^{13}C NMR (151 MHz, $CDCl_3$) δ 174.61, 157.73 (d, *J* = 1.8 Hz), 135.66 (d, *J* = 5.4 Hz), 133.71, 129.24 (d, *J* = 2.7 Hz), 128.89, 127.22, 126.19 (d, *J* = 3.5 Hz), 125.99, 119.18 (d, *J* = 3.8 Hz), 105.54, 63.83 (d, *J* = 27.8 Hz), 61.36 (d, *J* = 3.9 Hz), 55.35, 54.66, 45.47 (d, *J* = 2.4 Hz), 28.19 (d, *J* = 24.4 Hz), 25.04 (d, *J* = 8.8 Hz), 18.20 (d, *J* = 7.6 Hz). HRMS APCI: $[M\text{-}N2+H]^+$ calcd. for C19H23N4O3: 355.1765; Found 355.1762

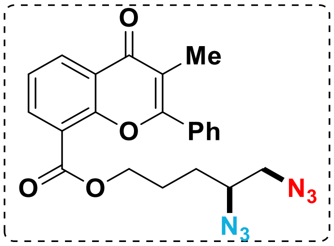

Prepared according to General Procedure A and obtained as colorless oil. ^{1}H NMR (600 MHz, Chloroform-*d*) δ 8.48 (dd, *J* = 7.9, 1.8 Hz, 1H), 8.27 (dd, *J* = 7.5, 1.8 Hz, 1H), 7.81 – 7.73 (m, 2H), 7.56 (dt, *J* = 4.8, 2.3 Hz, 3H), 7.46 (t, *J* = 7.7 Hz, 1H), 4.46 – 4.30 (m, 2H), 3.41-3.30 (m, 1H), 3.29 – 3.18 (m, 2H), 2.23 (s, 3H), 1.92 – 1.86 (m, 1H), 1.85 – 1.76 (m, 1H), 1.59 – 1.44 (m, 2H). ^{13}C NMR (151 MHz, $CDCl_3$) δ 178.20, 164.50, 160.97, 154.44, 136.20, 133.11, 130.98, 130.55, 129.31, 128.48, 124.07, 123.33, 120.46, 117.81, 64.73, 61.48, 54.76, 28.42, 25.17, 11.74. HRMS APCI: $[M-N2+H]^+$ calcd. for C22H21N4O4: 405.1557; Found 405.1547

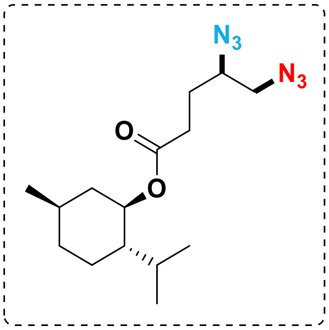

Prepared according to General Procedure A and obtained as colorless oil. ^{1}H NMR (600 MHz, Chloroform-*d*) δ 4.74-4.67 (m, 1H), 3.60-3.54 (m, 1H), 3.45 (dd, *J* = 12.7, 4.1 Hz, 1H), 3.36 (dd, *J* = 12.8, 7.4 Hz, 1H), 2.49 – 2.38 (m, 2H), 2.00-1.95 (m, 1H), 1.94 – 1.87 (m, 1H), 1.87 – 1.81 (m, 1H), 1.80 – 1.72 (m, 1H), 171-1.66 (m, 2H), 1.53-1.45 (m, 1H), 1.41-1.35 (m, 1H), 1.09-1.02 (m, 1H), 0.94 (m, 1H), 0.93 – 0.83 (m, 7H), 0.76 (dd, *J* = 7.0, 2.1 Hz, 3H). ^{13}C NMR (151 MHz, $CDCl_3$) δ 172.01, 74.67 (d, *J* = 2.8 Hz), 54.90, 61.24 (d, *J* = 3.2 Hz), 46.98 (d, *J* = 2.0 Hz), 40.90 (d, *J* = 5.0 Hz), 34.19, 31.39, 30.68 (d, *J* = 1.9 Hz), 27.13 (d, *J* = 4.9 Hz), 26.35 (d, *J* = 5.5 Hz), 23.39 (d, *J* = 2.1 Hz), 22.00, 20.74 (d, *J* = 2.2 Hz), 16.29 (d, *J* = 2.7 Hz). HRMS APCI: $[M-N2+H]^+$ calcd. for C15H27N4O2: 295.2129; Found 295.2127

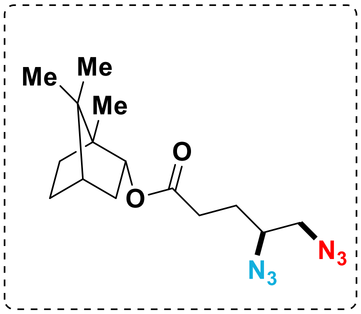

Prepared according to General Procedure A and obtained as colorless oil. ^{1}H NMR (600 MHz, Chloroform-*d*) δ 4.91 (dt, *J* = 10.0, 2.9 Hz, 1H), 3.61-3.57 (m, 1H), 3.50 – 3.43 (m, 1H), 3.39-3.35 (m, 1H), 2.55 – 2.44 (m, 2H), 2.42 – 2.32 (m, 1H), 1.94-1.88 (m, 2H), 1.81 – 1.72 (m, 2H), 1.69 (t, *J* = 4.5 Hz, 1H), 1.35-1.29 (m, 1H), 1.28 – 1.20 (m, 1H), 0.99-0.94 (m, 1H), 0.91 (s, 3H), 0.88 (s, 3H), 0.83 (d, *J* = 2.4 Hz, 3H). ^{13}C NMR (151 MHz, $CDCl_3$) δ 172.78, 80.42, 61.24, 54.91, 48.78, 47.85, 44.84, 36.80, 30.68, 28.05, 28.03, 27.15, 27.13, 27.11, 19.71, 18.84, 13.57. HRMS APCI: $[M-N2+H]^+$ calcd. for C15H25N4O2: 293.1972; Found 293.1971

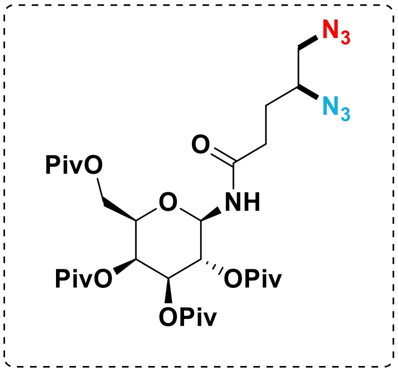

Prepared according to General Procedure A and obtained as colorless oil. ^{1}H NMR (600 MHz, Chloroform-*d*) δ 6.44 – 6.12 (m, 1H), 5.49 – 5.39 (m, 1H), 5.33 – 5.23 (m, 2H), 5.15-5.09 (m, 1H), 4.22 – 4.07 (m, 2H), 4.05 – 3.94 (m, 1H), 3.62 – 3.51 (m, 1H), 3.50-3.44 (m, 1H), 3.41-3.34 (m, 1H), 2.50 – 2.21 (m, 2H), 1.97-1.85 (m, 1H), 1.77-1.65 (m, 1H), 1.28 – 1.11 (m, 36H). ^{13}C NMR (151 MHz, $CDCl_3$) δ 178.83 (d, *J* = 5.1 Hz), 177.83, 176.96, 176.69 (d, *J* = 2.3 Hz), 171.47, 78.42, 72.66 (d, *J* = 4.8 Hz), 70.75, 68.26 (d, *J* = 7.1 Hz), 66.68, 60.87, 60.69 (d, *J* = 3.4 Hz), 54.90 (d, *J* = 13.7 Hz), 39.04 (d, *J* = 8.1 Hz), 38.74 (d, *J* = 6.9 Hz), 31.96 (d, *J* = 34.9 Hz), 27.18, 27.06 (d, *J* = 3.8 Hz), 26.97 (d, *J* = 3.8 Hz). HRMS APCI: $[M +H]^+$ calcd. for C31H52N7O10: 682.3770; Found 682.3760

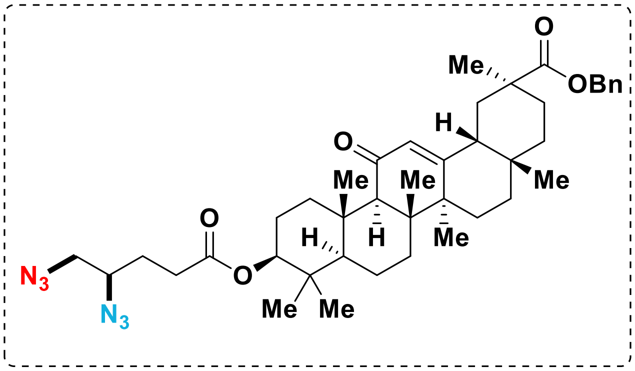

Prepared according to General Procedure A and obtained as colorless oil. ^{1}H NMR (600 MHz, Chloroform-*d*) δ 7.48 – 7.28 (m, 5H), 5.55 (s, 1H), 5.20 (d, *J* = 12.2 Hz, 1H), 5.09 (d, *J* = 12.2 Hz, 1H), 4.54 (dd, *J* = 12.1, 4.6 Hz, 1H), 3.61-3.54 (m, 1H), 3.45 (dd, *J* = 12.9, 3.3 Hz, 1H), 3.37 (dd, *J* = 12.8, 7.4 Hz, 1H), 2.87 – 2.77 (m, 1H), 2.48 (ddd, *J* = 9.0, 6.1, 3.6 Hz, 2H), 2.34 (s, 1H), 2.02 (dq, *J* = 14.5, 8.7, 6.8 Hz, 3H), 1.97 – 1.86 (m, 2H), 1.84 – 1.60 (m, 7H), 1.49 – 1.28 (m, 9H), 1.16 (d, *J* = 2.5 Hz, 6H), 1.11 (s, 3H), 1.07-0.96 (m, 2H), 0.91 – 0.87 (m, 7H), 0.73 (s, 3H). ^{13}C NMR (151 MHz, $CDCl_3$) δ 199.96, 176.20, 172.20 (d, *J* = 2.0 Hz), 169.13 (d, *J* = 2.2 Hz), 136.13, 128.62, 128.45, 128.30, 128.25, 81.16, 66.23, 61.65, 61.20, 55.00, 54.88, 48.22, 45.35, 43.99, 43.17, 41.05, 38.75, 38.08, 37.64, 36.91, 32.67, 31.78, 31.16, 30.74 (d, *J* = 12.4 Hz), 29.70, 28.41, 28.28, 28.12, 27.13 (d, *J* = 8.0 Hz), 26.42 (d, *J* = 10.4 Hz), 23.59 (d, *J* = 6.3 Hz), 23.30, 18.66, 17.37, 16.77, 16.41. HRMS APCI: $[M +H]^+$ calcd. for C42H59N6O5: 727.4541; Found 727.4533

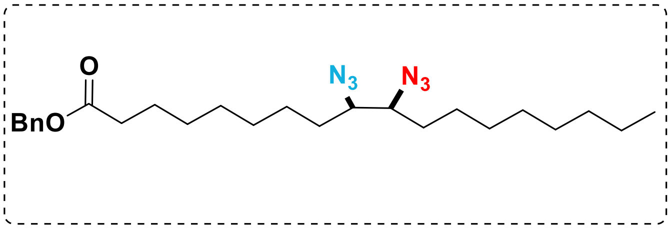

Prepared according to General Procedure A and obtained as colorless oil. ^{1}H NMR (600 MHz, Chloroform-*d*) δ 7.42 – 7.30 (m, 5H), 5.12 (s, 2H), 3.35-3.30 (m, 1H), 3.28-3.22 (m, 1H), 2.36 (t, *J* = 7.5 Hz, 2H), 1.70-1.63 (m, 3H), 1.58-1.50 (m, 3H), 1.48 – 1.42 (m, 1H), 1.39 – 1.21 (m, 19H), 0.89 (t, *J* = 6.9 Hz, 3H). ^{13}C NMR (151 MHz, $CDCl_3$) δ 173.62, 136.07, 128.55, 128.19, 66.10, 65.86, 65.82, 65.24, 65.20, 34.25, 31.82, 31.26, 31.24, 30.36, 30.28, 29.40, 29.35, 29.34, 29.19, 29.14, 29.04, 28.96, 26.31, 26.25, 26.19, 26.12, 24.85, 22.66, 14.12. HRMS APCI: $[M-N2+H]^+$ calcd. for C25H41N4O2: 429.3224; Found 429.3222

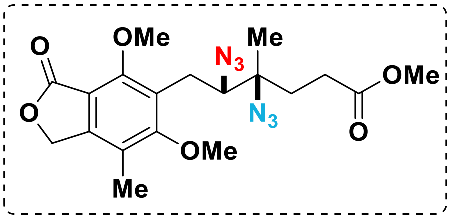

Prepared according to General Procedure A and obtained as colorless oil. ^{1}H NMR (600 MHz, Chloroform-*d*) δ 5.11 (s, 2H), 4.09 (s, 3H), 3.78 (s, 3H), 3.69-3.64 (m, 4H), 3.00 (dd, J = 13.5, 10.6 Hz, 0.78H), 2.89 (dd, J = 13.4, 11.0 Hz, 0.22H), 2.82 (dd, J = 13.5, 2.9 Hz, 0.78H), 2.74 (dd, J = 13.4, 2.8 Hz, 0.22H)., 2.48 – 2.38 (m, 2H), 2.15 (s, 3H), 2.07 – 1.89 (m, 2H), 1.37-1.32 (m, 3H). ^{13}C NMR (151 MHz, $CDCl_3$) δ 173.31, 168.76, 163.23, 157.01, 148.20, 124.81, 120.04, 112.35, 69.30, 68.83, 68.47, 66.12, 65.86, 62.84, 61.05, 51.98, 51.96, 31.97, 31.90, 28.70, 28.52, 25.17, 25.11, 19.46, 18.94, 11.77, 11.74. HRMS APCI: $[M-N2+H]^+$ calcd. for C19H25N4O6: 405.1769; Found 405.1755

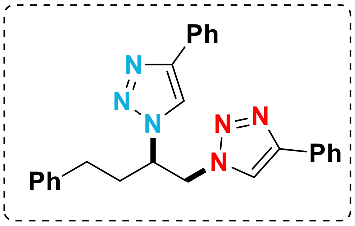

The compound characterization was reported in literature and prepared according to Derivatization Procedure A.[6] ^{1}H NMR (600 MHz, Chloroform-*d*) δ 7.78 – 7.68 (m, 2H), 7.66 – 7.59 (m, 2H), 7.44 – 7.36 (m, 3H), 7.34 – 7.27 (m, 6H), 7.25 – 7.21 (m, 2H), 7.14 – 7.10 (m, 2H), 5.05 – 4.91 (m, 3H), 2.75 – 2.61 (m, 2H), 2.61-2.50 (m, 1H), 2.46 – 2.32 (m, 1H). ^{13}C NMR (151 MHz, $CDCl_3$) δ 147.96, 147.65, 139.22, 129.89, 129.86, 128.89, 128.85, 128.81, 128.47, 128.42, 128.35, 126.77, 125.77, 125.73, 121.06, 120.67, 61.11, 53.87, 33.74, 31.59.

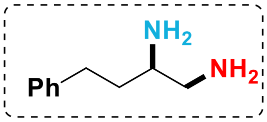

The compound characterization was reported in literature and prepared according to Derivatization Procedure B.[9] ^{1}H NMR (600 MHz, Chloroform-*d*) δ 7.31-7.27 (m, 2H), 7.23 – 7.15 (m, 3H), 2.81 – 2.73 (m, 2H), 2.73 – 2.60 (m, 2H), 2.55-2.45 (m, 1H), 1.81 – 1.67 (m, 1H), 1.61-1.51 (m, 1H). ^{13}C NMR (151 MHz, $CDCl_3$) δ 142.13, 128.42, 128.34, 125.85, 53.22, 48.71, 37.47, 32.62.

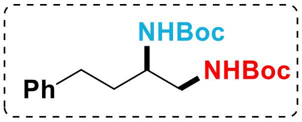

Prepared according to Derivatization Procedure C. ^{1}H NMR (600 MHz, Chloroform-*d*) δ 7.31 – 7.24 (m, 2H), 7.21-7.15 (m, 3H), 4.92-4.78 (m, 1H), 4.75-4.55 (m, 1H), 3.78-3.57 (m, 1H), 3.31 – 3.07 (m, 2H), 2.77 – 2.58 (m, 2H), 1.82-1.69 (m, 2H), 1.45 (s, 9H), 1.43 (s, 9H). ^{13}C NMR (151 MHz, $CDCl_3$) δ 156.60, 156.23, 141.53, 128.47, 128.39, 126.00, 79.42, 51.14, 44.82, 34.85, 32.27, 28.40. HRMS APCI: $[M+H]^+$ calcd. for C20H33N2O4: 365.2435; Found 365.2433

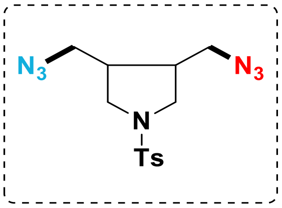

Prepared according to General Procedure A and obtained as colorless oil. ^{1}H NMR (600 MHz, Chloroform-*d*) δ 7.76 – 7.69 (m, 2H), 7.36 (d, J = 7.8 Hz, 2H), 3.45-3.38 (m, 2H), 3.34 – 3.27 (m, 2H), 3.25 – 3.21 (m, 0.61H), 3.18 – 3.13 (m, 1.48H), 3.13 – 3.08 (m, 1.38H), 3.04 – 3.00 (m, 0.62H), 2.46-2.43 (m, 3H), 2.43-2.36 (m, 1.43H), 2.14 – 2.09 (m, 0.58H). ^{13}C NMR (151 MHz, $CDCl_3$) δ 143.99, 143.91, 133.29, 132.60, 129.85, 129.81, 127.72, 127.50, 52.81, 50.59, 50.26, 49.58, 41.18, 39.79, 21.58, 21.55. HRMS APCI: $[M+H]^+$ calcd. for C13H18N7O2: 336.1237; Found 336.1233

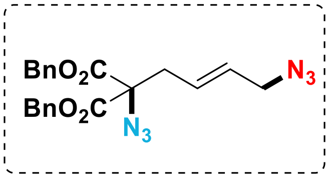

Prepared according to General Procedure A and obtained as colorless oil. ^{1}H NMR (600 MHz, Chloroform-*d*) δ 7.40 – 7.25 (m, 10H), 5.63-5.53 (m, 1H), 5.50 – 5.42 (m, 1H), 5.28 – 5.11 (m, 4H), 3.57 (d, J = 6.4 Hz, 2H), 2.69 (d, J = 7.0 Hz, 2H). ^{13}C NMR (151 MHz, $CDCl_3$) δ 166.57, 134.48, 128.81, 128.73, 128.71, 128.57, 127.53, 71.44, 68.48, 52.25, 36.89. HRMS APCI: $[M+H]^+$ calcd. for C21H21N6O4: 421.1619; Found 421.1616

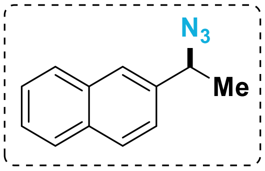

Prepared according to General Procedure A and obtained as colorless oil. ^{1}H NMR (600 MHz, Chloroform-*d*) δ 7.89 – 7.81 (m, 3H), 7.79 – 7.74 (m, 1H), 7.53 – 7.47 (m, 2H), 7.47-7.43 (m, 1H), 4.79 (q, J = 6.8 Hz, 1H), 1.62 (d, J = 6.8 Hz, 3H). ^{13}C NMR (151 MHz, $CDCl_3$) δ 138.20, 133.17, 133.07, 128.76, 128.01, 127.71, 126.42, 126.23, 125.30, 124.22, 61.29, 21.63. HRMS APCI: $[M+H]^+$ calcd. for C12H12N: 170.0964; Found 170.0961

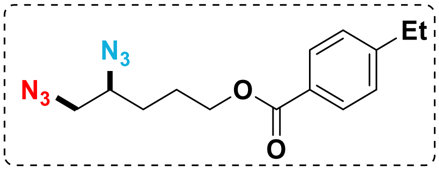

Prepared according to General Procedure A and obtained as colorless oil. ^{1}H NMR (600 MHz, Chloroform-*d*) δ 7.99 – 7.91 (m, 2H), 7.34 – 7.20 (m, 2H), 4.42 – 4.27 (m, 2H), 3.59 – 3.50 (m, 1H), 3.44 (dd, J = 12.7, 4.2 Hz, 1H), 3.37 (dd, J = 12.7, 7.3 Hz, 1H), 2.71 (q, J = 7.6 Hz, 2H), 2.00 – 1.90 (m, 1H), 1.90 – 1.80 (m, 1H), 1.77-1.63 (m, 2H), 1.26 (t, J = 7.6 Hz, 4H). ^{13}C NMR (151 MHz, $CDCl_3$) δ 166.60, 149.97, 129.70, 127.96, 127.53, 63.92, 61.61, 54.86, 28.97, 28.55, 25.34, 15.24. HRMS APCI: $[M+H]^+$ calcd. for C14H19N4O2: 275.1503; Found 275.1498

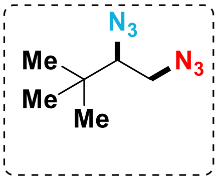

Prepared according to General Procedure A and obtained as colorless oil. ^{1}H NMR (600 MHz, Chloroform-*d*) δ 3.54 – 3.45 (m, 1H), 3.30 – 3.18 (m, 2H), 0.96 (s, 9H). ^{13}C NMR (151 MHz, $CDCl_3$) δ 72.78, 52.47, 35.02, 26.46. HRMS APCI: $[M-N2+H]^+$ calcd. for C6H13N4: 141.1135; Found 141.1133

III. Supplementary Figures

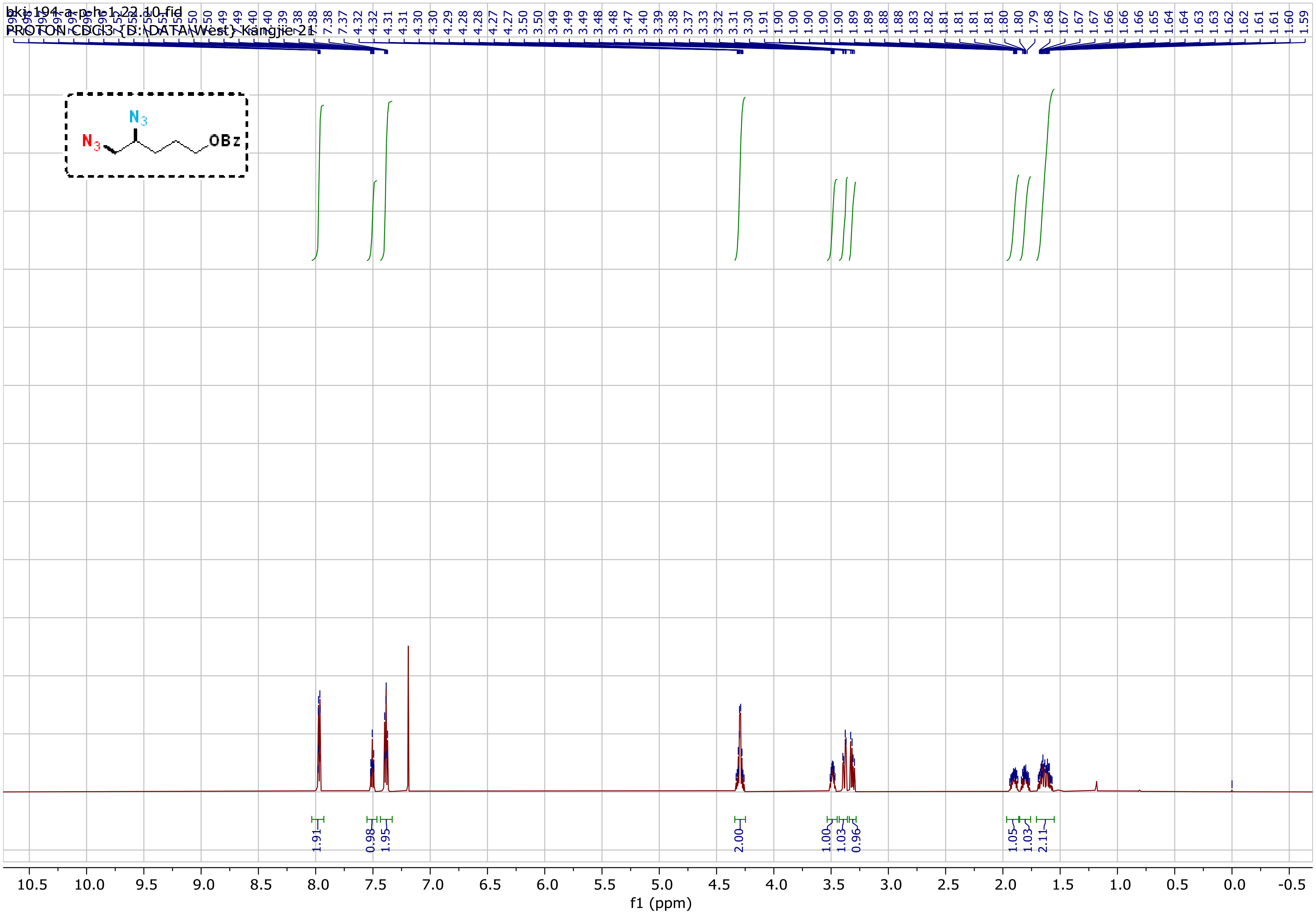

Supplementary Figure 1. ^{1}H NMR of **1** (600 MHz, $CDCl_3$)

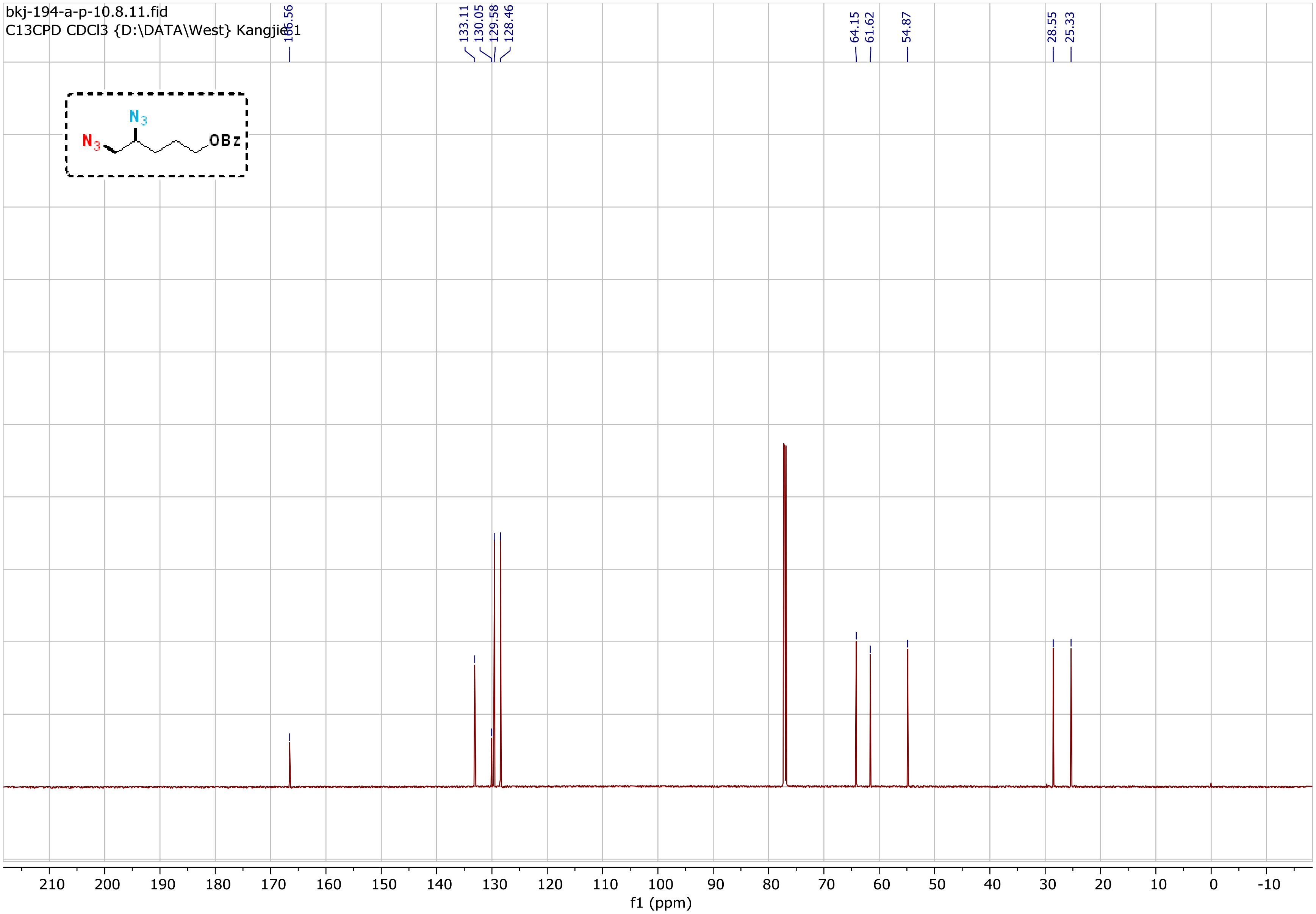

Supplementary Figure 2. ^{13}C NMR Spectrum of **1** (151 MHz, $CDCl_3$)

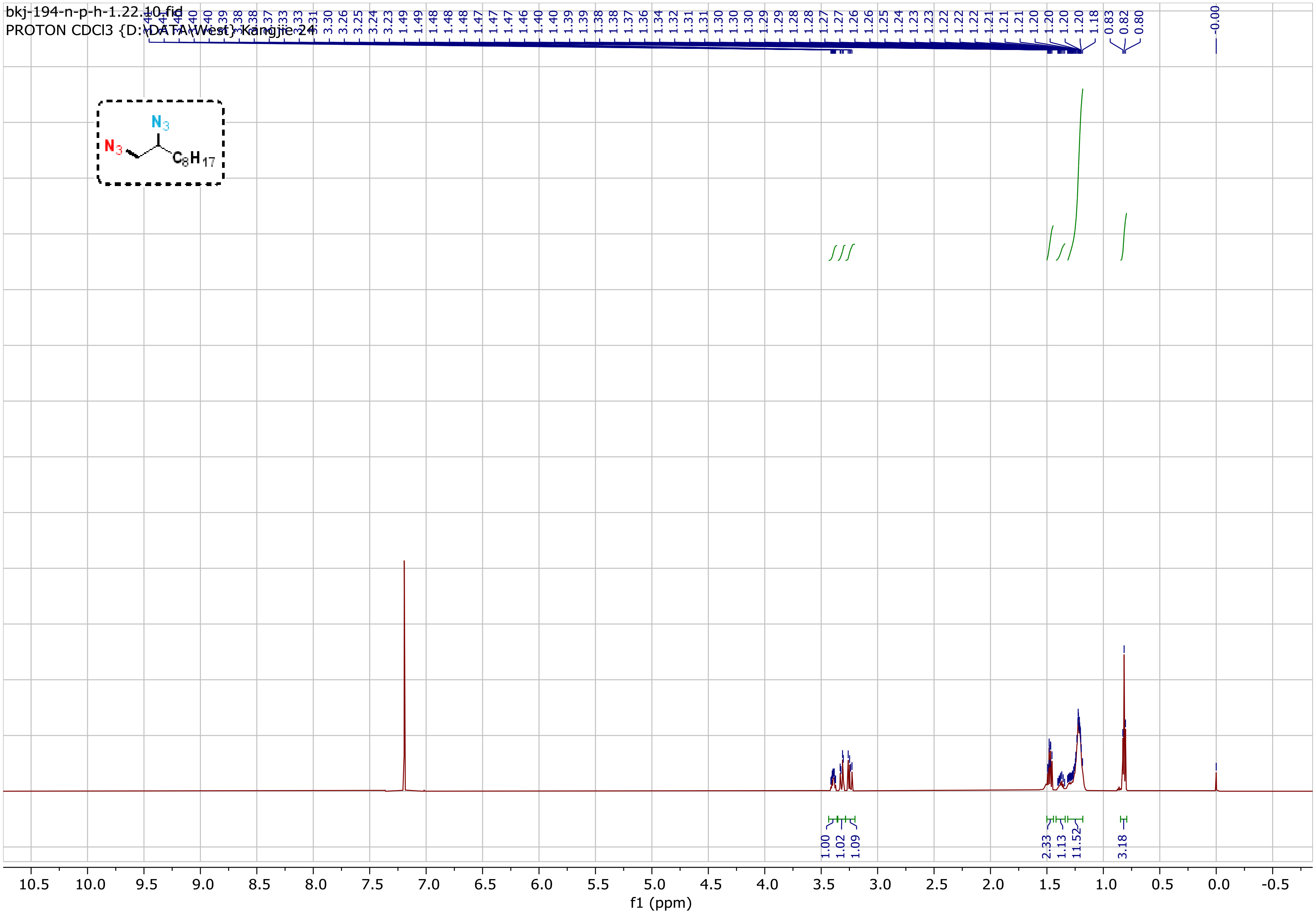

Supplementary Figure 3. ^{1}H NMR Spectrum of **2** (600 MHz, $CDCl_3$)

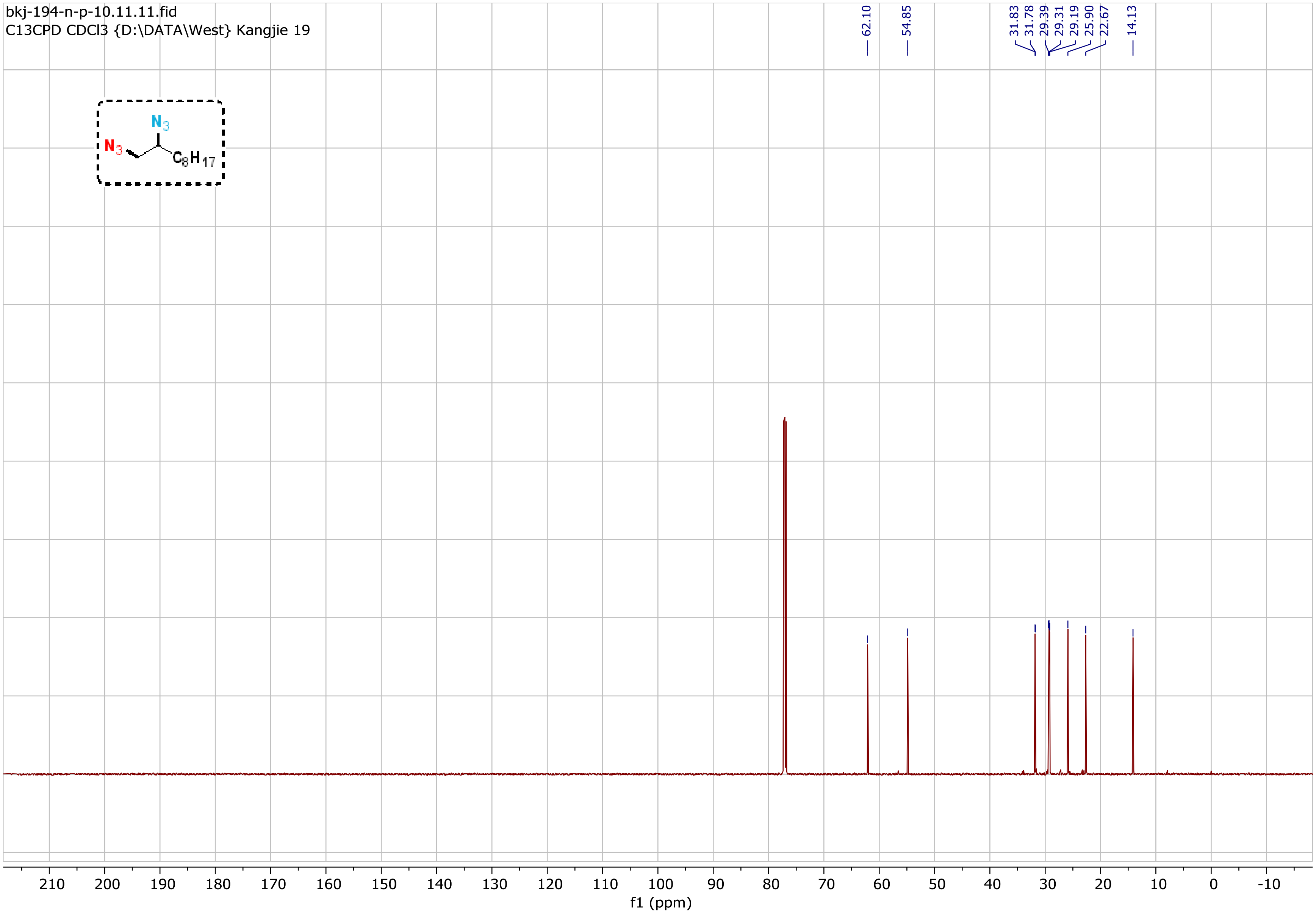

Supplementary Figure 4. ^{13}C NMR Spectrum of **2** (151 MHz, $CDCl_3$)

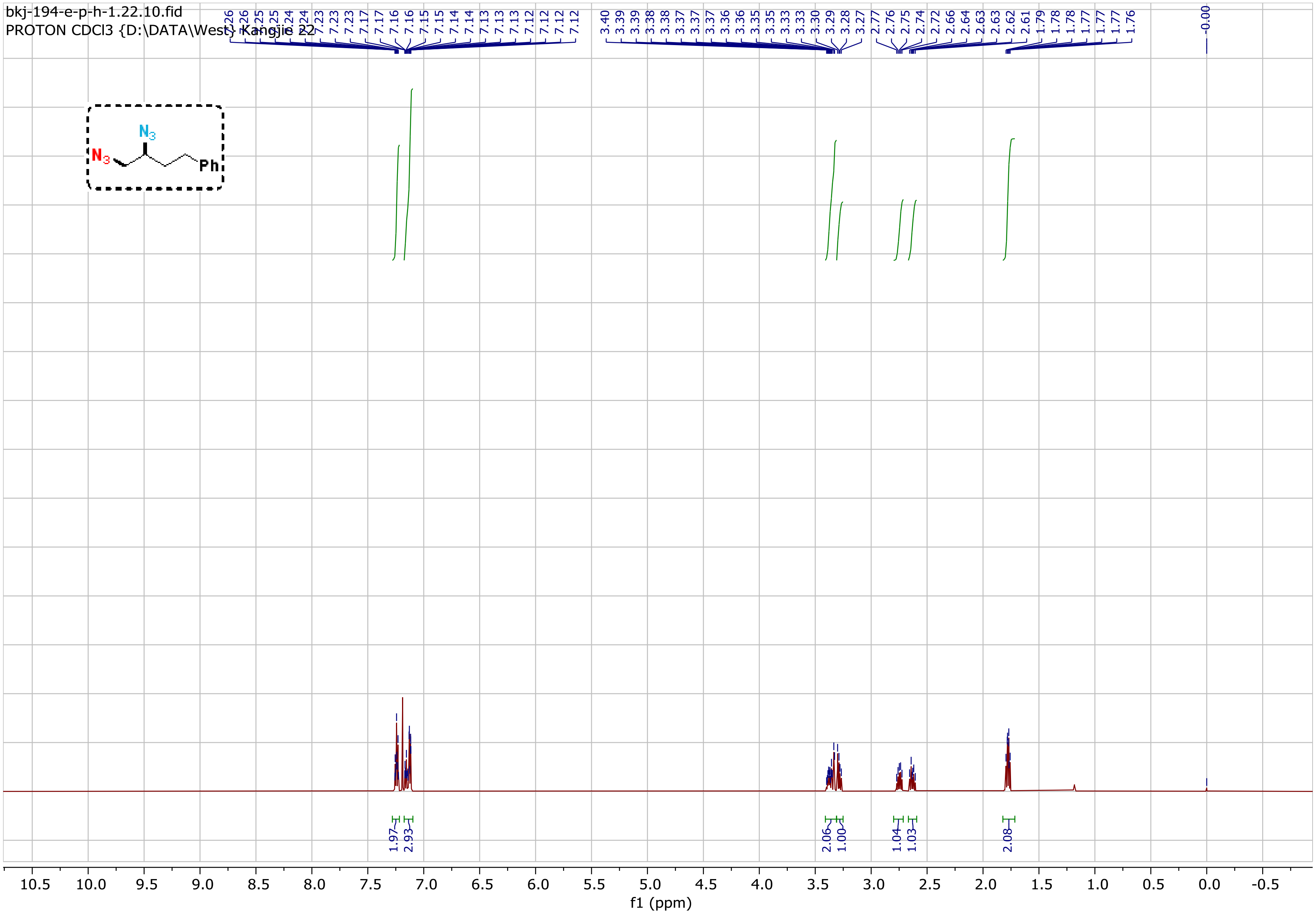

Supplementary Figure 5. ^{1}H NMR Spectrum of **3** (600 MHz, $CDCl_3$)

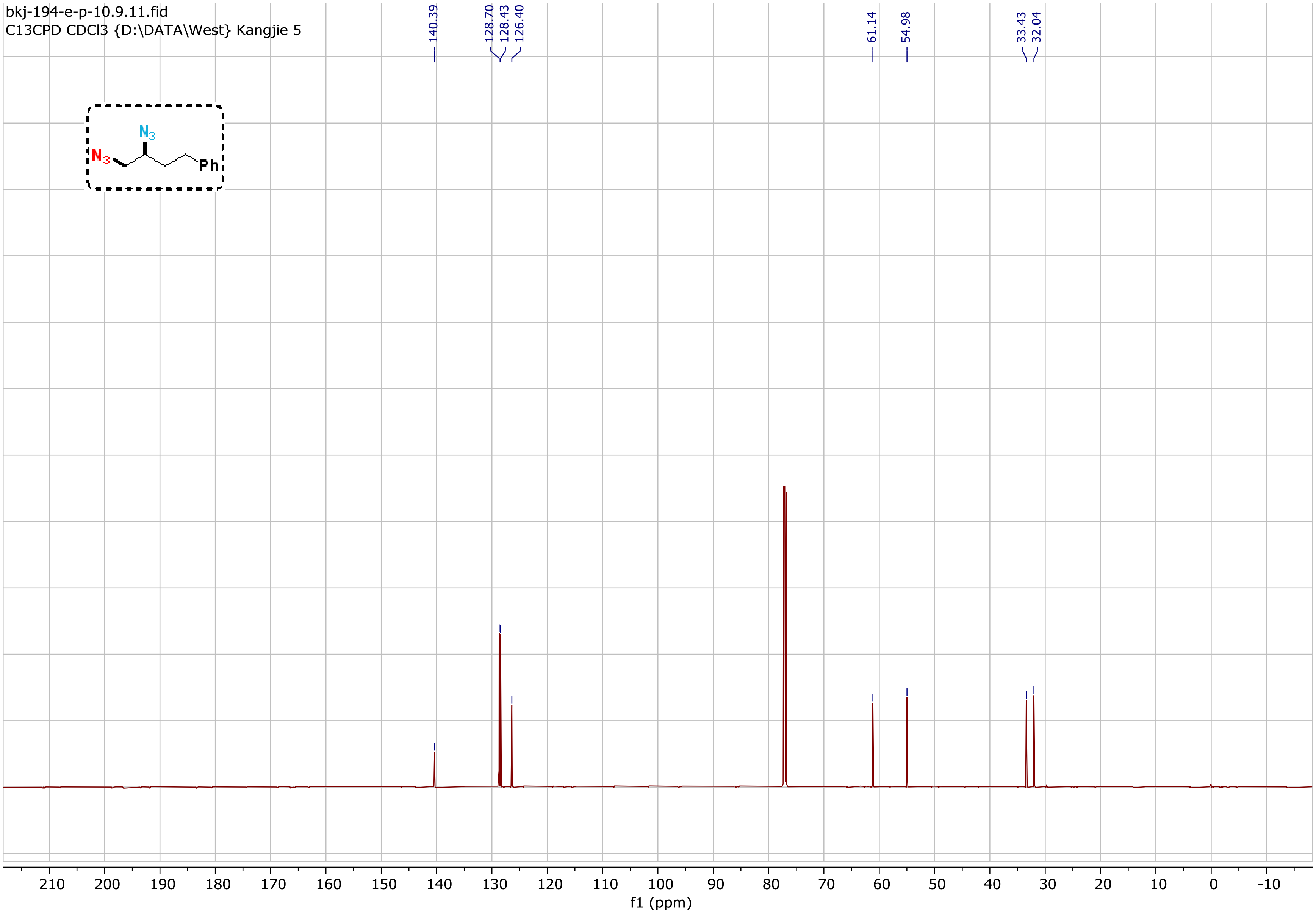

Supplementary Figure 6. ^{13}C NMR Spectrum of **3** (151 MHz, $CDCl_3$)

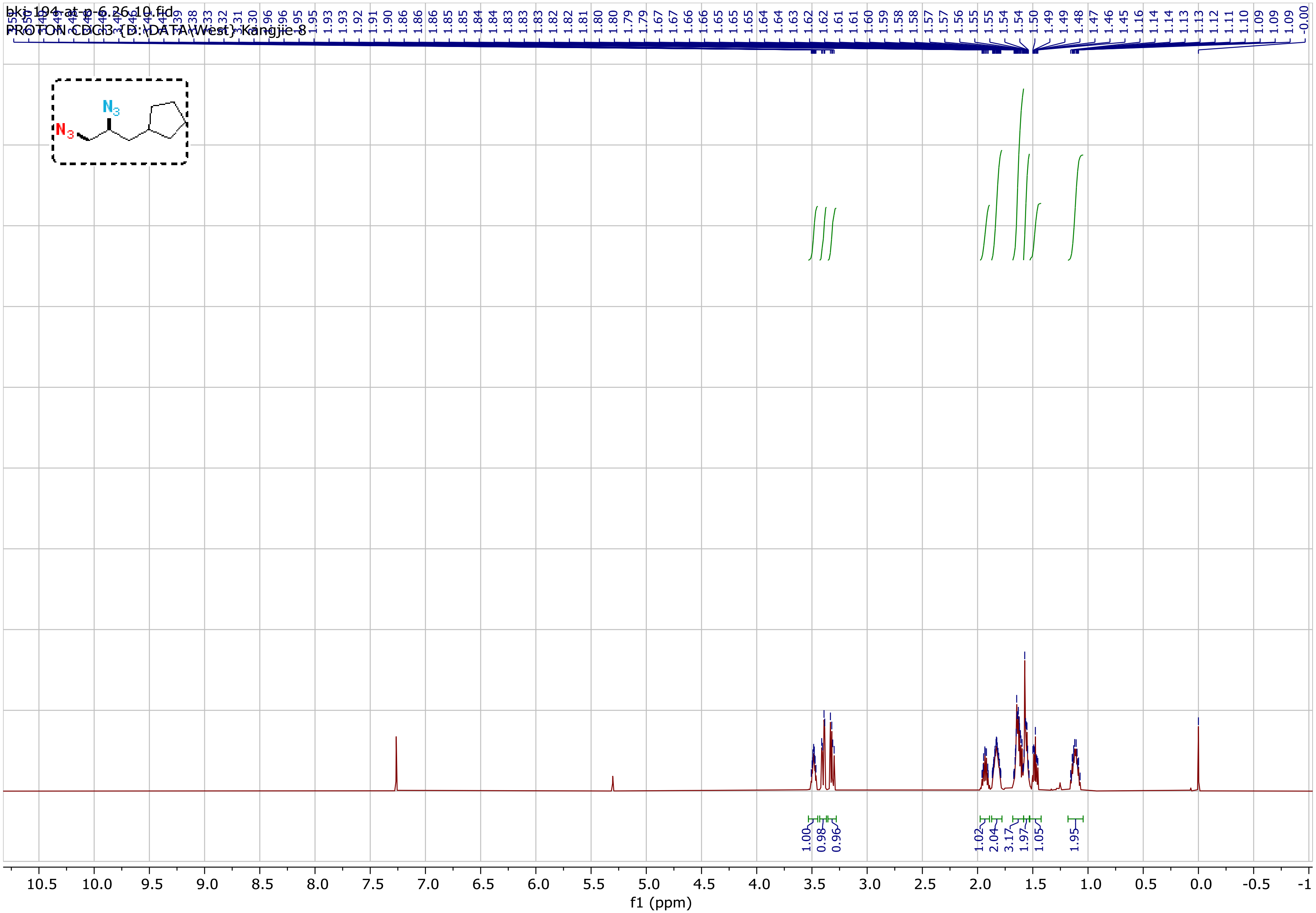

Supplementary Figure 7. ^{1}H NMR Spectrum of **4** (600 MHz, $CDCl_3$)

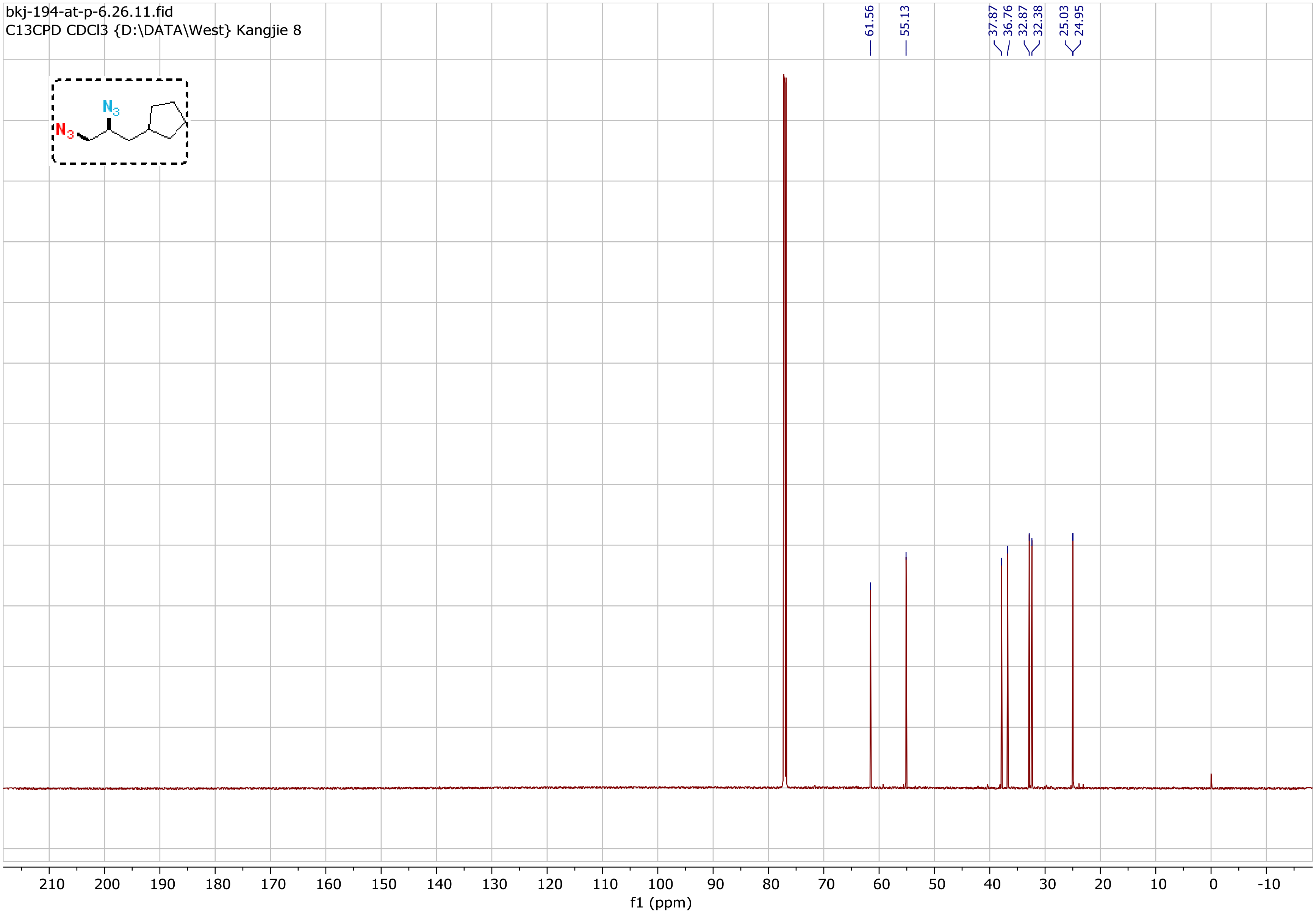

Supplementary Figure 8. ^{13}C NMR Spectrum of **4** (151 MHz, $CDCl_3$)

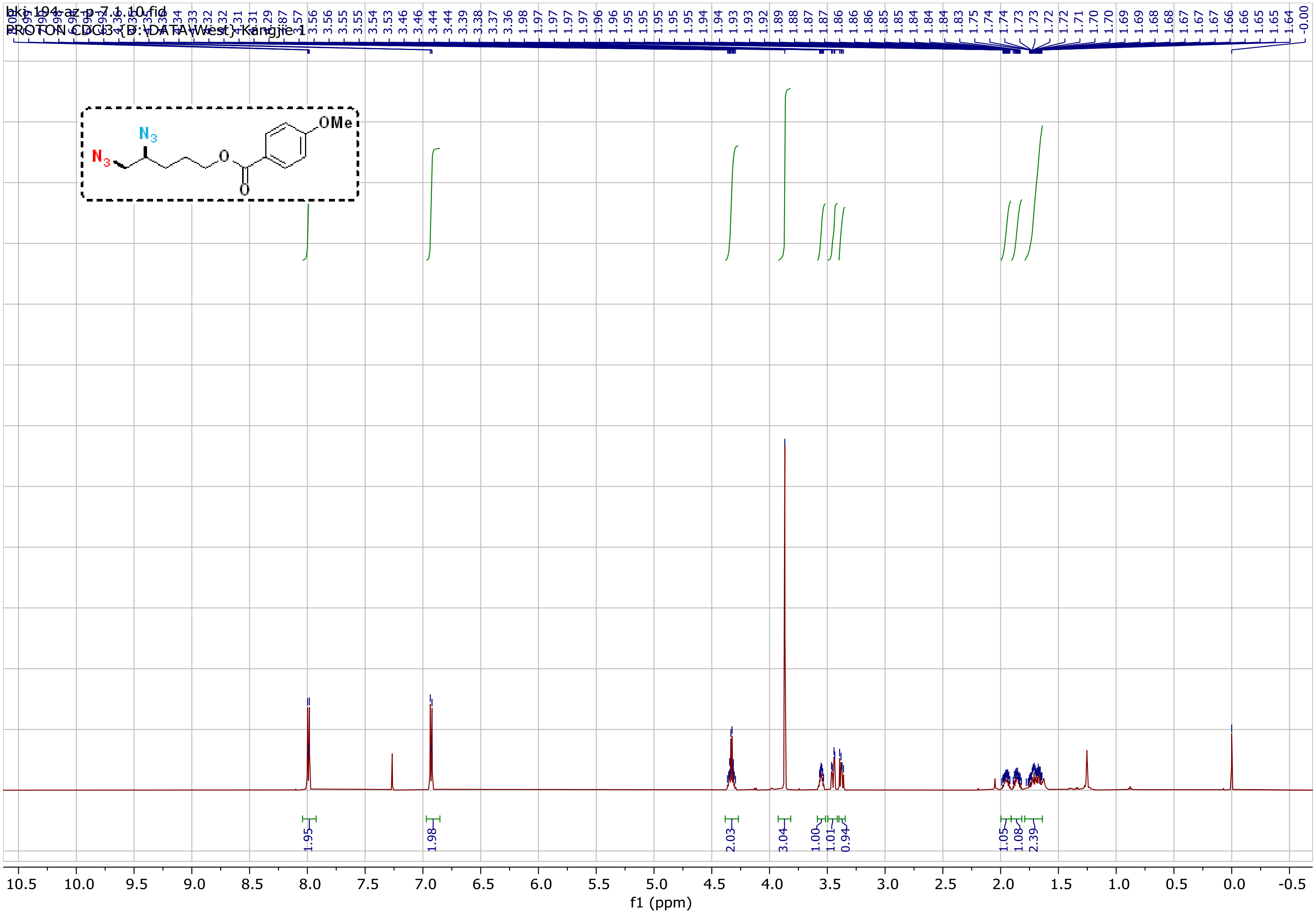

Supplementary Figure 9. ^{1}H NMR Spectrum of **5** (600 MHz, $CDCl_3$)

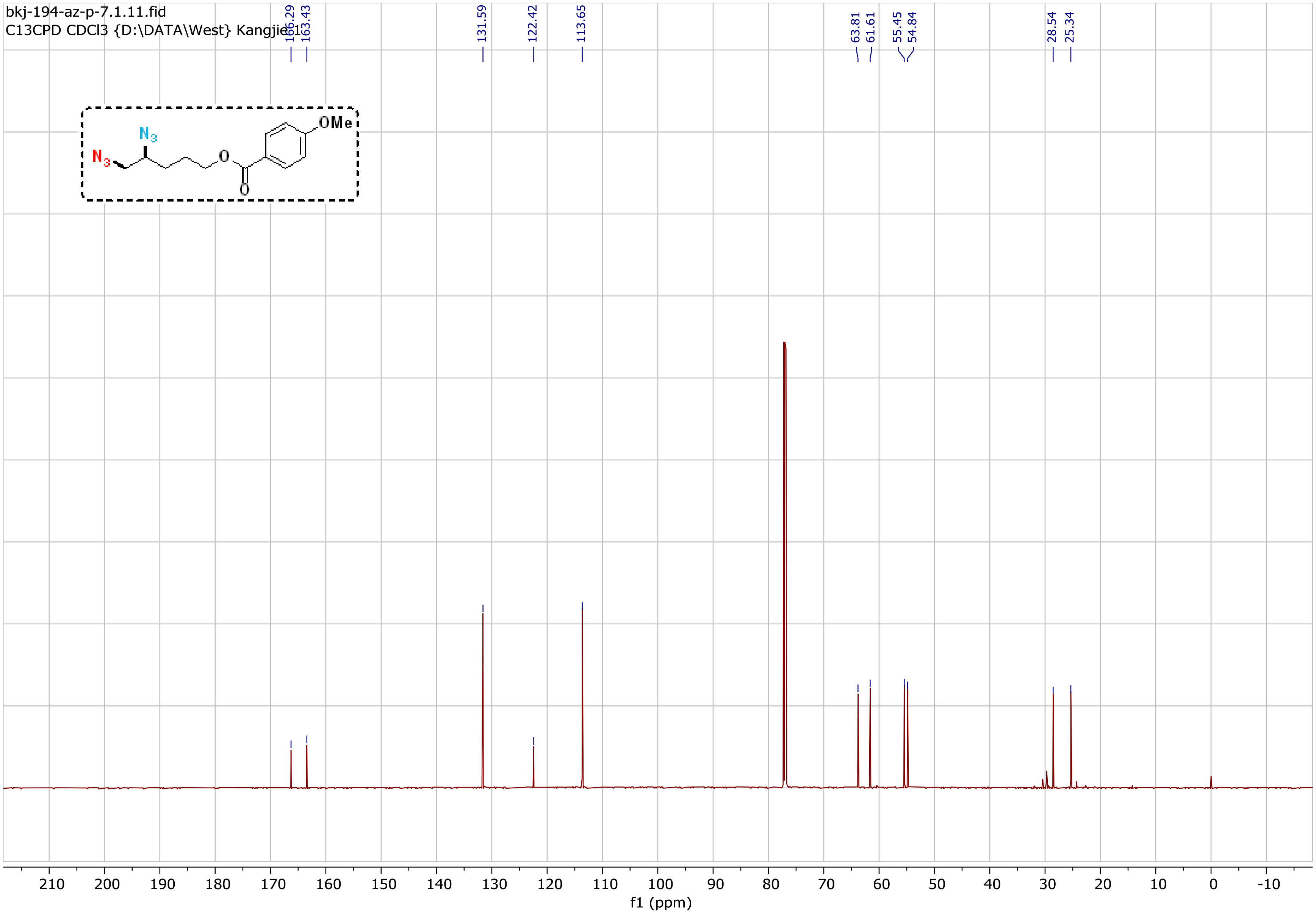

Supplementary Figure 10. ^{13}C NMR Spectrum of **5** (151 MHz, $CDCl_3$)

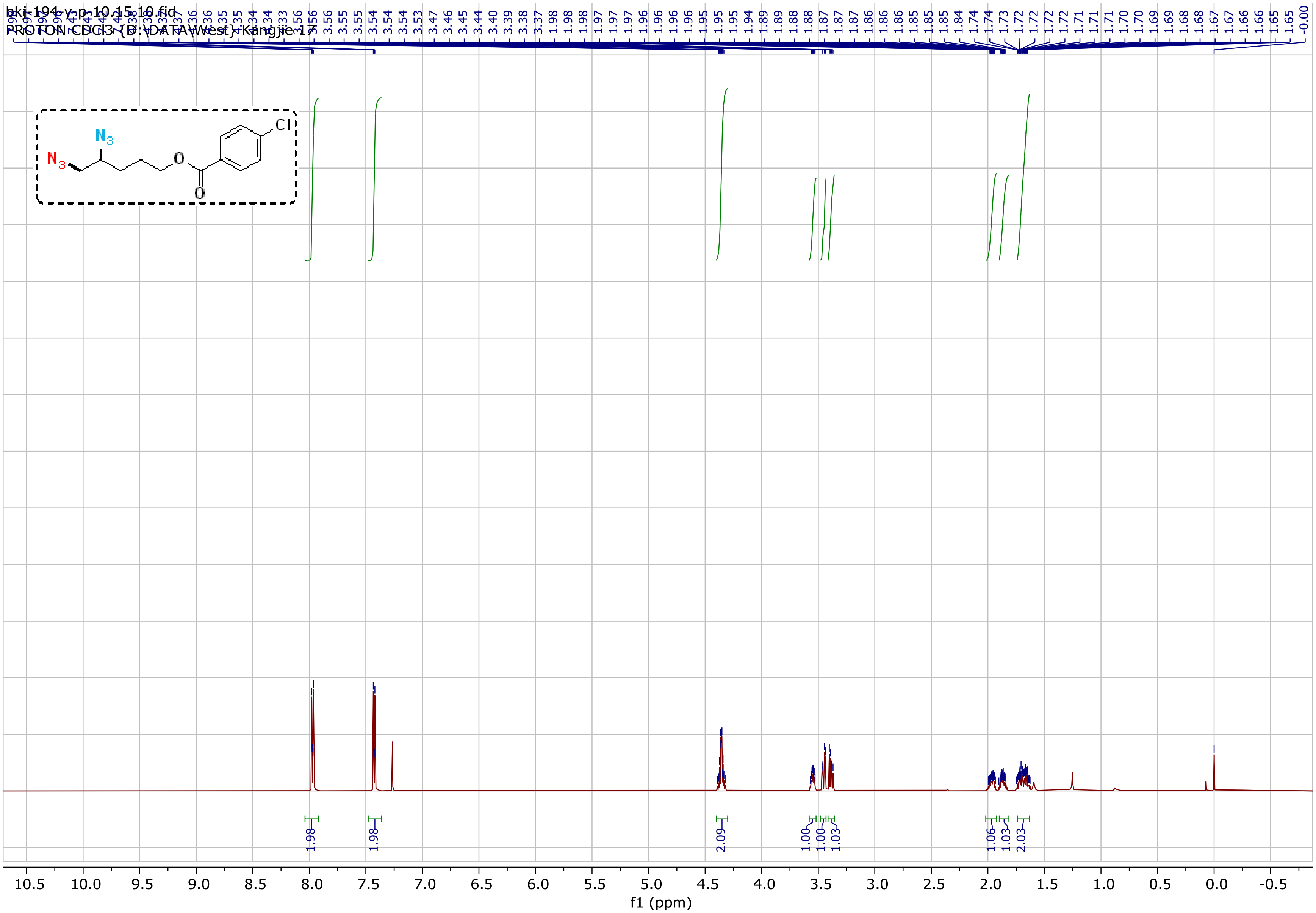

Supplementary Figure 11. ^{1}H NMR Spectrum of **6** (600 MHz, $CDCl_3$)

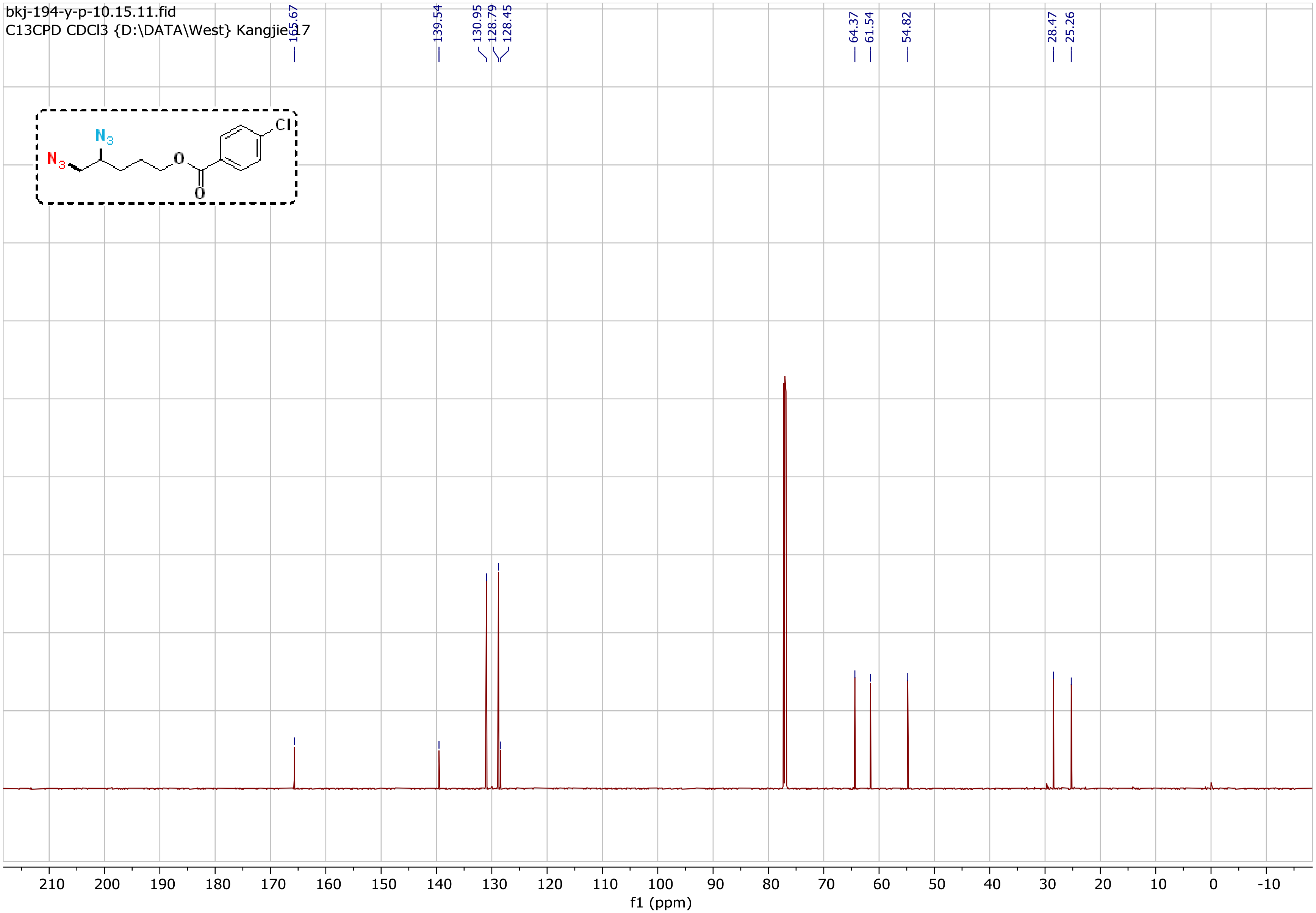

Supplementary Figure 12. ^{13}C NMR Spectrum of **6** (151 MHz, $CDCl_3$)

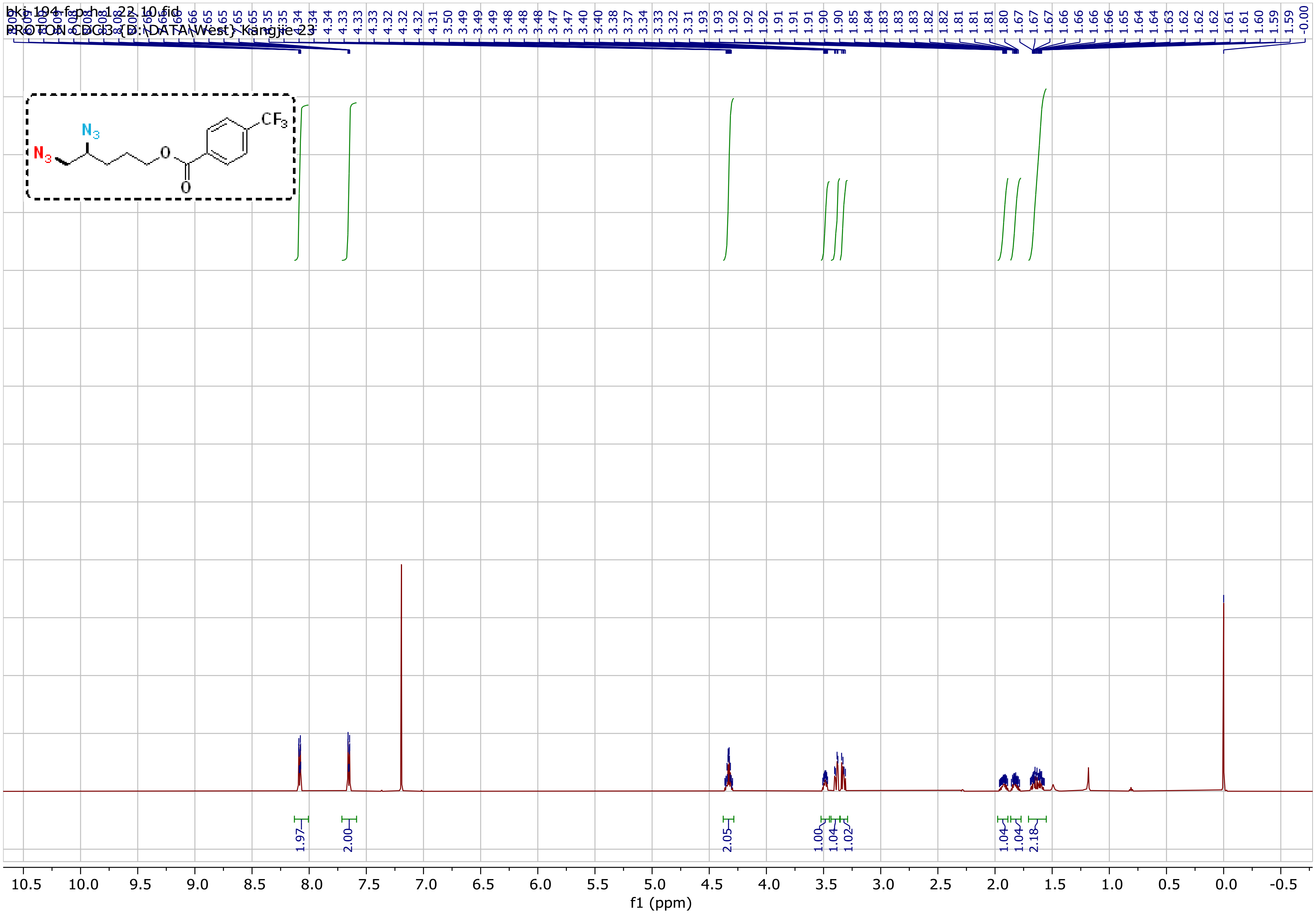

Supplementary Figure 13. ^{1}H NMR Spectrum of **7** (600 MHz, $CDCl_3$)

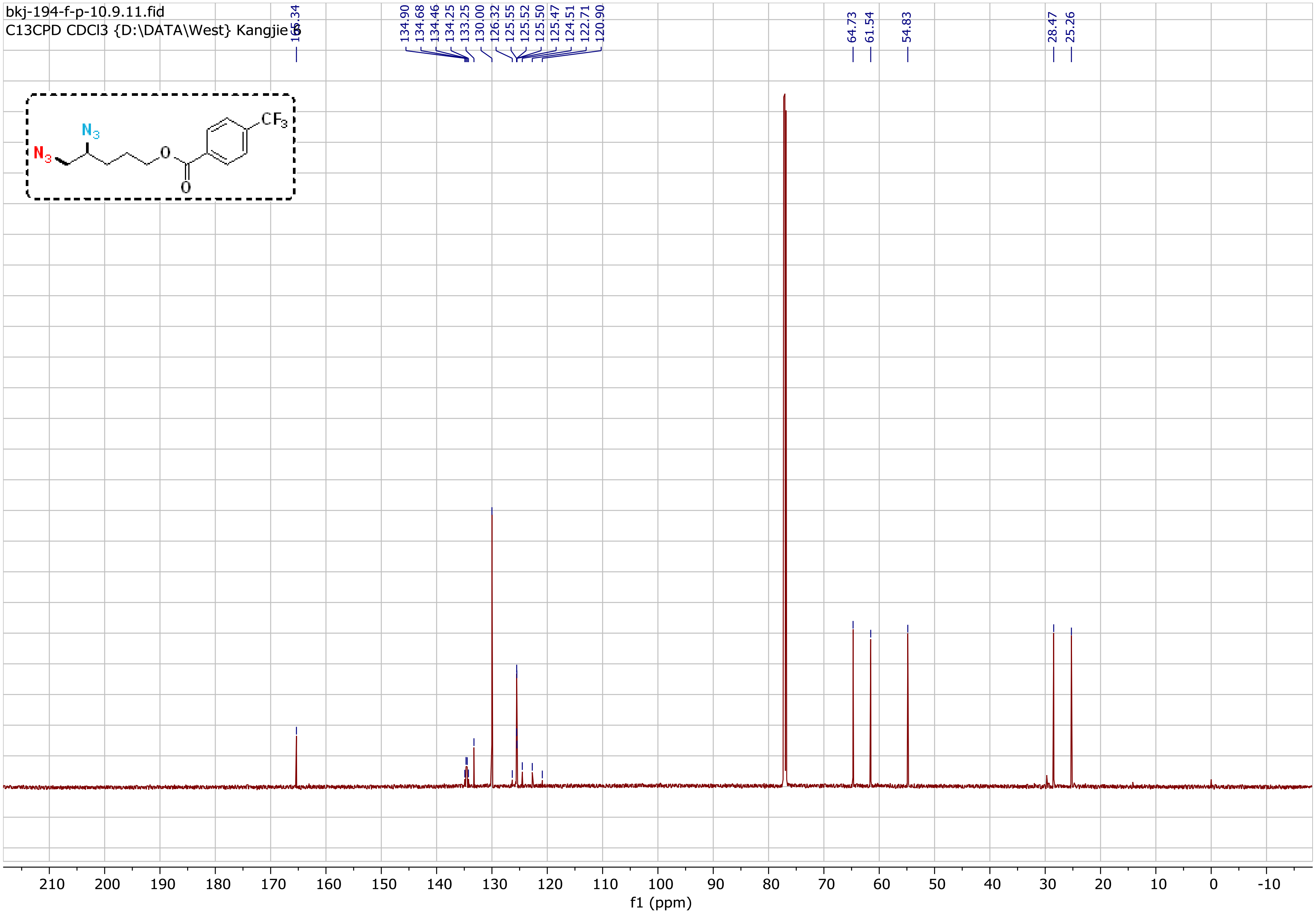

Supplementary Figure 14. ^{13}C NMR Spectrum of **7** (151 MHz, $CDCl_3$)

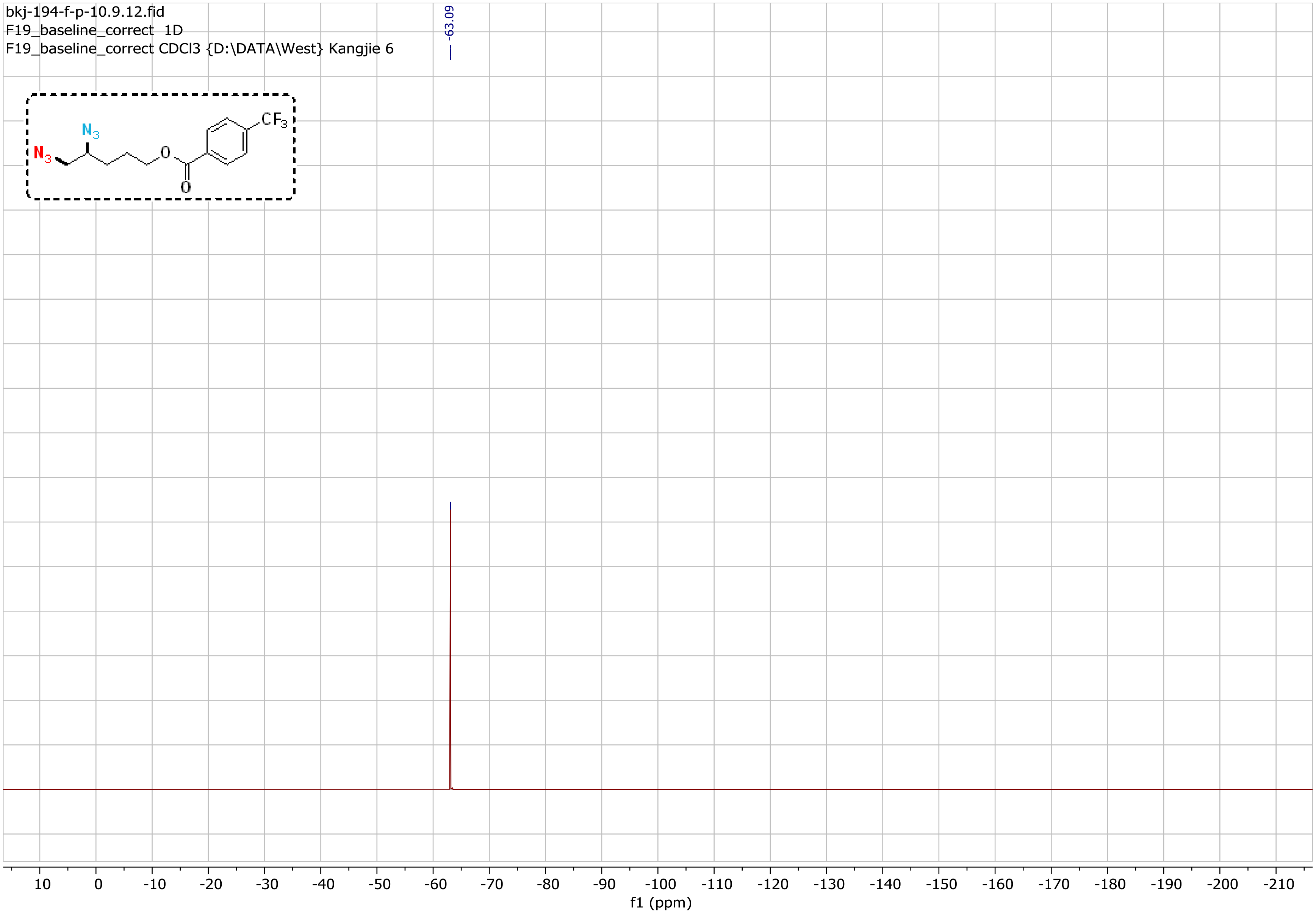

Supplementary Figure 15. ^{19}F NMR Spectrum of **7** (565 MHz, $CDCl_3$)

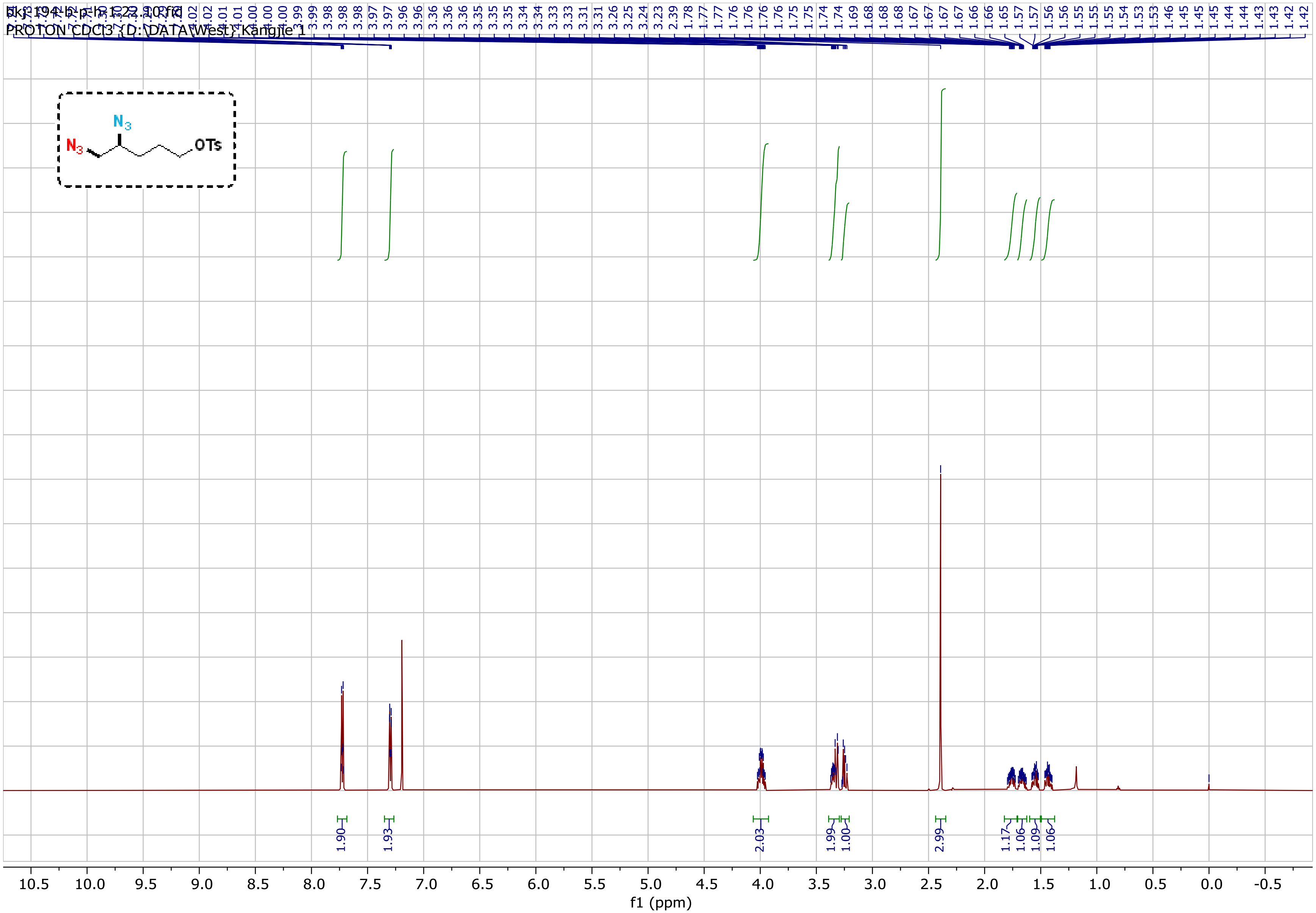

Supplementary Figure 16. ^{1}H NMR Spectrum of **8** (600 MHz, $CDCl_3$)

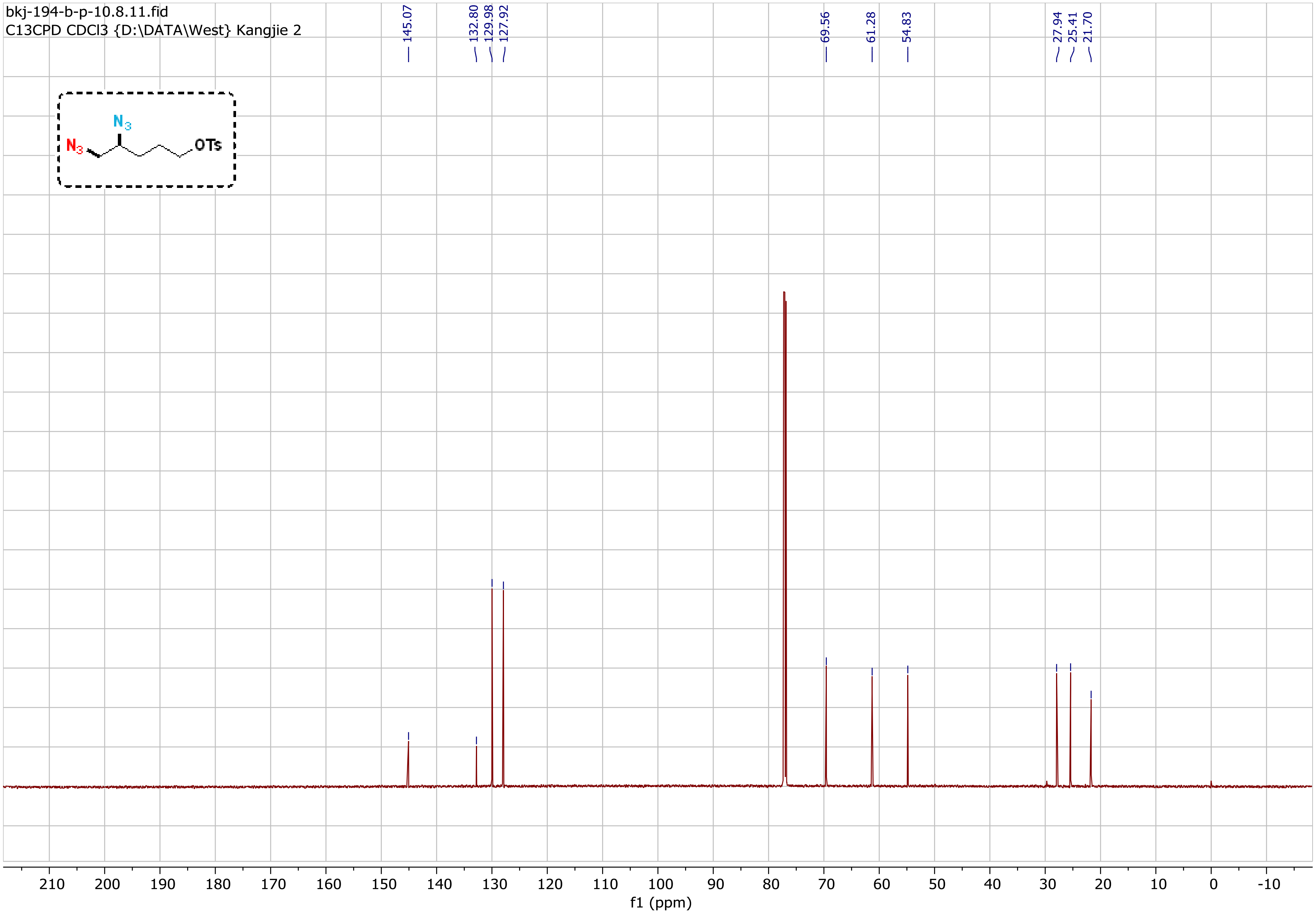

Supplementary Figure 17. ^{13}C NMR Spectrum of **8** (151 MHz, $CDCl_3$)

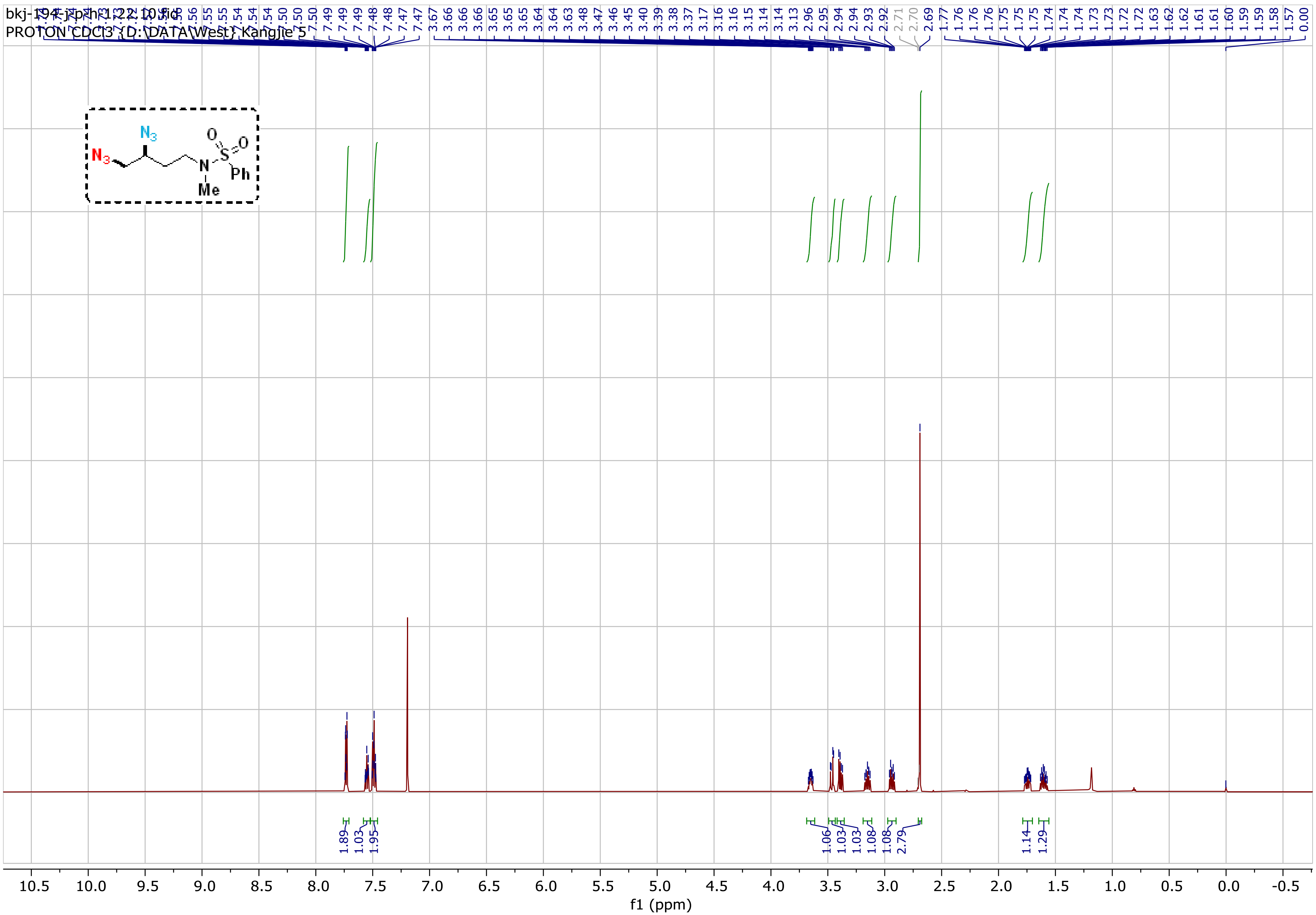

Supplementary Figure 18.^{1}H NMR Spectrum of **9** (600 MHz, $CDCl_3$)

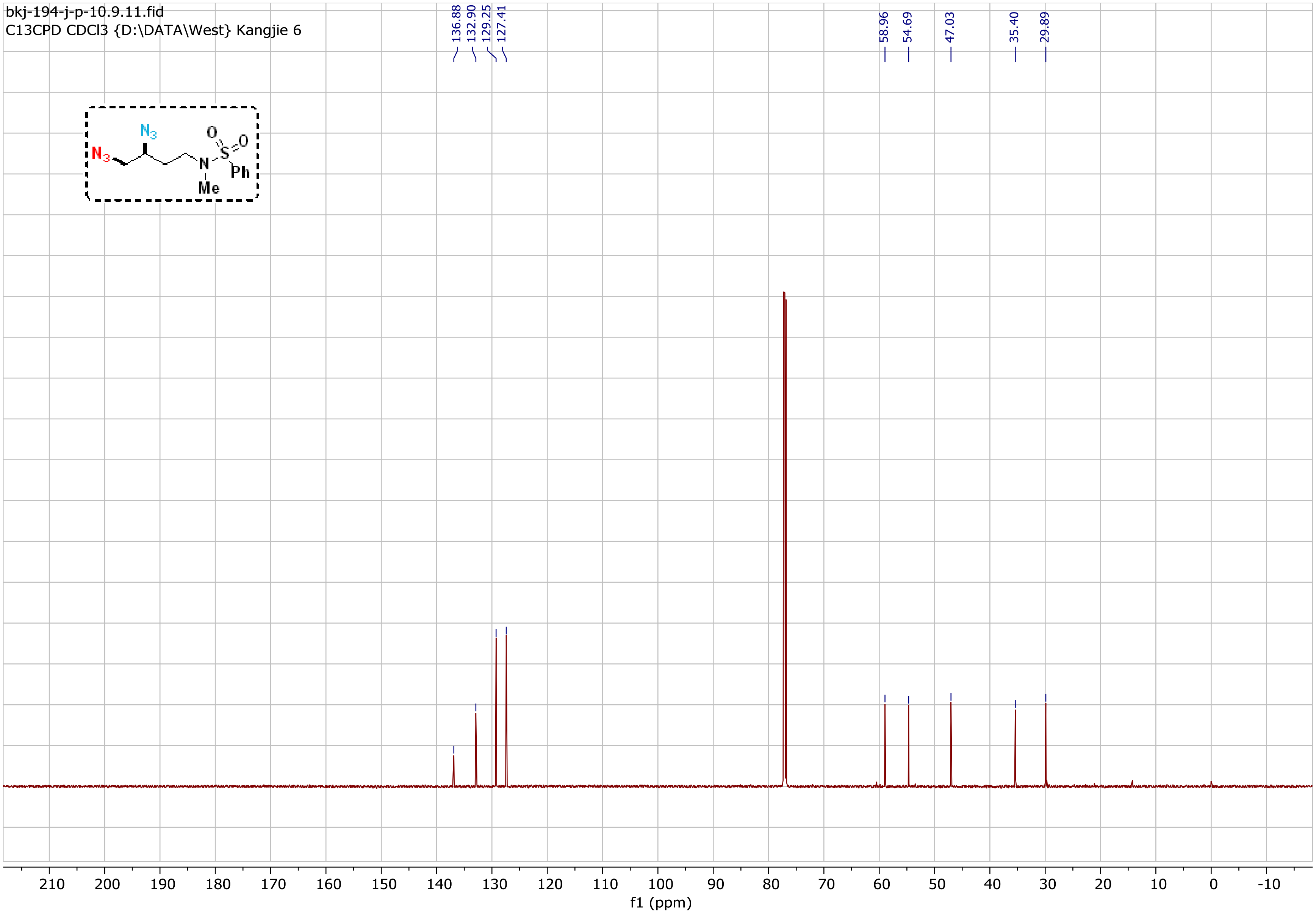

Supplementary Figure 19. ^{13}C NMR Spectrum of **9** (151 MHz, $CDCl_3$)

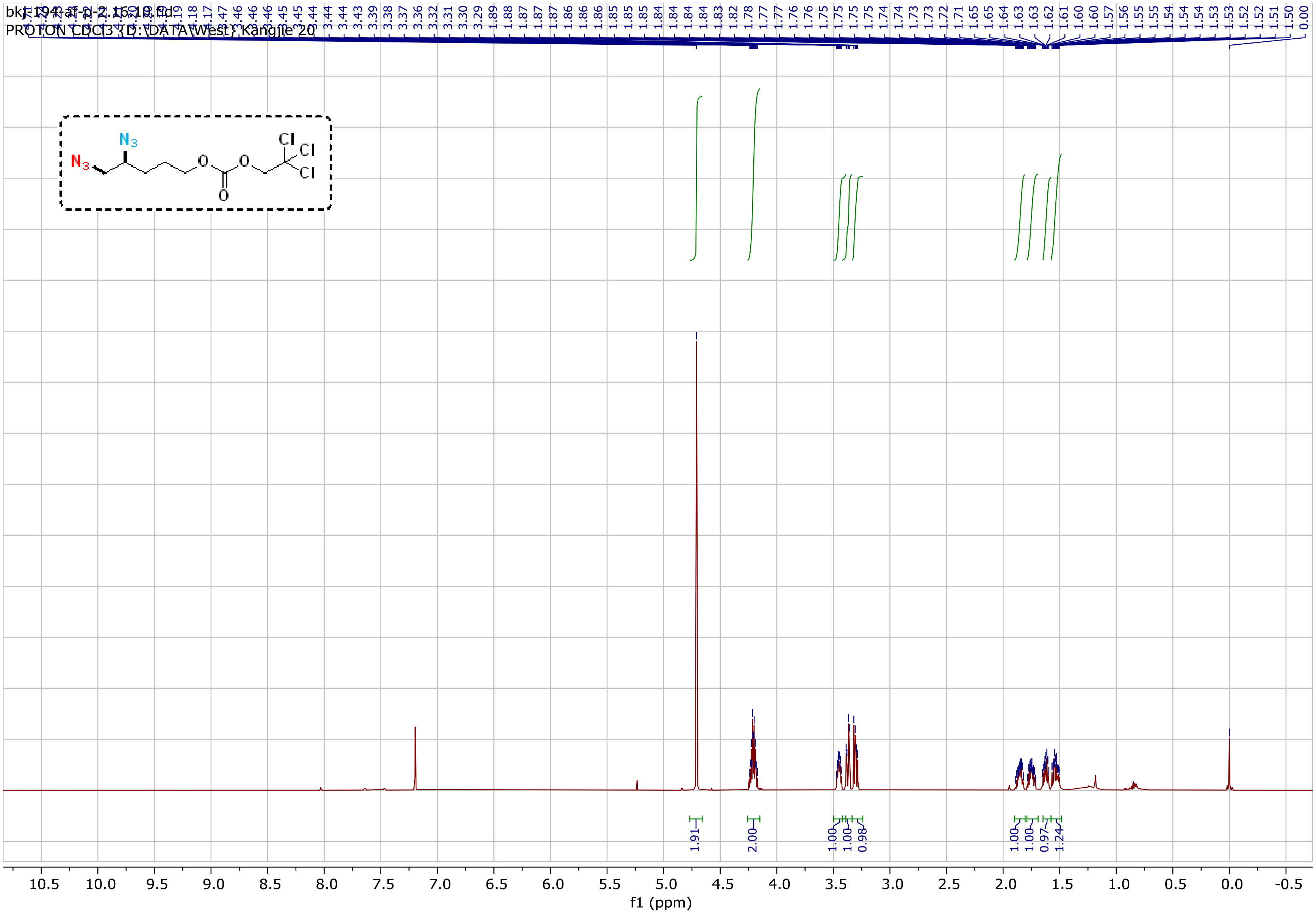

Supplementary Figure 20. ^{1}H NMR Spectrum of **10** (600 MHz, $CDCl_3$)

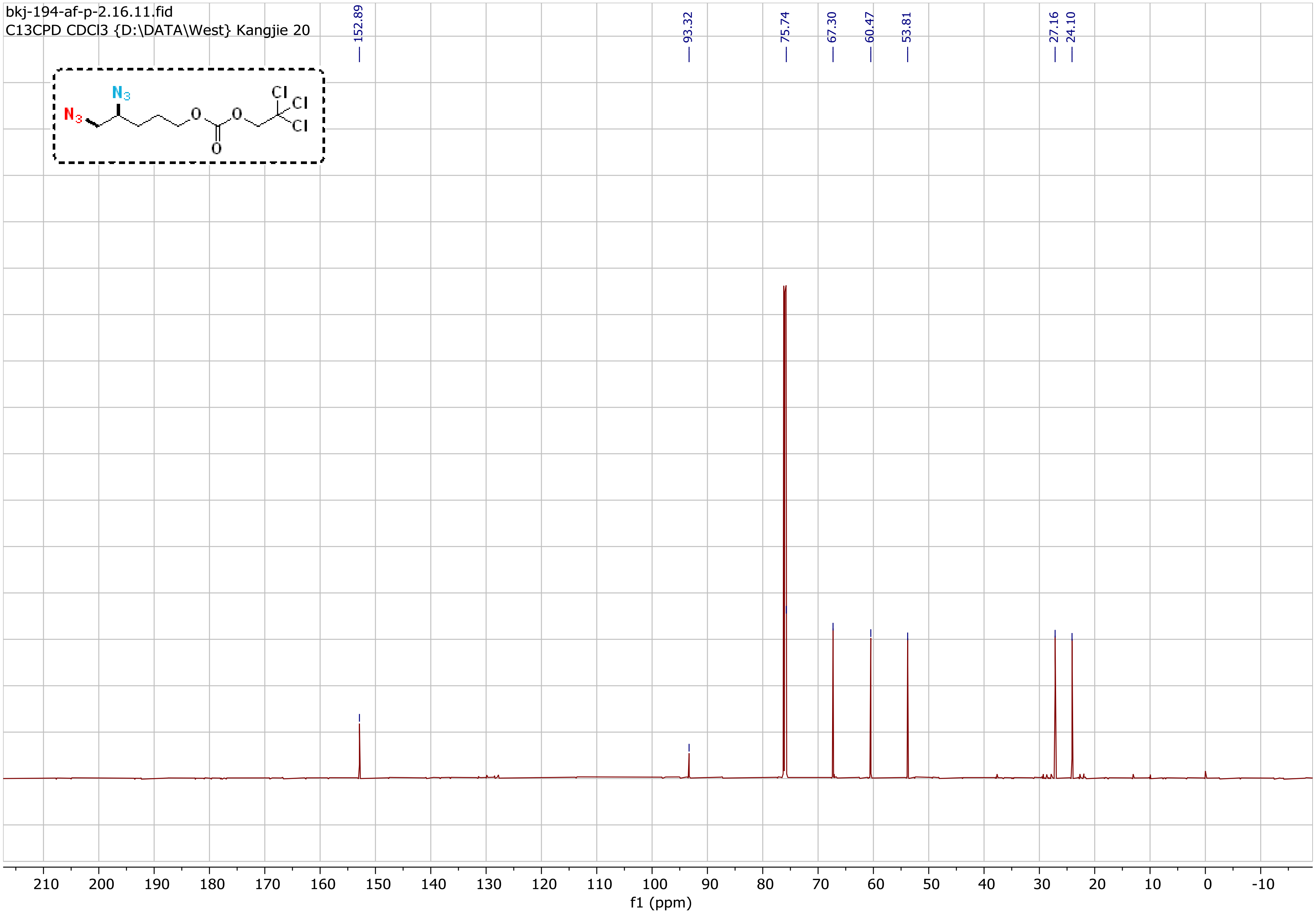

Supplementary Figure 21. ^{13}C NMR Spectrum of **10** (151 MHz, $CDCl_3$)

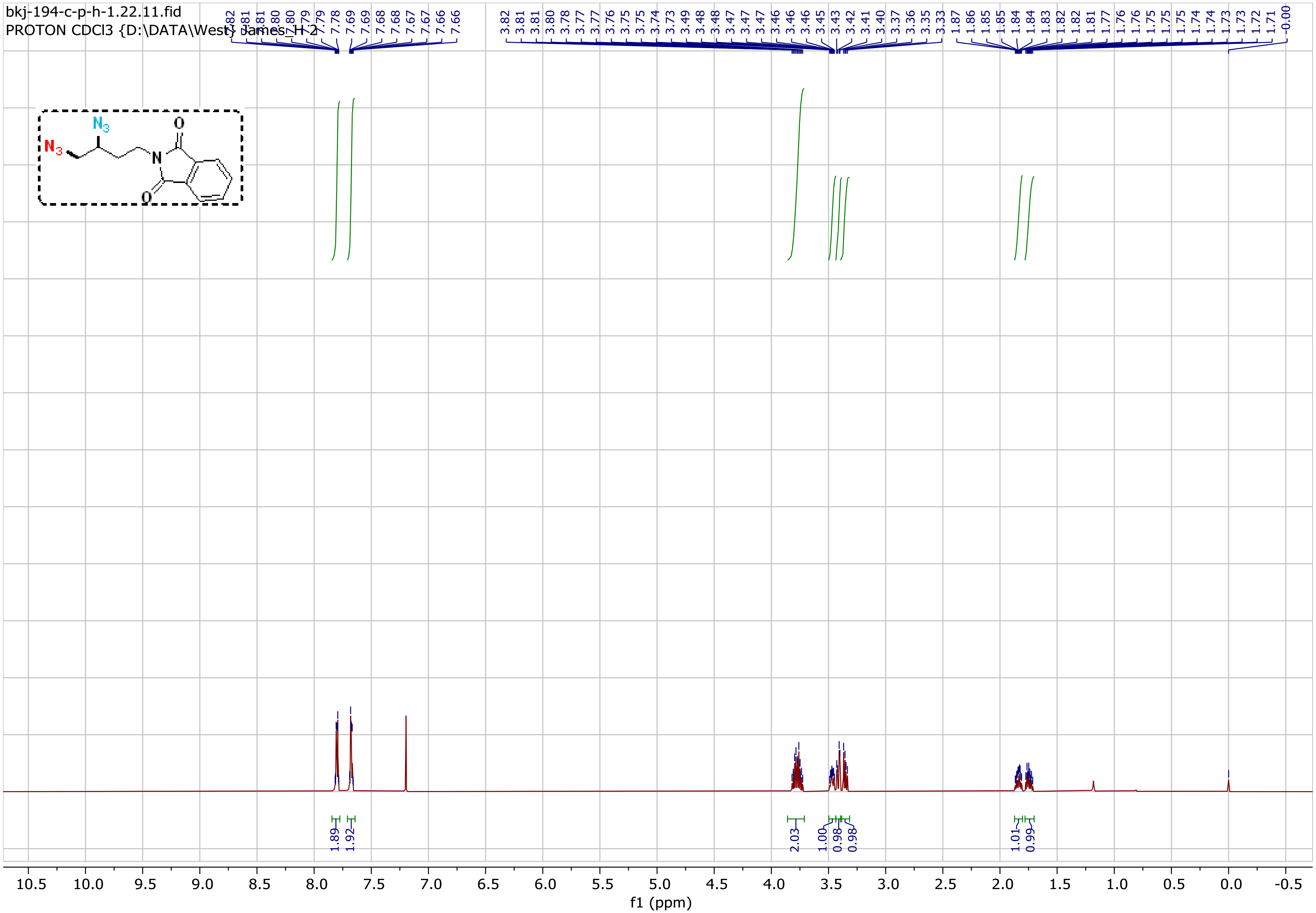

Supplementary Figure 22. ^{1}H NMR Spectrum of **11** (600 MHz, $CDCl_3$)

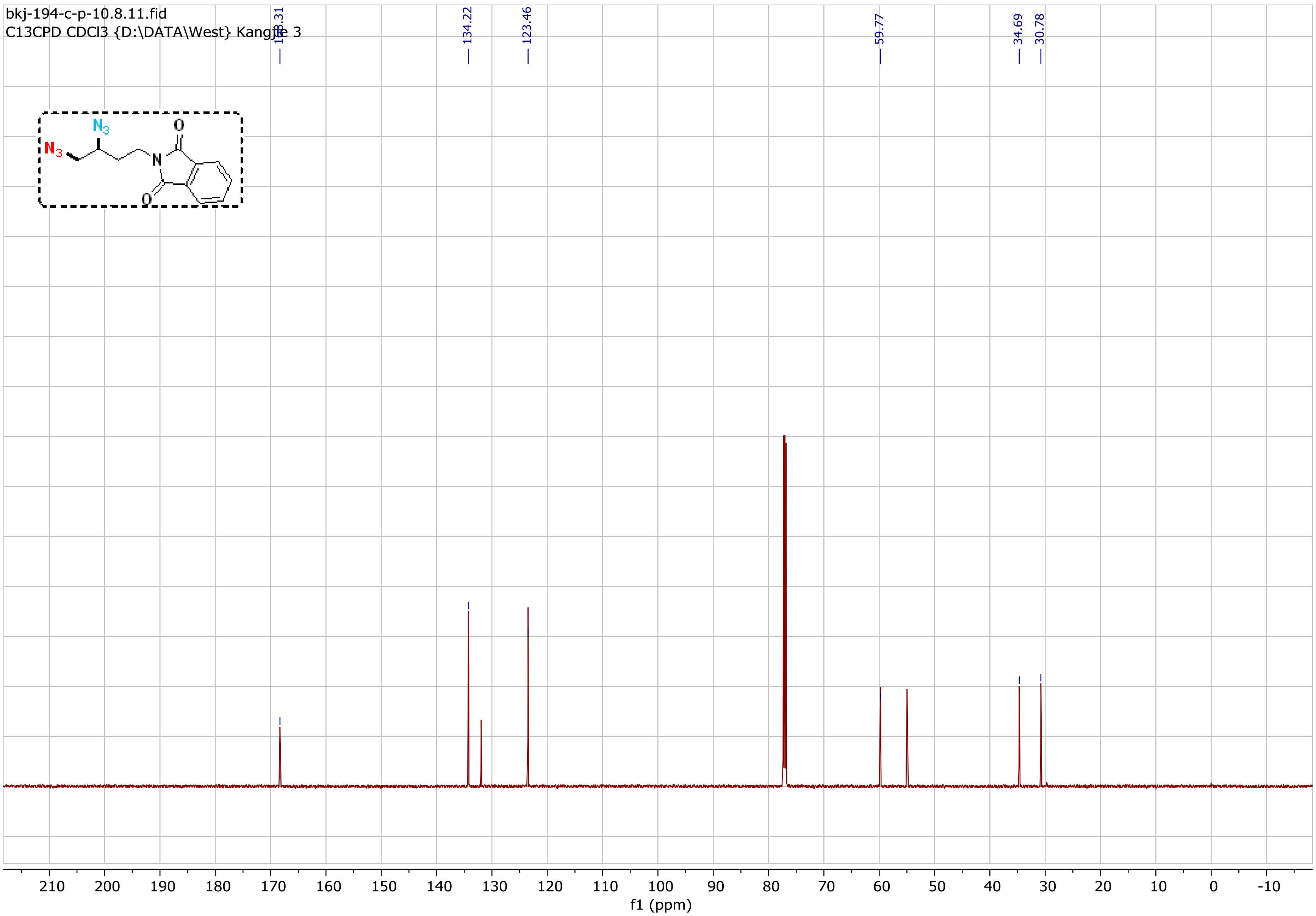

Supplementary Figure 23. ^{13}C NMR Spectrum of **11** (151 MHz, $CDCl_3$)

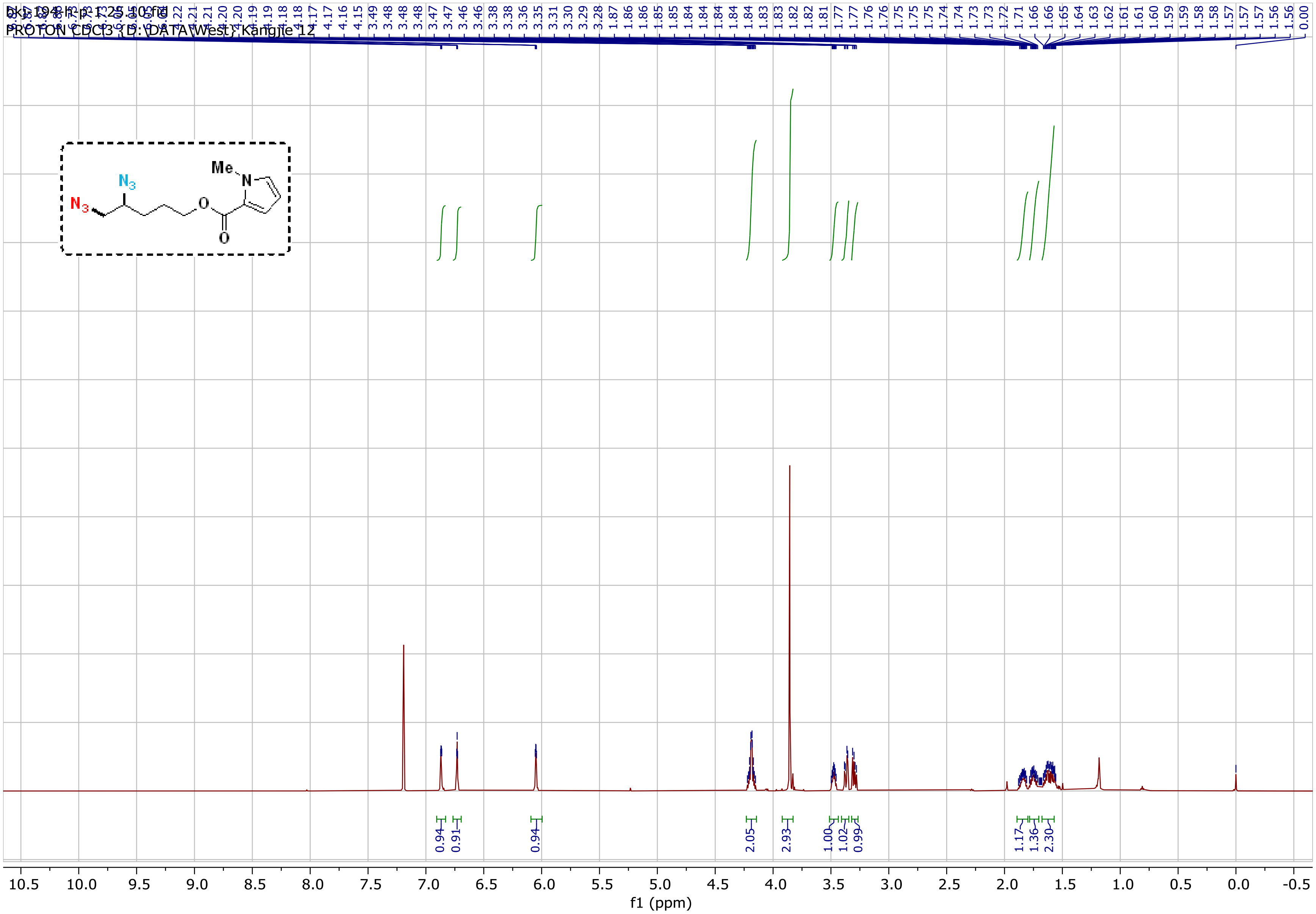

Supplementary Figure 24. ^{1}H NMR Spectrum of **12** (600 MHz, $CDCl_3$)

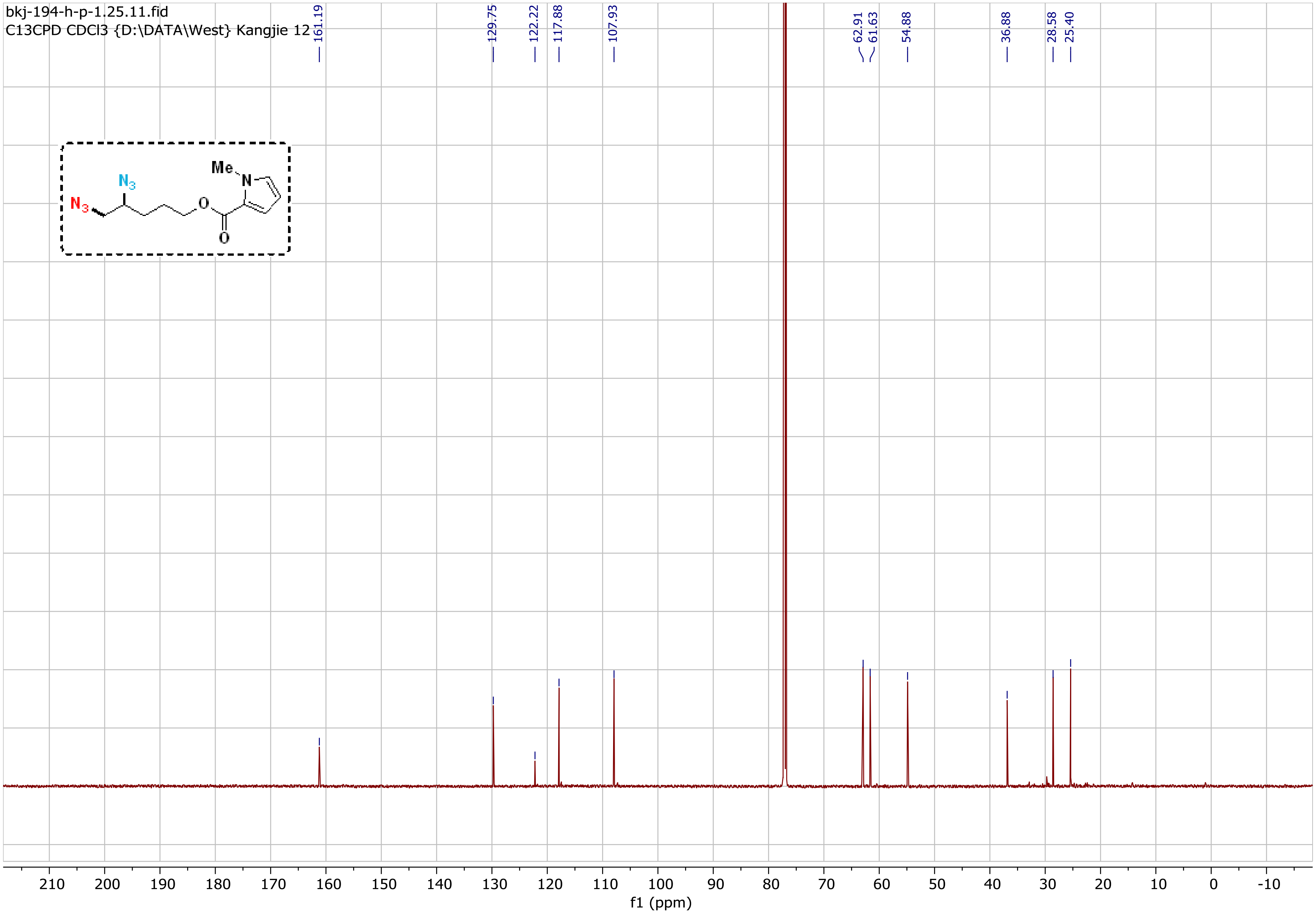

Supplementary Figure 25. ^{13}C NMR Spectrum of **12** (151 MHz, $CDCl_3$)

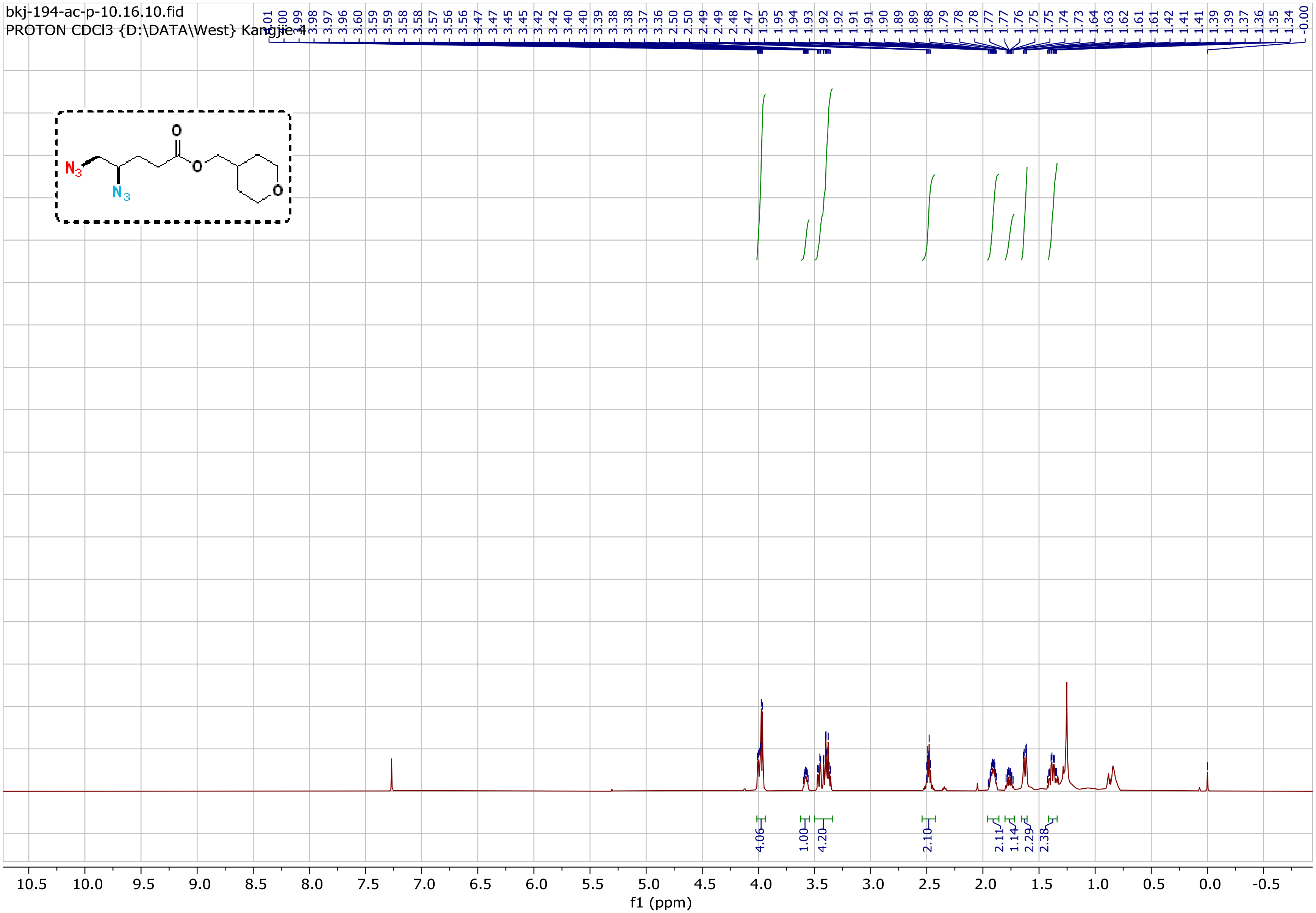

Supplementary Figure 26. ^{1}H NMR Spectrum of **13** (600 MHz, $CDCl_3$)

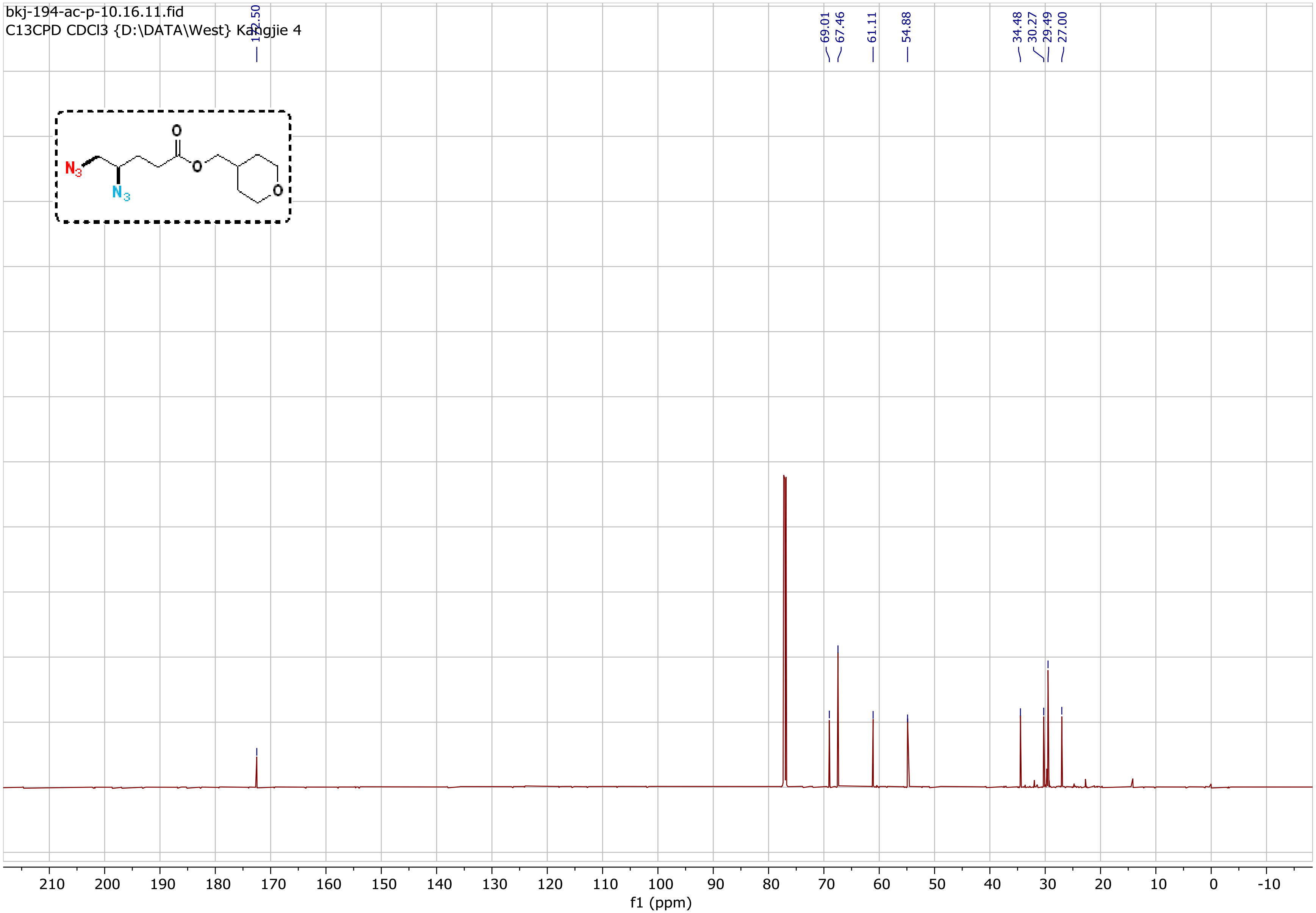

Supplementary Figure 27. ^{13}C NMR Spectrum of **13** (151 MHz, $CDCl_3$)

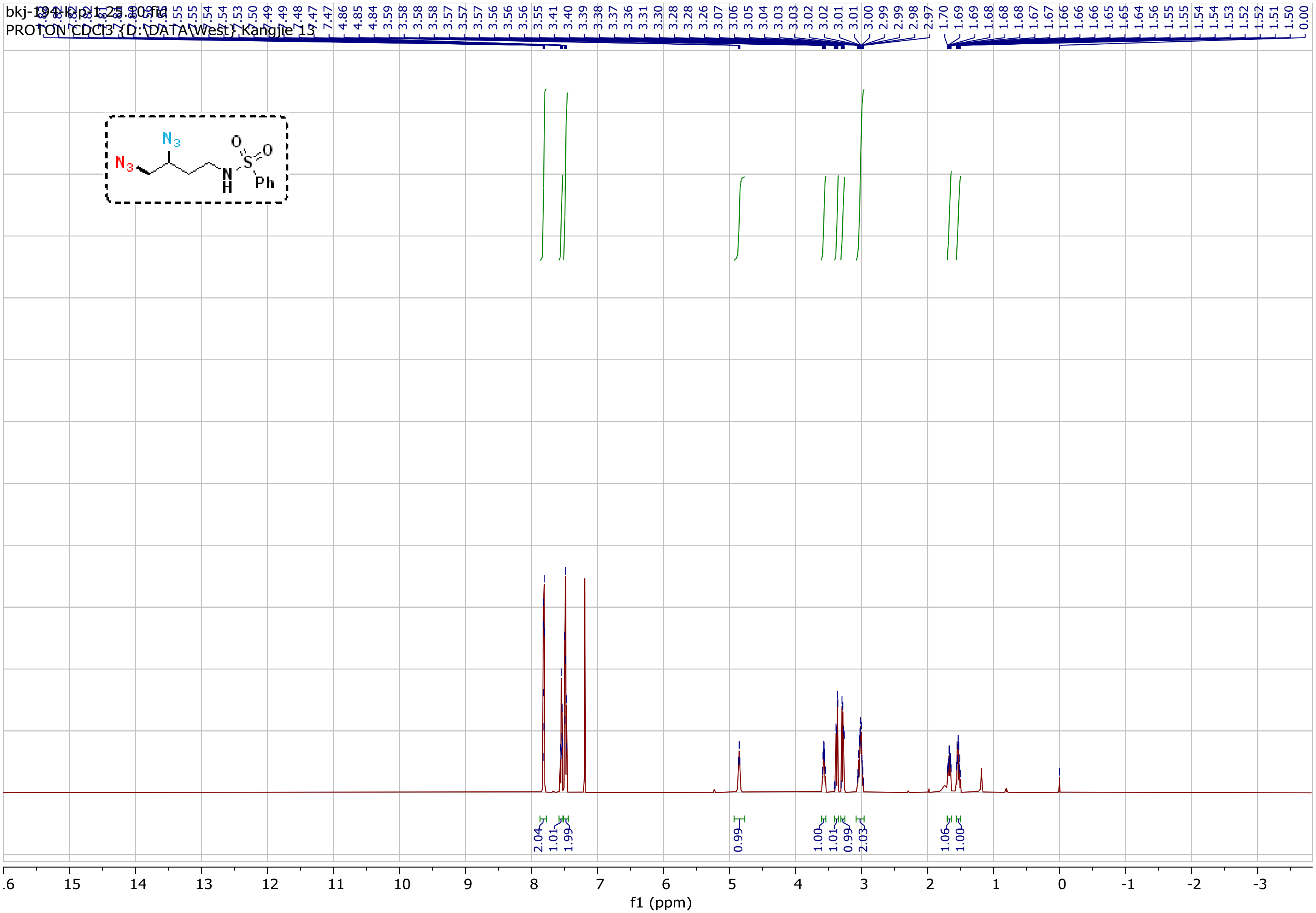

Supplementary Figure 28. ^{1}H NMR Spectrum of **14** (600 MHz, $CDCl_3$)

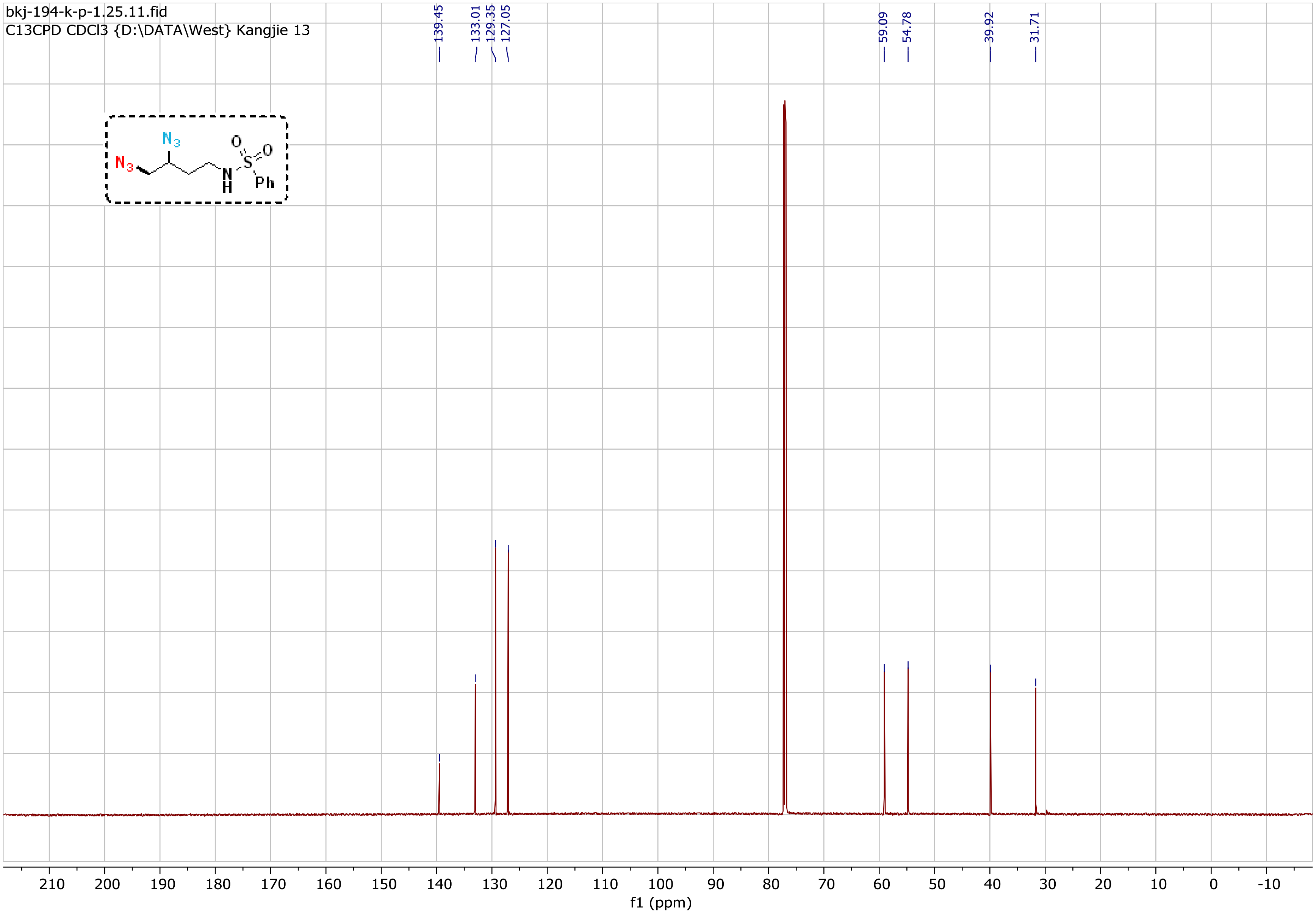

Supplementary Figure 29. ^{13}C NMR Spectrum of **14** (151 MHz, $CDCl_3$)

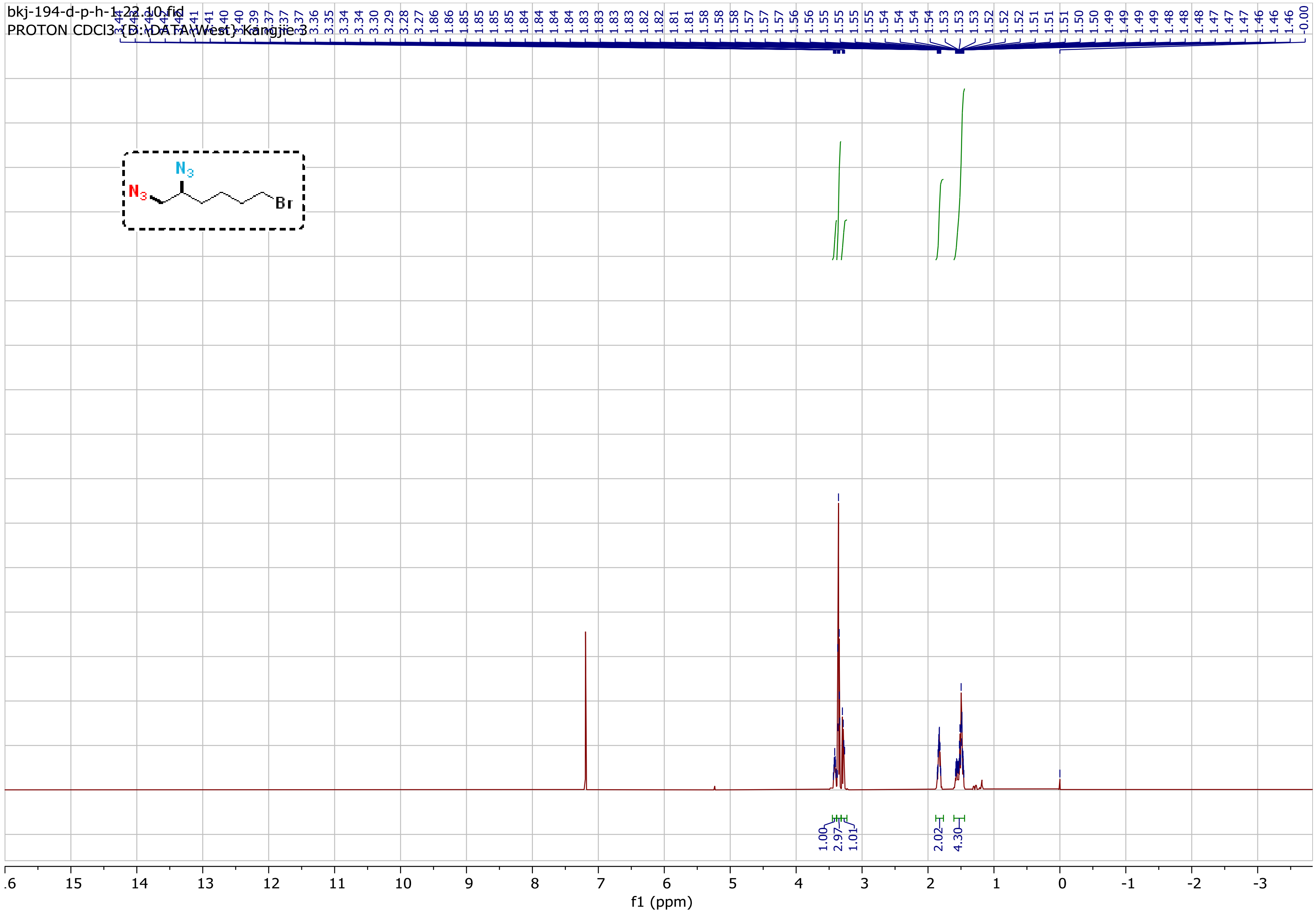

Supplementary Figure 30. ^{1}H NMR Spectrum of **15** (600 MHz, $CDCl_3$)

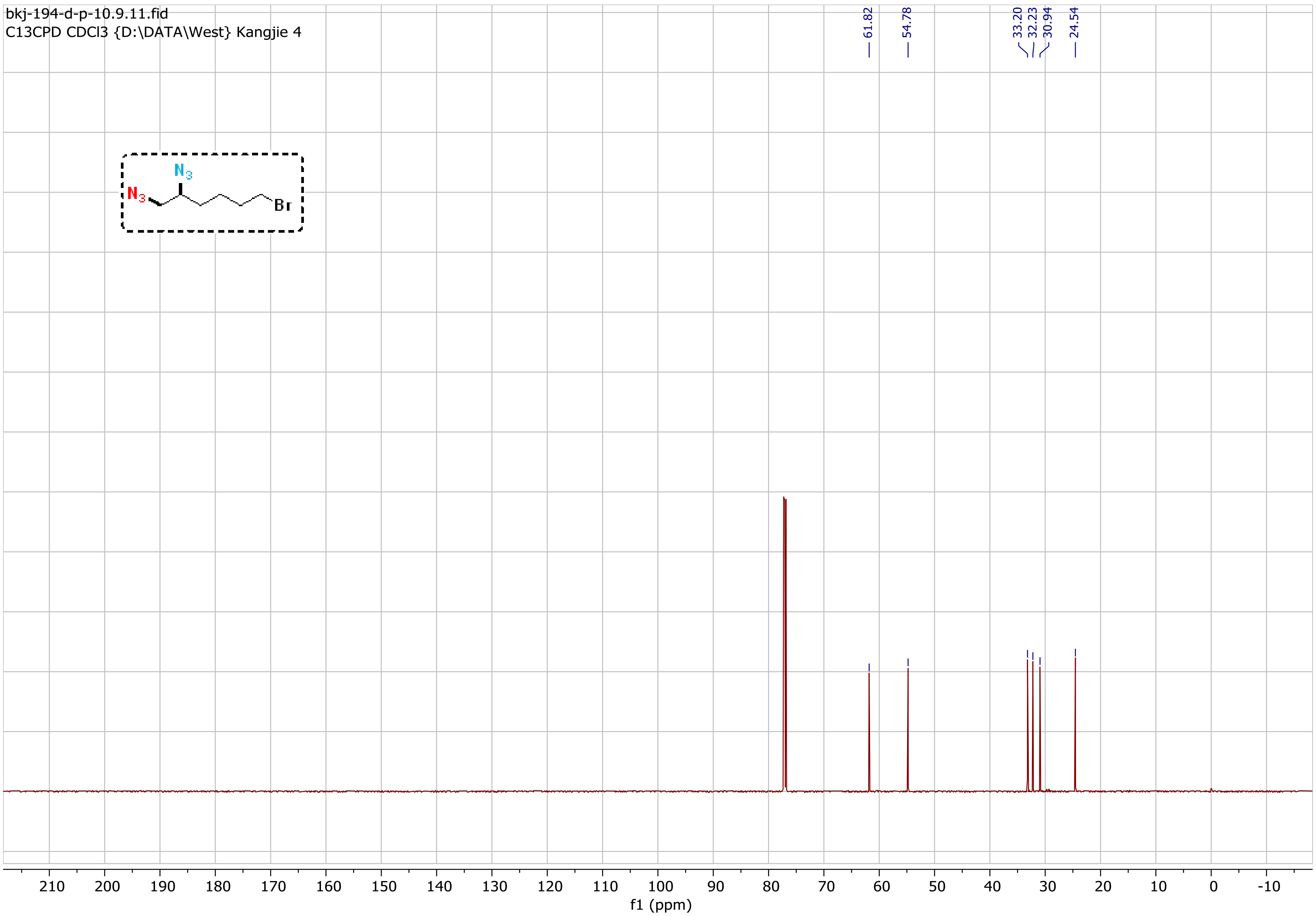

Supplementary Figure 31. ^{13}C NMR Spectrum of **15** (151 MHz, $CDCl_3$)

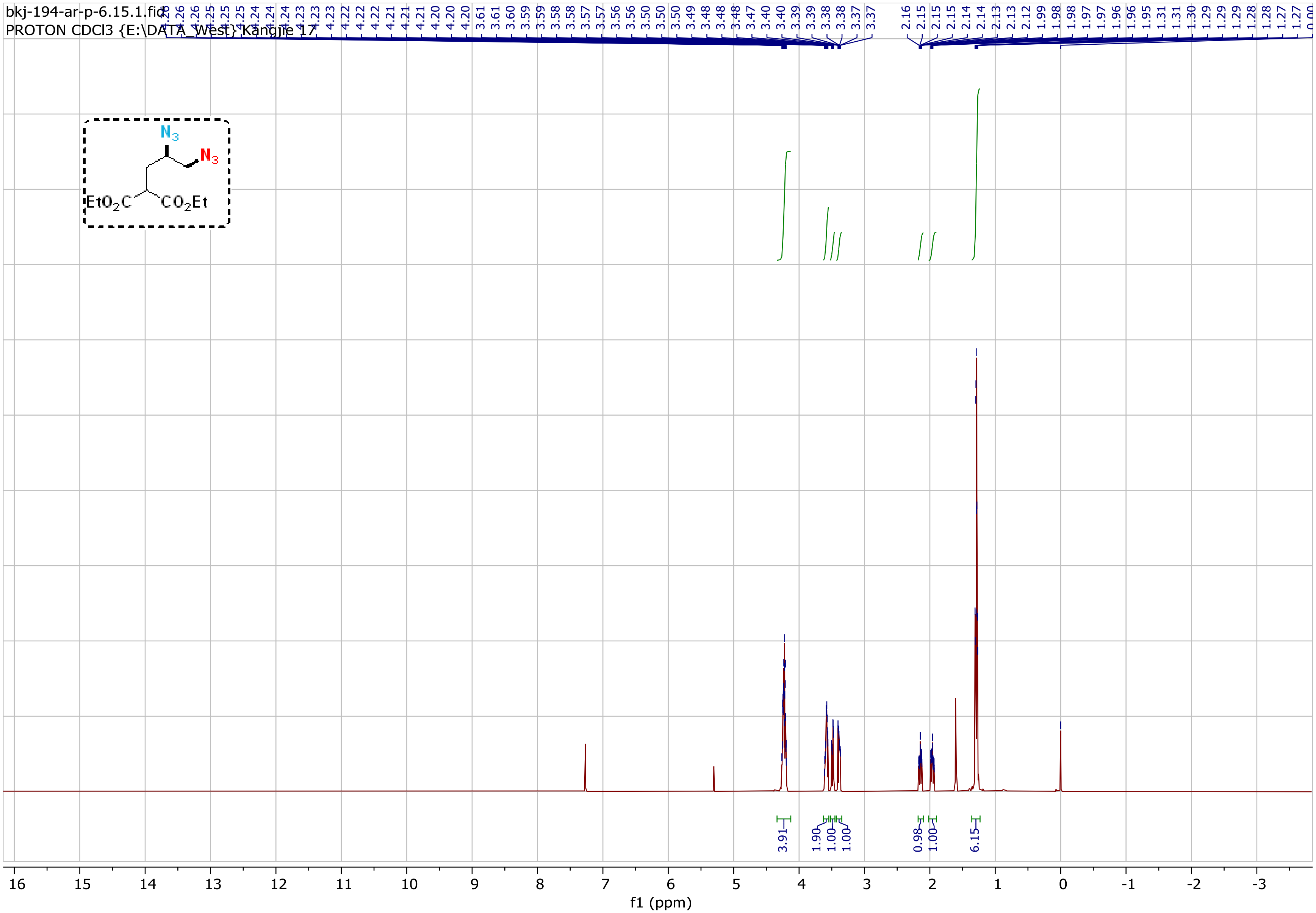

Supplementary Figure 32. ^{1}H NMR Spectrum of **16** (600 MHz, $CDCl_3$)

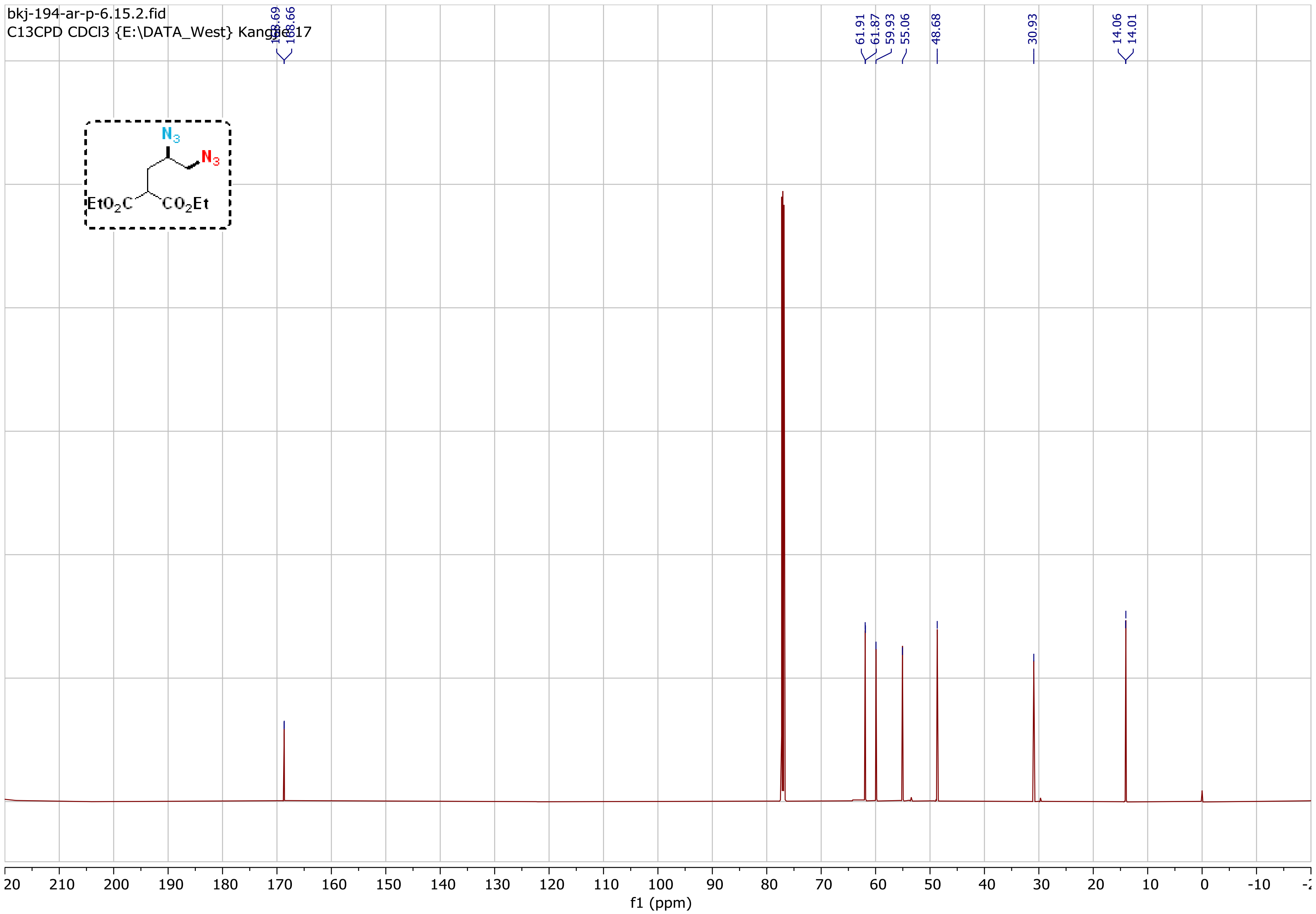

Supplementary Figure 33. ^{13}C NMR Spectrum of **16** (151 MHz, $CDCl_3$)

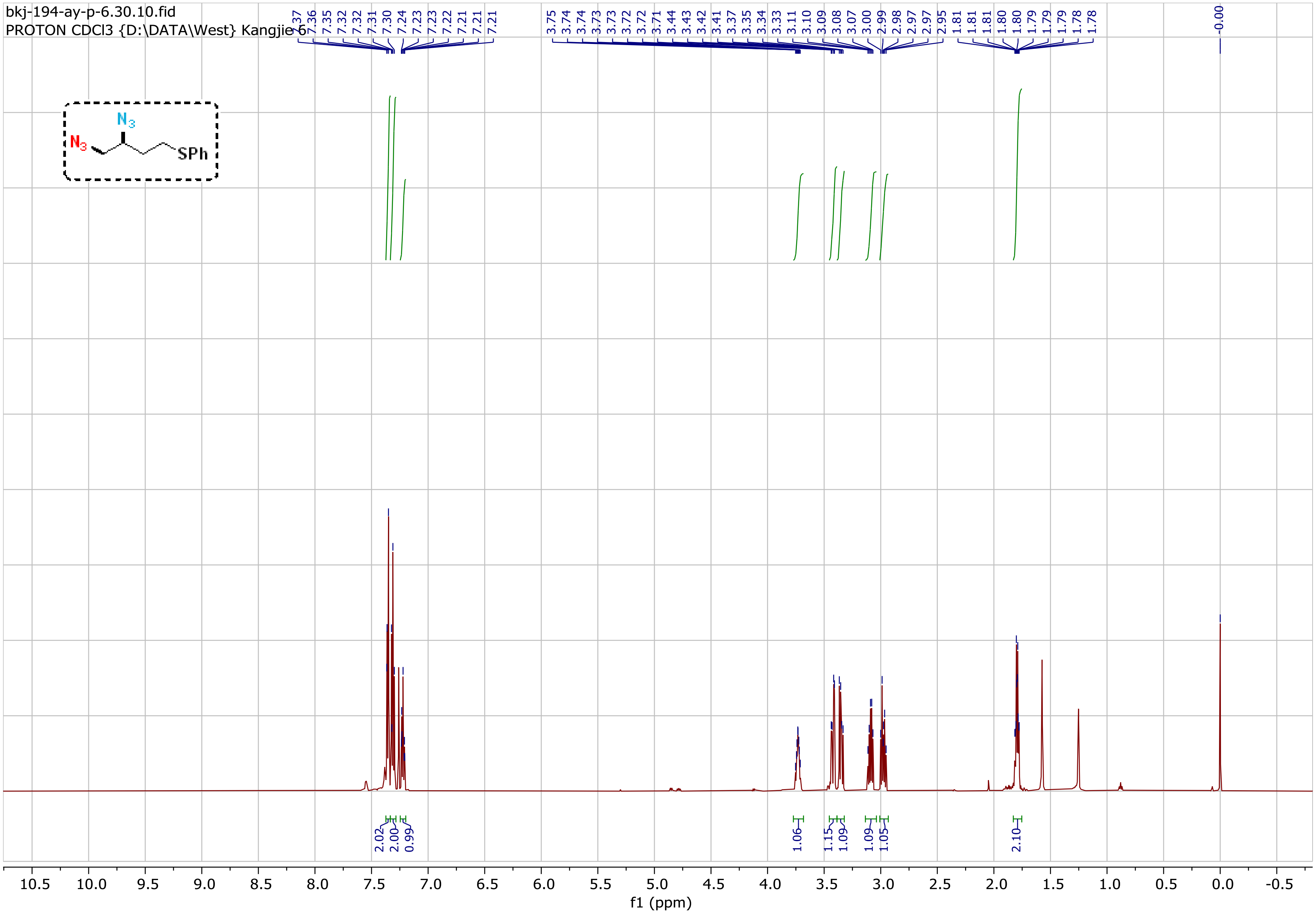

Supplementary Figure 34. ^{1}H NMR Spectrum of **17** (600 MHz, $CDCl_3$)

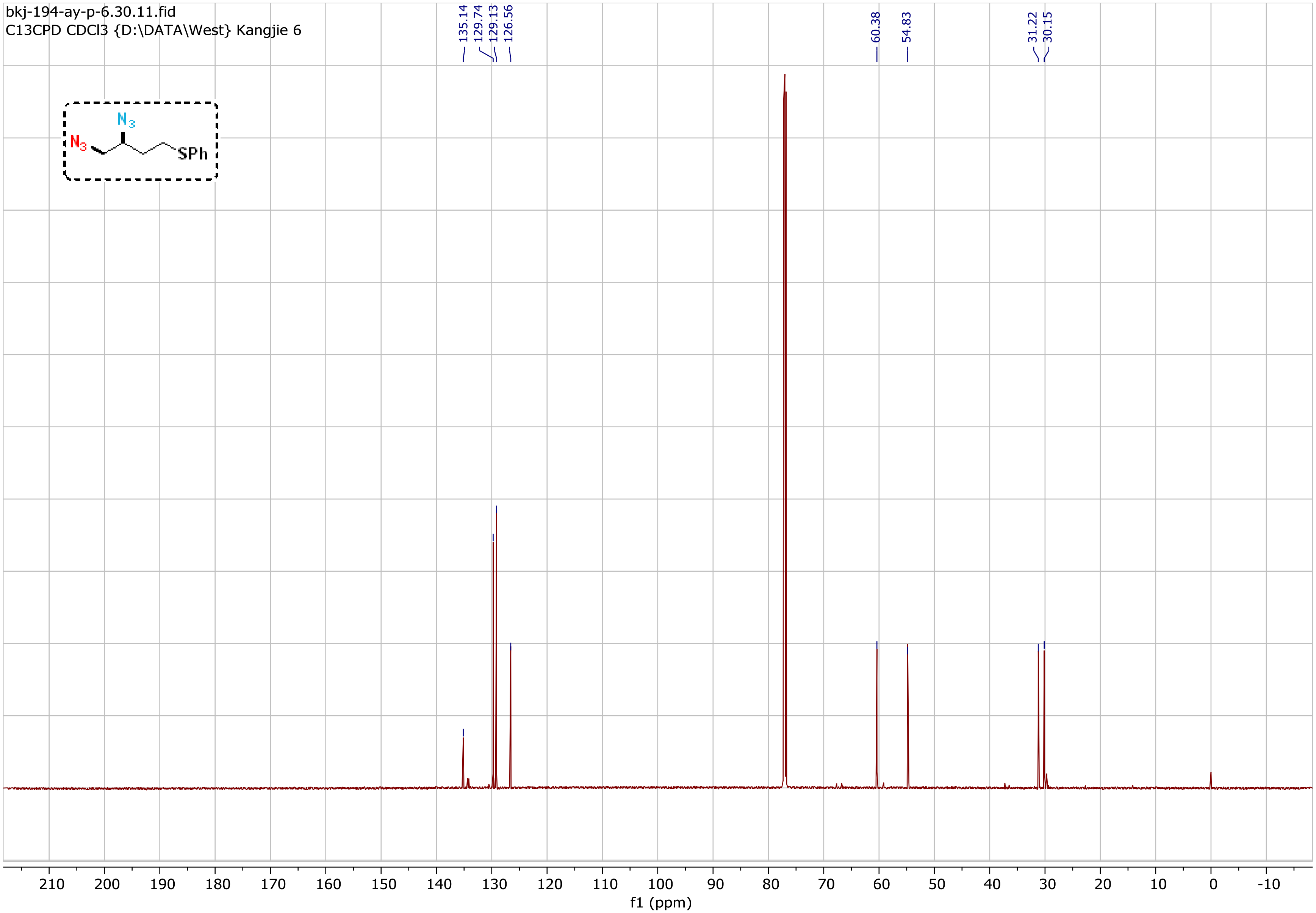

Supplementary Figure 35. ^{13}C NMR Spectrum of **17** (151 MHz, $CDCl_3$)

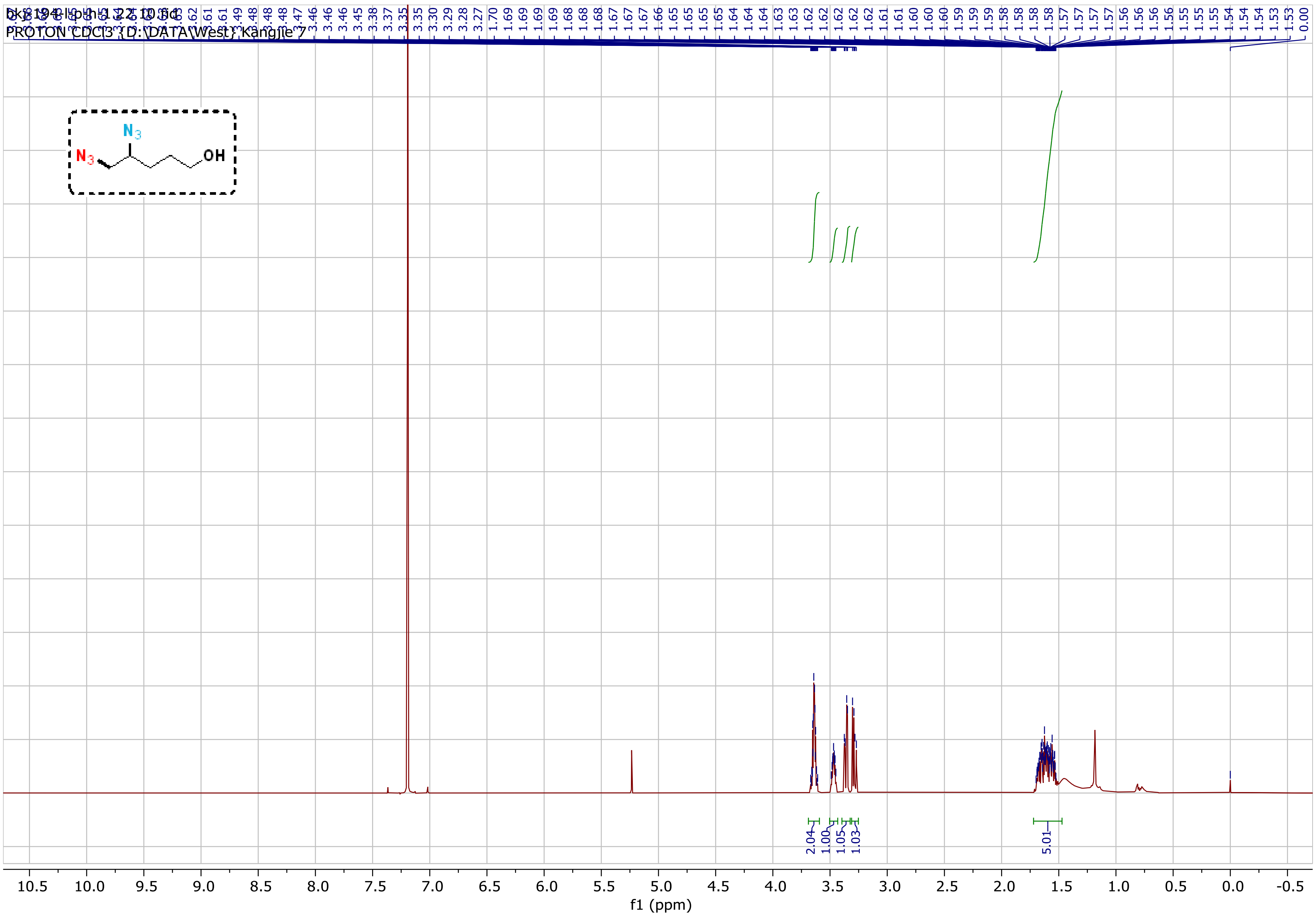

Supplementary Figure 36. ^{1}H NMR Spectrum of **18** (600 MHz, $CDCl_3$)

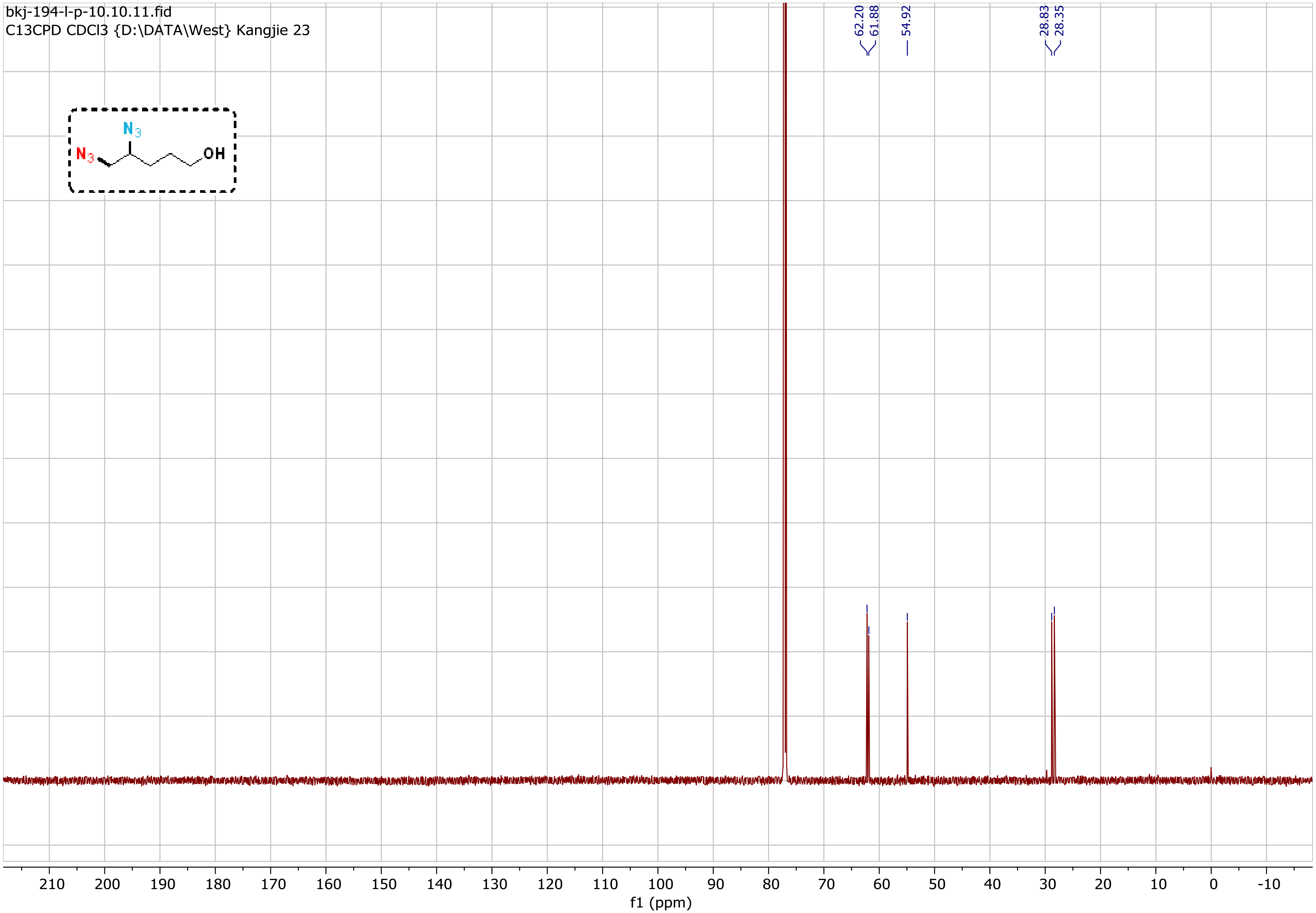

Supplementary Figure 37. ^{13}C NMR Spectrum of **18** (151 MHz, $CDCl_3$)

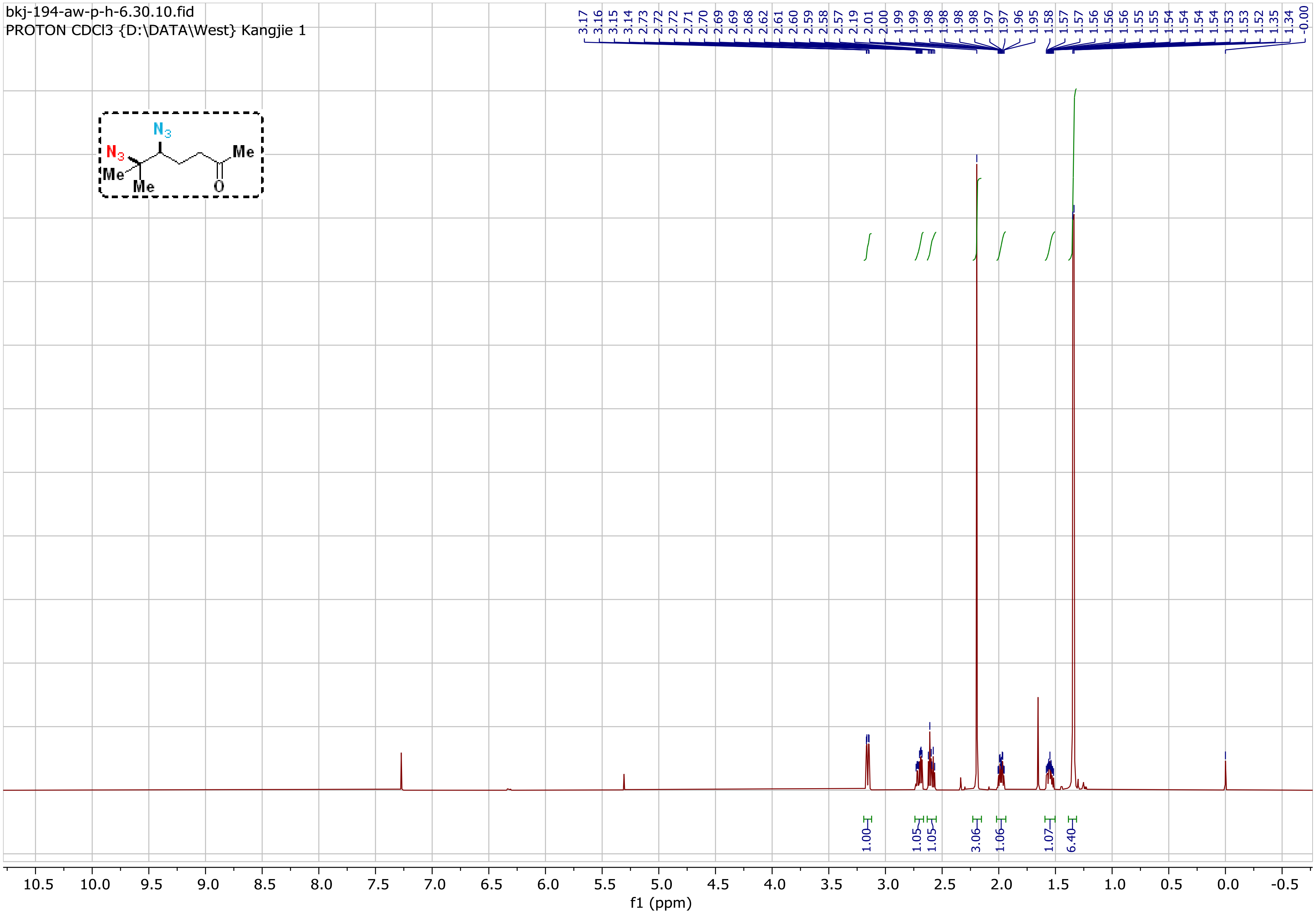

Supplementary Figure 38. ^{1}H NMR Spectrum of **19** (600 MHz, $CDCl_3$)

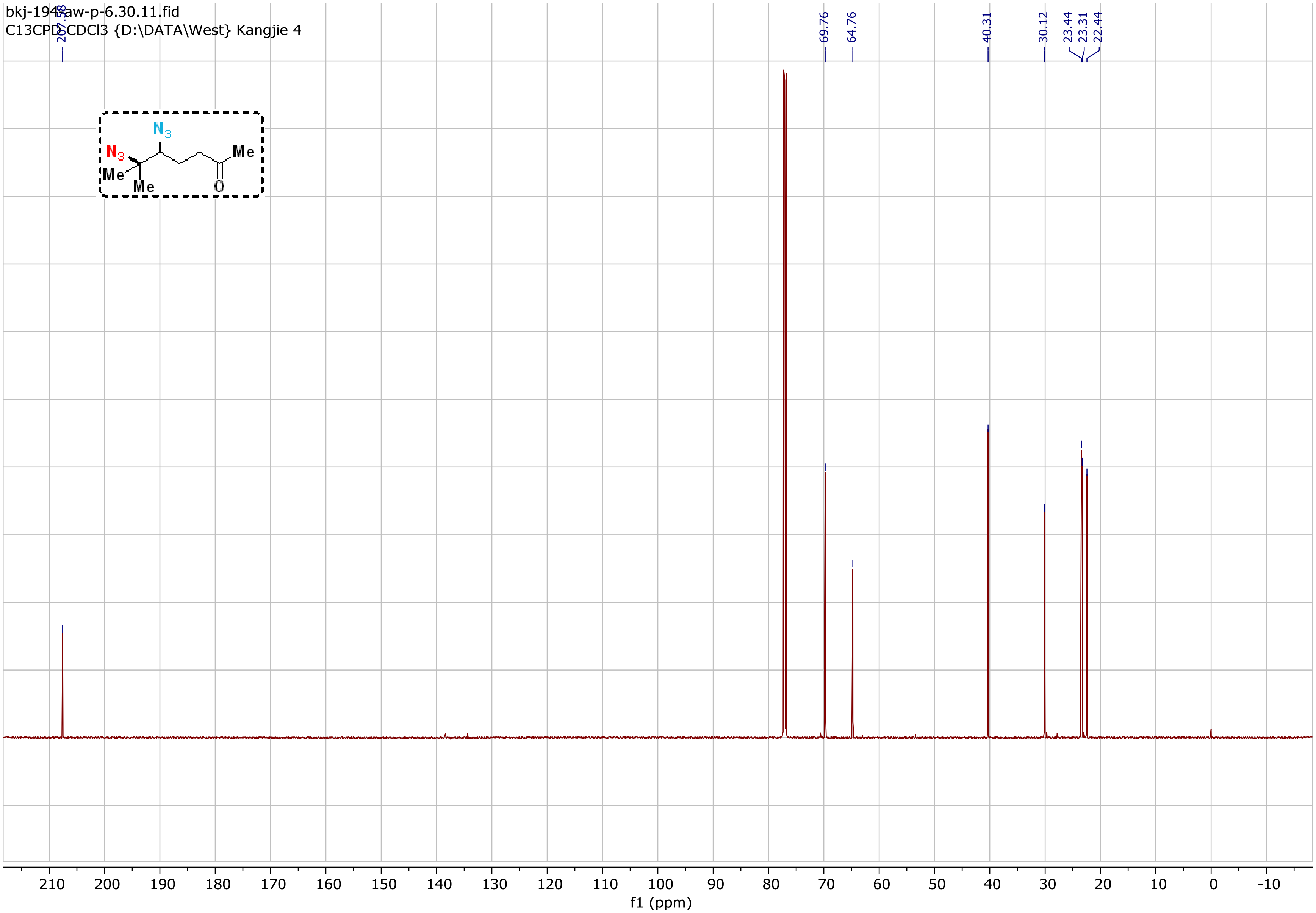

Supplementary Figure 39. ^{13}C NMR Spectrum of **19** (151 MHz, $CDCl_3$)

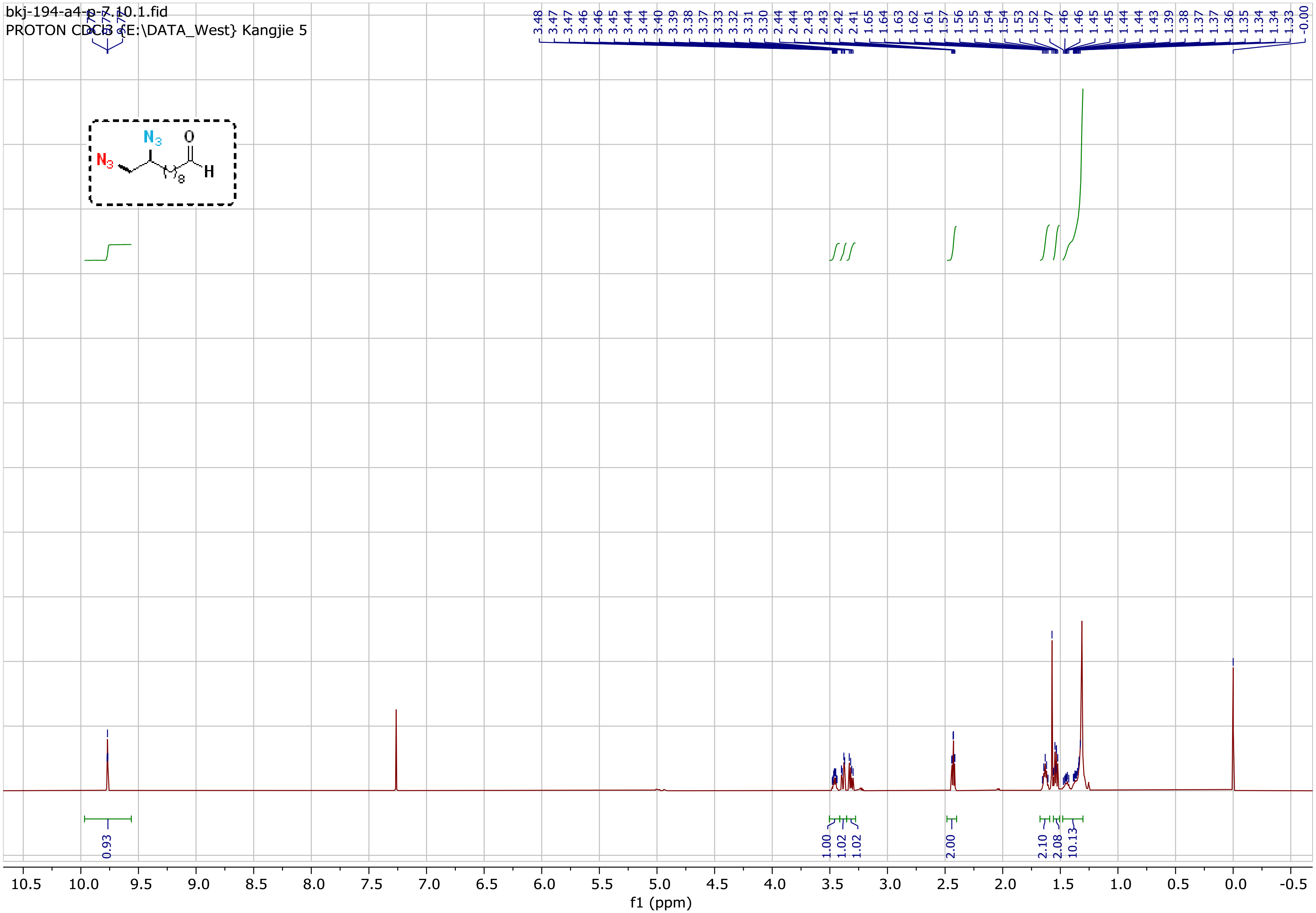

Supplementary Figure 40. ^{1}H NMR Spectrum of **20** (600 MHz, $CDCl_3$)

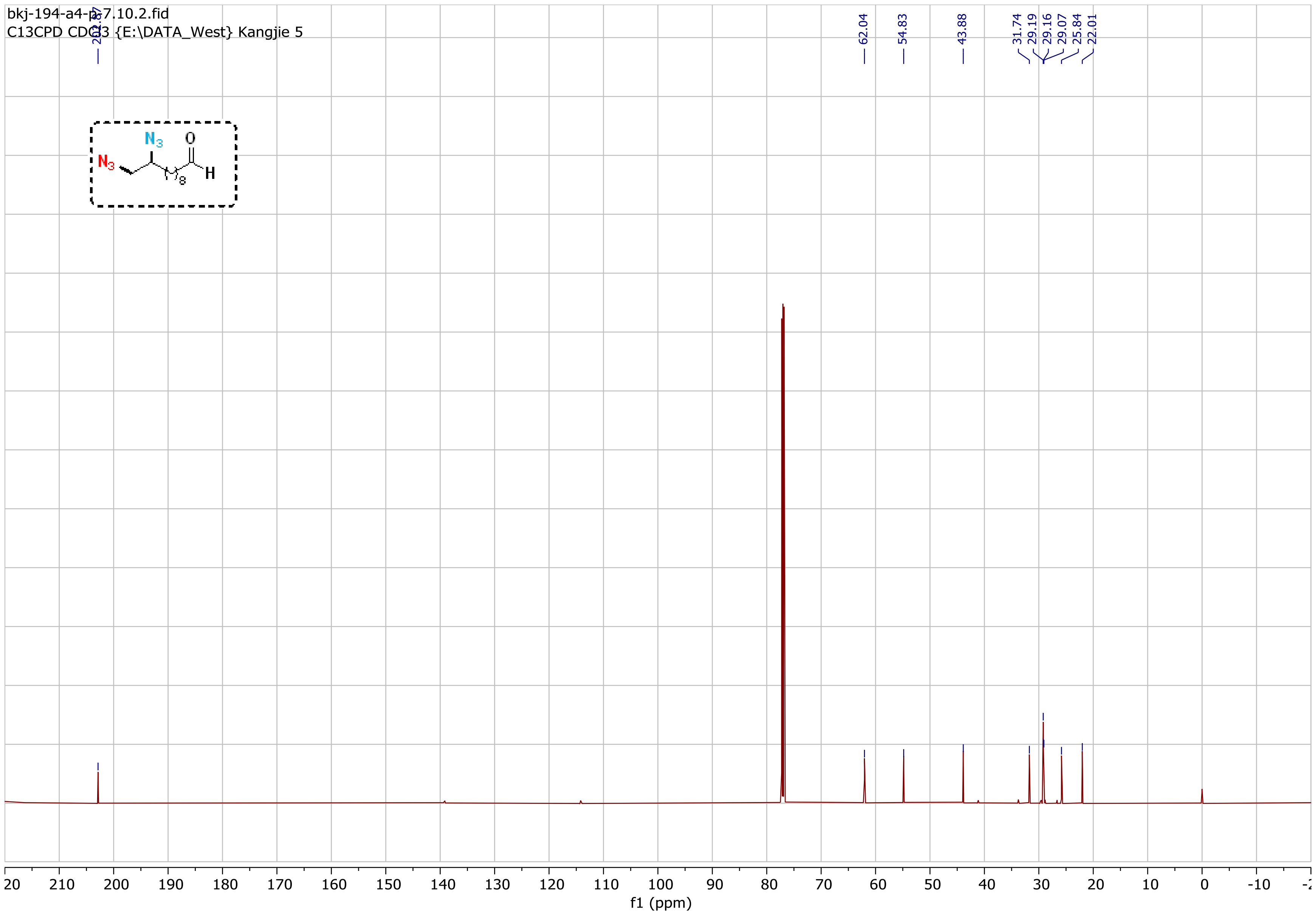

Supplementary Figure 41. ^{13}C NMR Spectrum of 2**0** (151 MHz, $CDCl_3$)

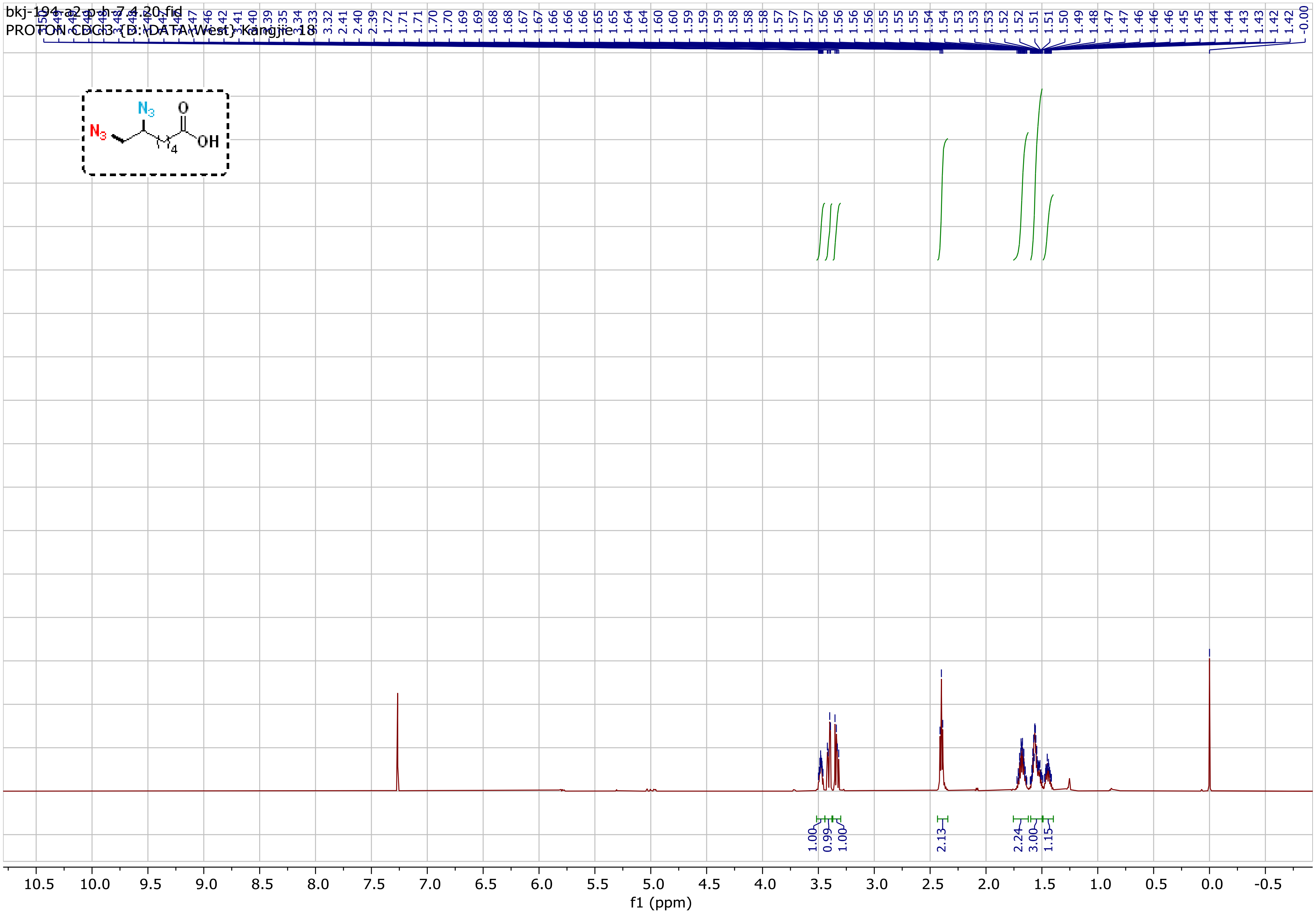

Supplementary Figure 42. ^{1}H NMR Spectrum of **21** (600 MHz, $CDCl_3$)

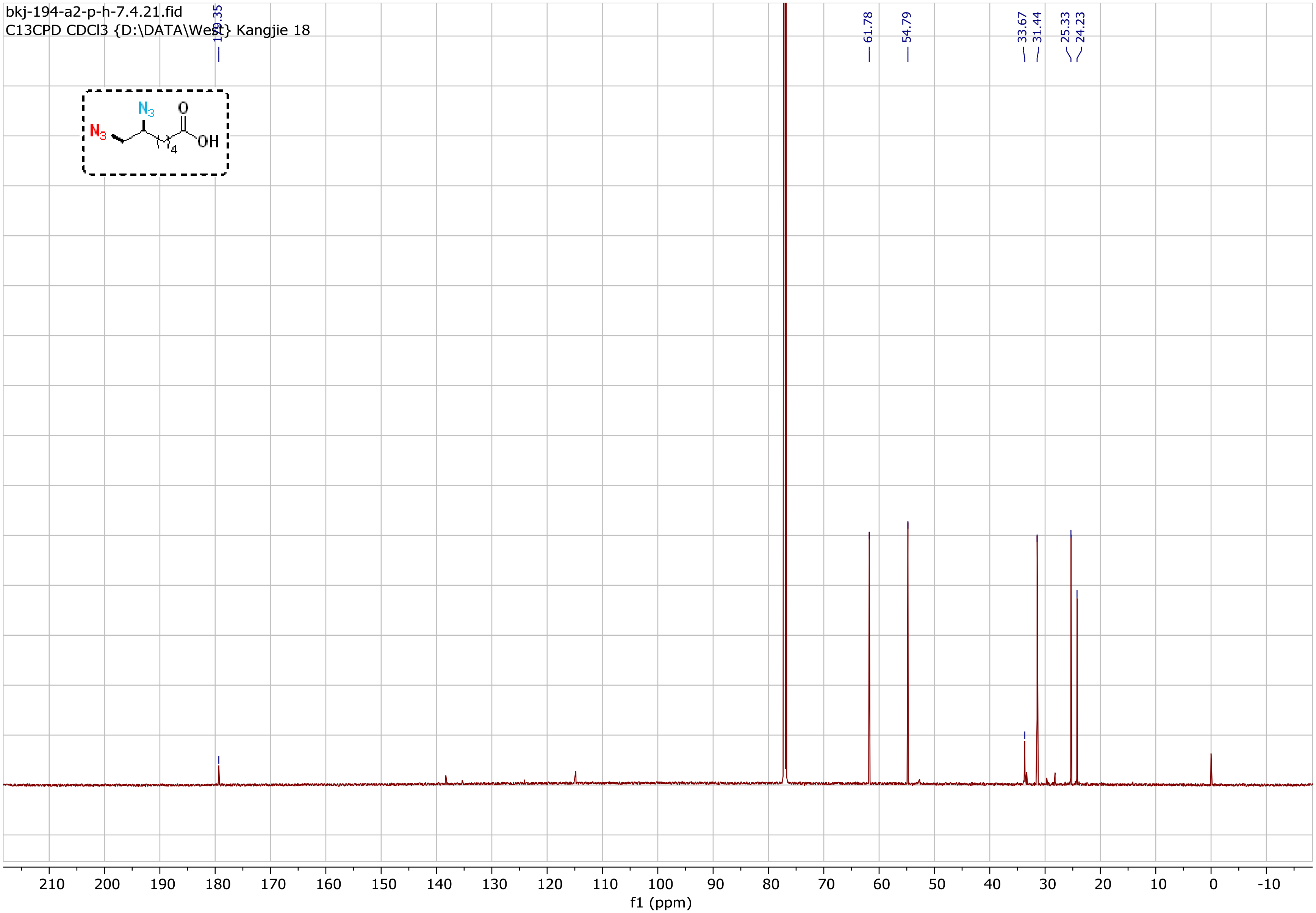

Supplementary Figure 43. ^{13}C NMR Spectrum of **21** (151 MHz, $CDCl_3$)

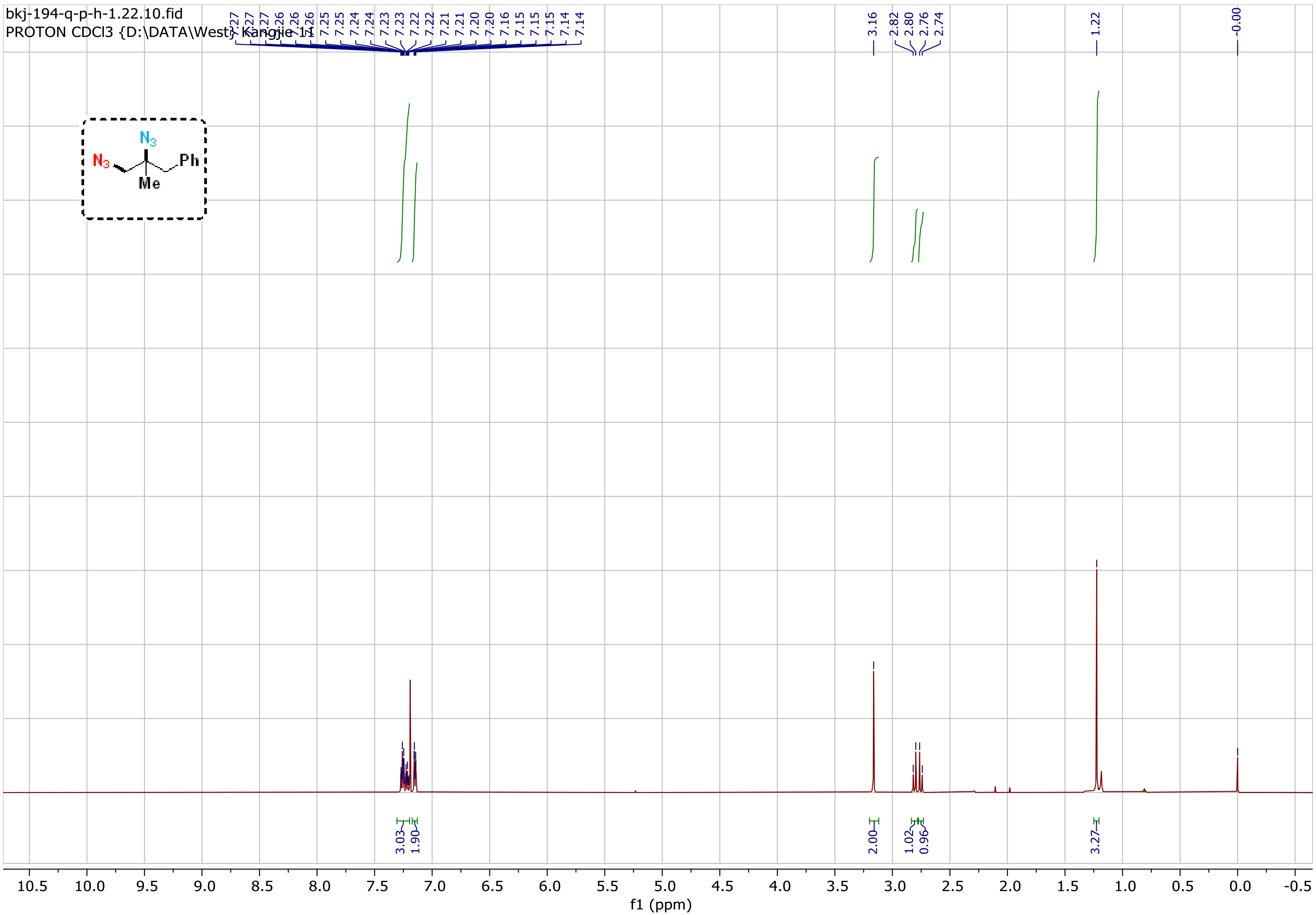

Supplementary Figure 44. ¹H NMR Spectrum of **22** (600 MHz, $CDCl_3$)

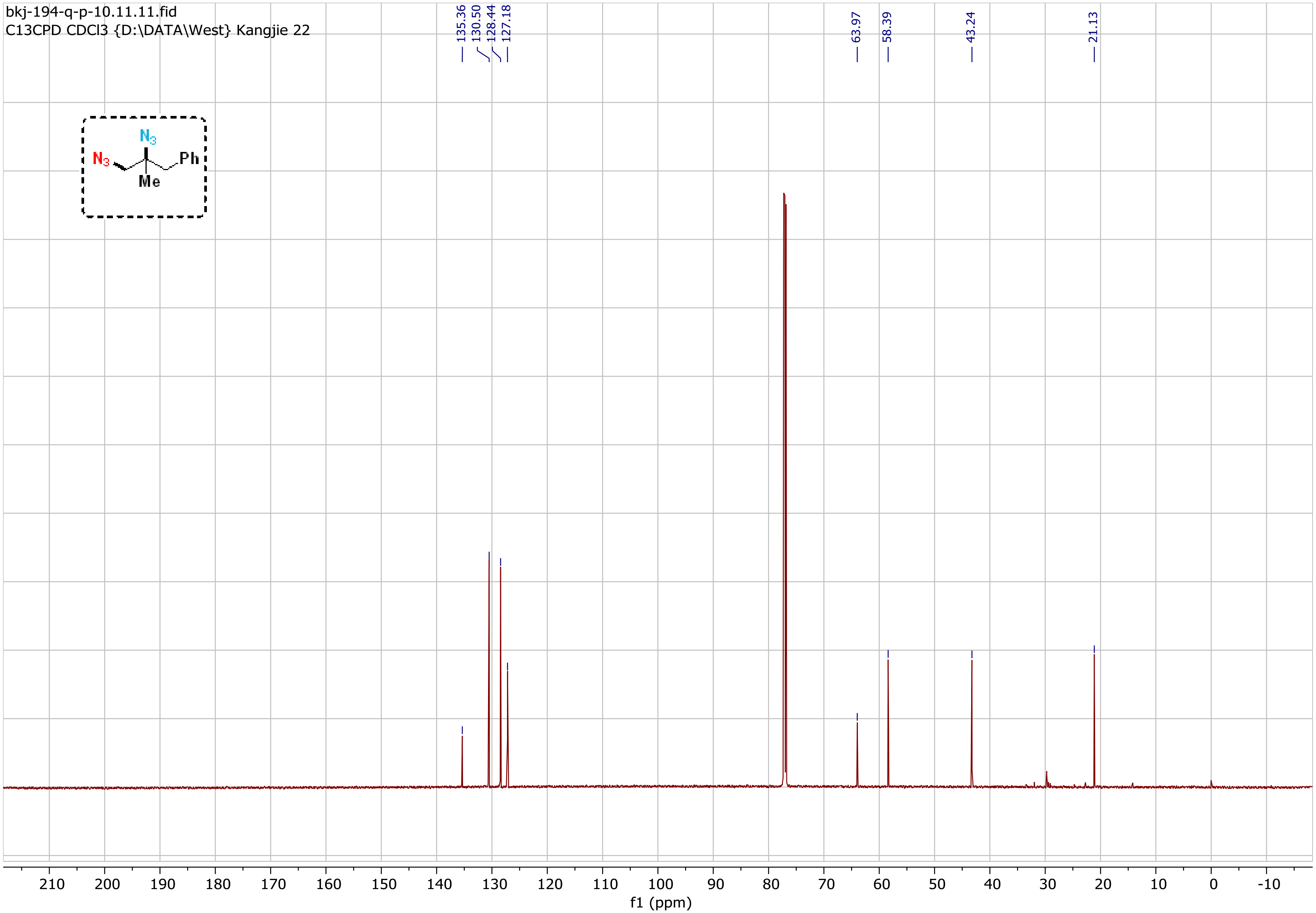

Supplementary Figure 45. ^{13}C NMR Spectrum of **22** (151 MHz, $CDCl_3$)

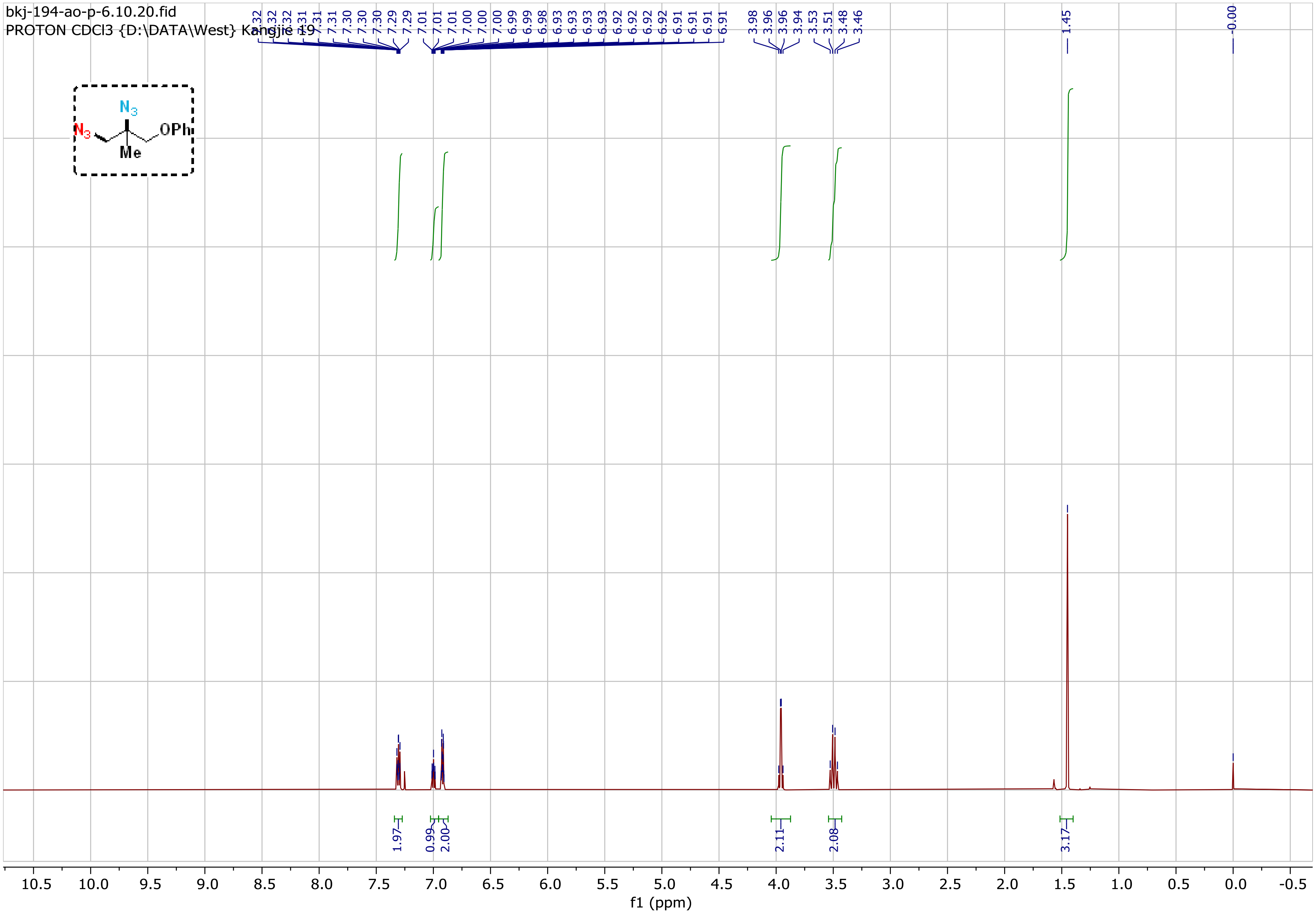

Supplementary Figure 46. ^{1}H NMR Spectrum of **23** (600 MHz, $CDCl_3$)

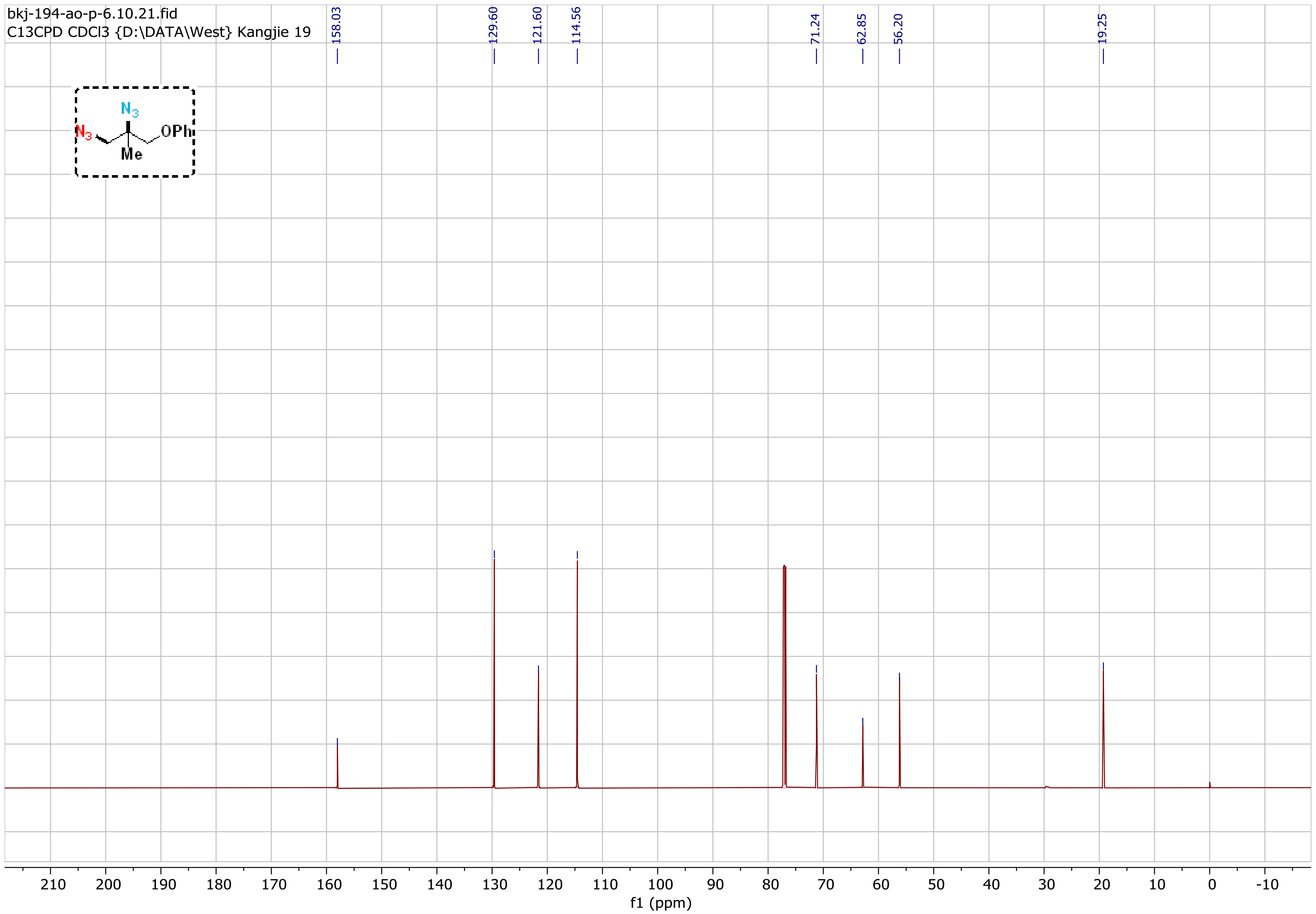

Supplementary Figure 47. ^{13}C NMR Spectrum of **23** (151 MHz, $CDCl_3$)

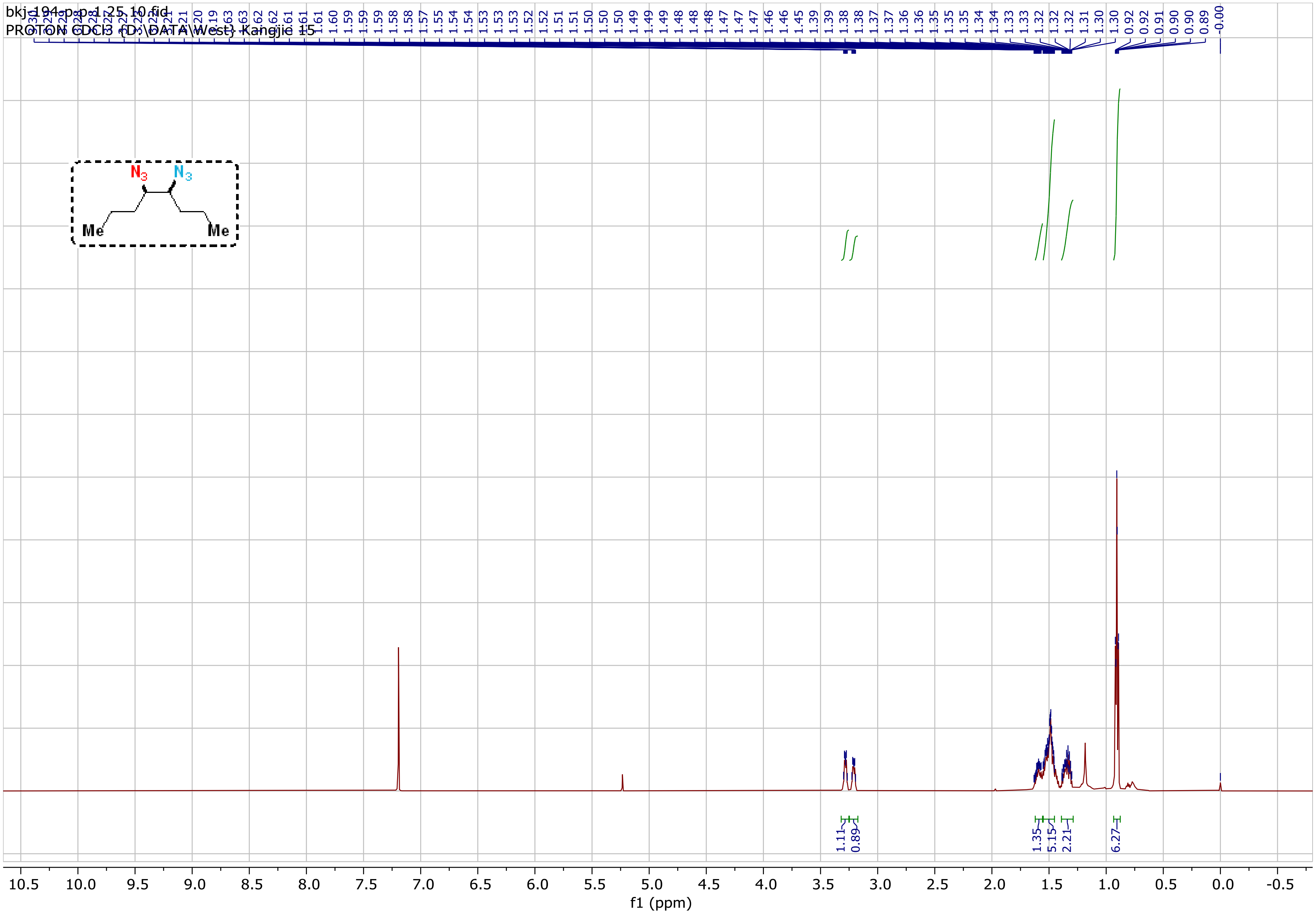

Supplementary Figure 48. ^{1}H NMR Spectrum of **24** (600 MHz, $CDCl_3$)

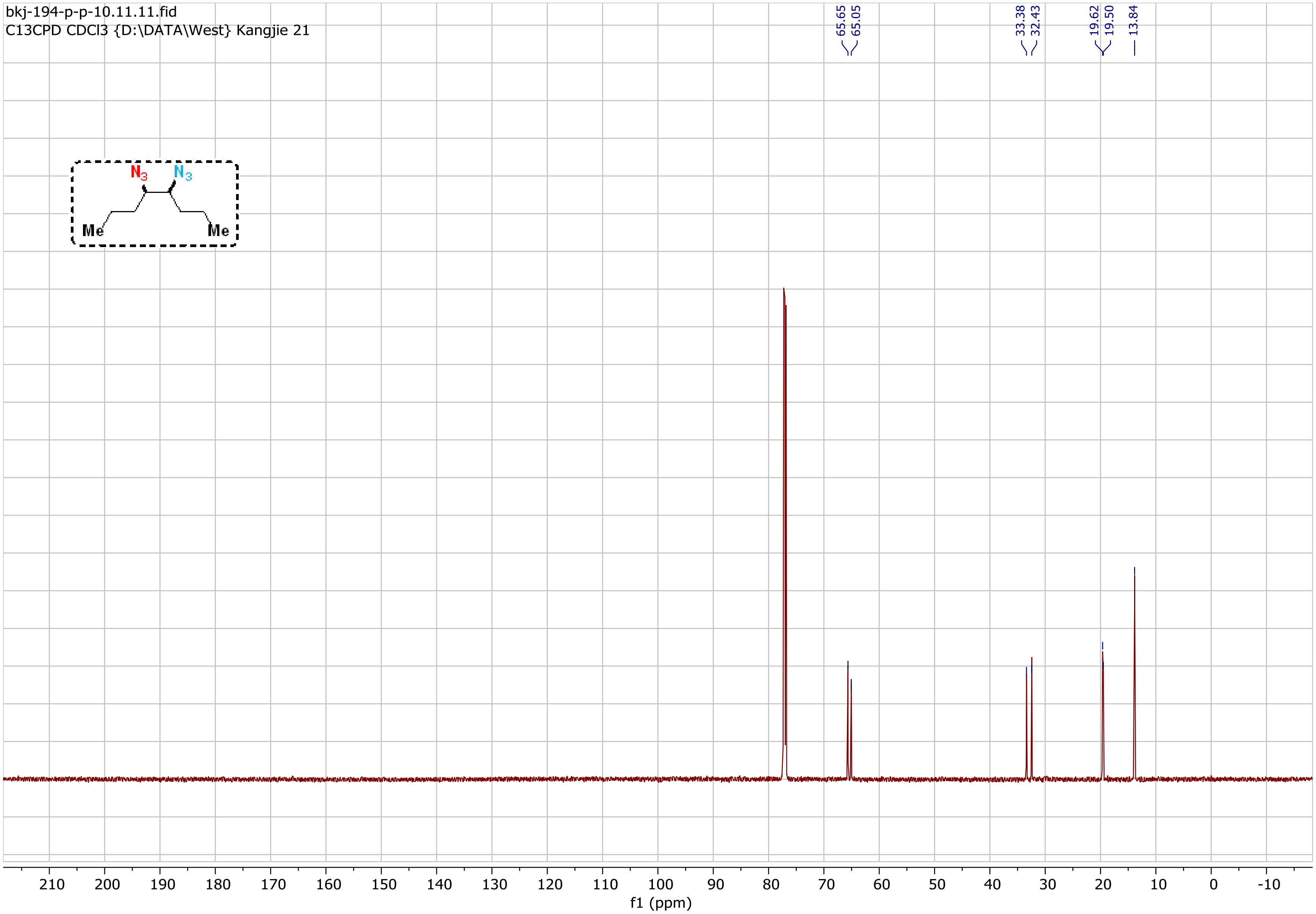

Supplementary Figure 49. ^{13}C NMR Spectrum of **24** (151 MHz, $CDCl_3$)

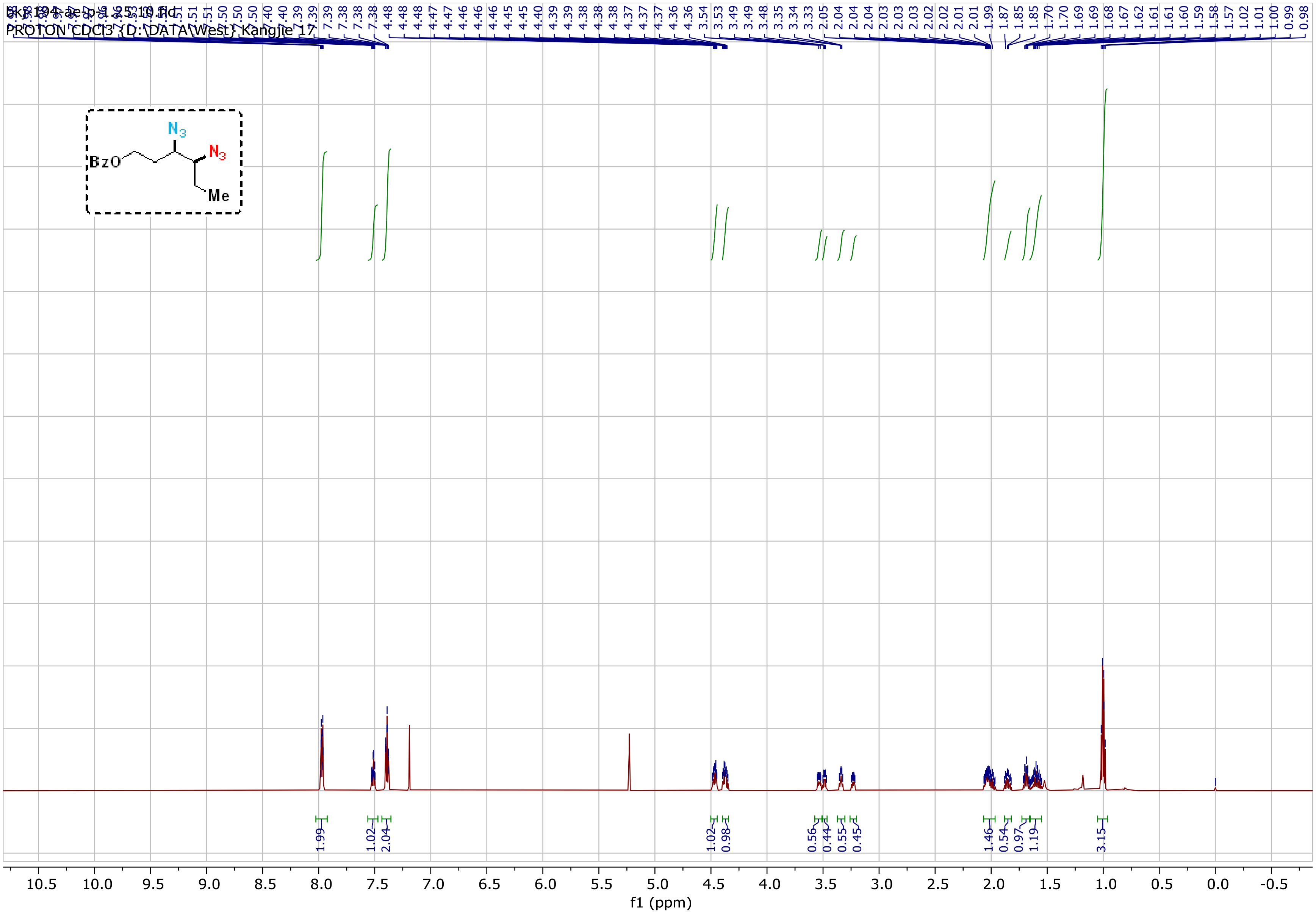

Supplementary Figure 50. ^{1}H NMR Spectrum of **25** (600 MHz, $CDCl_3$)

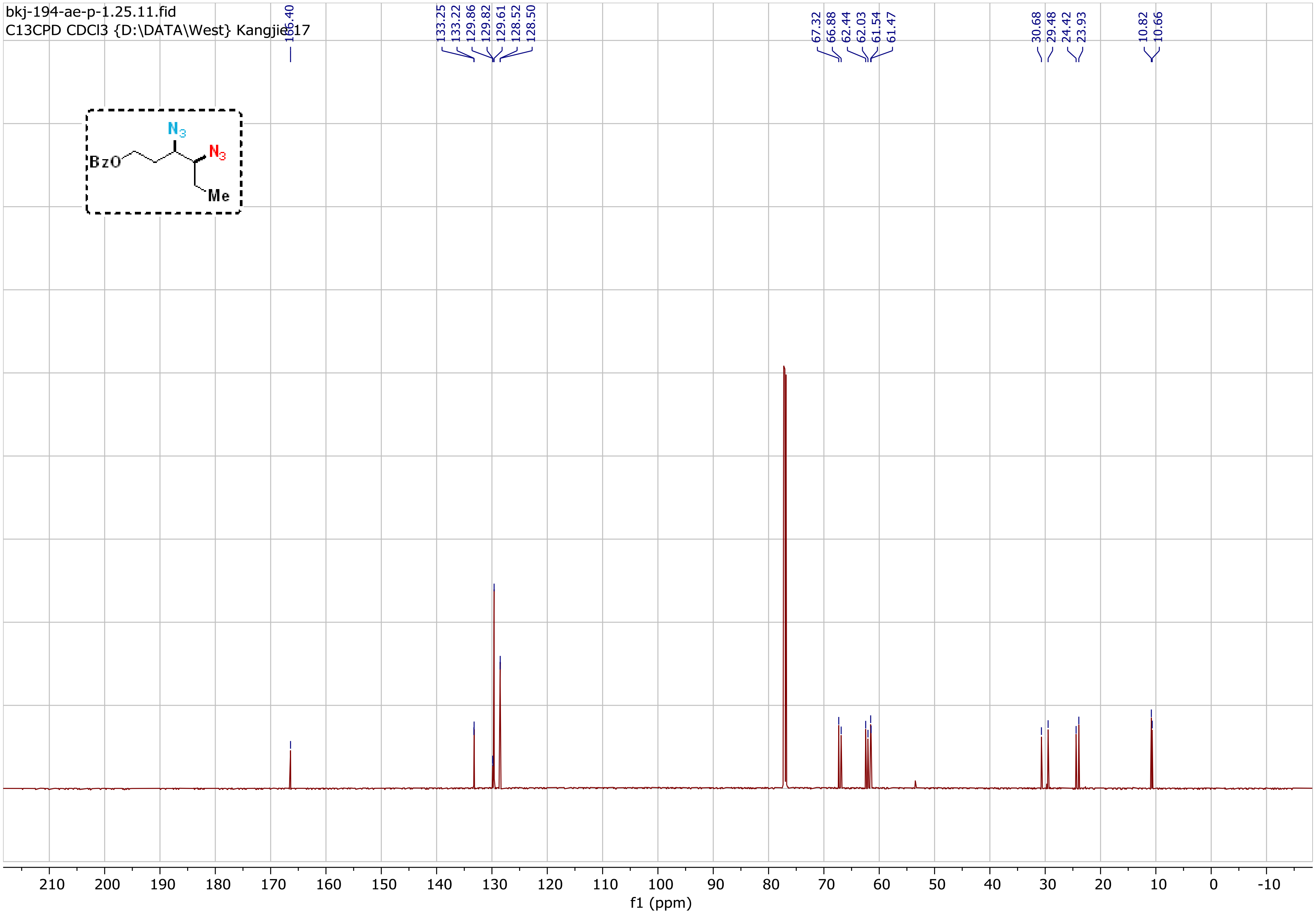

Supplementary Figure 51. ^{13}C NMR Spectrum of **25** (151 MHz, $CDCl_3$)

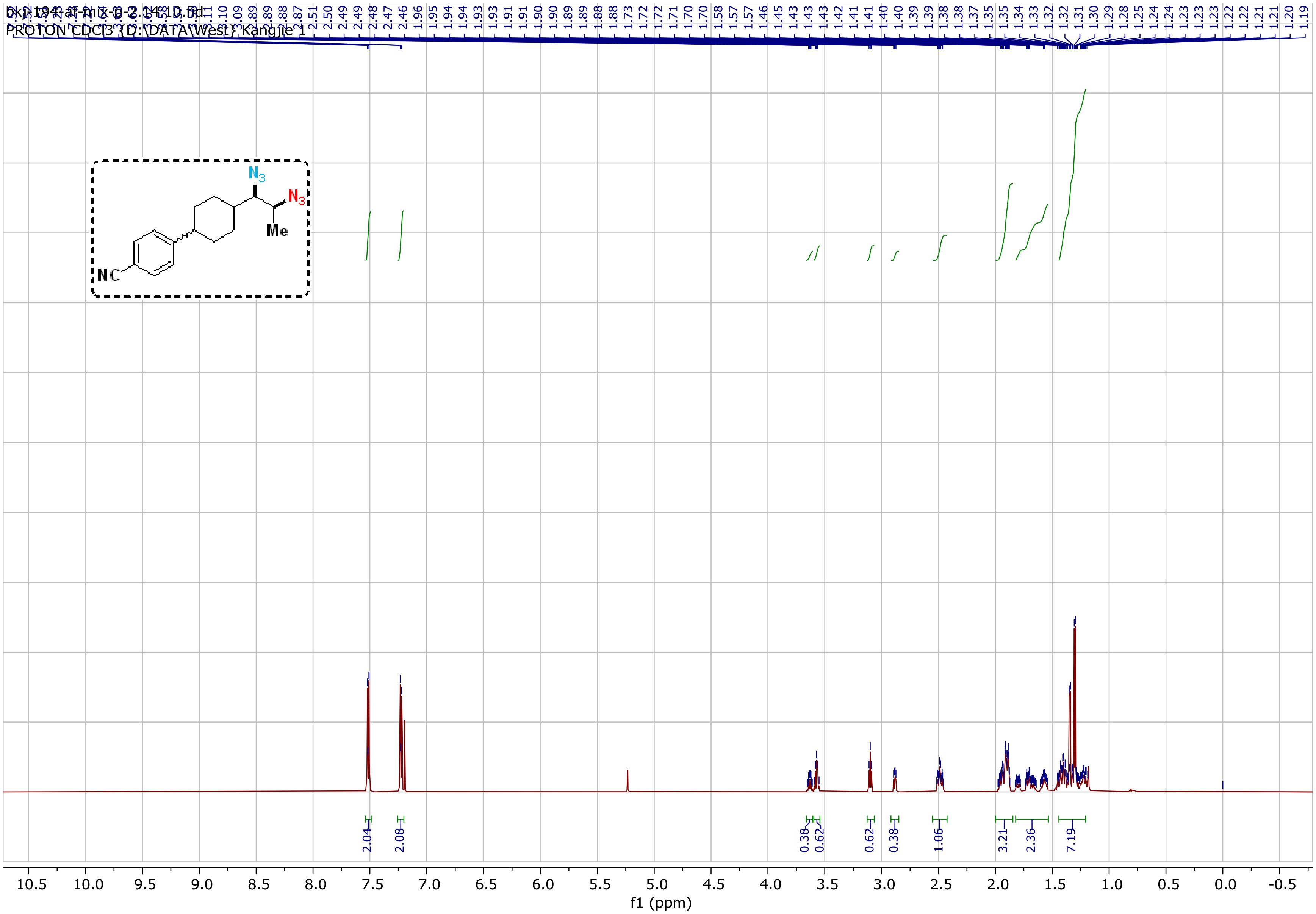

Supplementary Figure 52. ^{1}H NMR Spectrum of **26** (600 MHz, $CDCl_3$)

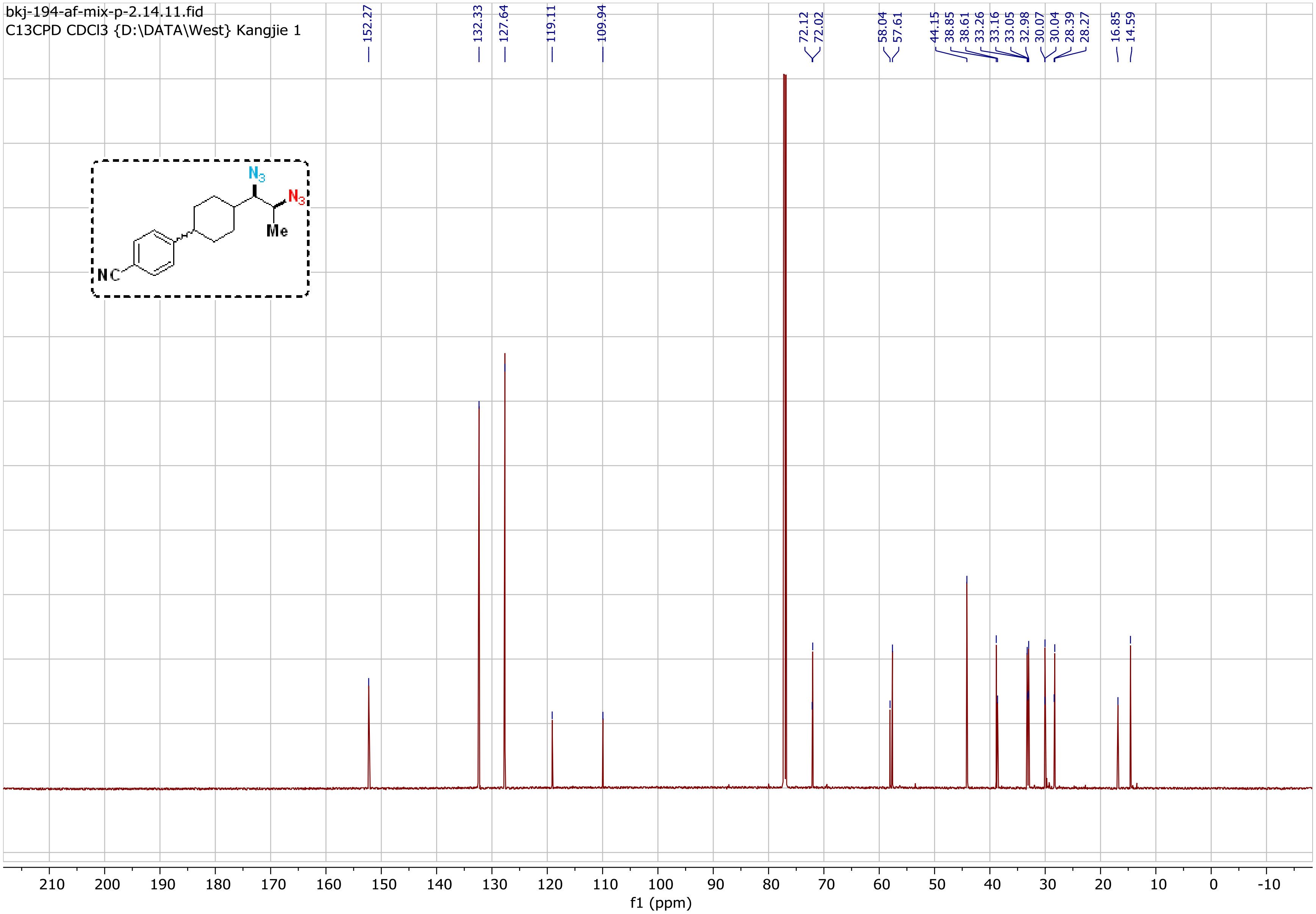

Supplementary Figure 53. ^{13}C NMR Spectrum of **26** (151 MHz, $CDCl_3$)

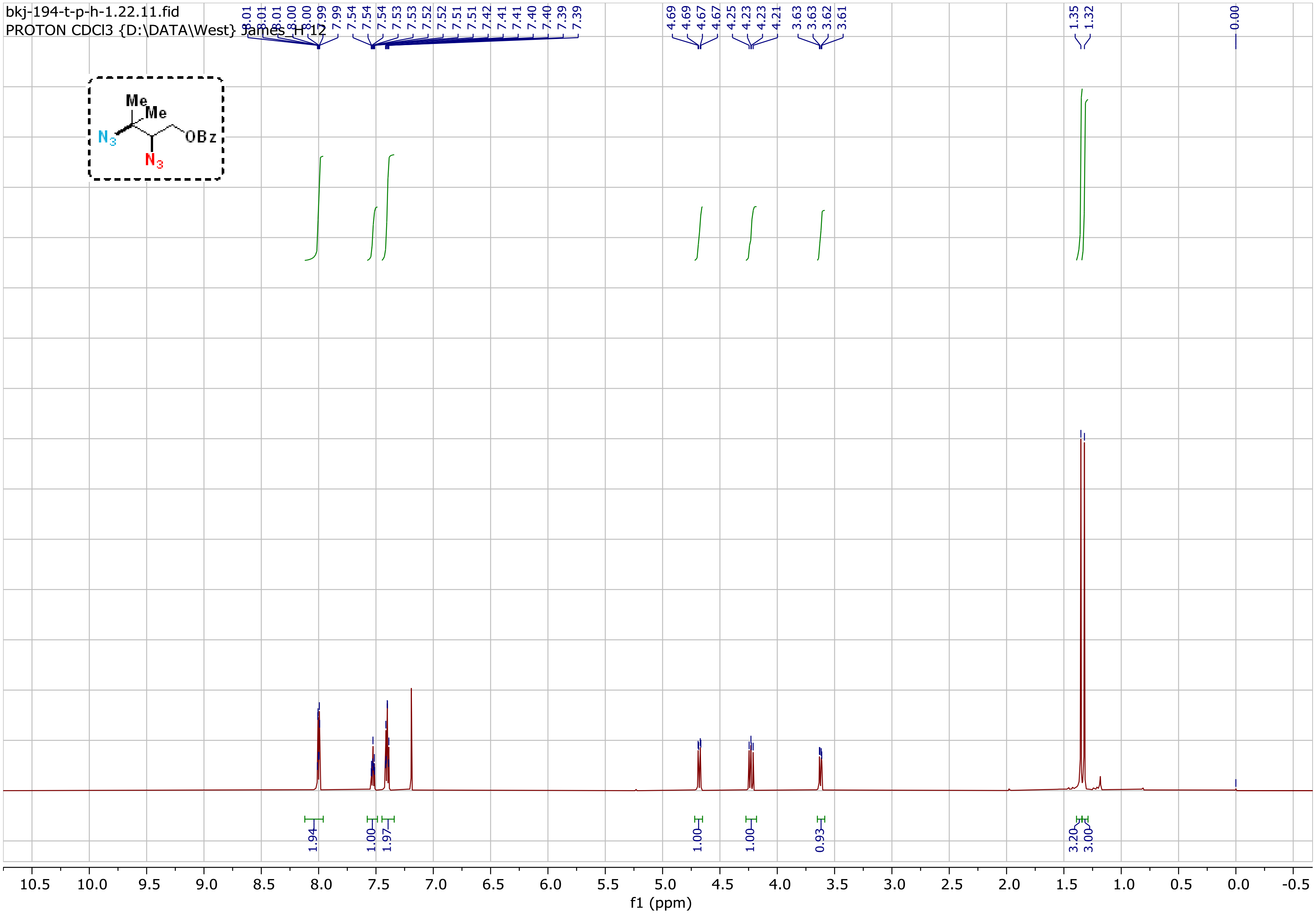

Supplementary Figure 54. ^{1}H NMR Spectrum of **27** (600 MHz, $CDCl_3$)

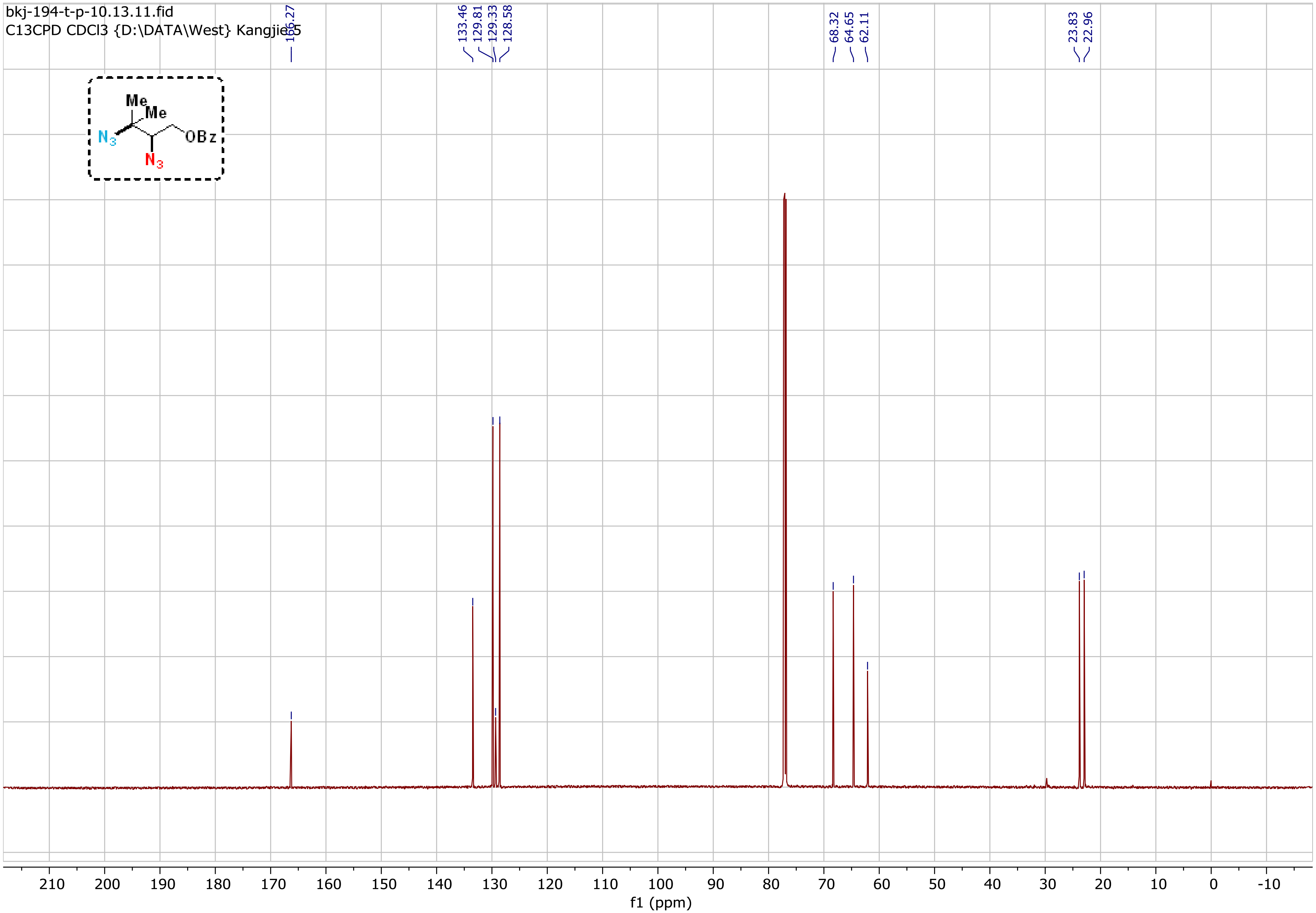

Supplementary Figure 55. ^{13}C NMR Spectrum of **27** (151 MHz, $CDCl_3$)

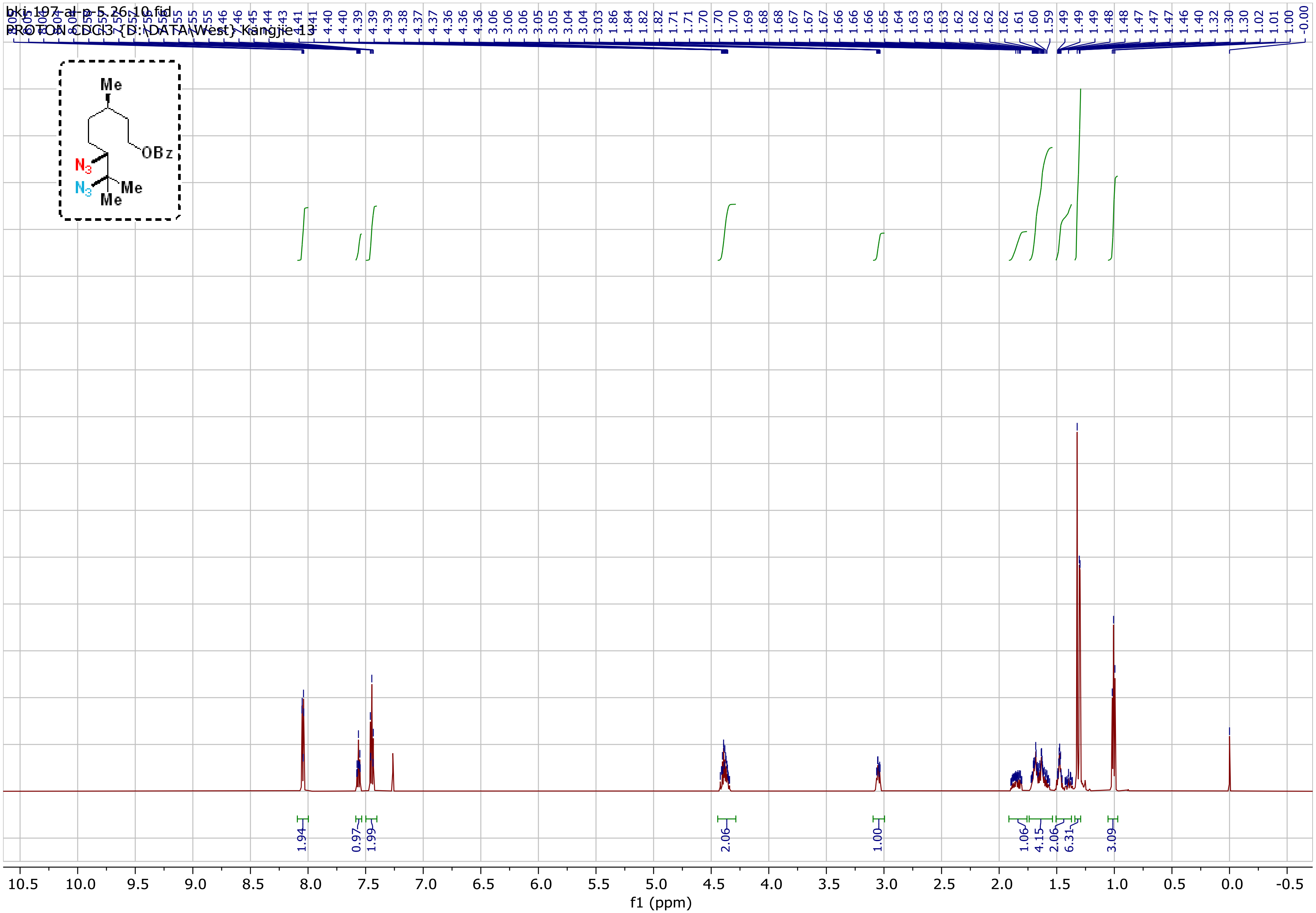

Supplementary Figure 56. ^{1}H NMR Spectrum of **28** (600 MHz, $CDCl_3$)

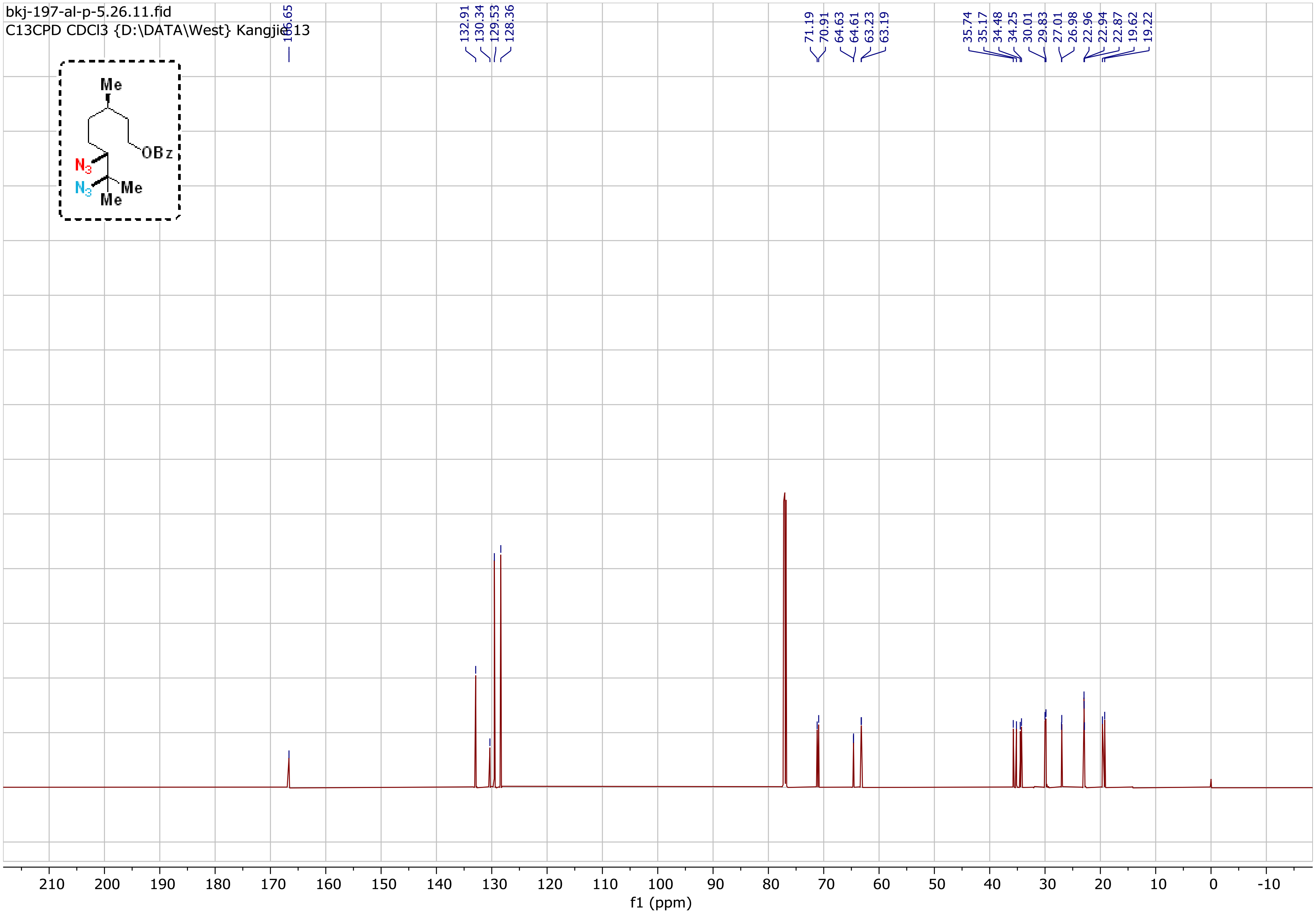

Supplementary Figure 57. ^{13}C NMR Spectrum of **28** (151 MHz, $CDCl_3$)

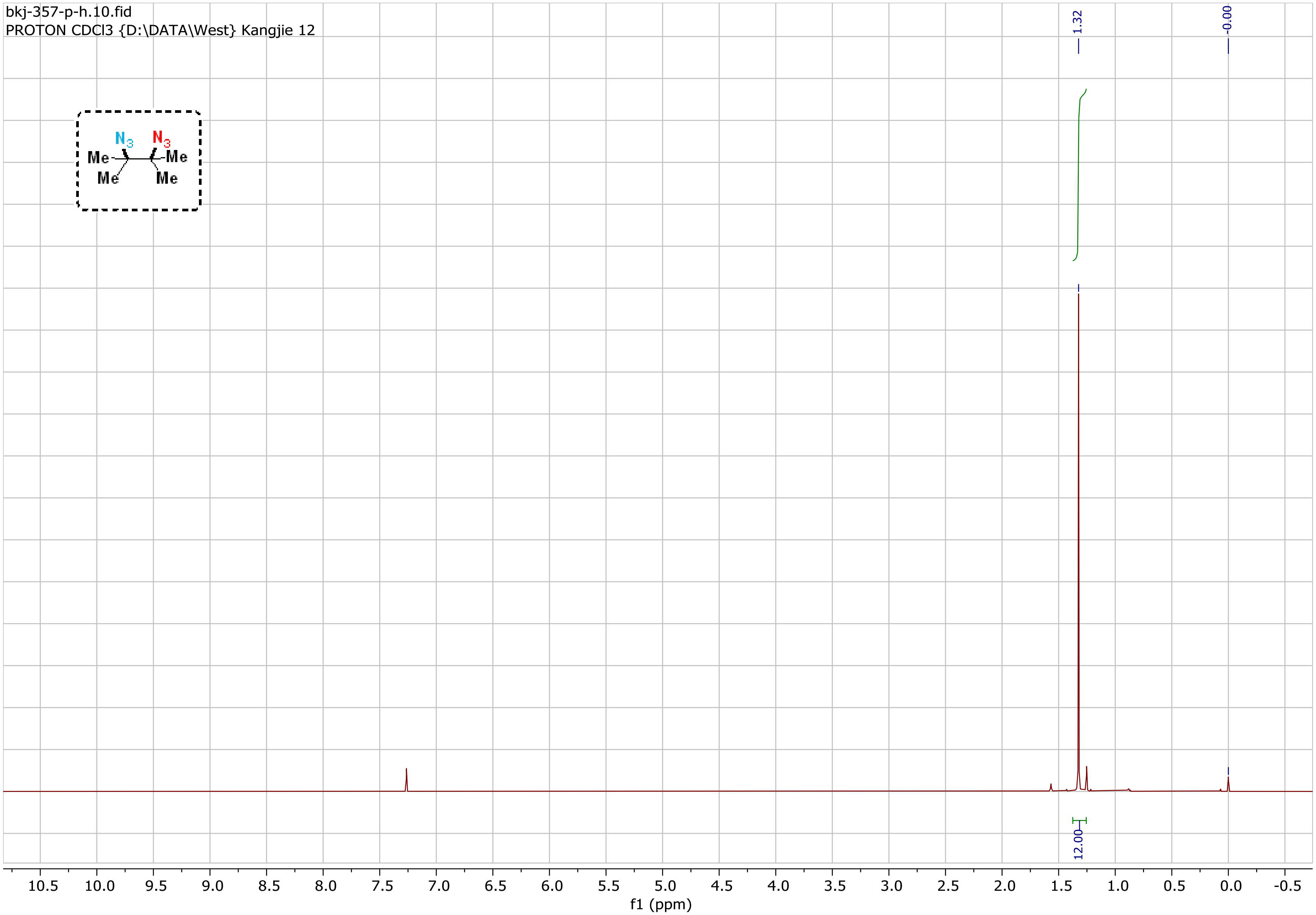

Supplementary Figure 58. ^{1}H NMR Spectrum of **29** (600 MHz, $CDCl_3$)

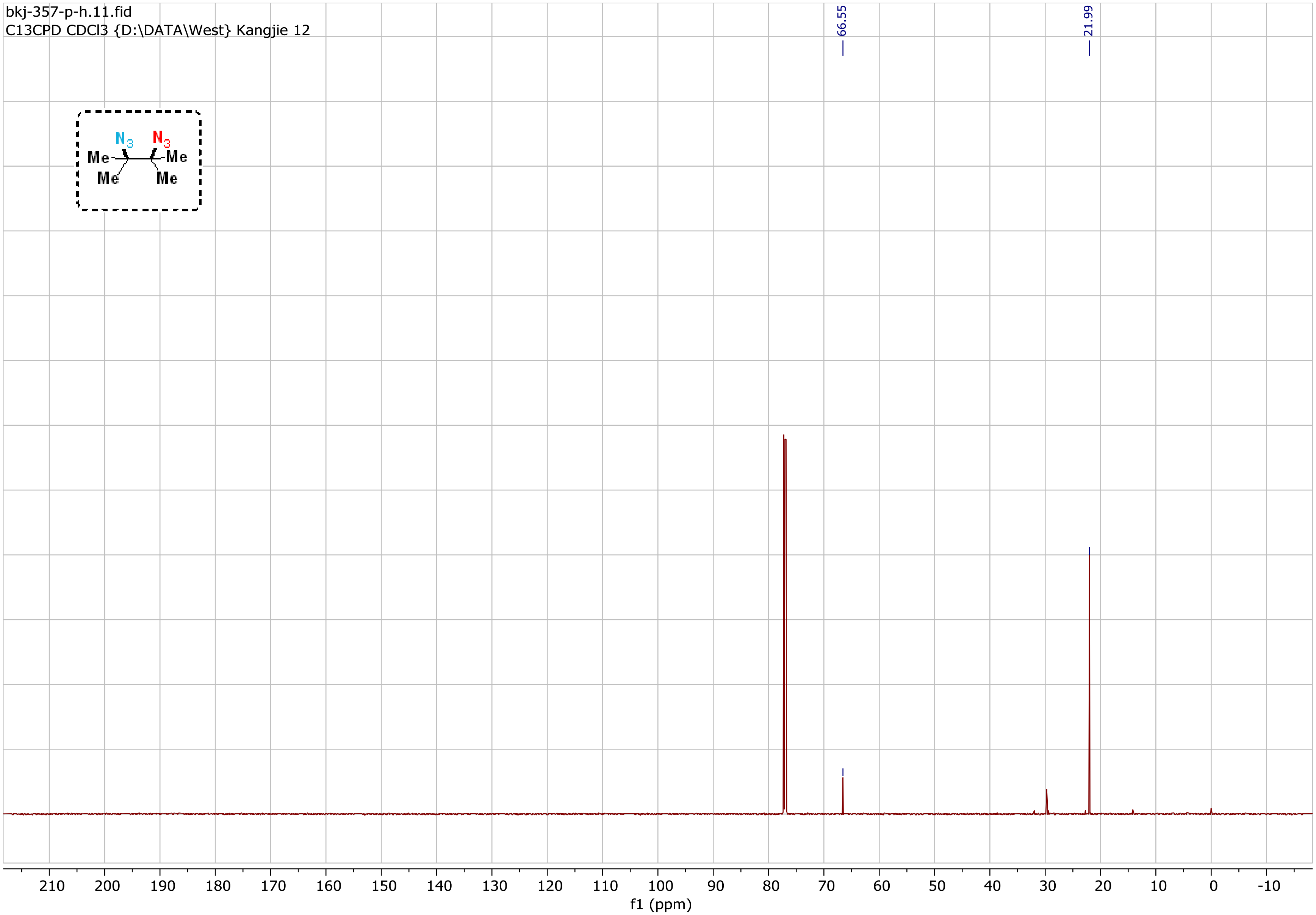

Supplementary Figure 59. ^{13}C NMR Spectrum of **29** (151 MHz, $CDCl_3$)

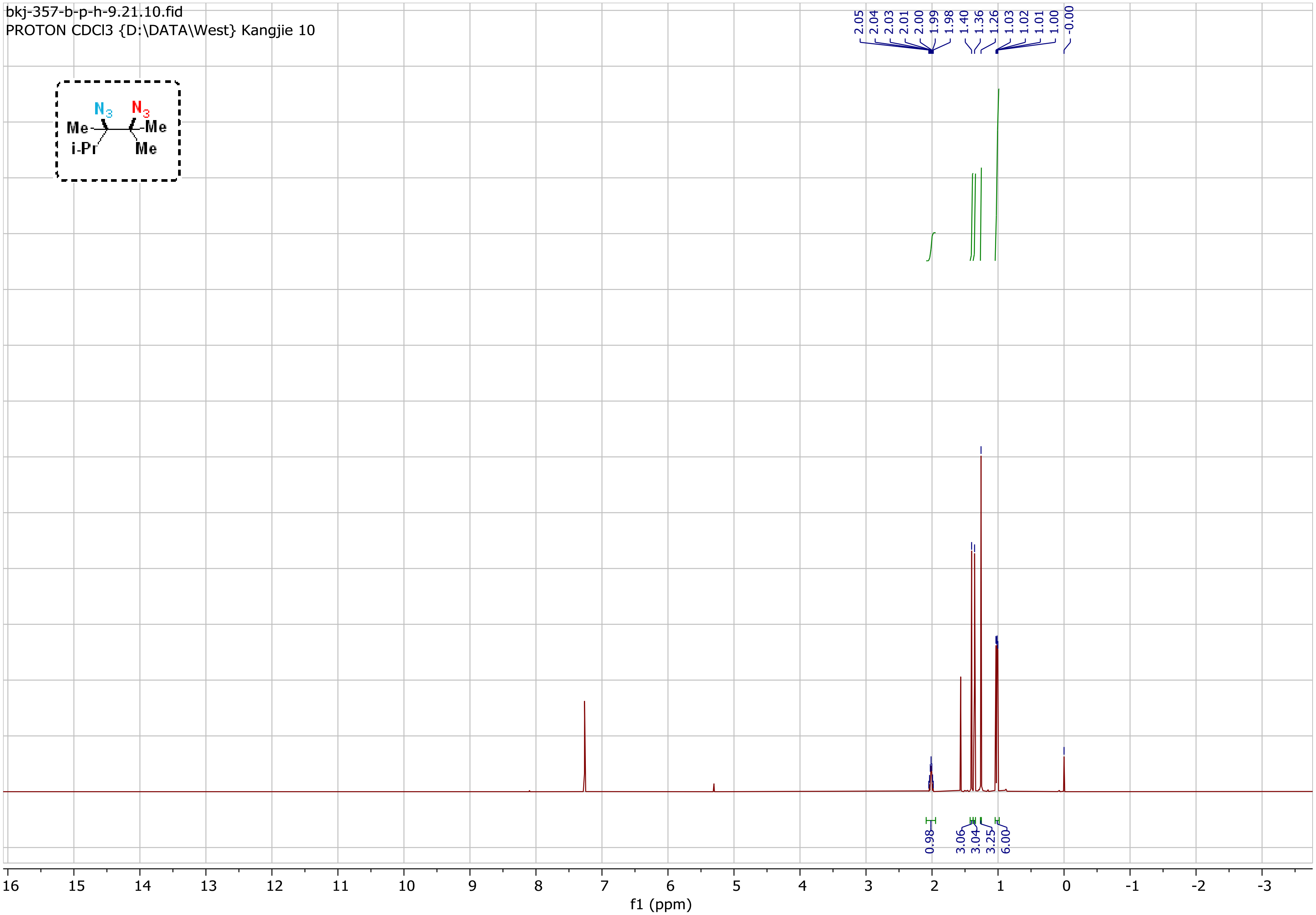

Supplementary Figure 60. ^{1}H NMR Spectrum of **30** (600 MHz, $CDCl_3$)

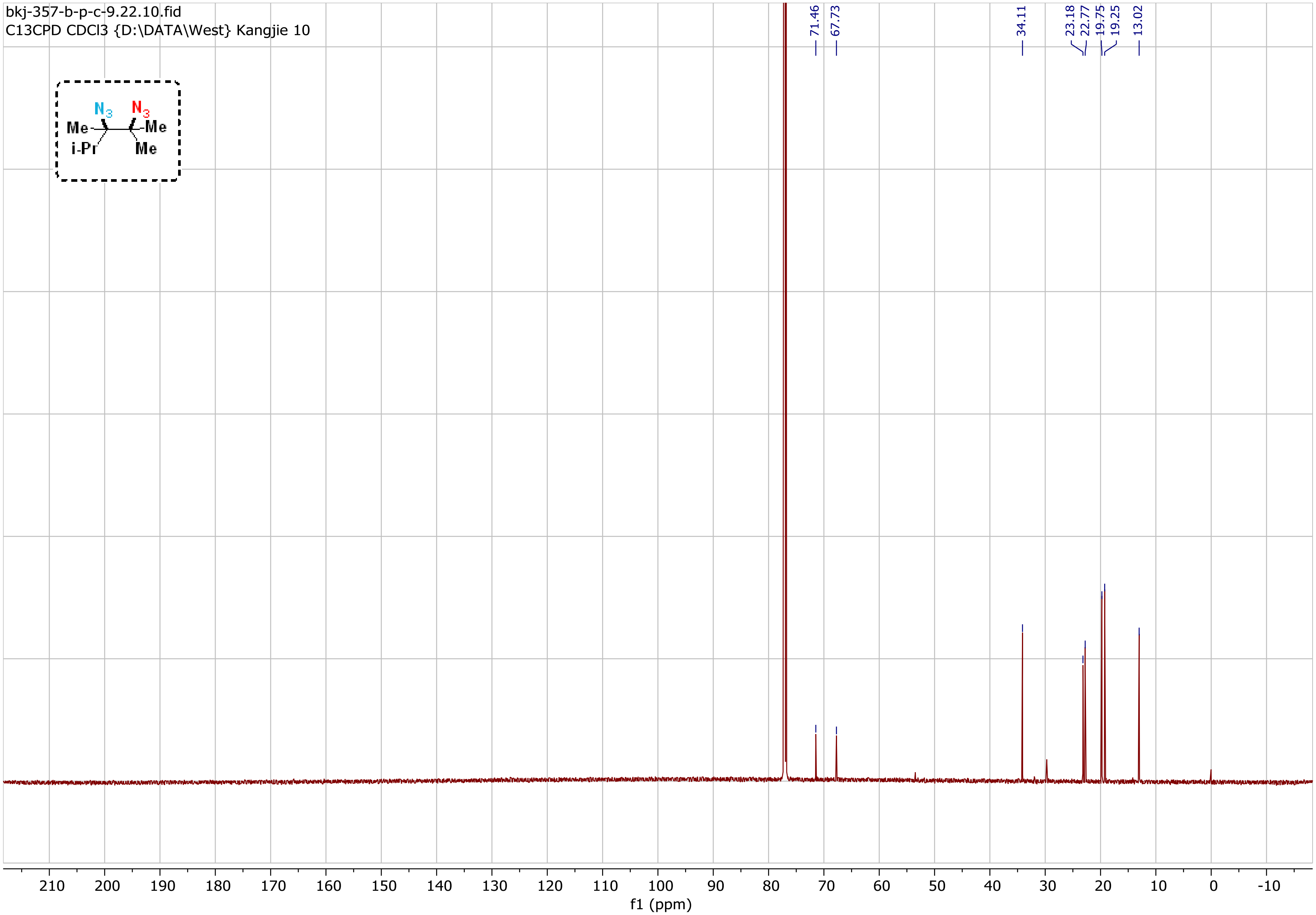

Supplementary Figure 61. ^{13}C NMR Spectrum of **30** (151 MHz, $CDCl_3$)

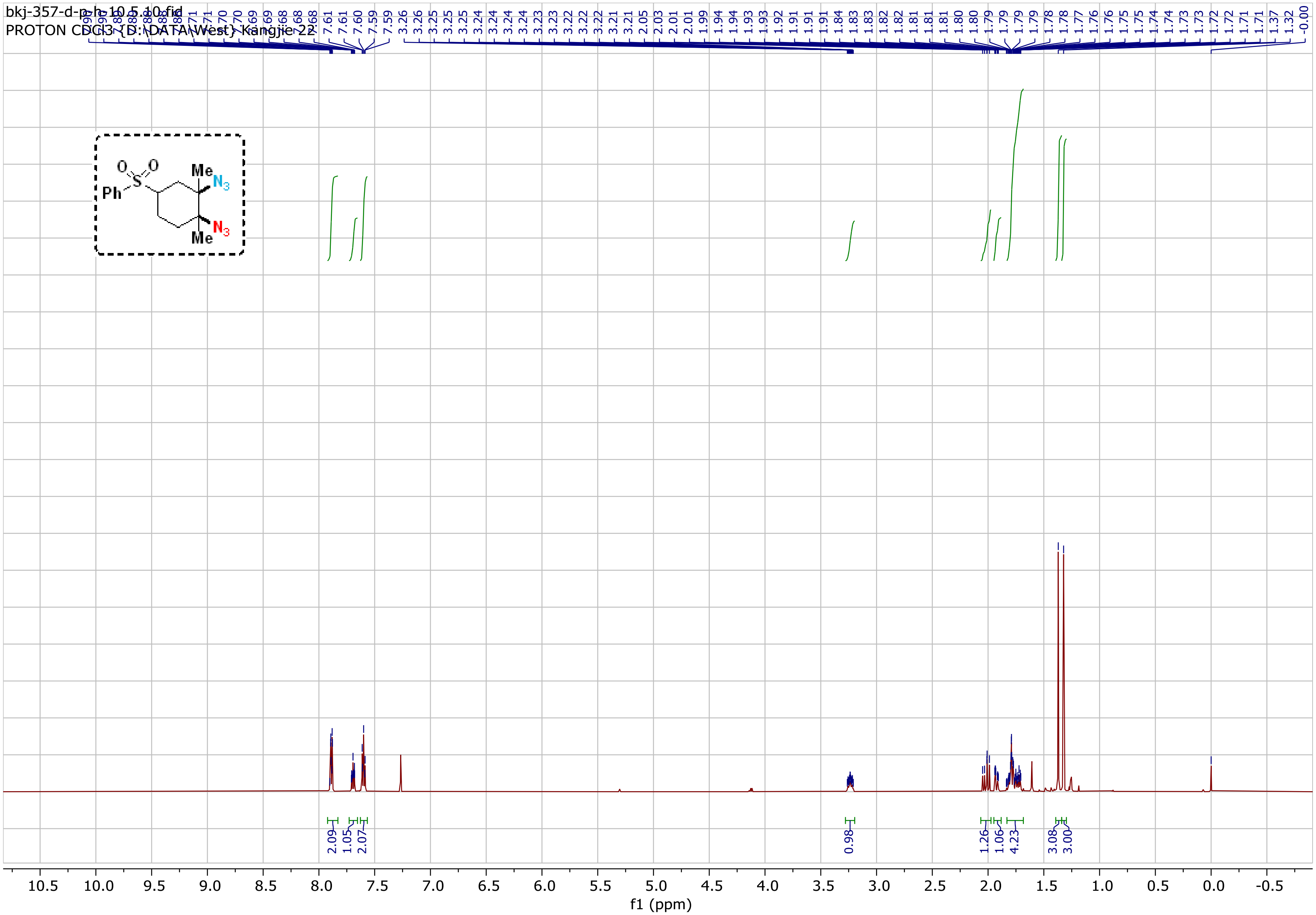

Supplementary Figure 62. ^{1}H NMR Spectrum of **31** (600 MHz, $CDCl_3$)

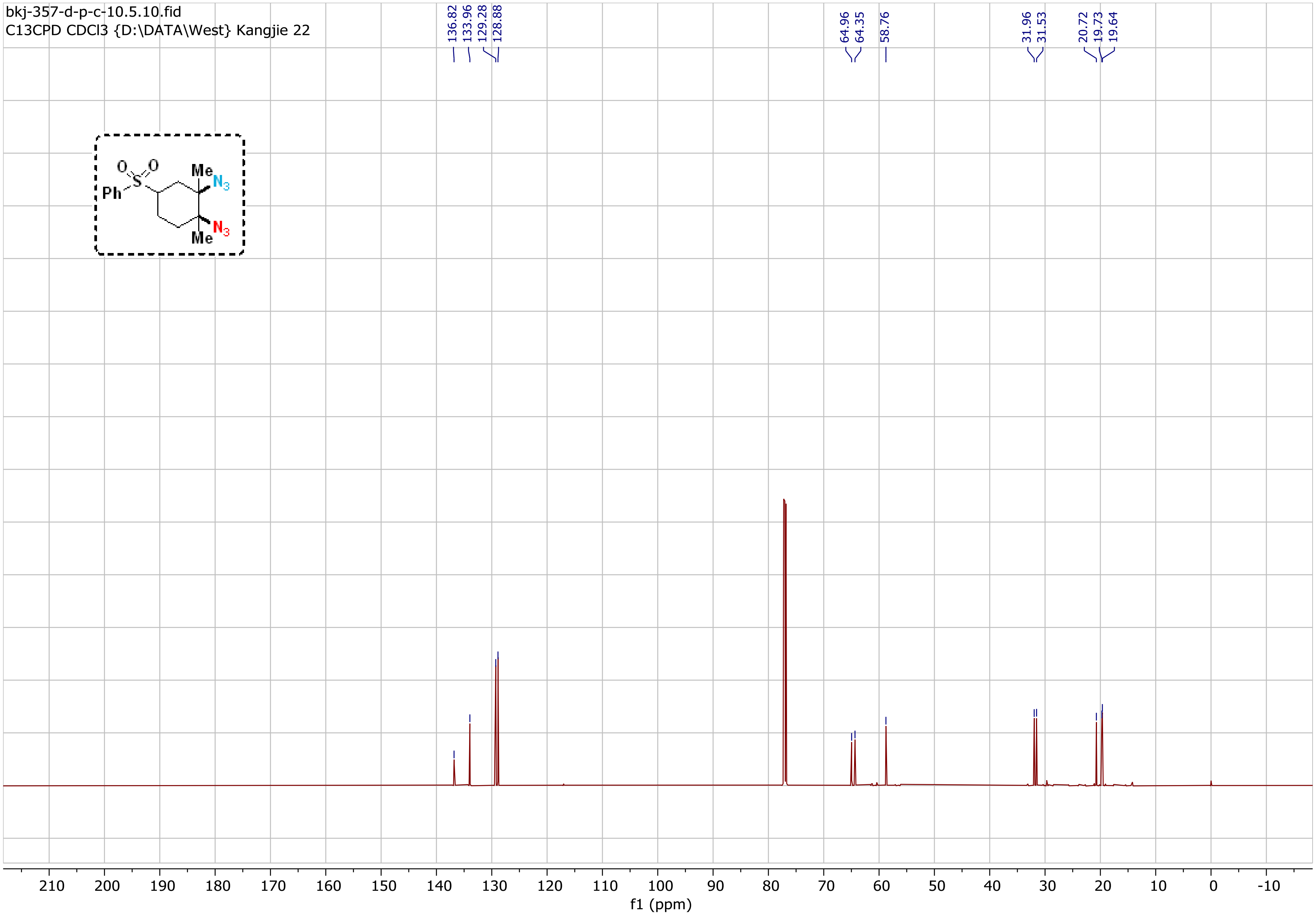

Supplementary Figure 63. ^{13}C NMR Spectrum of **31** (151 MHz, $CDCl_3$)

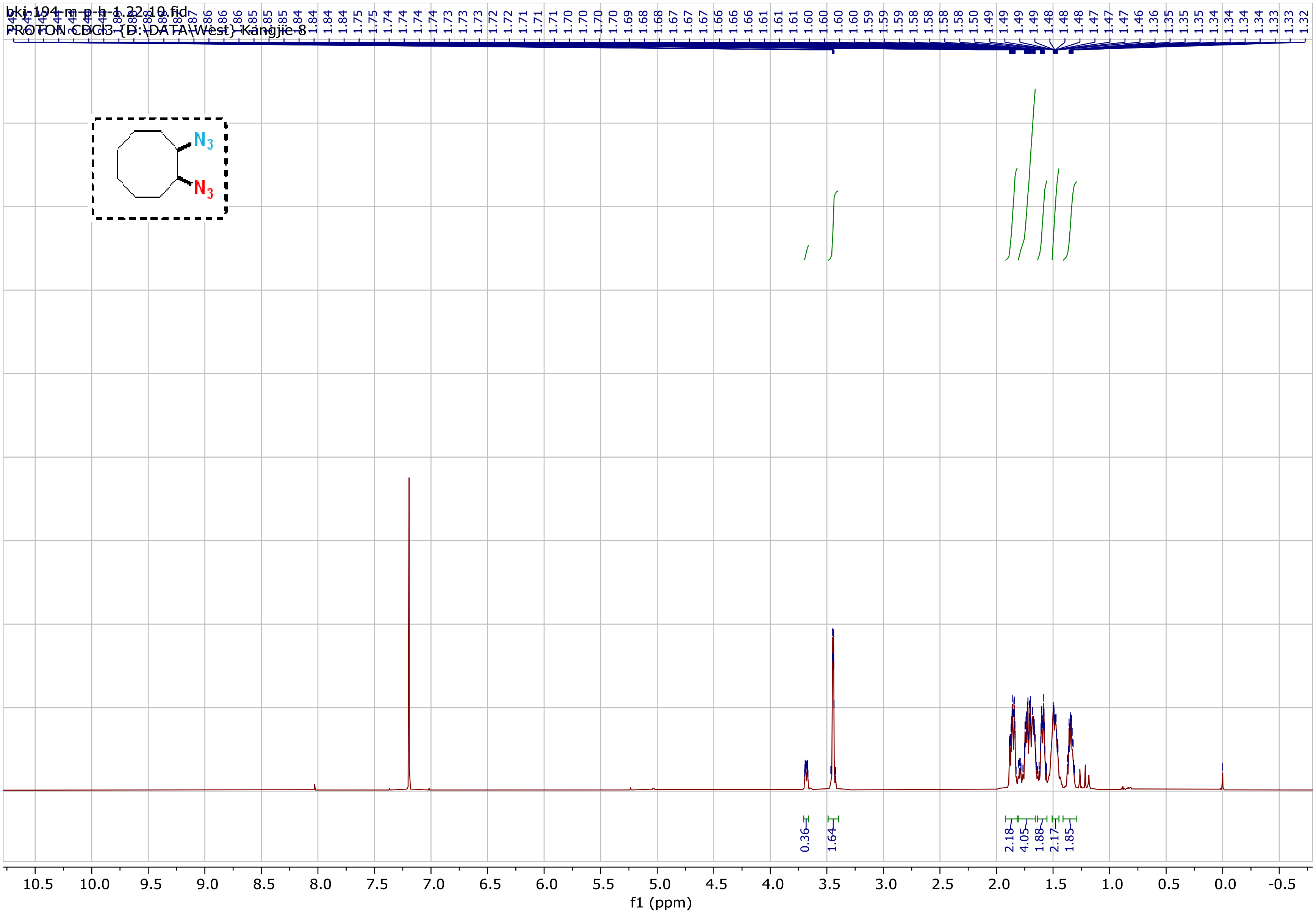

Supplementary Figure 64. ^{1}H NMR Spectrum of **32** (600 MHz, $CDCl_3$)

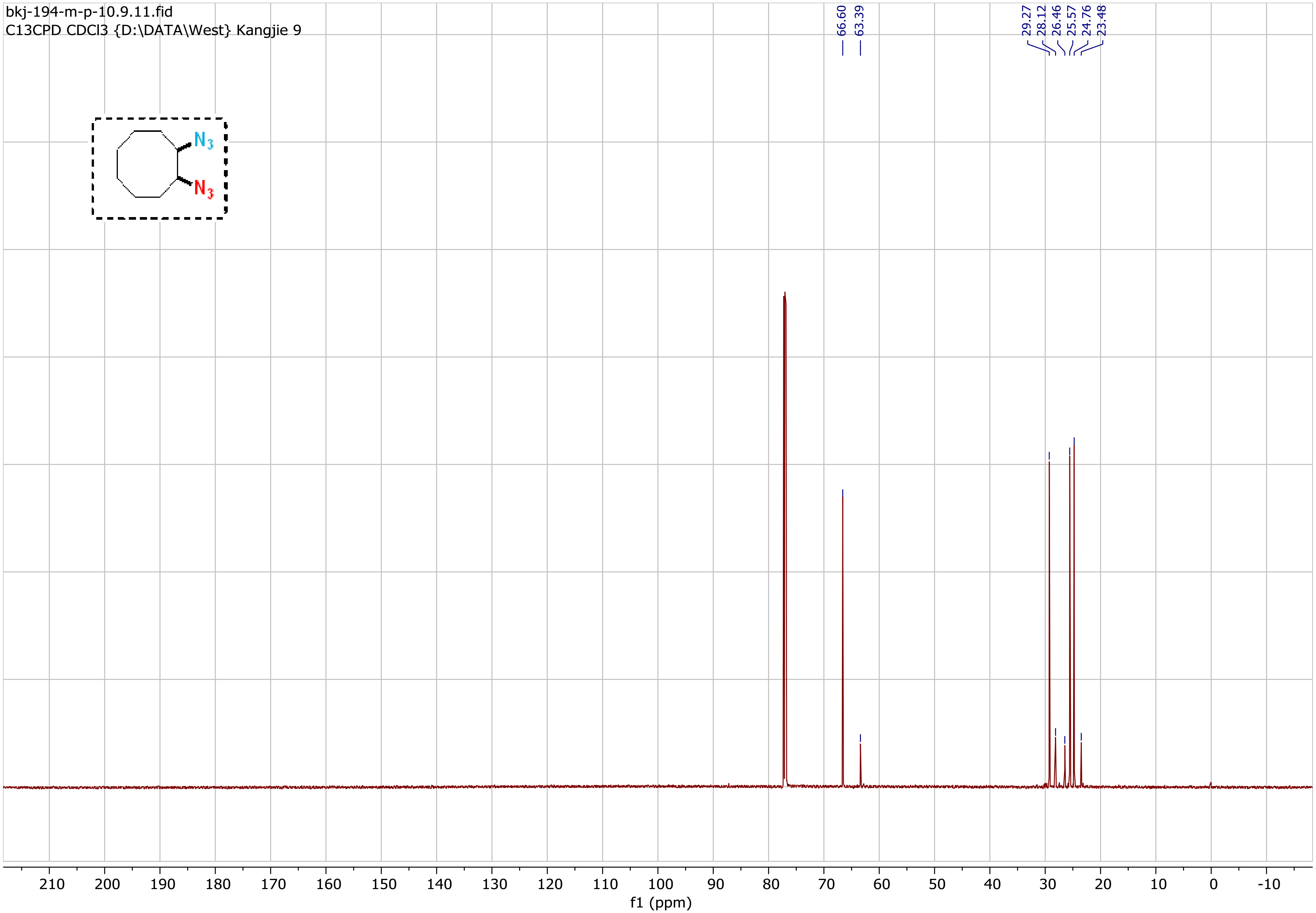

Supplementary Figure 65. ^{13}C NMR Spectrum of **32** (151 MHz, $CDCl_3$)

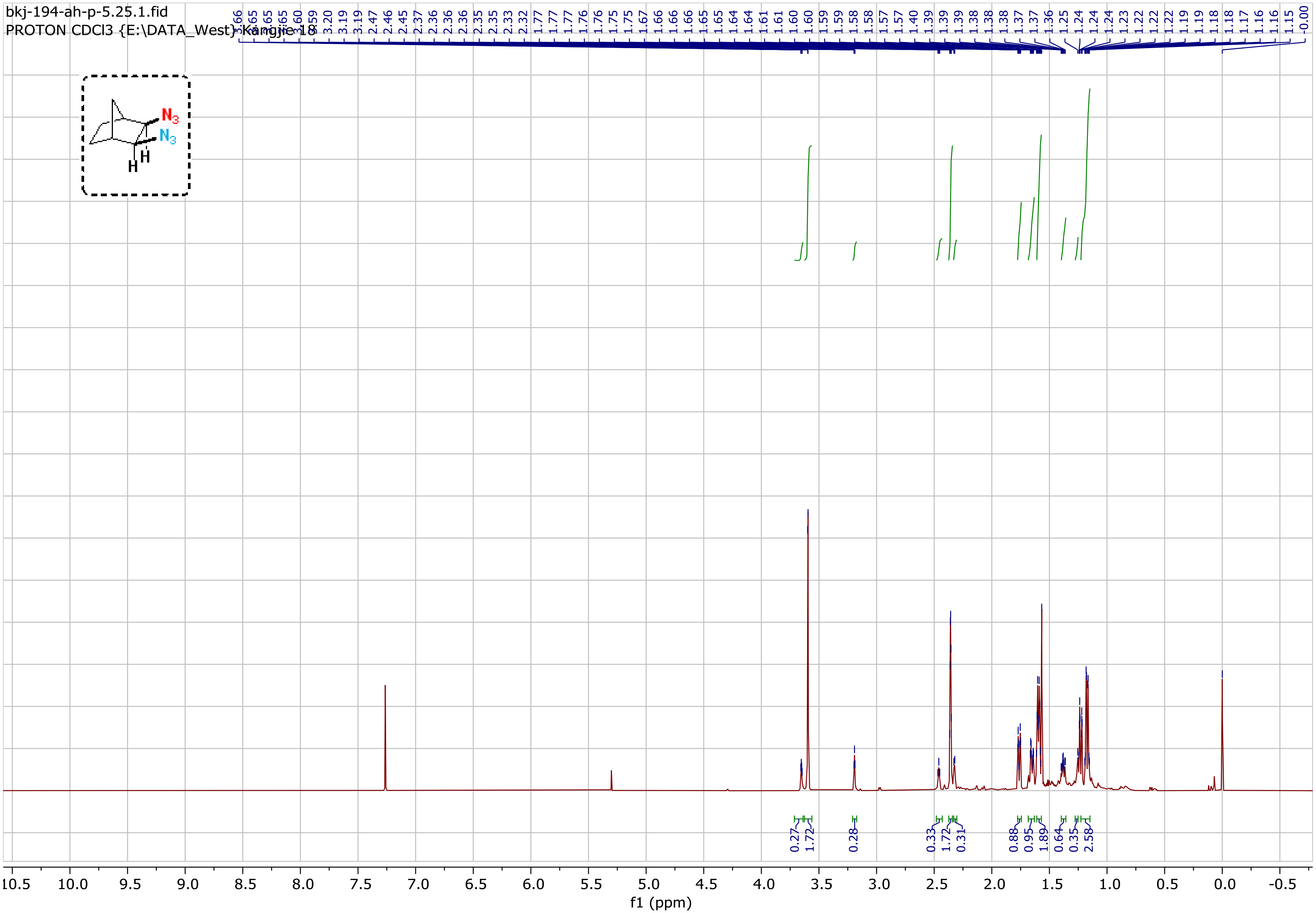

Supplementary Figure 66. ^{1}H NMR Spectrum of **33** (600 MHz, $CDCl_3$)

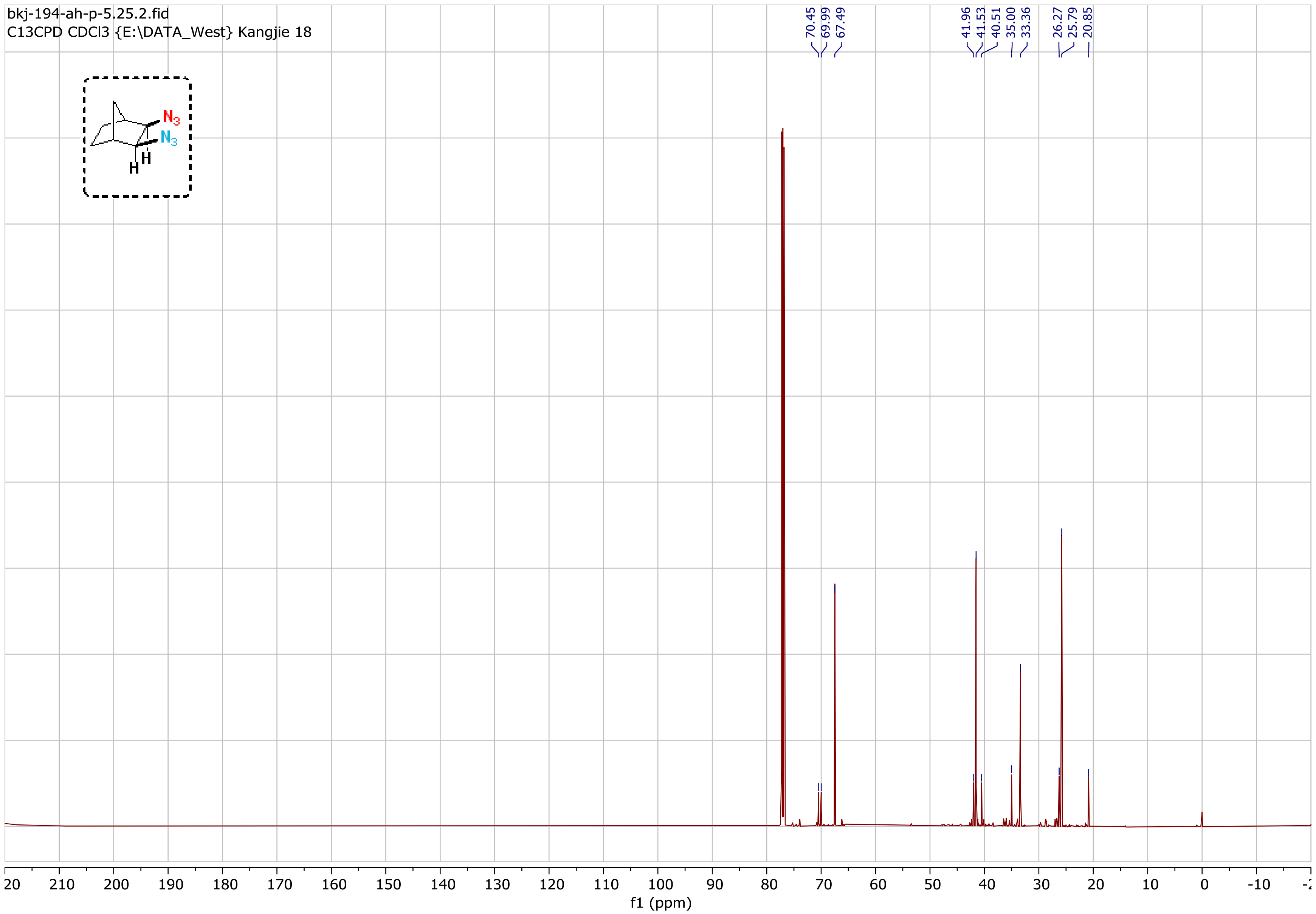

Supplementary Figure 67. ^{13}C NMR Spectrum of **33** (151 MHz, $CDCl_3$)

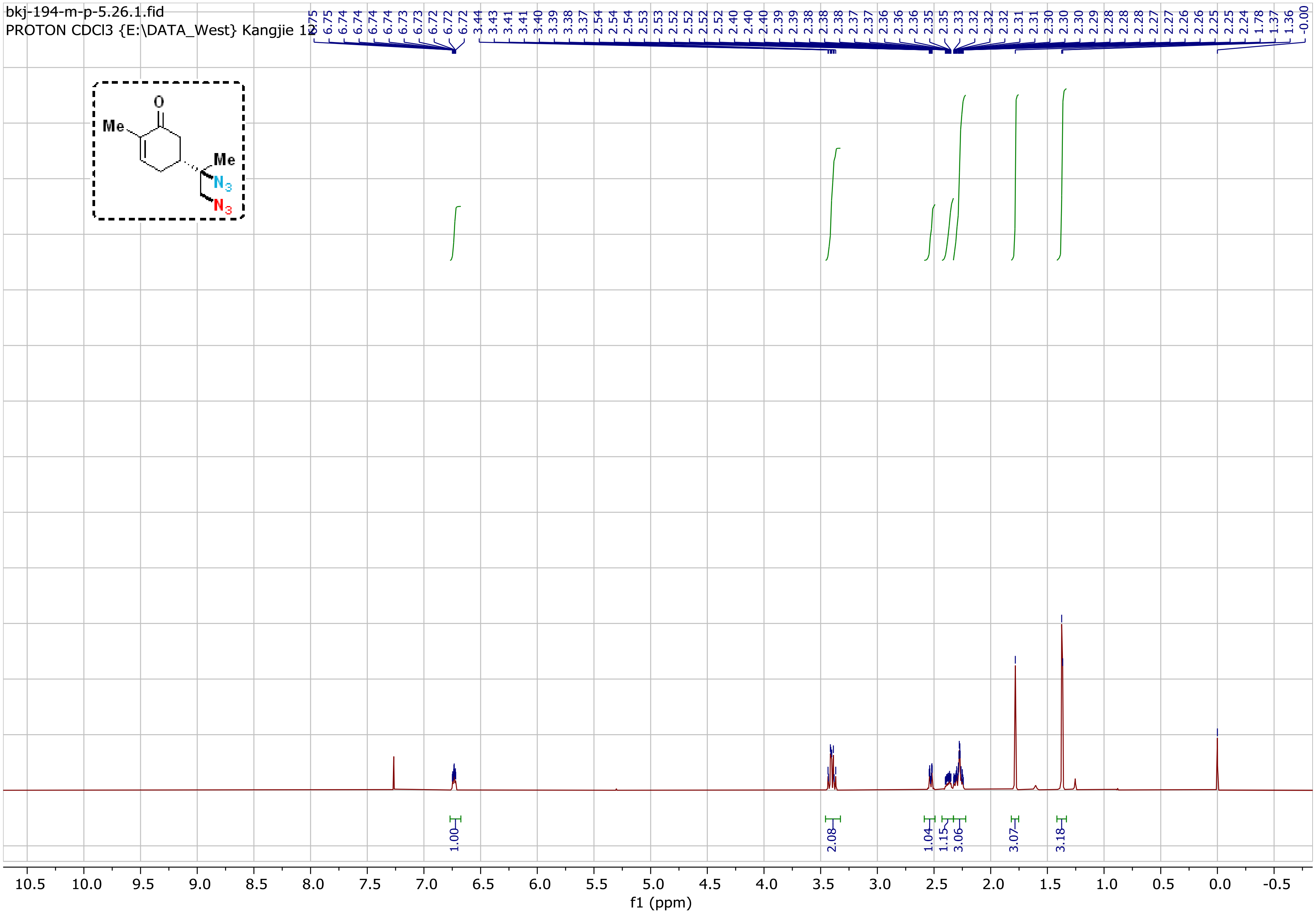

Supplementary Figure 68. ^{1}H NMR Spectrum of **34** (600 MHz, $CDCl_3$)

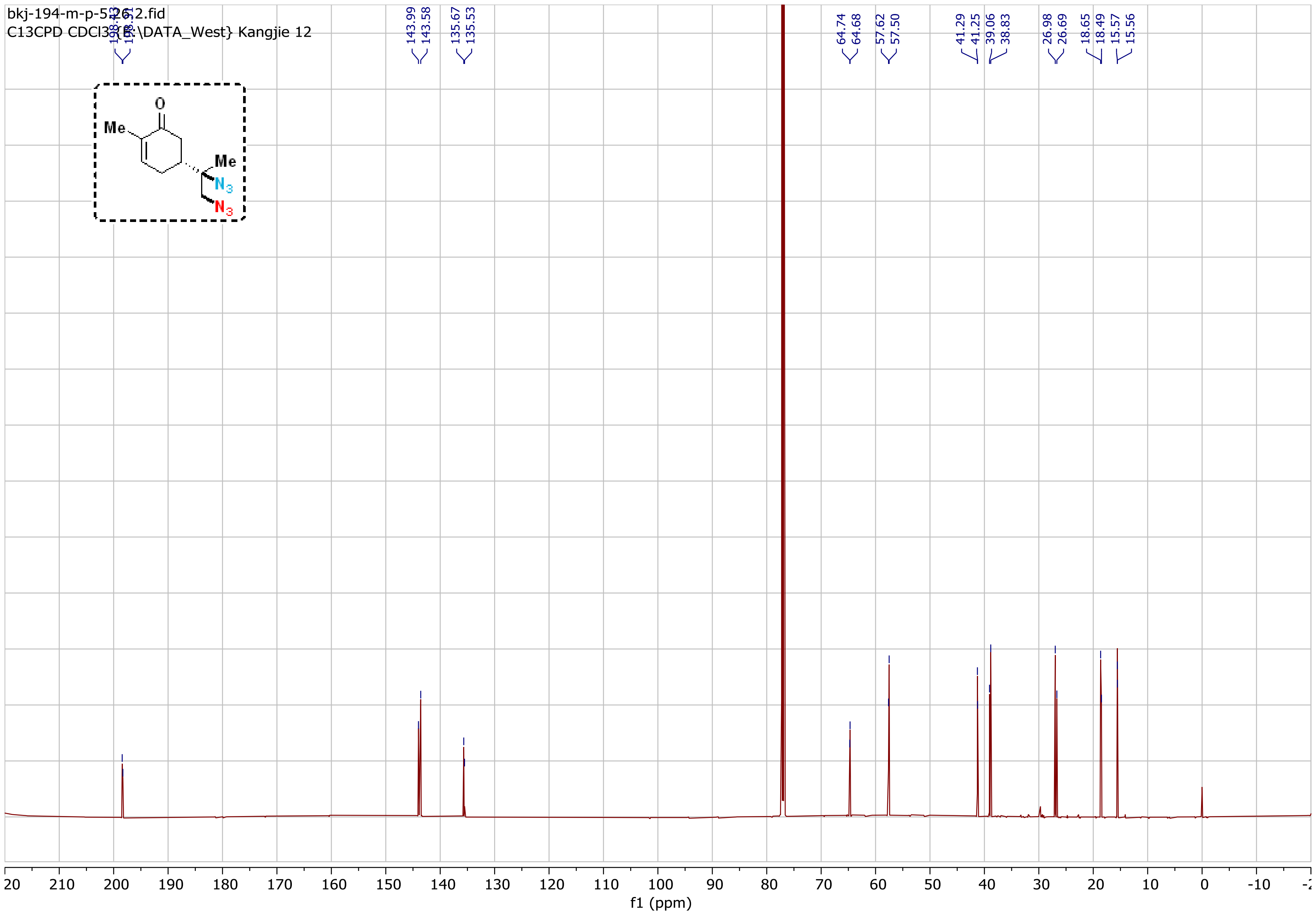

Supplementary Figure 69. ^{13}C NMR Spectrum of **34** (151 MHz, $CDCl_3$)

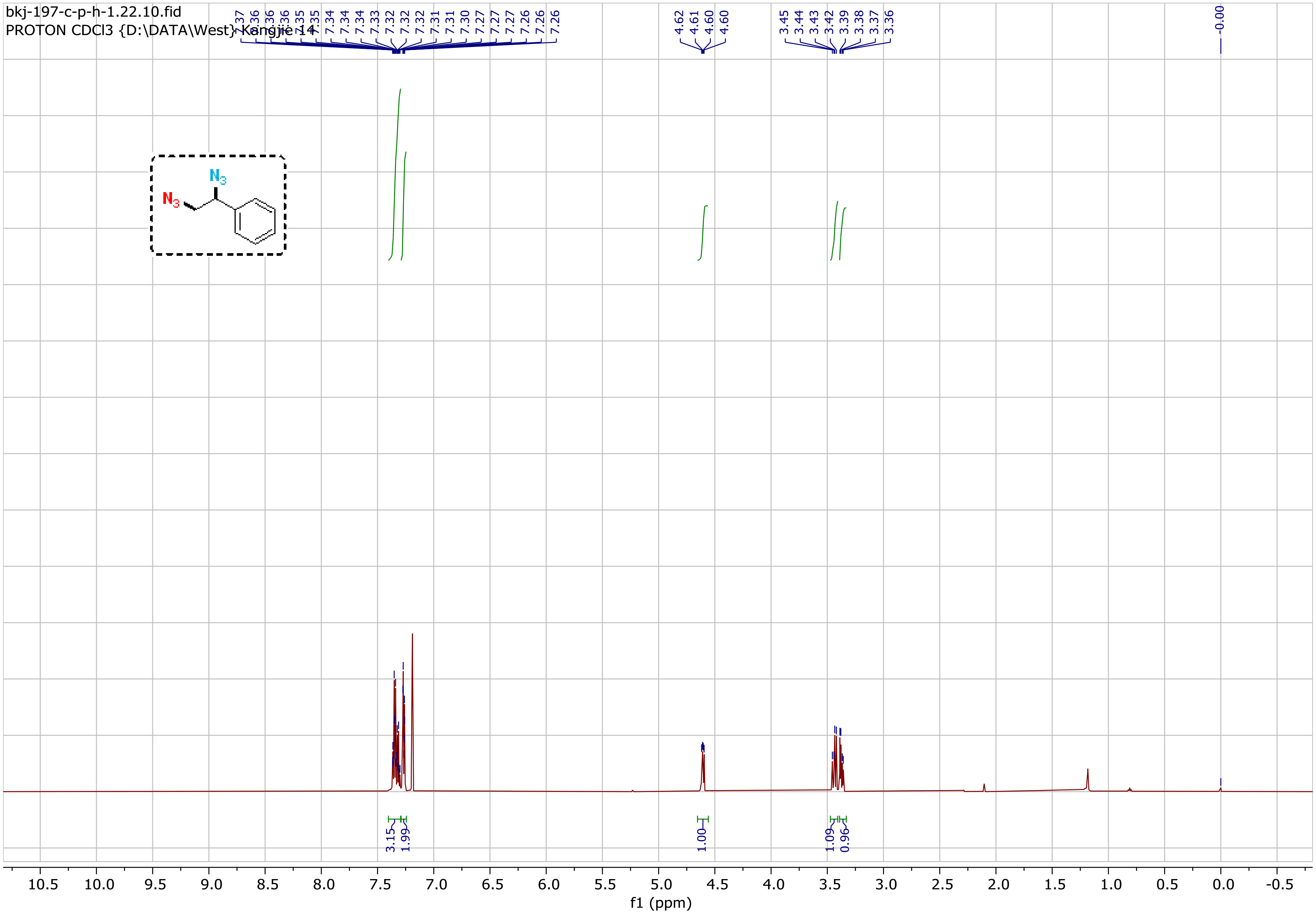

Supplementary Figure 70. ^{1}H NMR Spectrum of **35** (600 MHz, $CDCl_3$)

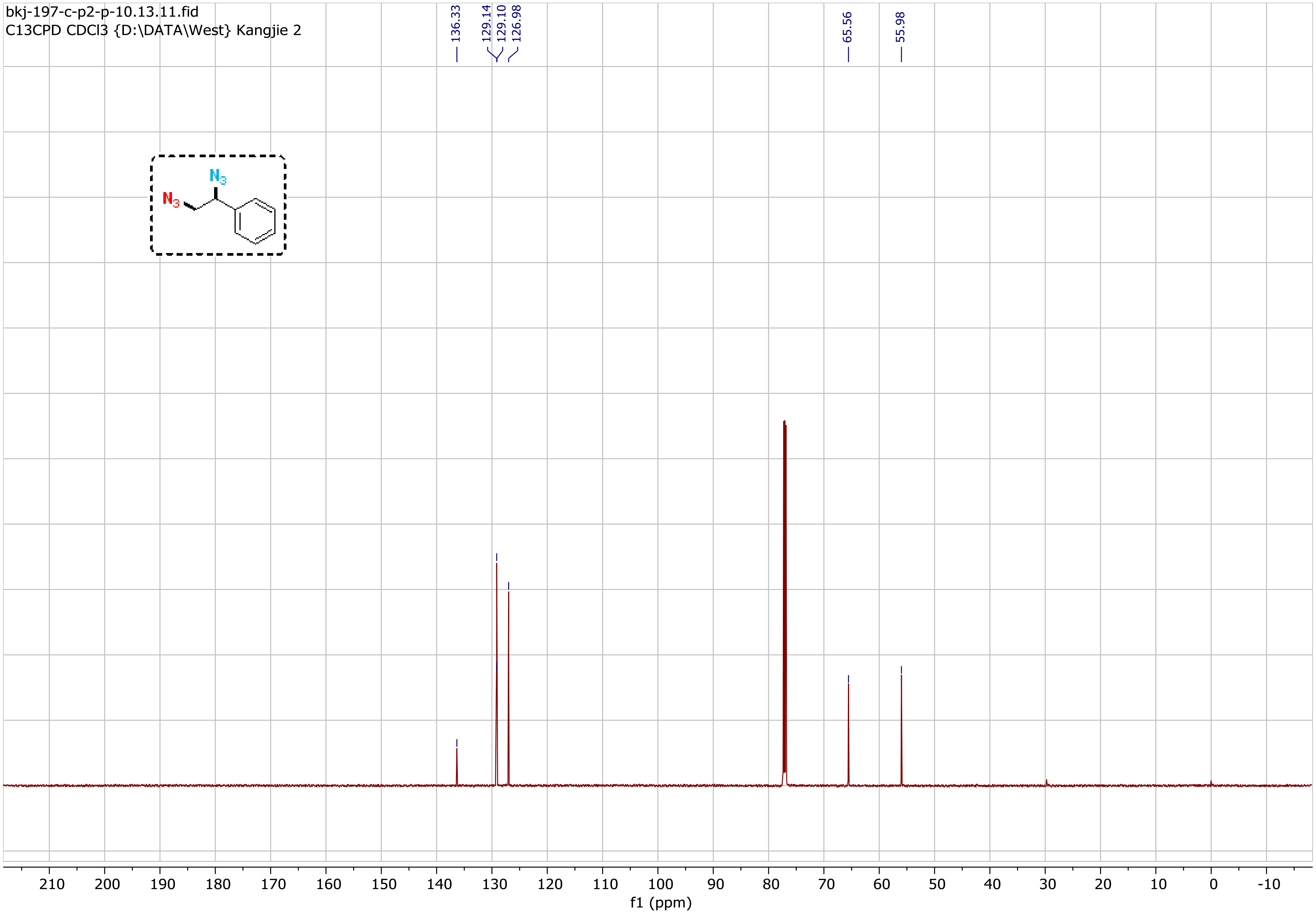

Supplementary Figure 71. ^{13}C NMR Spectrum of **35** (151 MHz, $CDCl_3$)

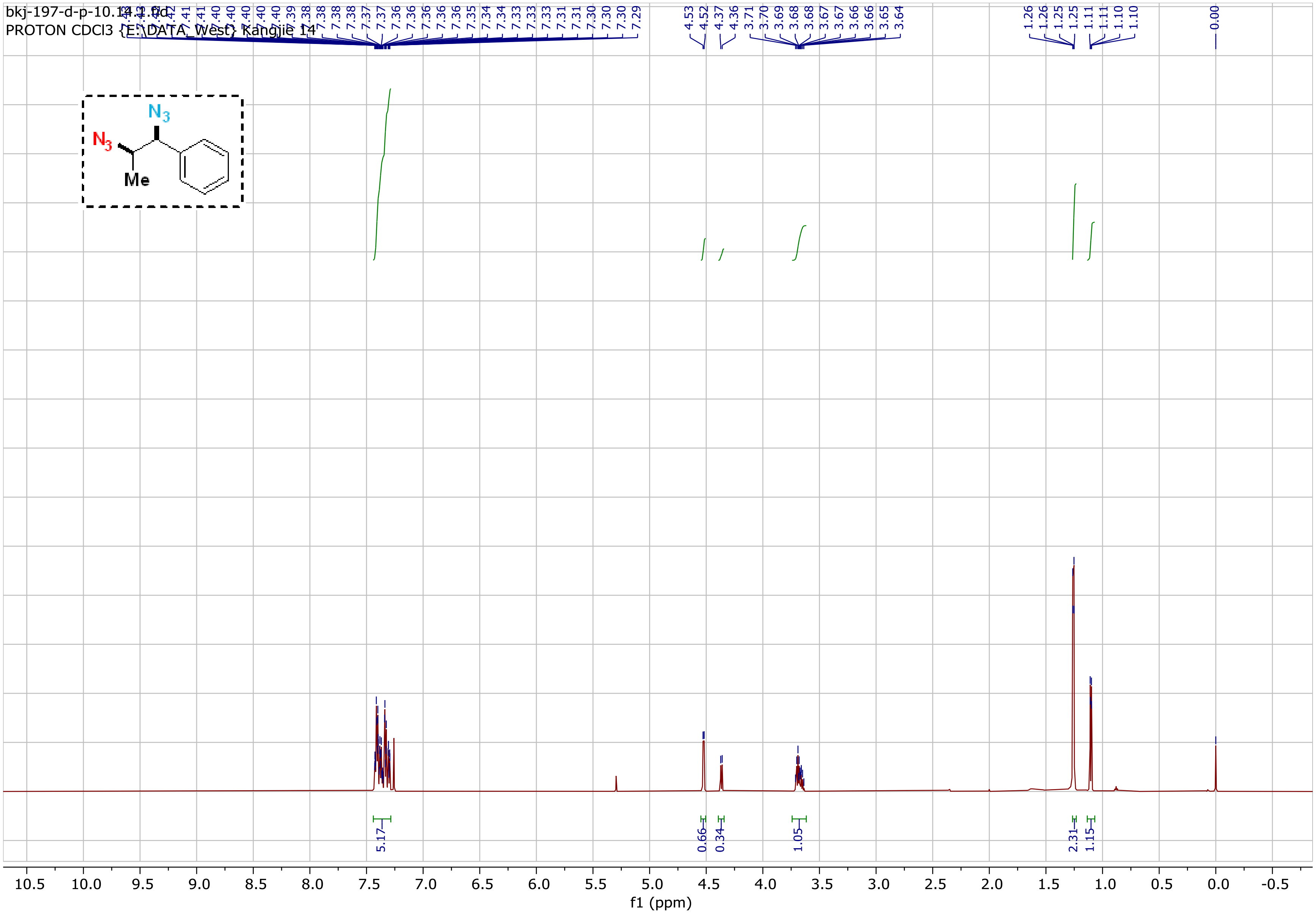

Supplementary Figure 72. ^{1}H NMR Spectrum of **36** (600 MHz, $CDCl_3$)

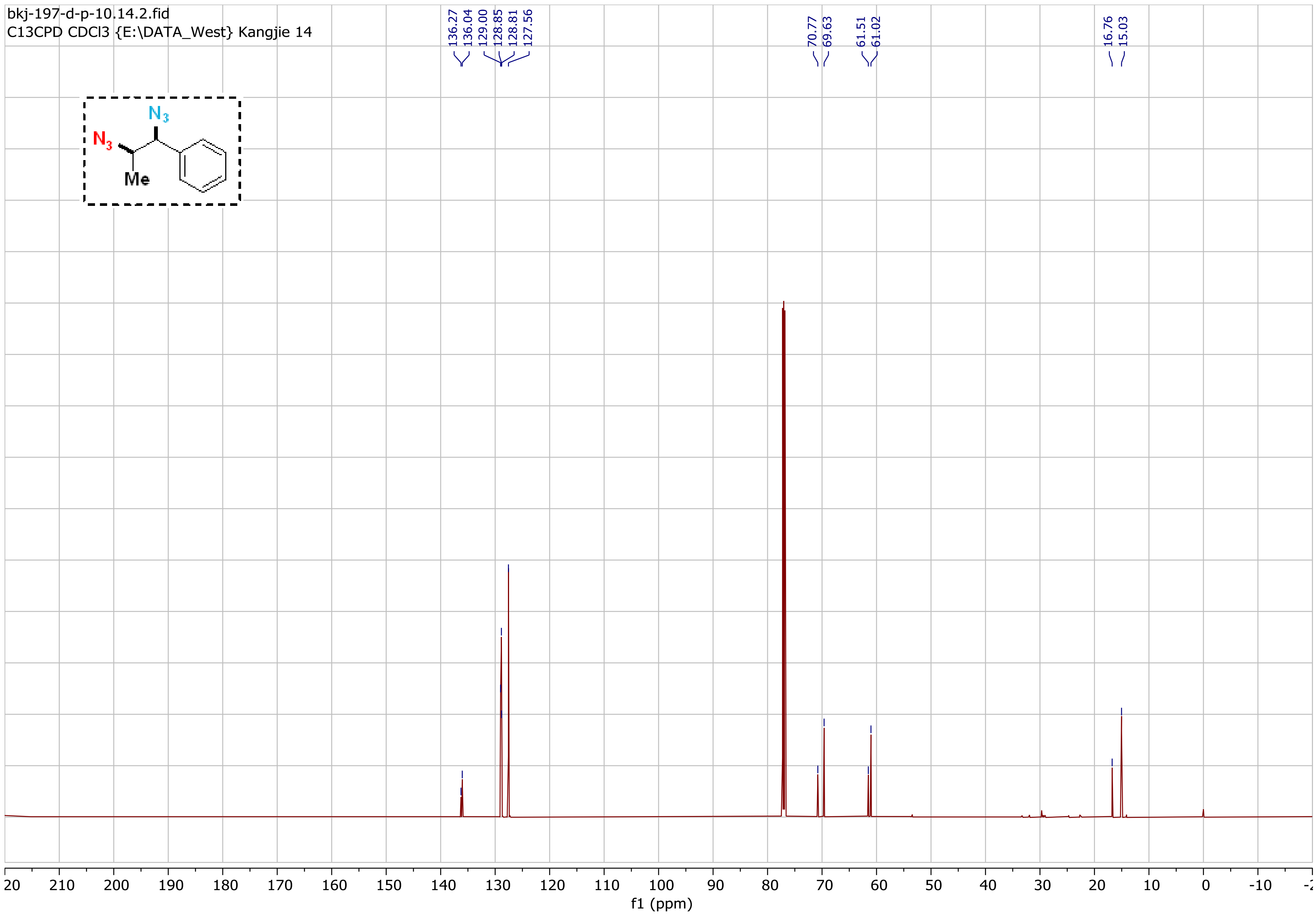

Supplementary Figure 73. ^{13}C NMR Spectrum of **36** (151 MHz, $CDCl_3$)

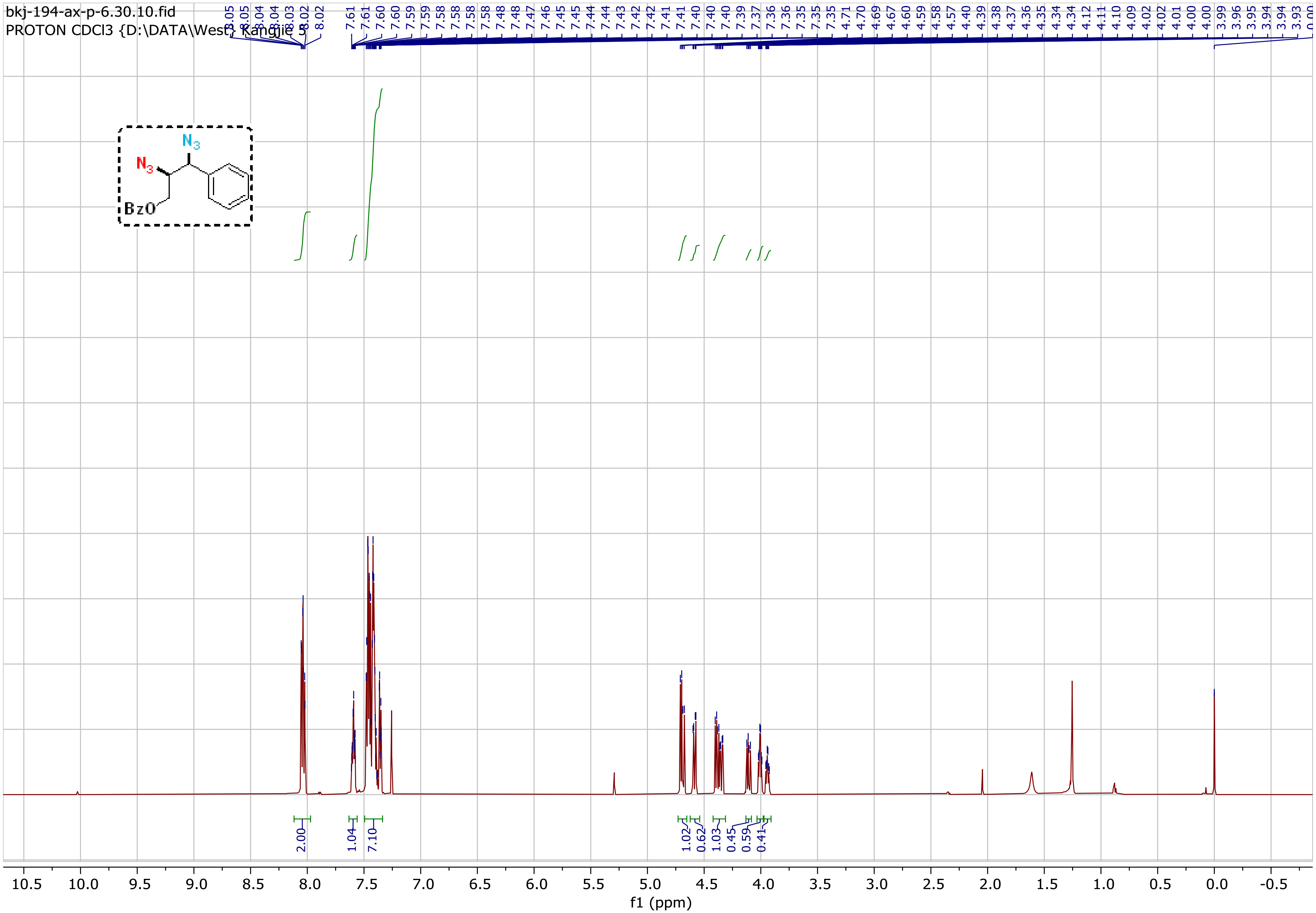

Supplementary Figure 74. ^{1}H NMR Spectrum of **37** (600 MHz, $CDCl_3$)

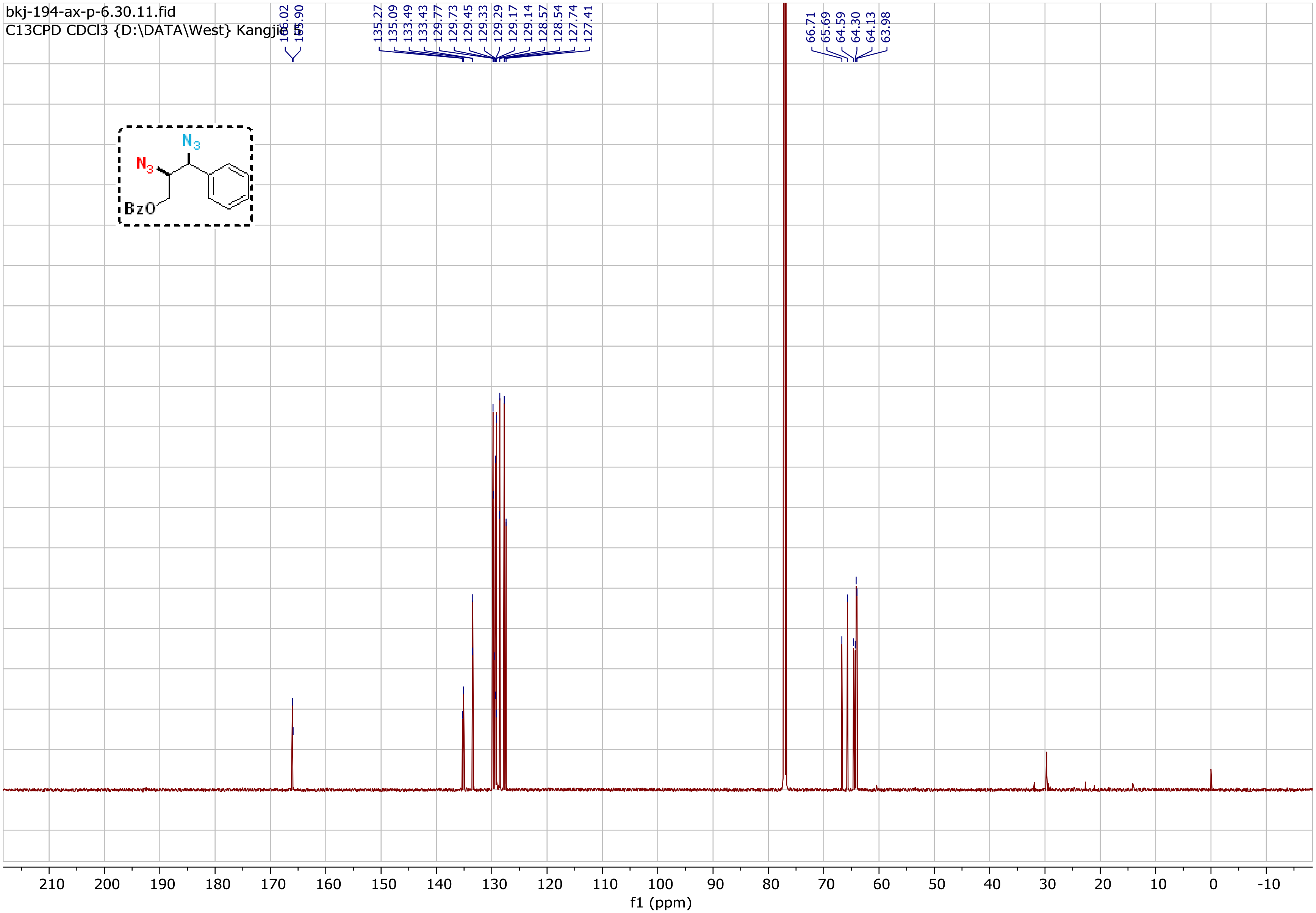

Supplementary Figure 75. ^{13}C NMR Spectrum of **37** (151 MHz, $CDCl_3$)

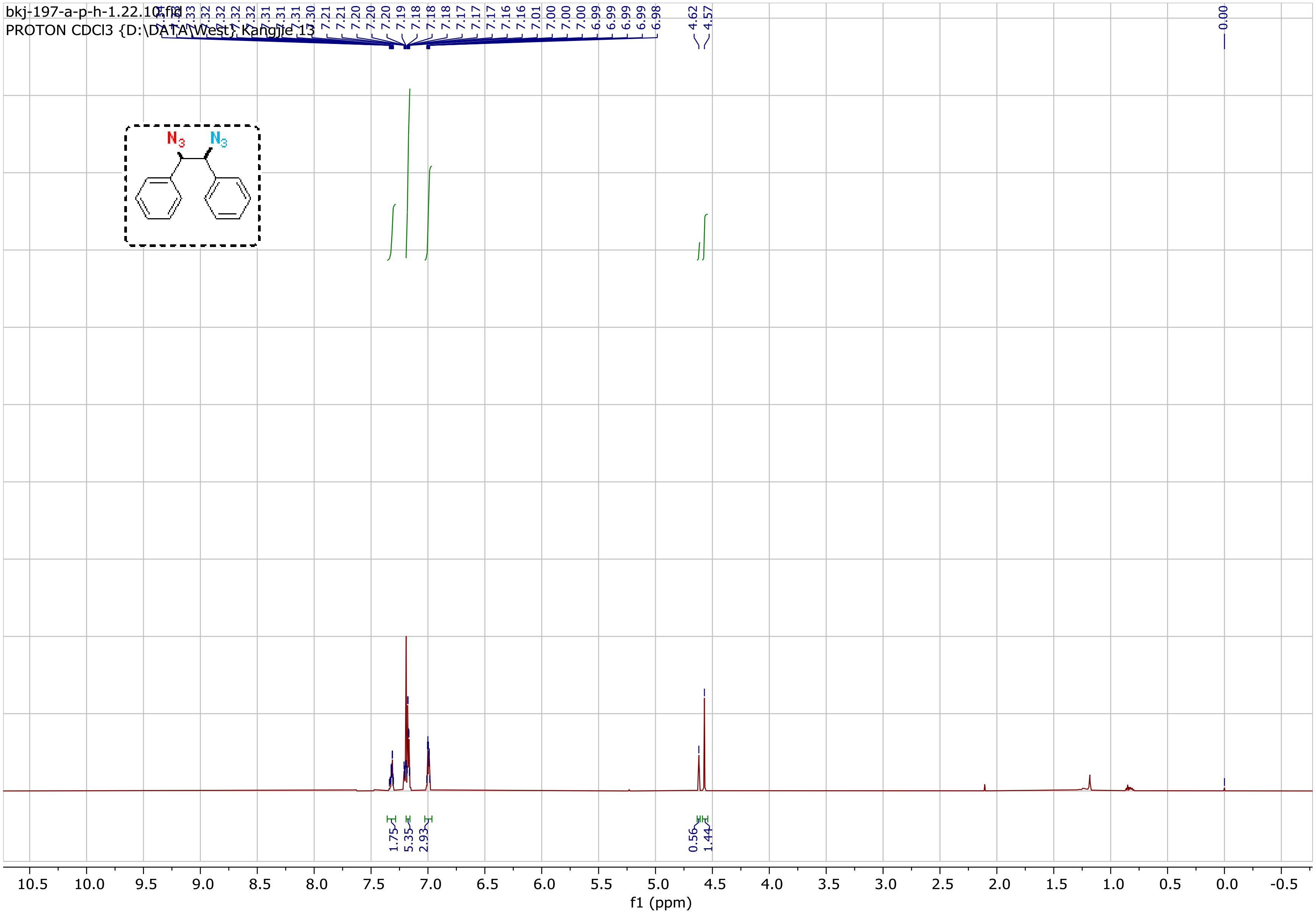

Supplementary Figure 76. ^{1}H NMR Spectrum of **38** (600 MHz, $CDCl_3$)

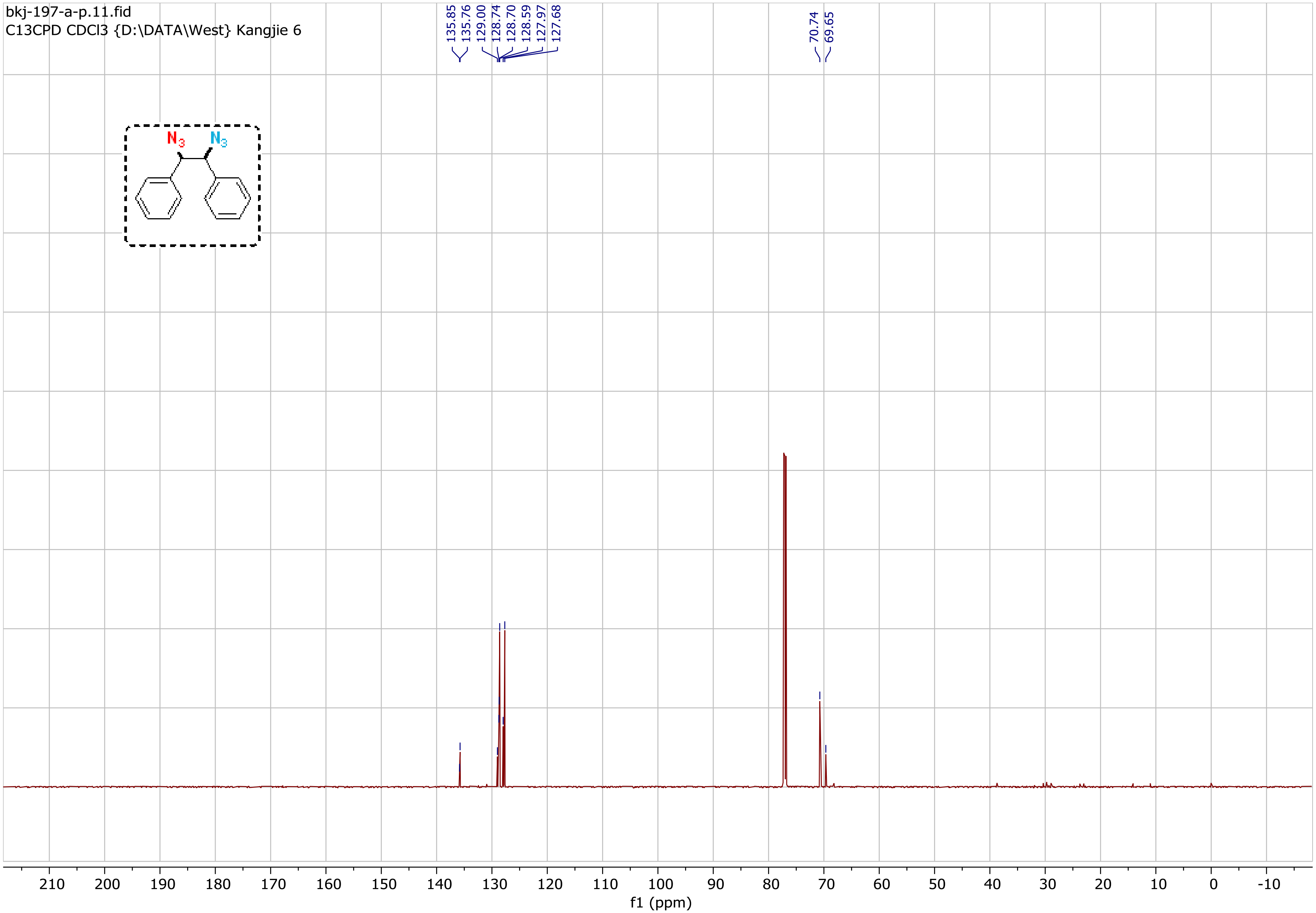

Supplementary Figure 77. ^{13}C NMR Spectrum of **38** (151 MHz, $CDCl_3$)

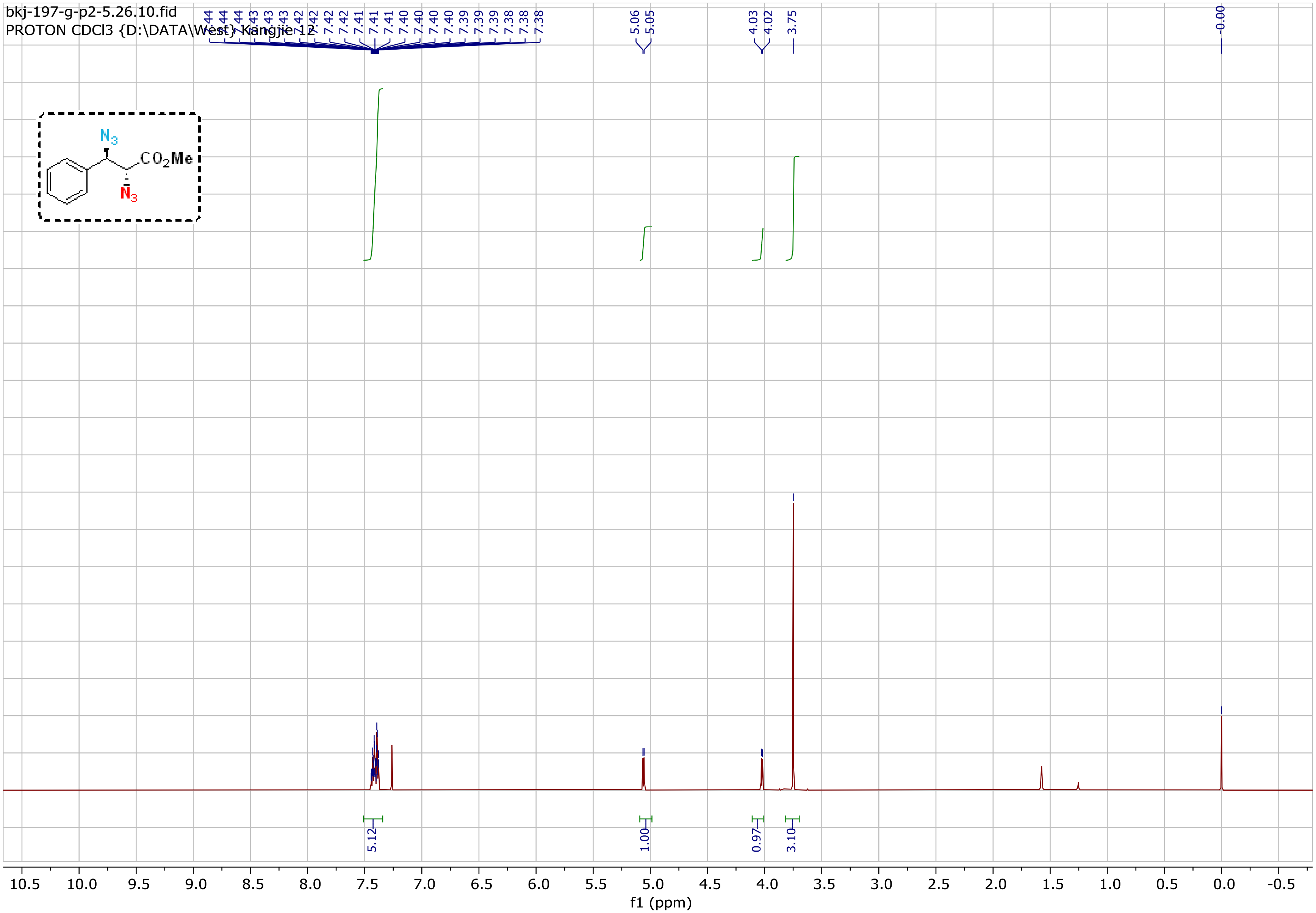

Supplementary Figure 78. ^{1}H NMR Spectrum of **39** (trans-) (600 MHz, $CDCl_3$)

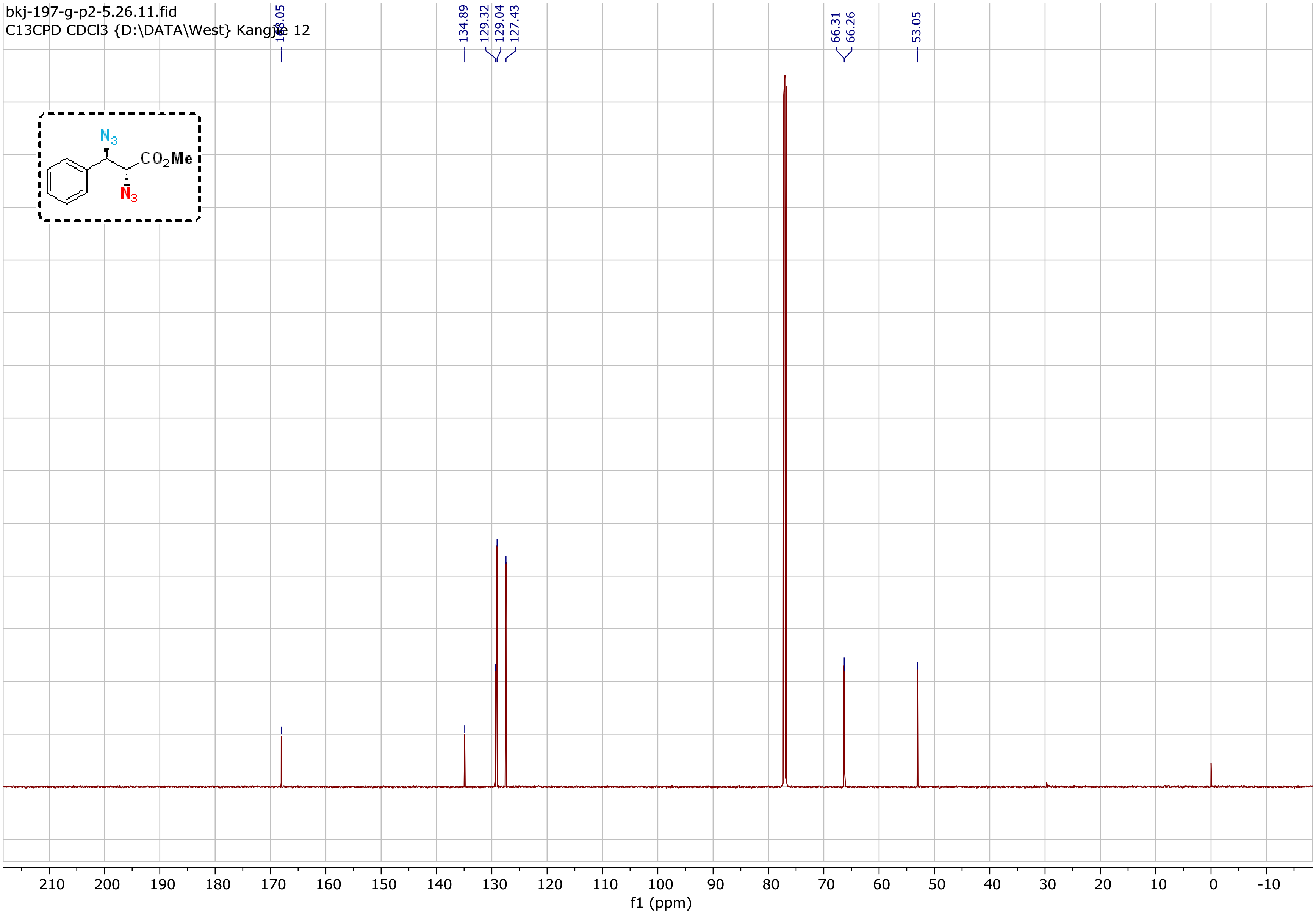

Supplementary Figure 79. ^{13}C NMR Spectrum of **39** (trans-) (151 MHz, $CDCl_3$)

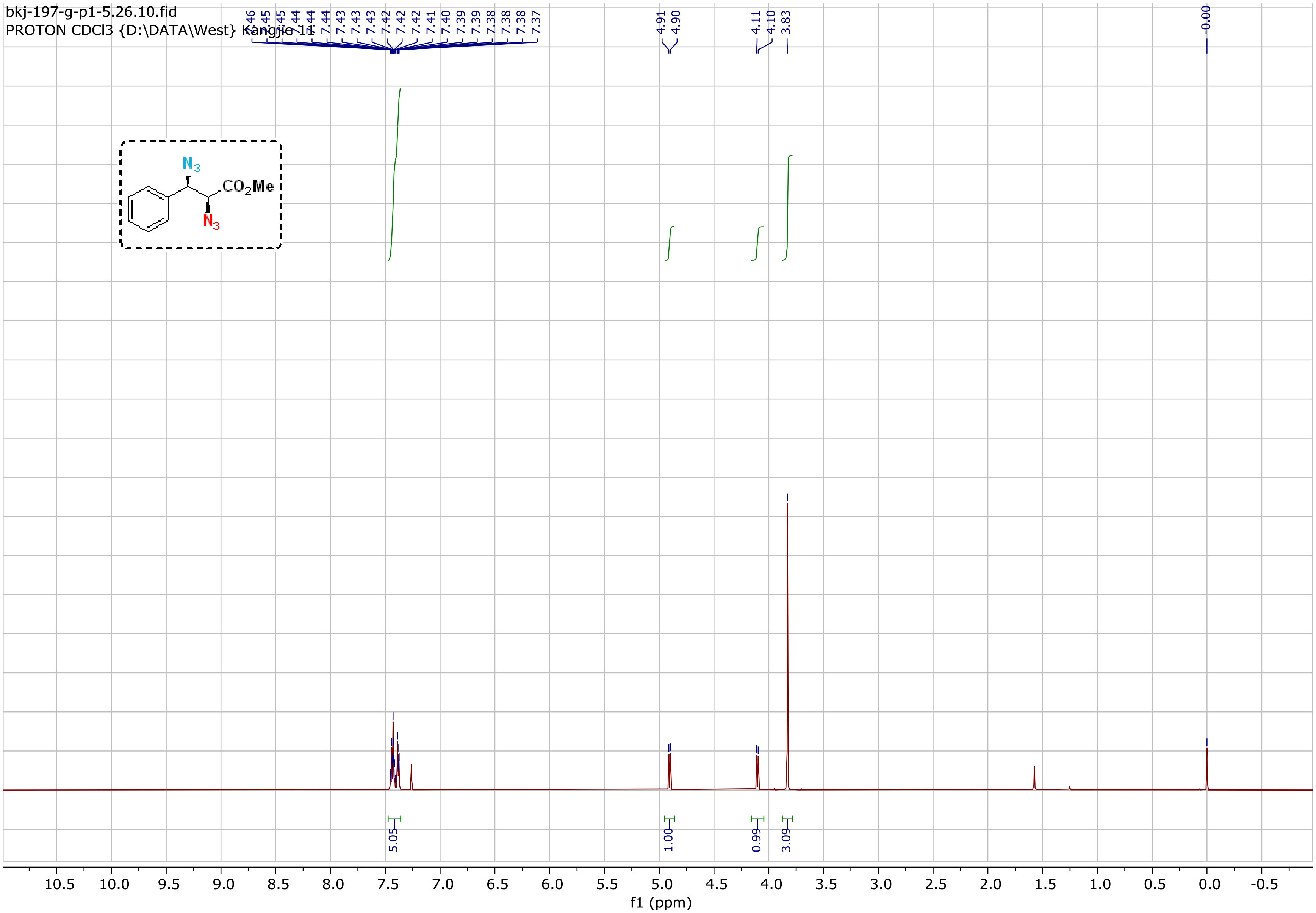

Supplementary Figure 80. ^{1}H NMR Spectrum of **39** (cis-) (600 MHz, $CDCl_3$)

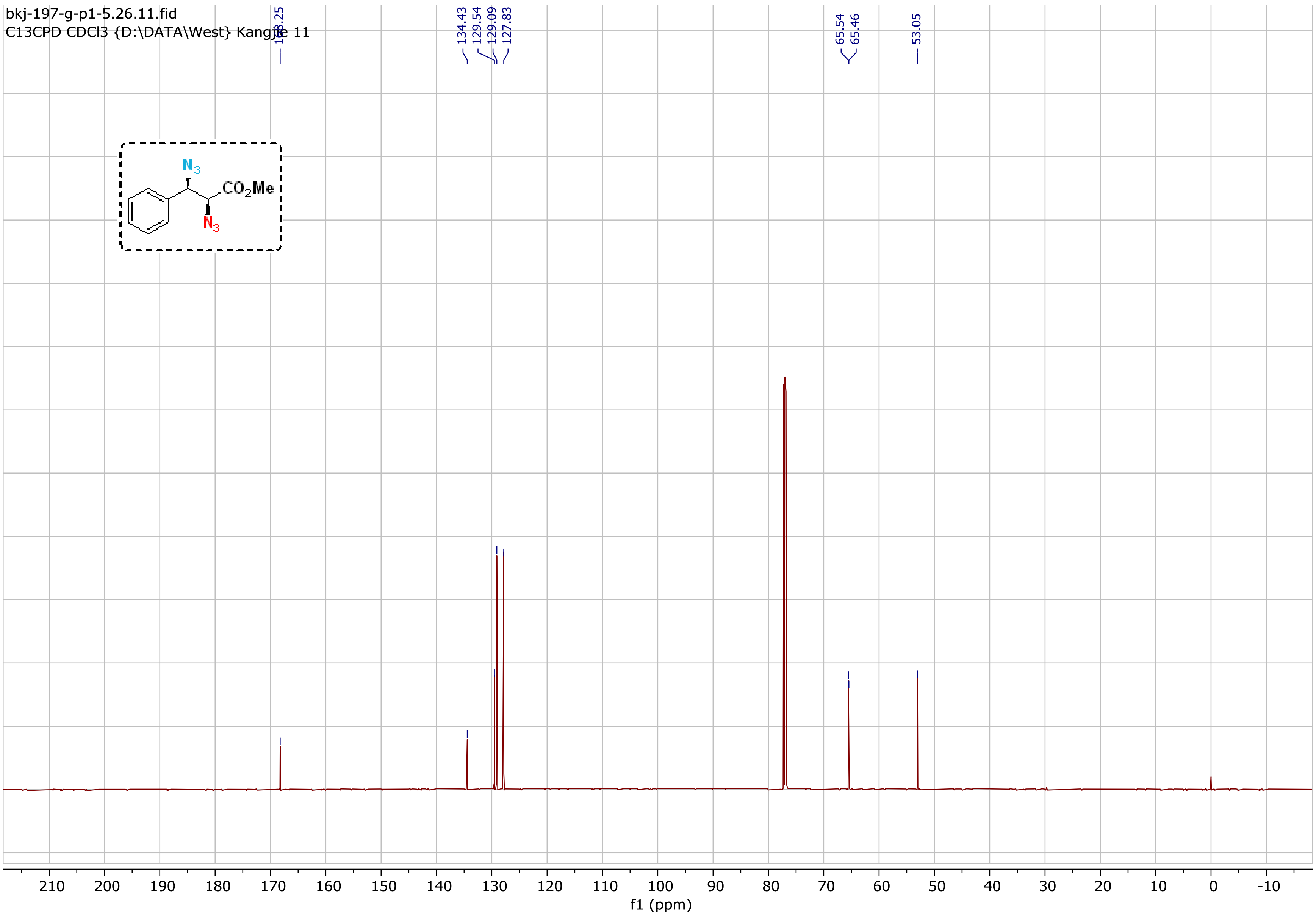

Supplementary Figure 81. ^{13}C NMR Spectrum of **39** (cis-) (151 MHz, $CDCl_3$)

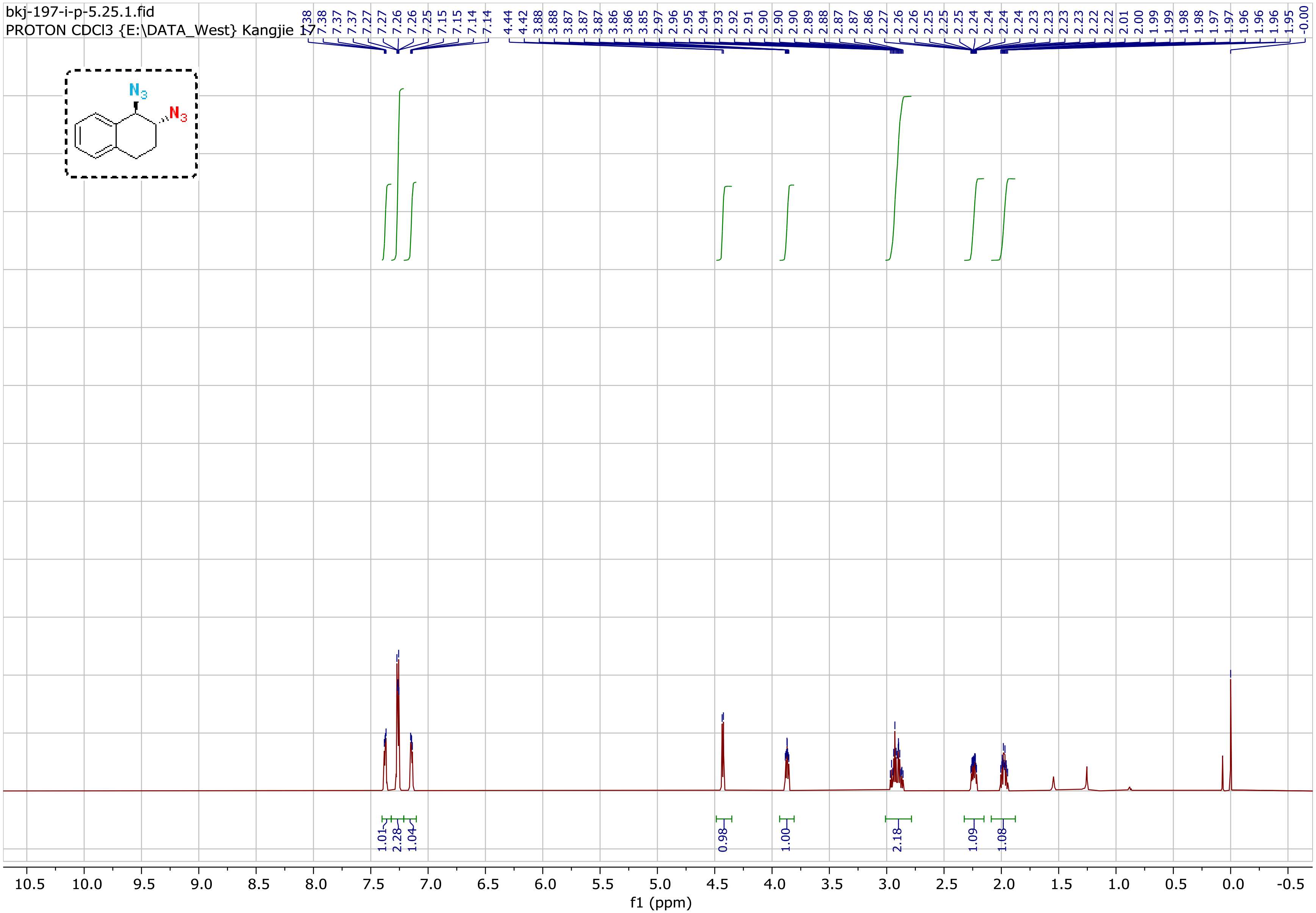

Supplementary Figure 82. ^{1}H NMR Spectrum of **40** (trans-) (600 MHz, $CDCl_3$)

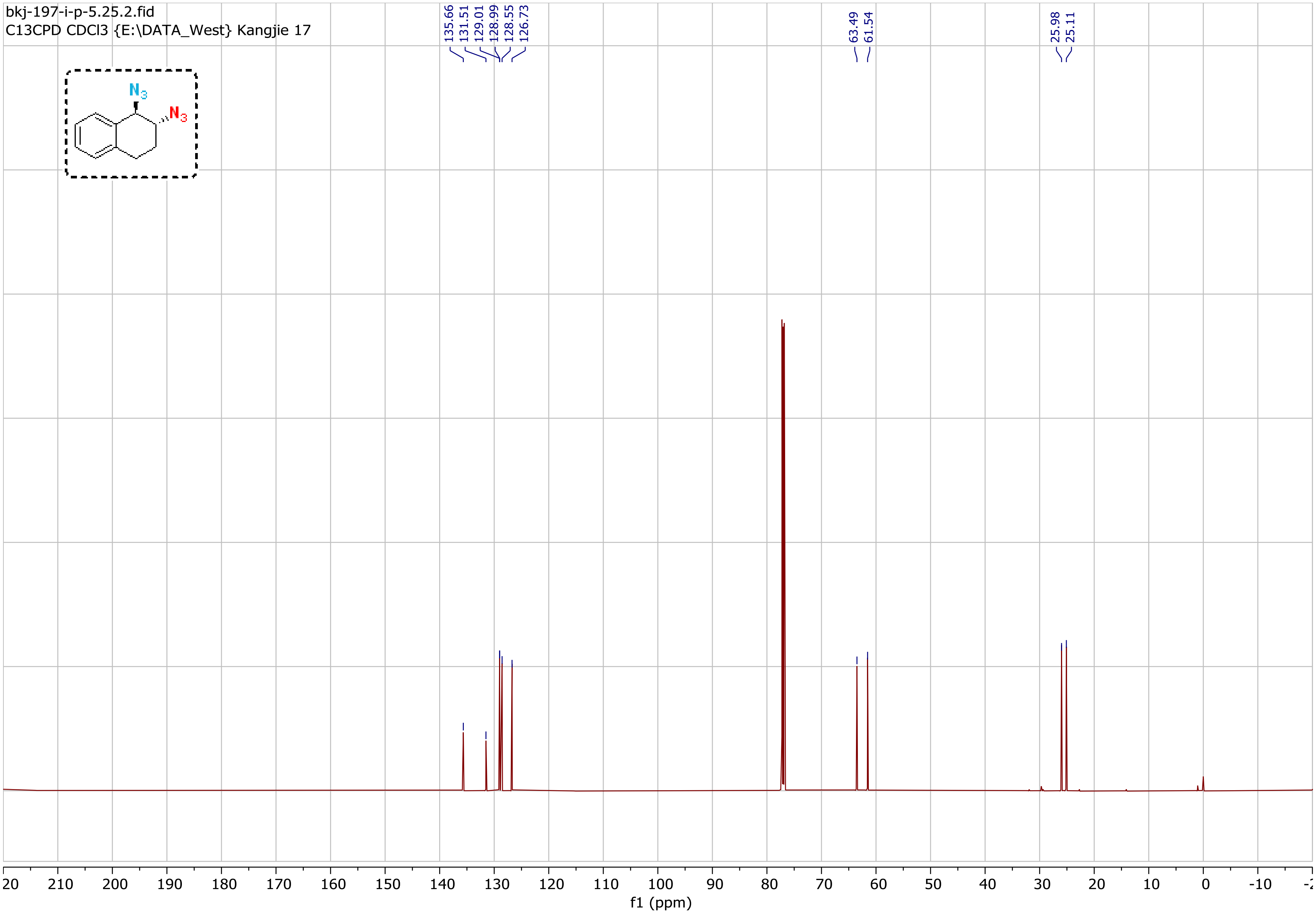

Supplementary Figure 83. ^{13}C NMR Spectrum of **40** (trans-) (151 MHz, $CDCl_3$)

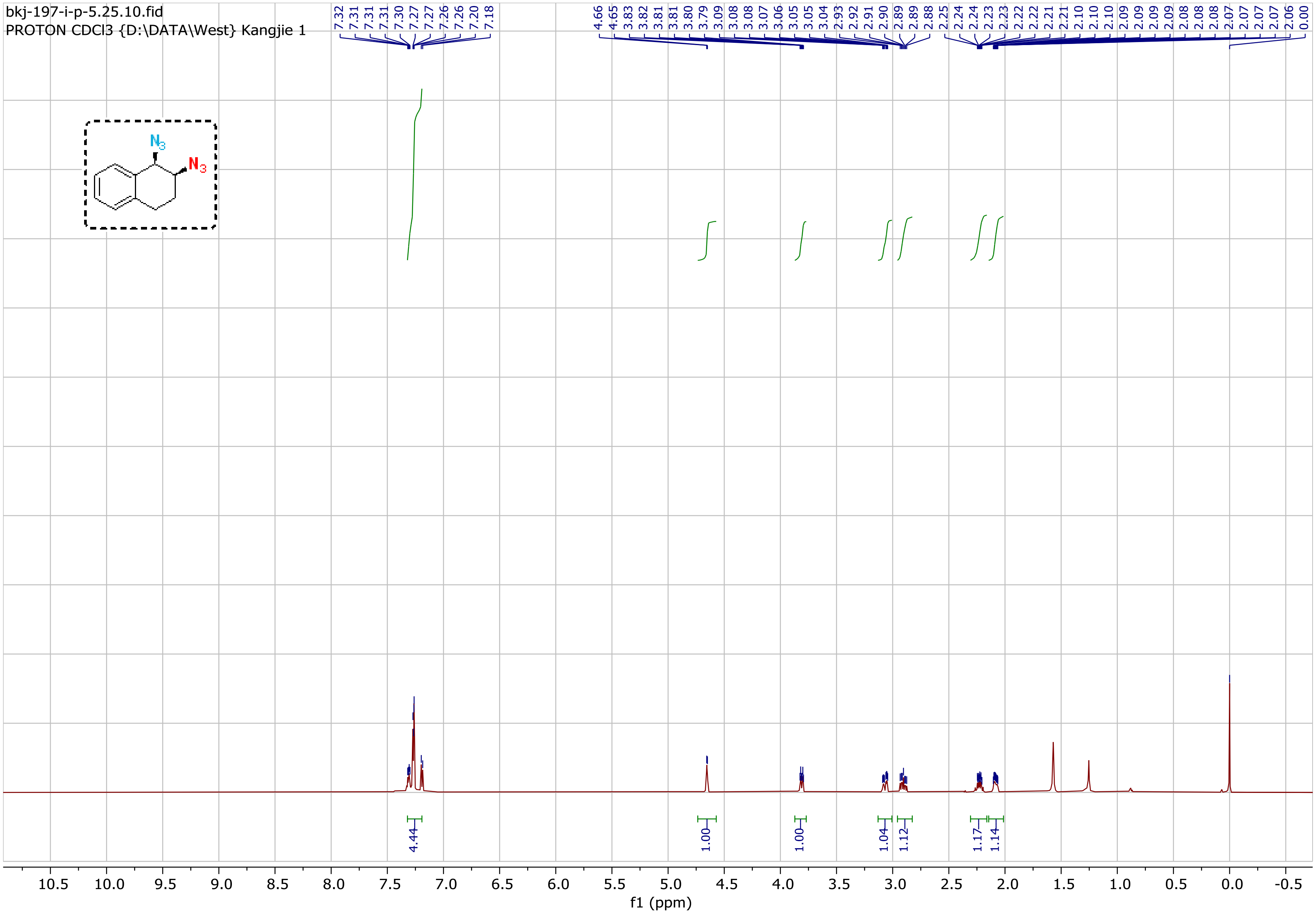

Supplementary Figure 84. ^{1}H NMR Spectrum of **40** (cis-) (600 MHz, $CDCl_3$)

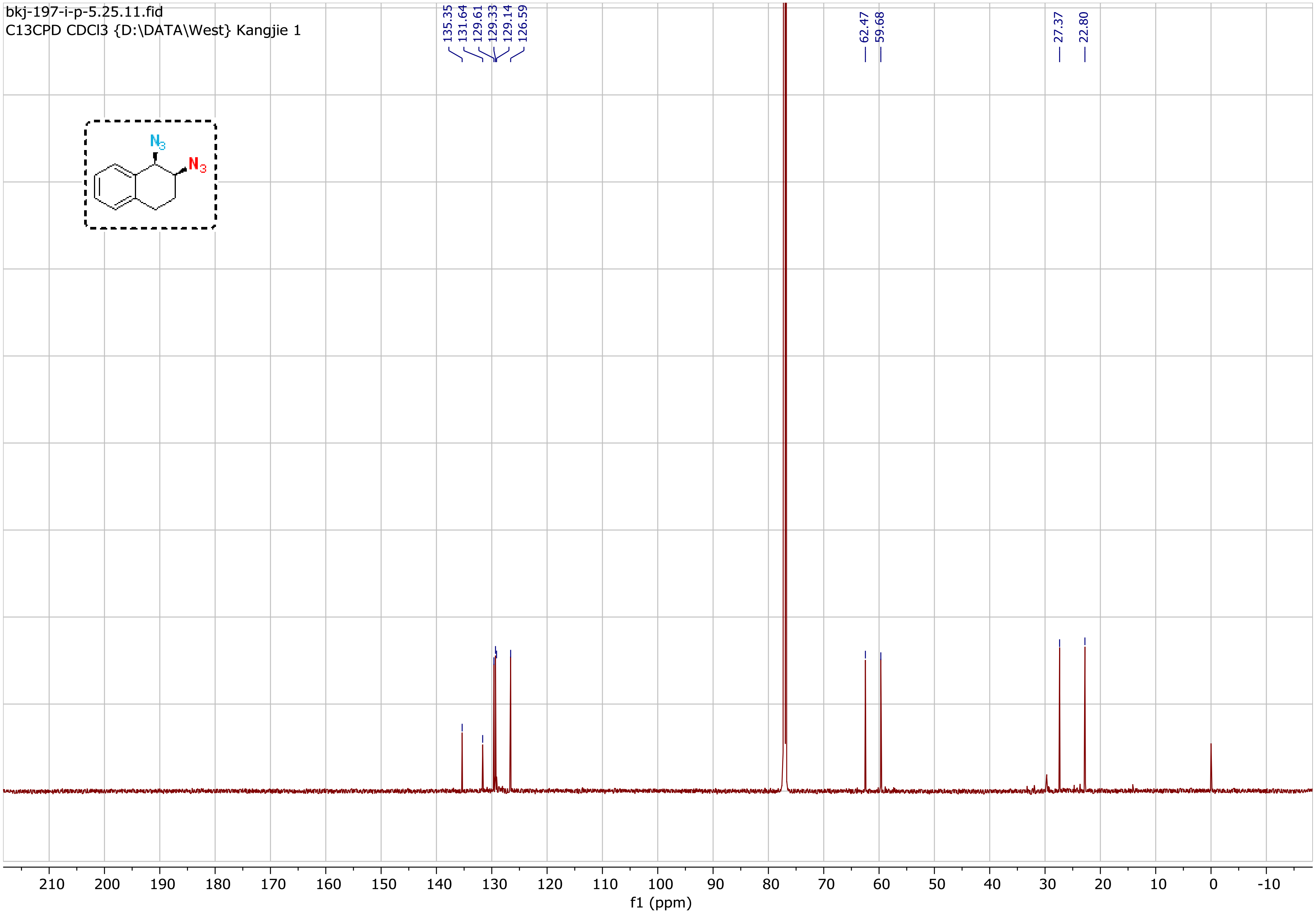

Supplementary Figure 85. ^{13}C NMR Spectrum of **40** (cis-) (151 MHz, $CDCl_3$)

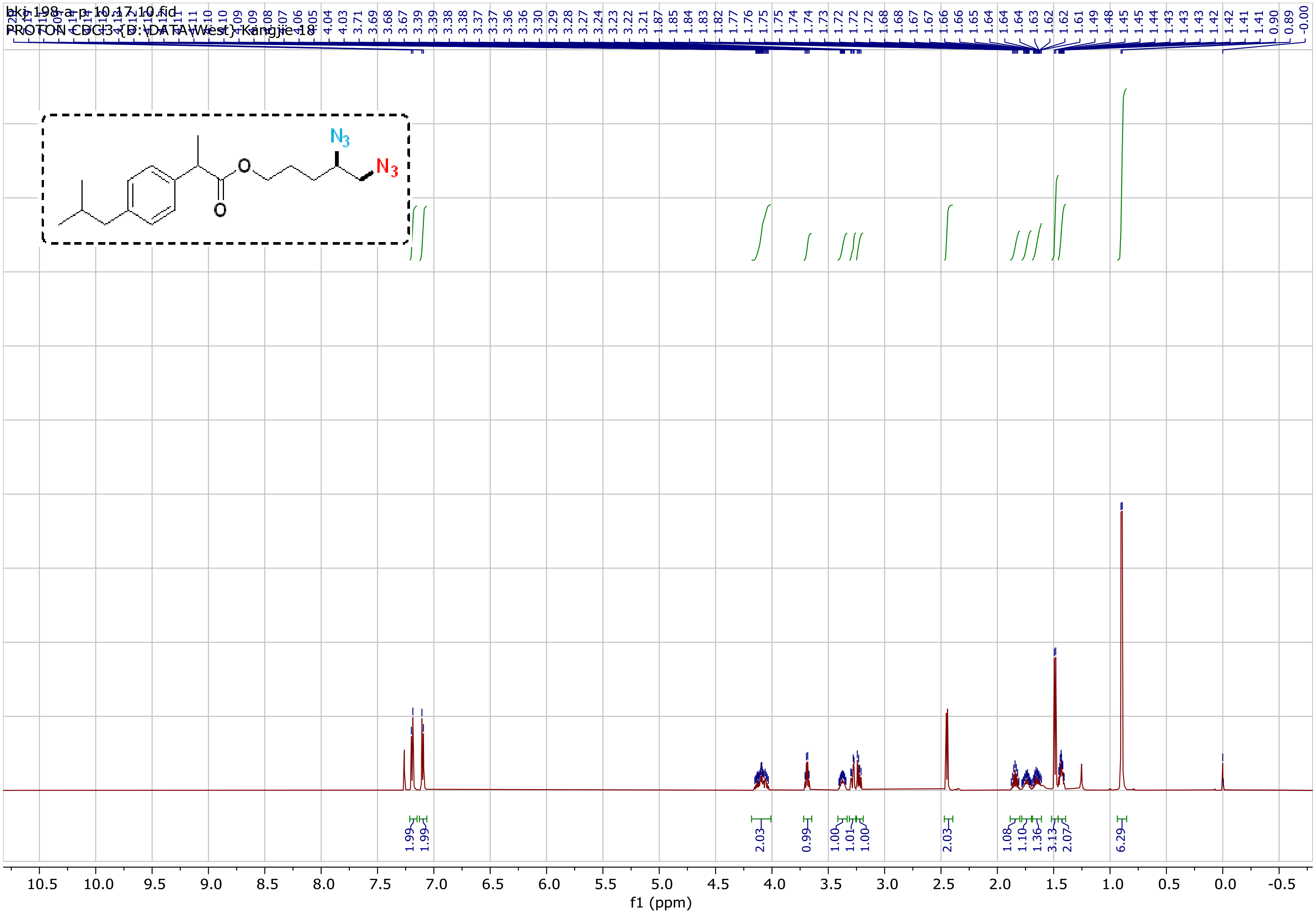

Supplementary Figure 86. ^{1}H NMR Spectrum of **41** (600 MHz, $CDCl_3$)

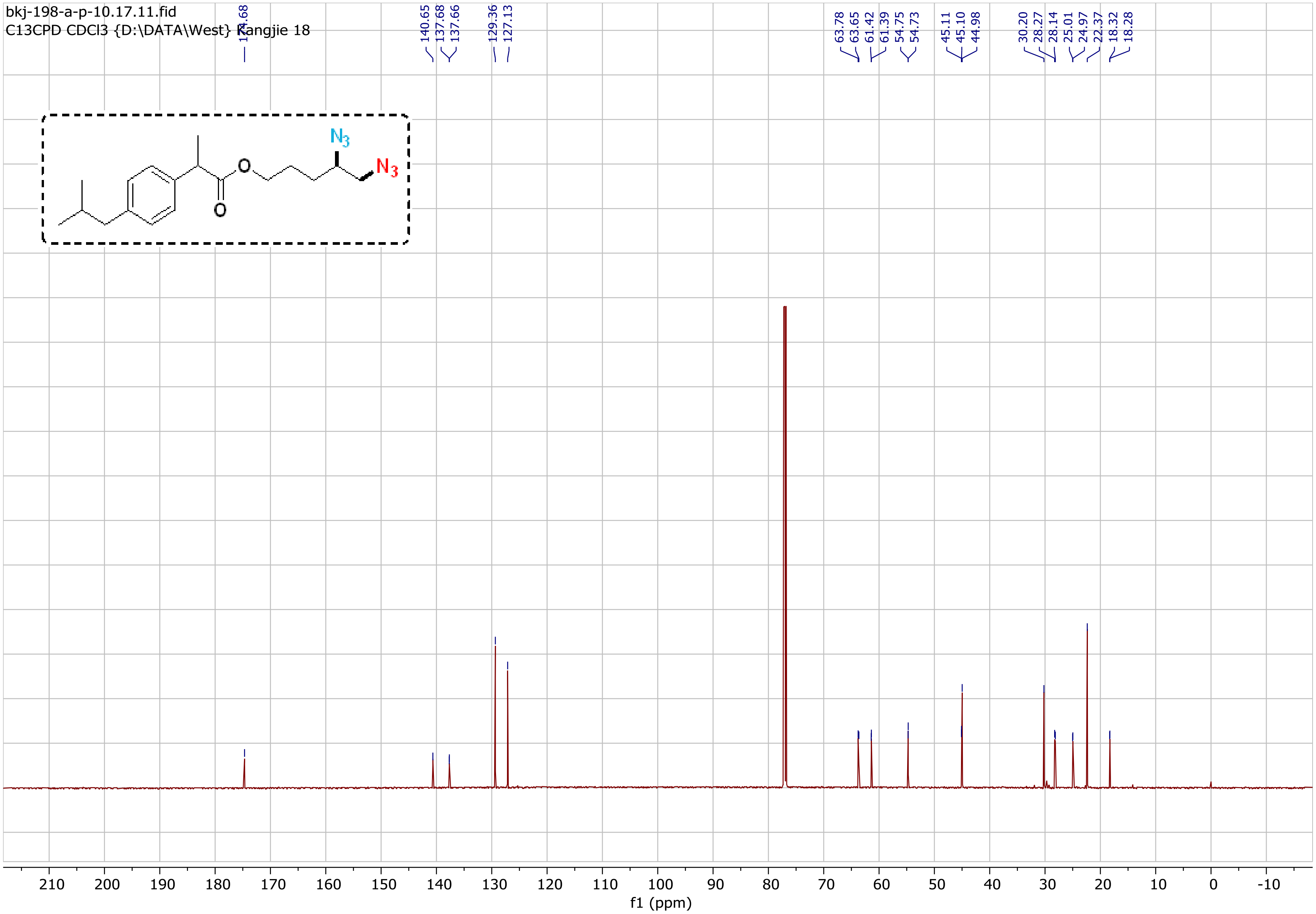

Supplementary Figure 87. ^{13}C NMR Spectrum of **41** (151 MHz, $CDCl_3$)

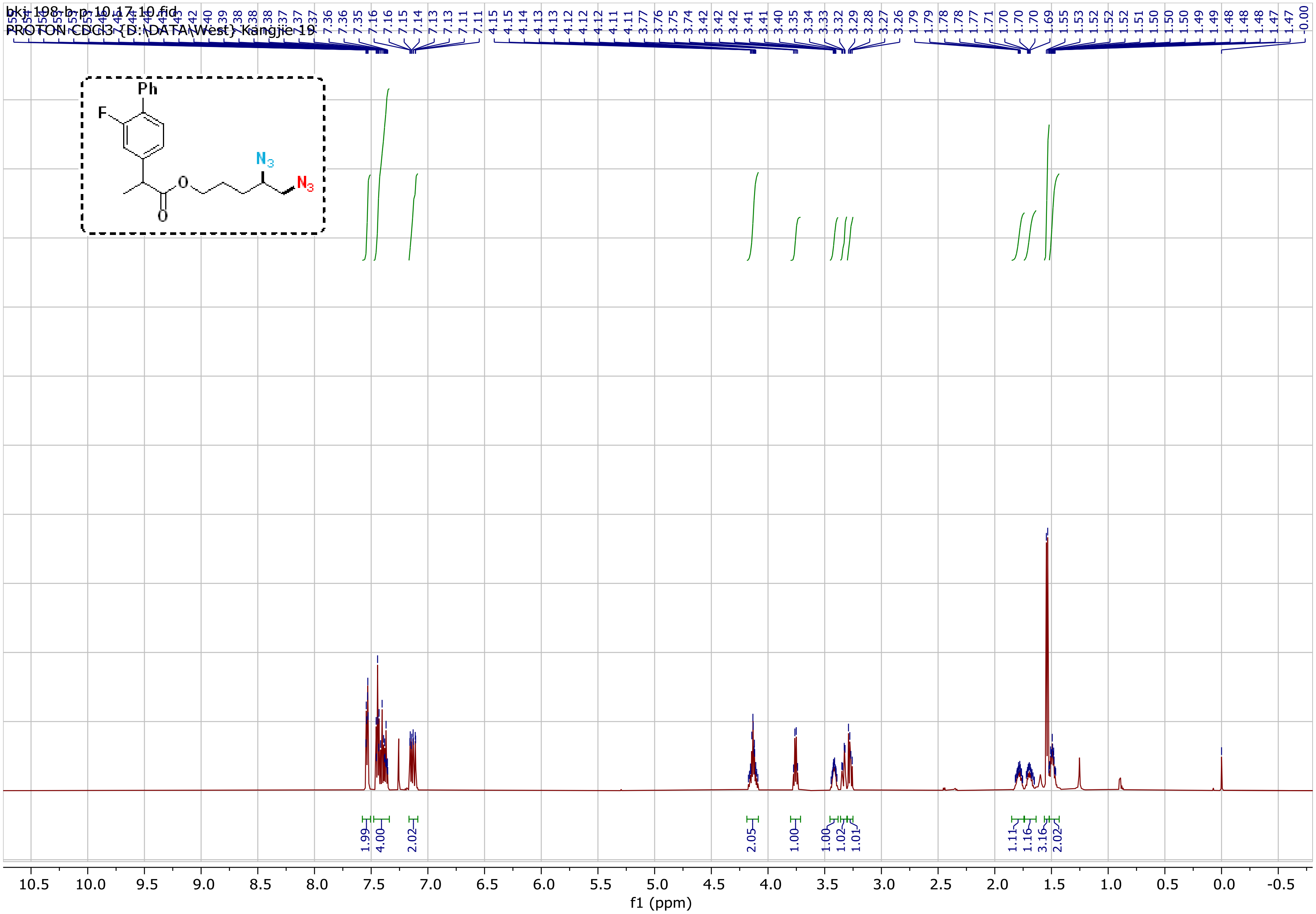

Supplementary Figure 88. ^{1}H NMR Spectrum of **42** (600 MHz, $CDCl_3$)

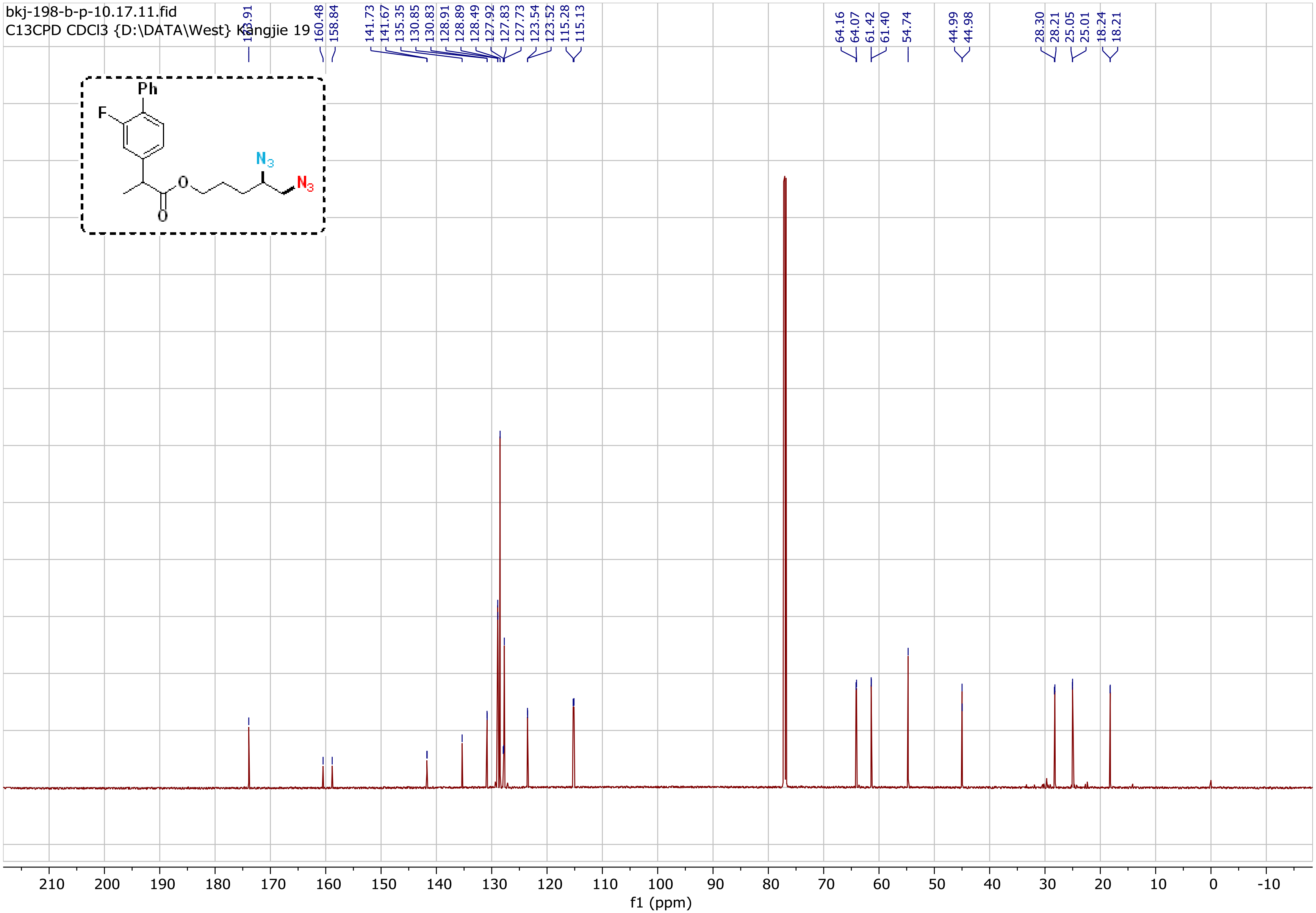

Supplementary Figure 89. ^{13}C NMR Spectrum of **42** (151 MHz, $CDCl_3$)

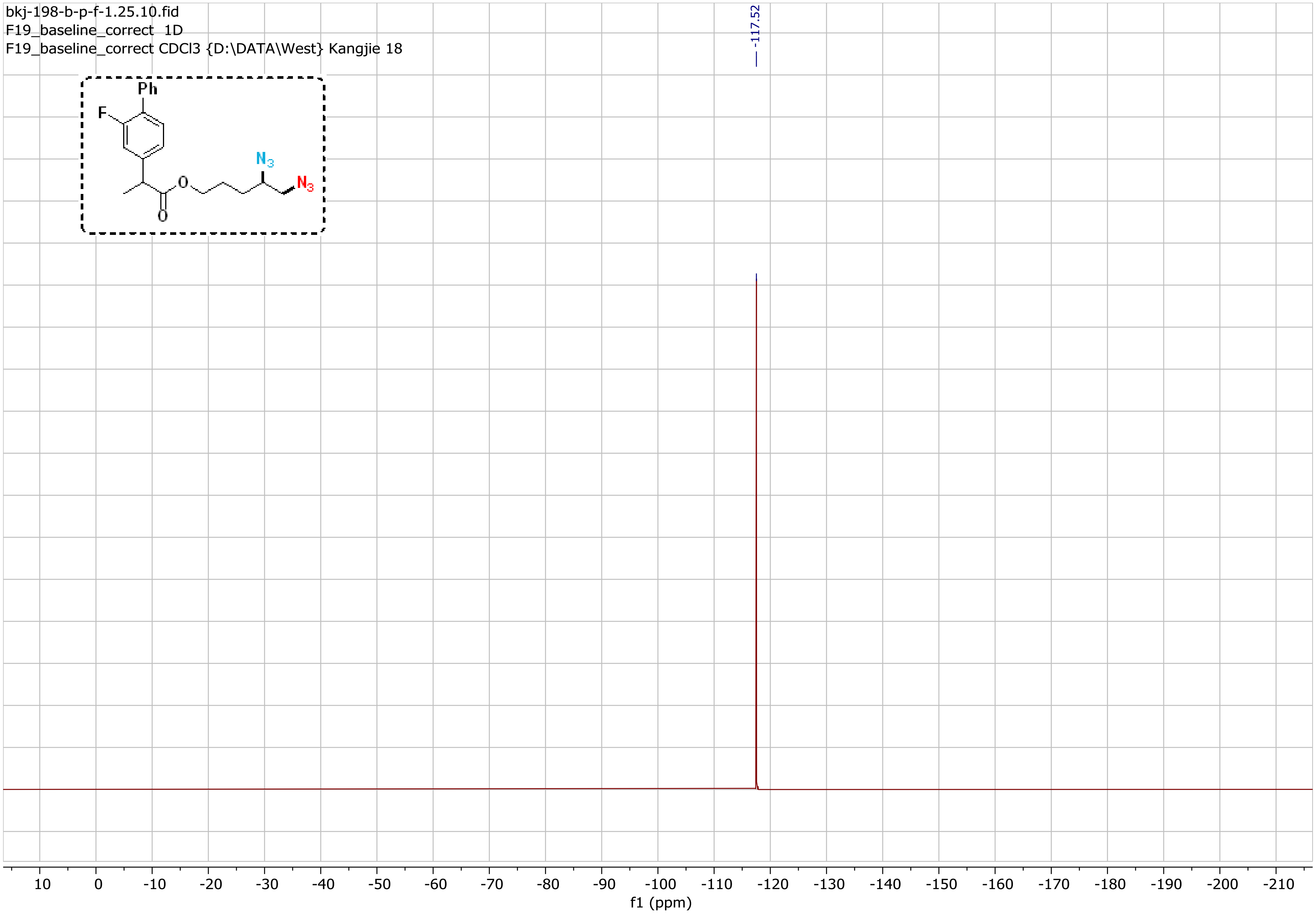

Supplementary Figure 90. ^{19}F NMR Spectrum of **42** (565 MHz, $CDCl_3$)

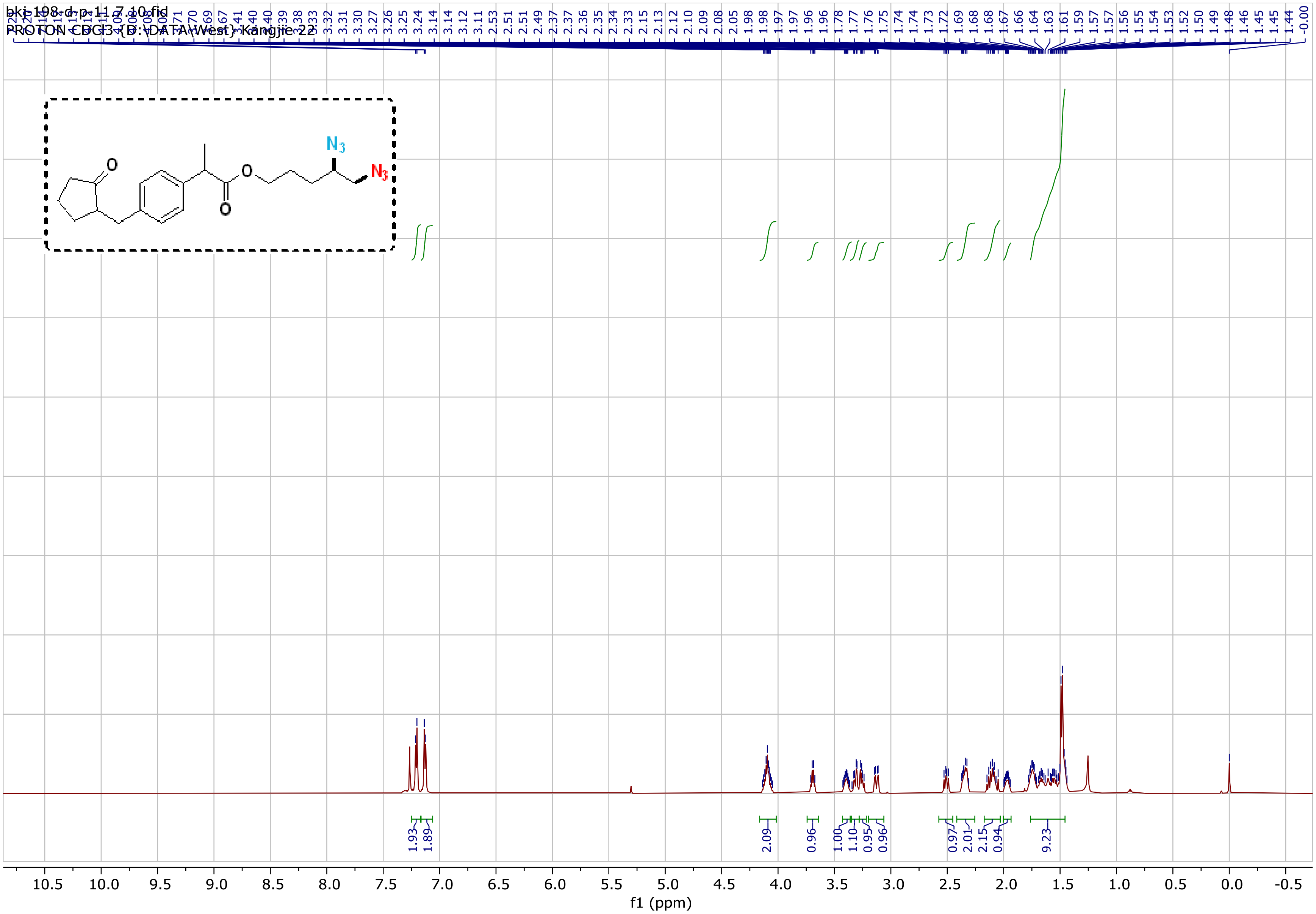

Supplementary Figure 91. ^{1}H NMR Spectrum of **43** (600 MHz, $CDCl_3$)

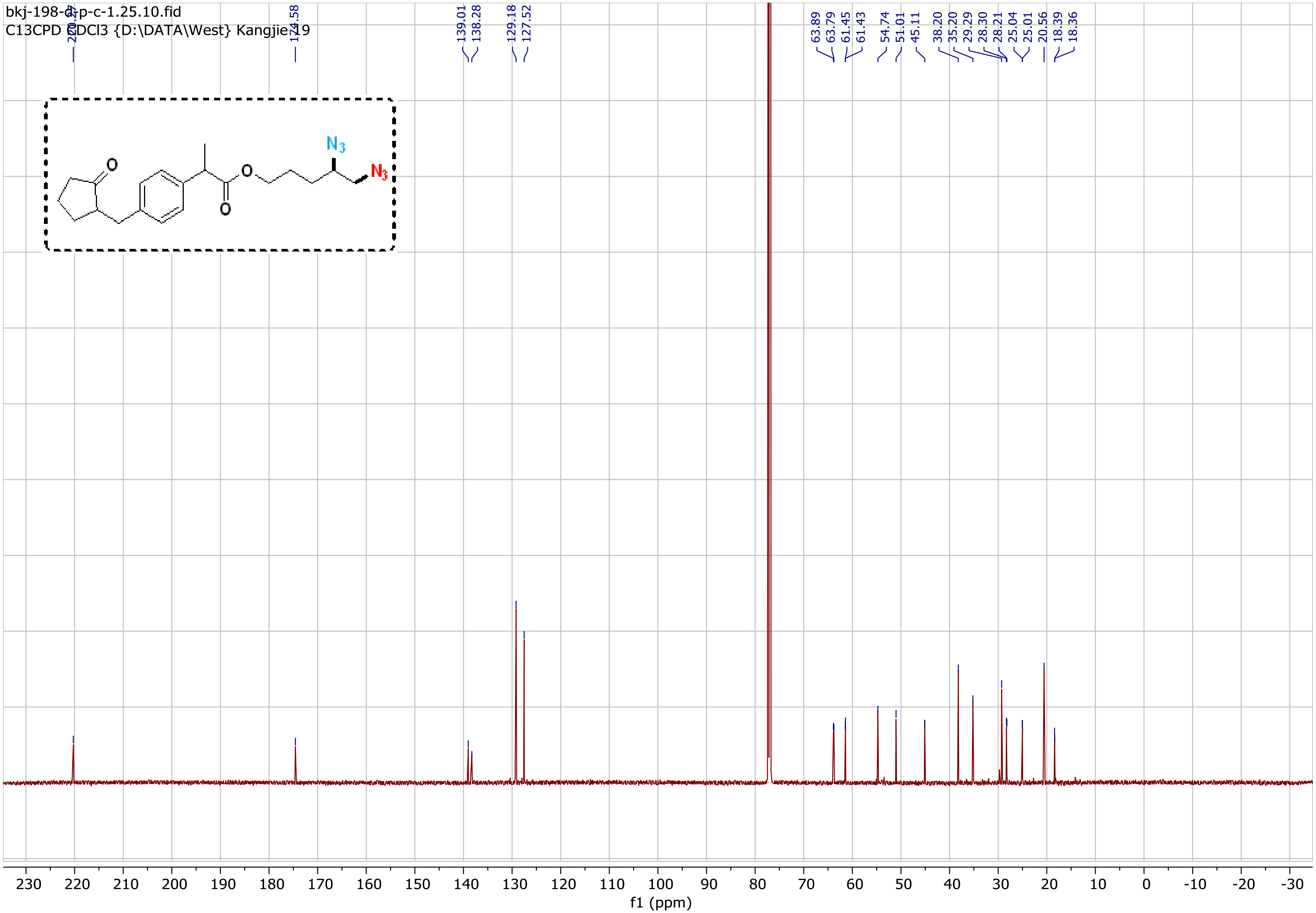

Supplementary Figure 92. ^{13}C NMR Spectrum of **43** (151 MHz, $CDCl_3$)

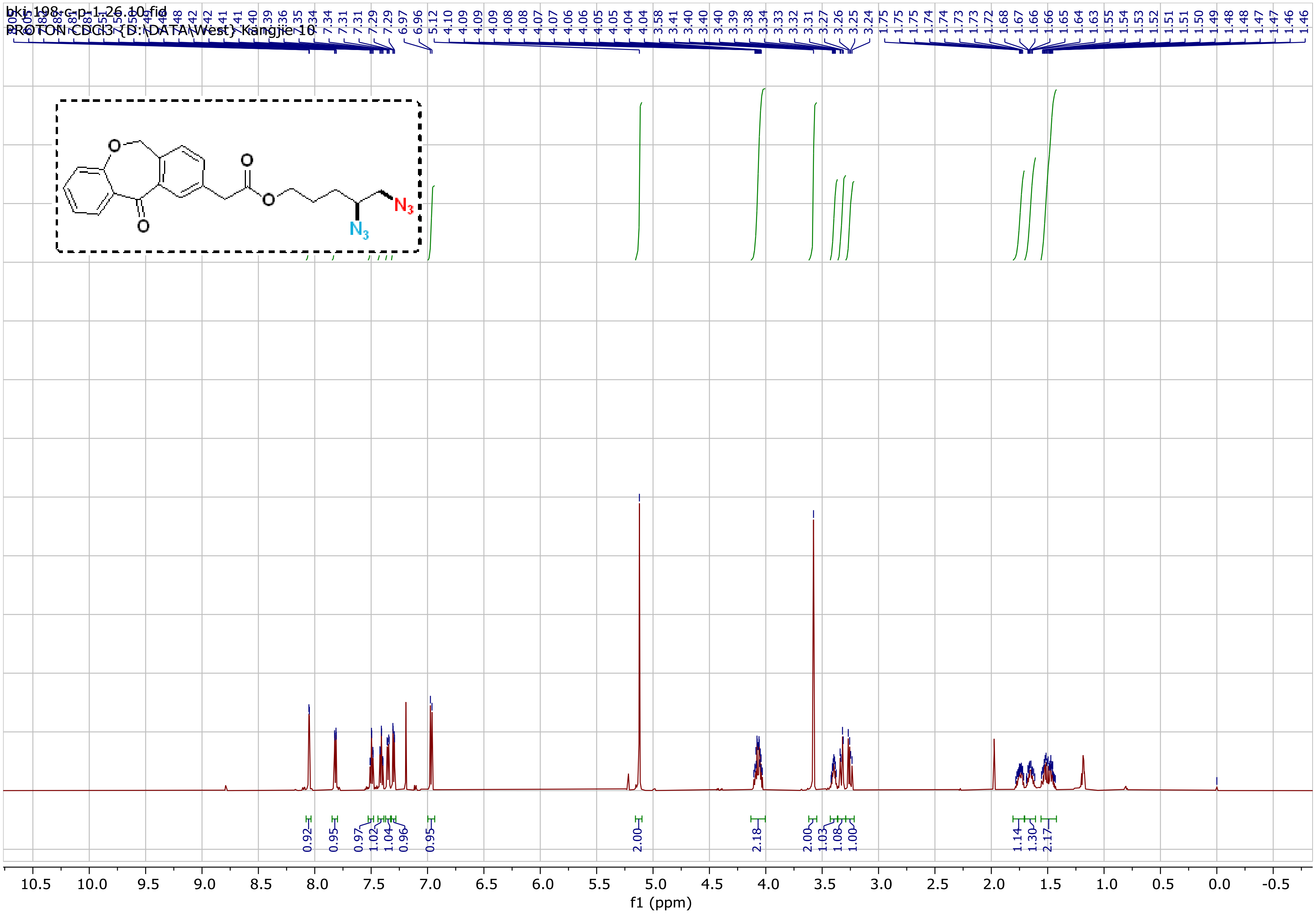

Supplementary Figure 93. ^{1}H NMR Spectrum of **44** (600 MHz, $CDCl_3$)

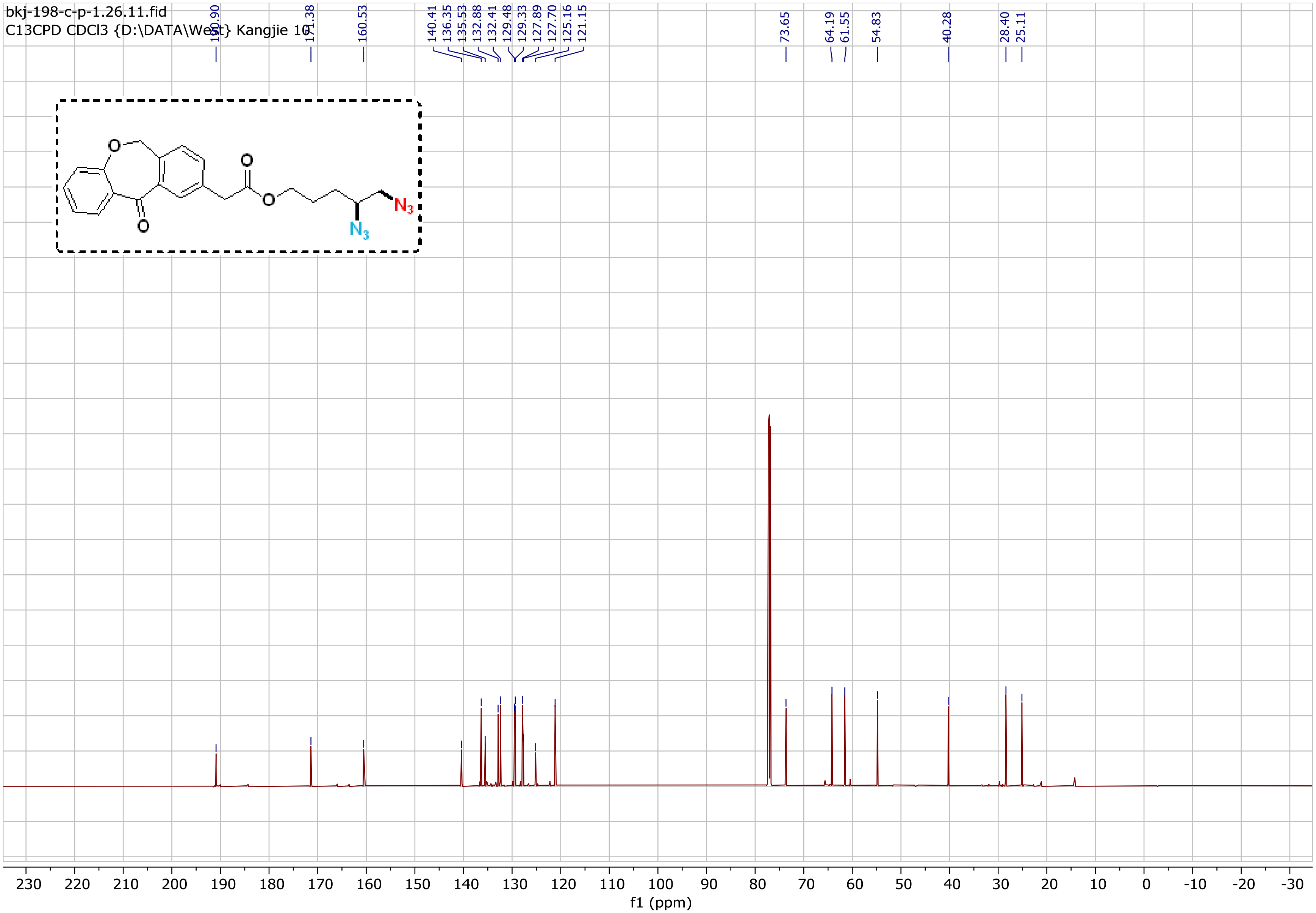

Supplementary Figure 94. ^{13}C NMR Spectrum of **44** (151 MHz, $CDCl_3$)

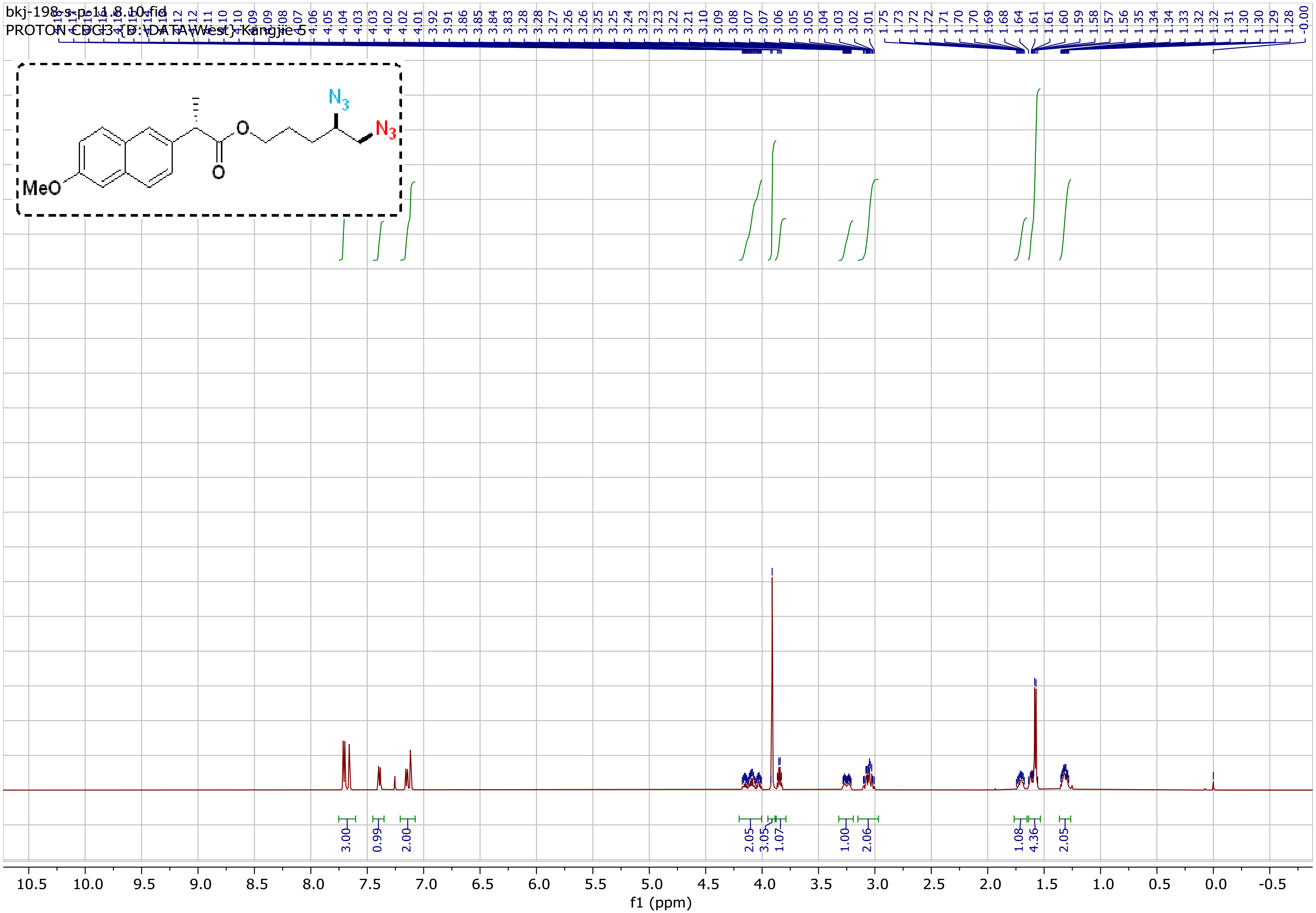

Supplementary Figure 95. ^{1}H NMR Spectrum of **45** (600 MHz, $CDCl_3$)

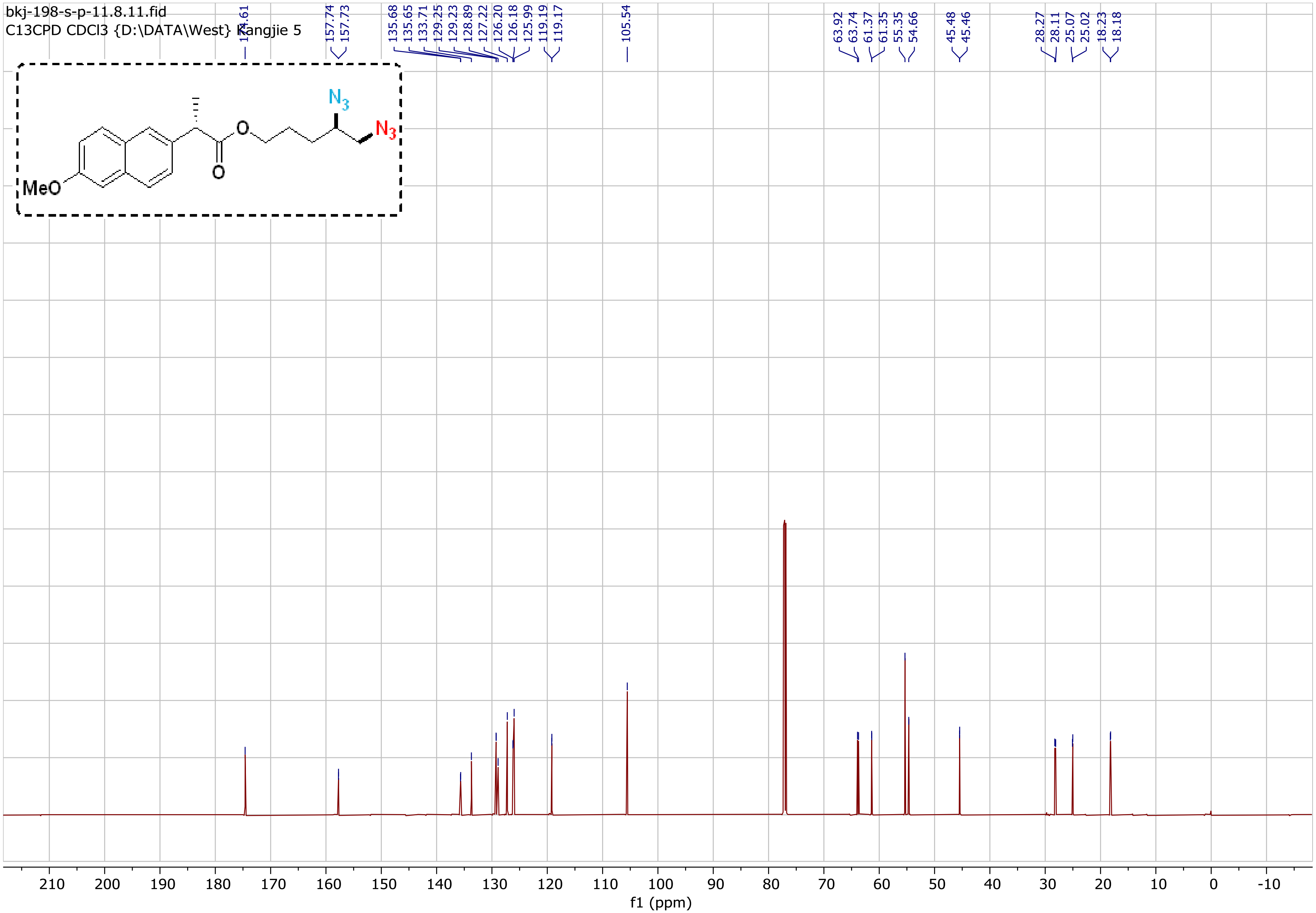

Supplementary Figure 96. ^{13}C NMR Spectrum of **45** (151 MHz, $CDCl_3$)

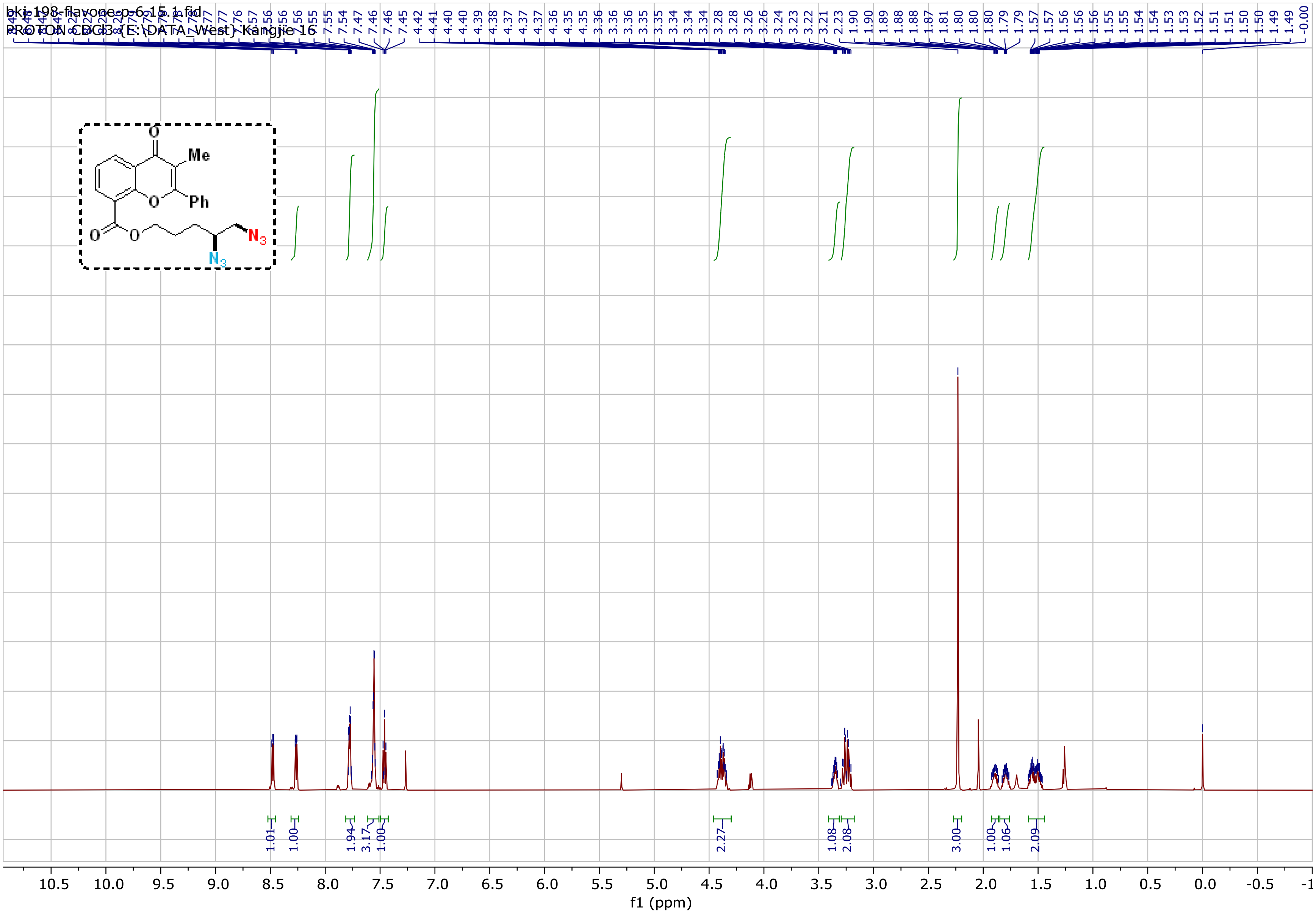

Supplementary Figure 97. ^{1}H NMR Spectrum of **46** (600 MHz, $CDCl_3$)

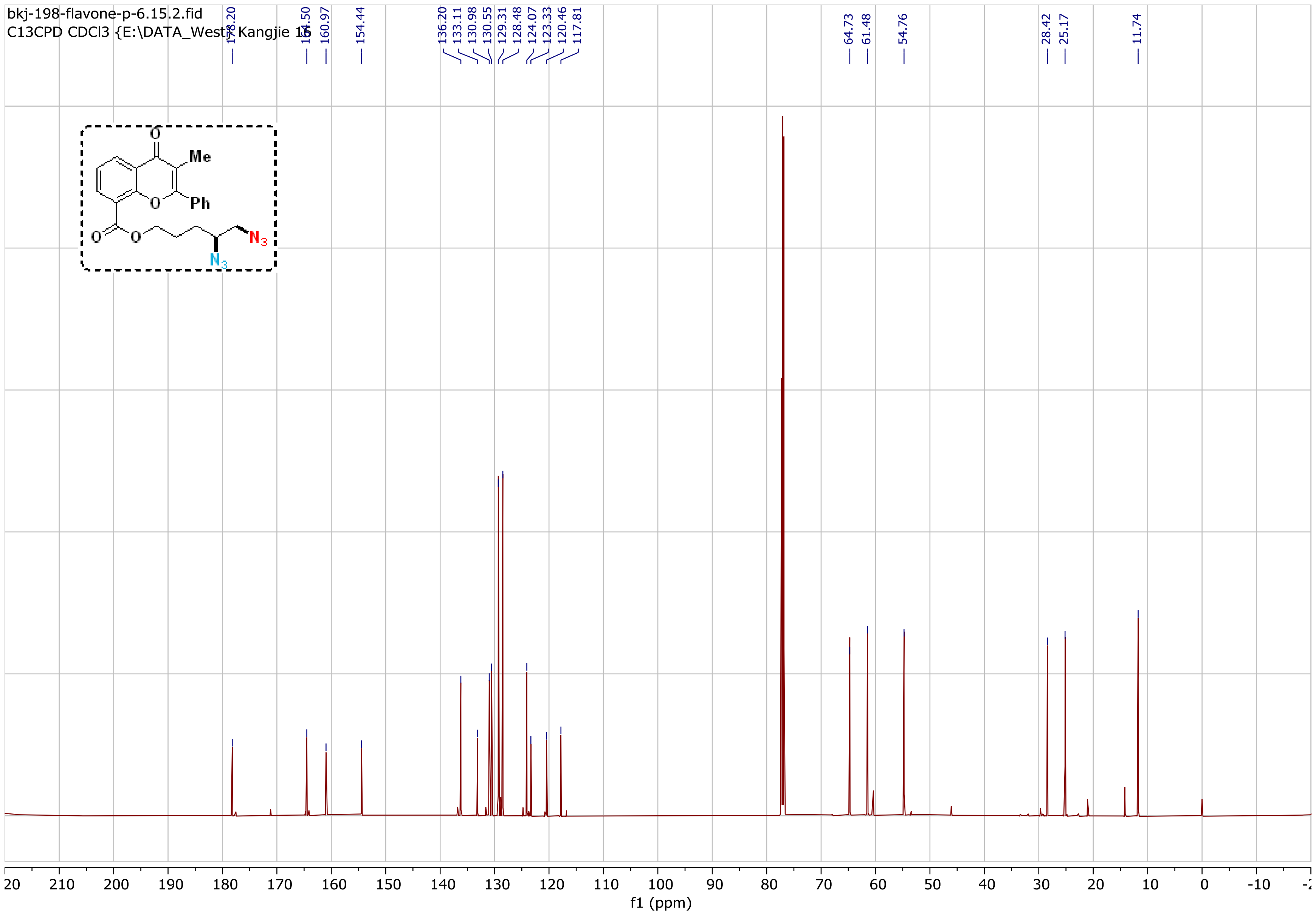

Supplementary Figure 98. ^{13}C NMR Spectrum of **46** (151 MHz, $CDCl_3$)

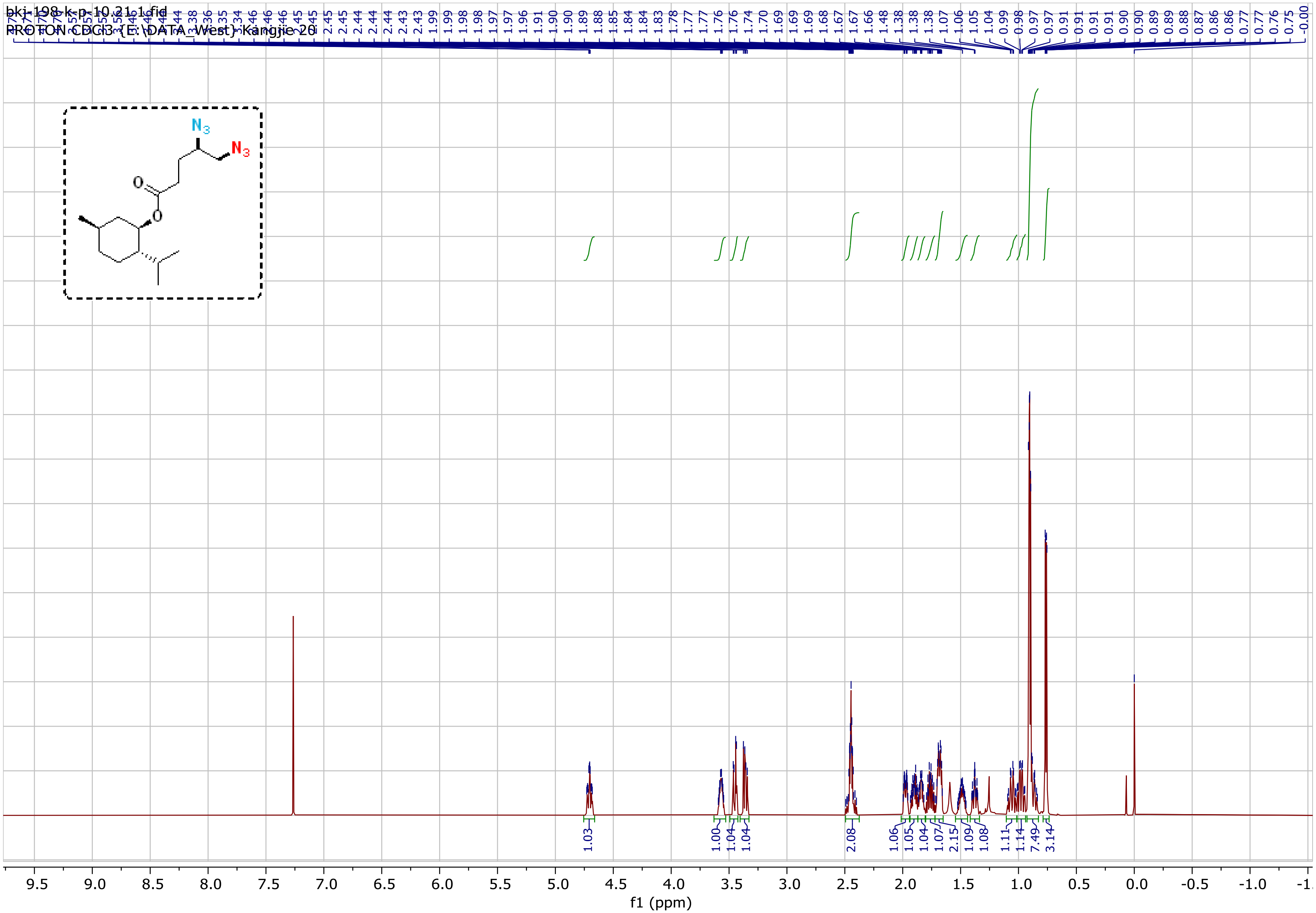

Supplementary Figure 99. ^{1}H NMR Spectrum of **47** (600 MHz, $CDCl_3$)

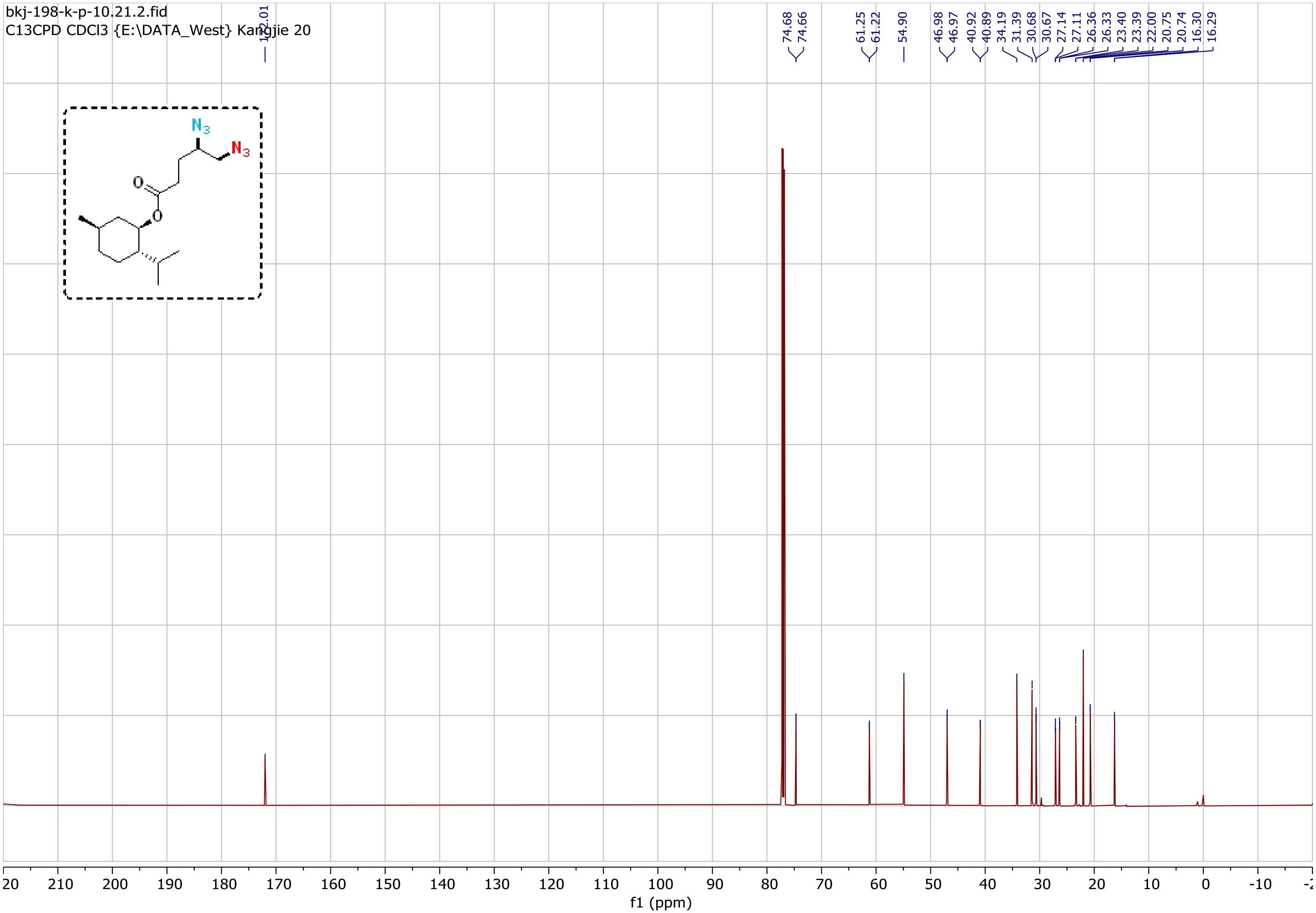

Supplementary Figure 100. ^{13}C NMR Spectrum of **47** (151 MHz, $CDCl_3$)

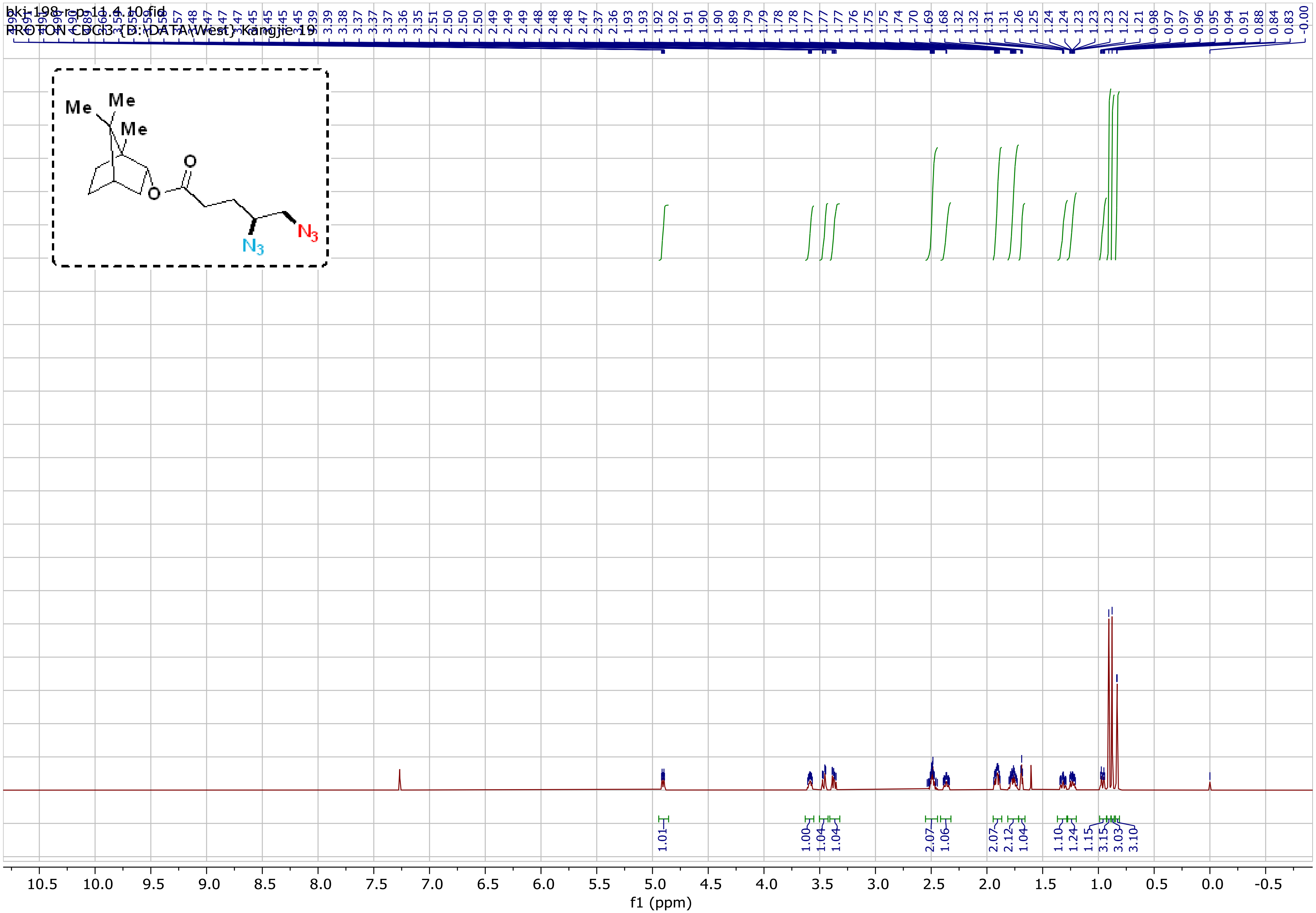

Supplementary Figure 101. ^{1}H NMR Spectrum of **48** (600 MHz, $CDCl_3$)

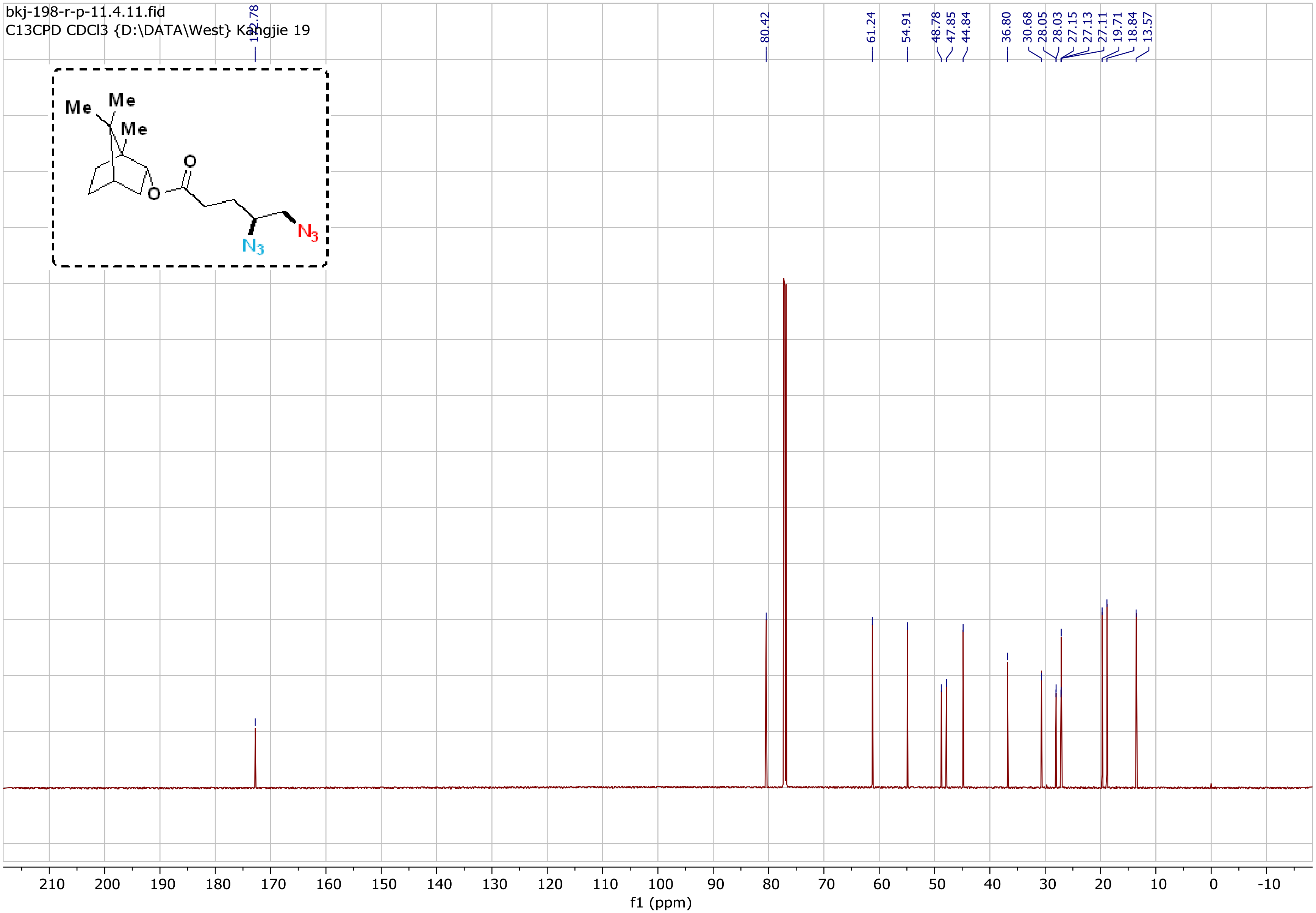

Supplementary Figure 102. ^{13}C NMR Spectrum of **48** (151 MHz, $CDCl_3$)

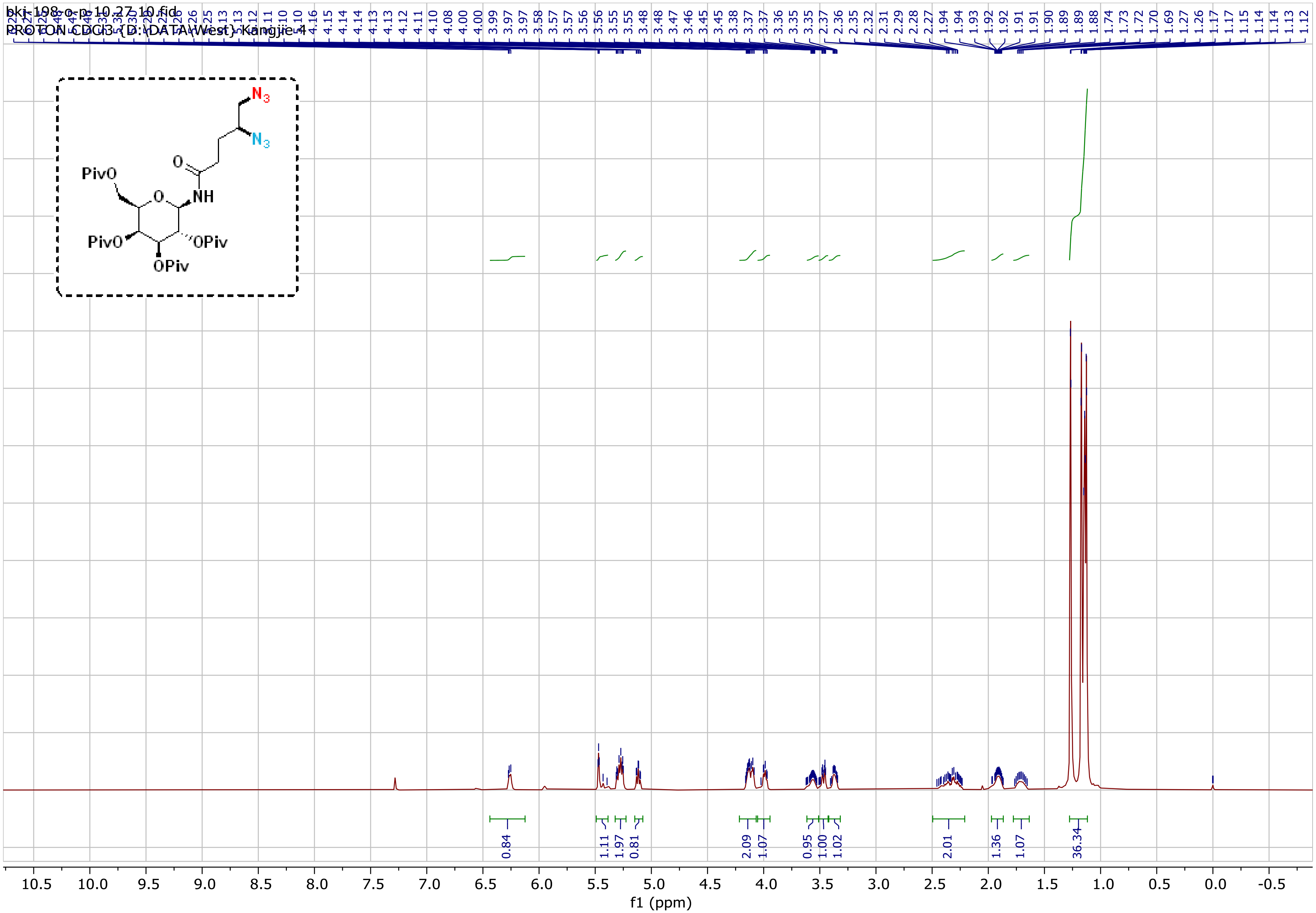

Supplementary Figure 103. ^{1}H NMR Spectrum of **49** (600 MHz, $CDCl_3$)

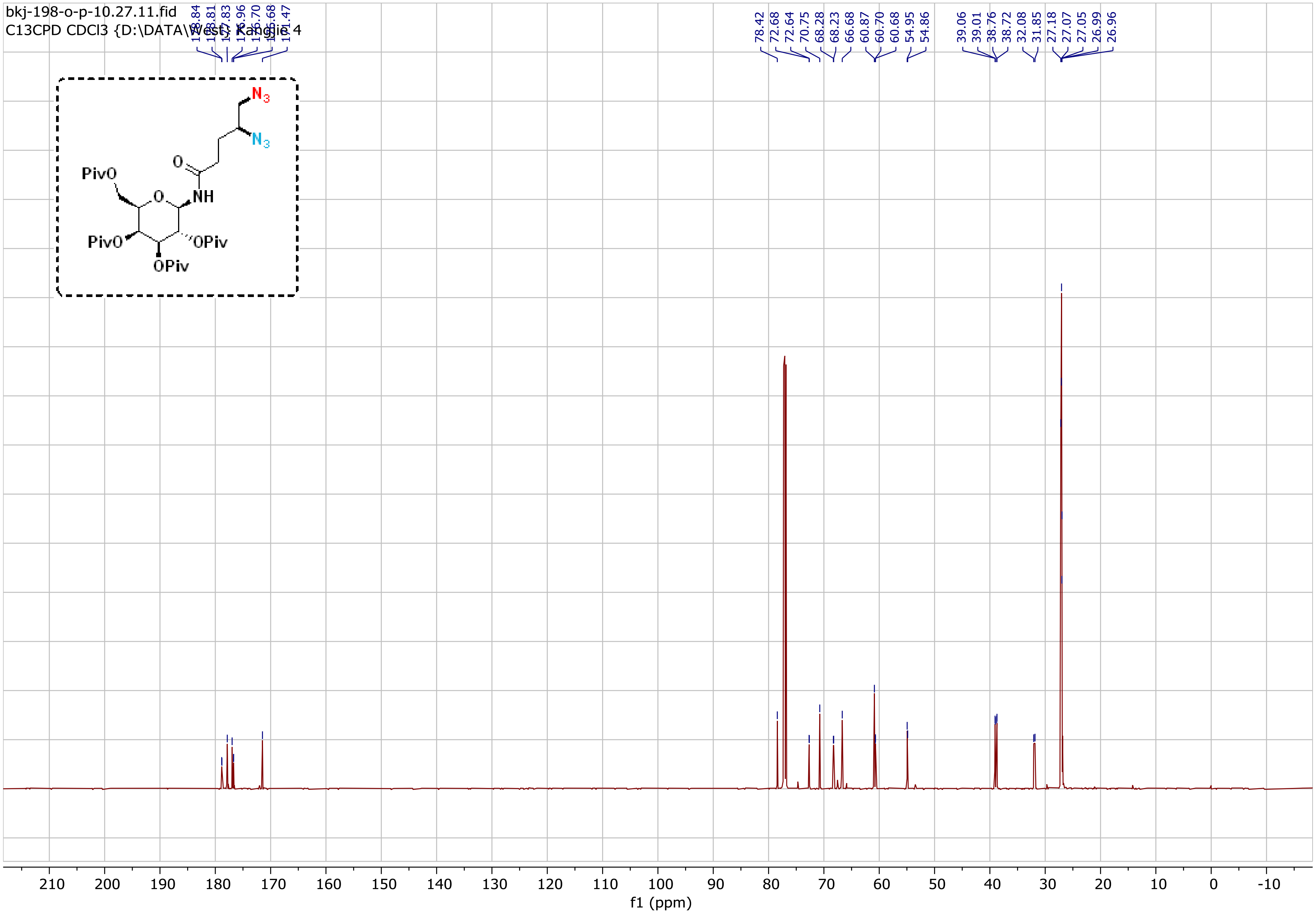

Supplementary Figure 104. ^{13}C NMR Spectrum of **49** (151 MHz, CDCl$_3$)

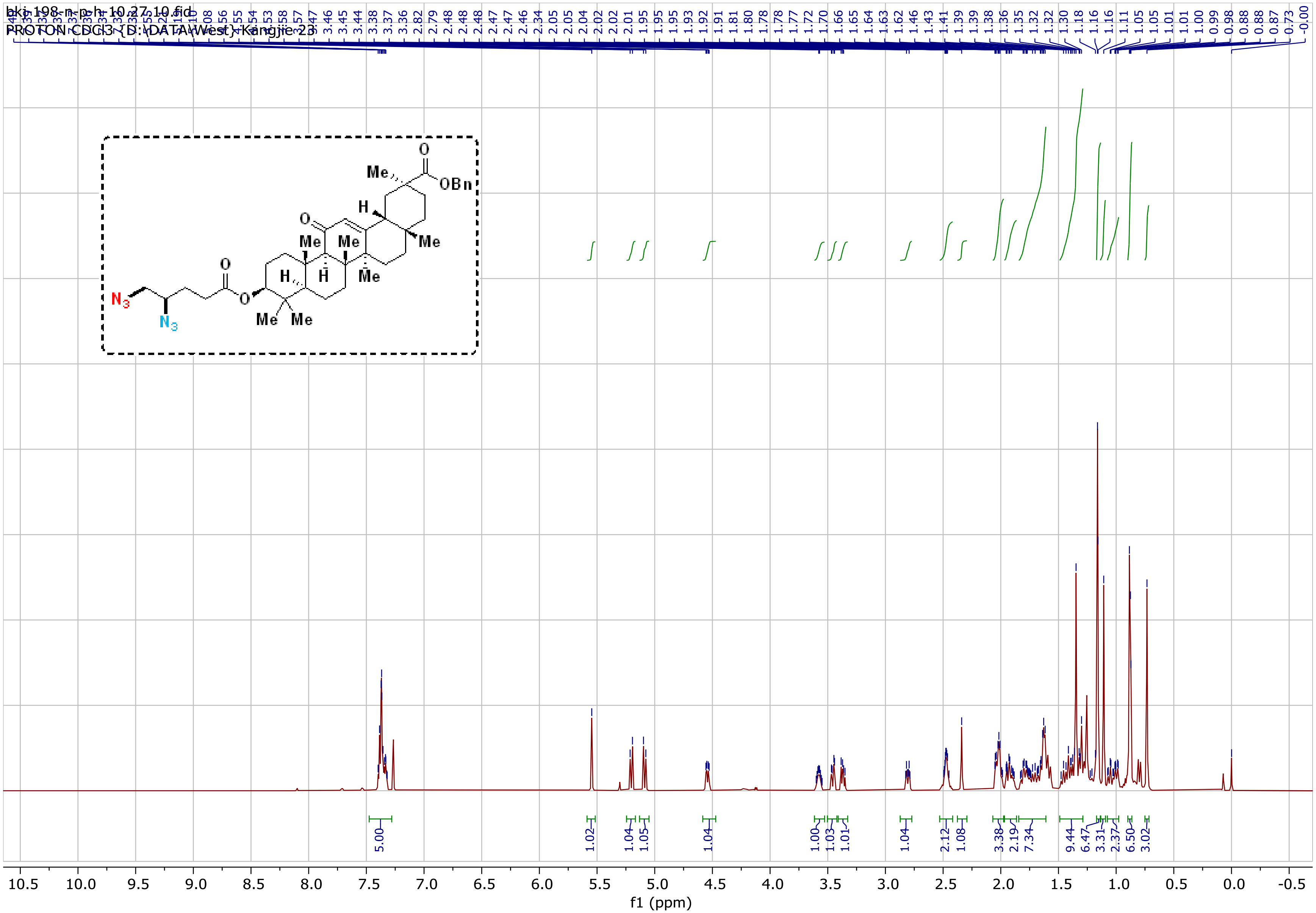

Supplementary Figure 105. ^{1}H NMR Spectrum of **50** (600 MHz, $CDCl_3$)

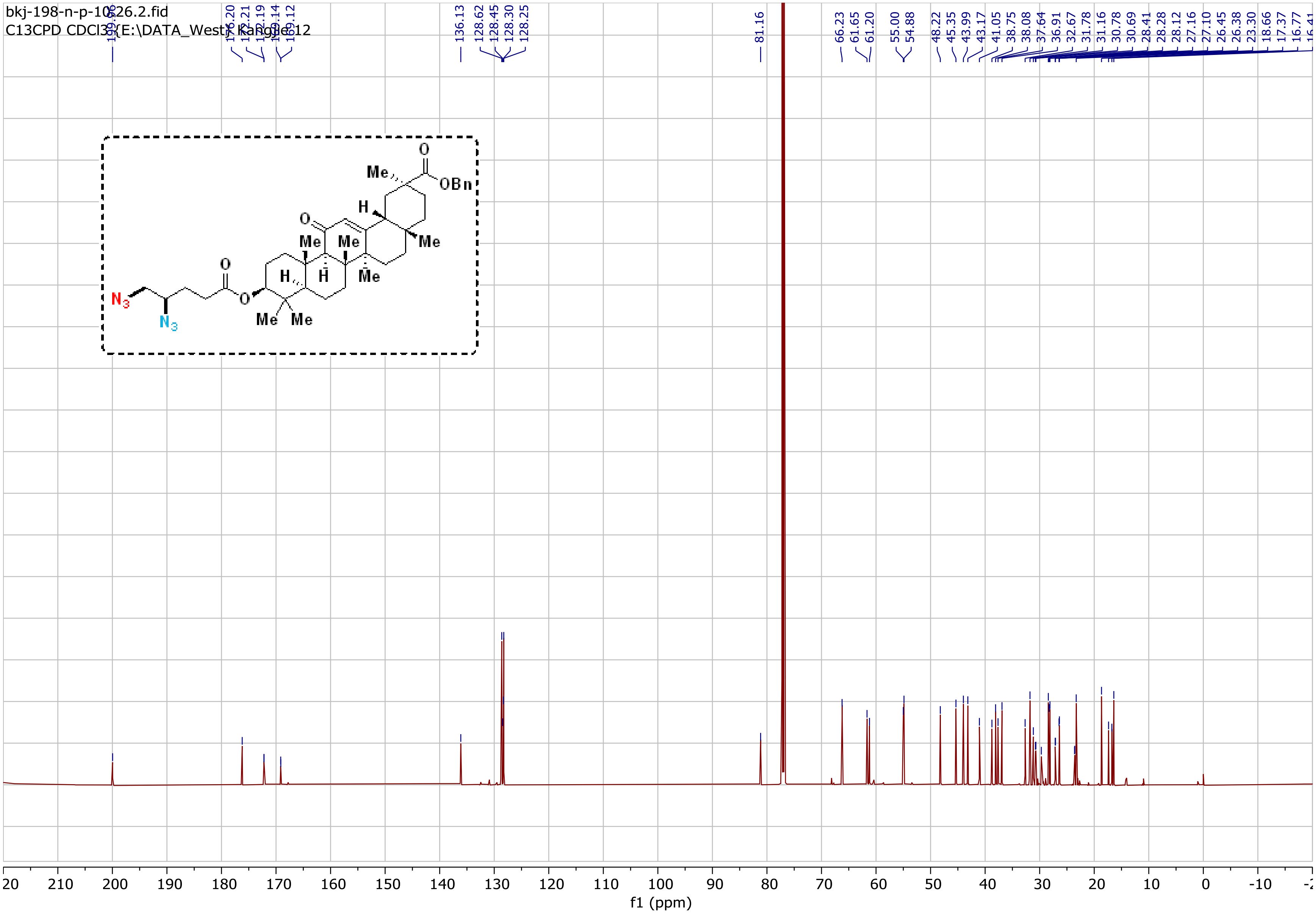

Supplementary Figure 106. ^{13}C NMR Spectrum of **50** (151 MHz, $CDCl_3$)

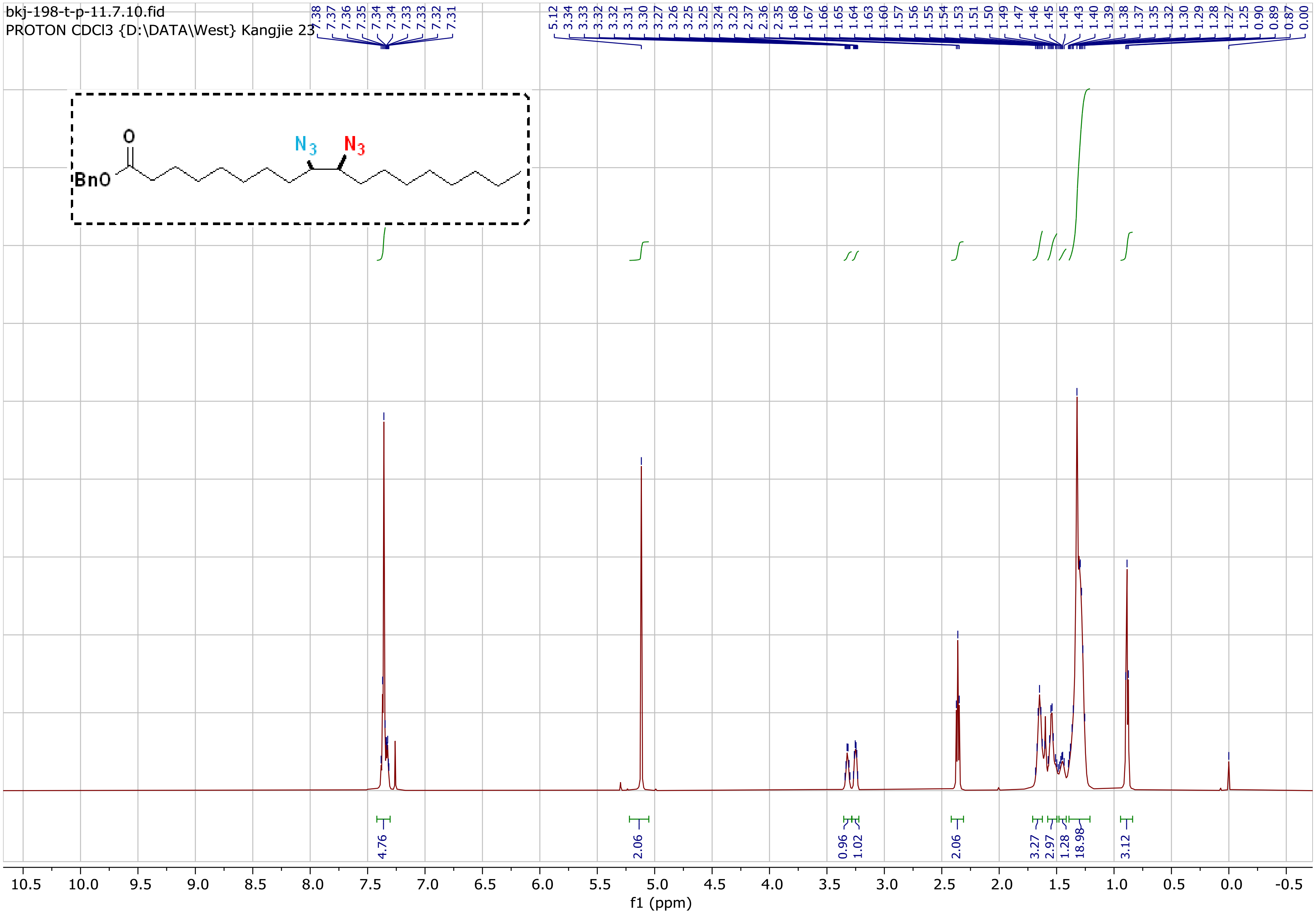

Supplementary Figure 107. ^{1}H NMR Spectrum of **51** (600 MHz, $CDCl_3$)

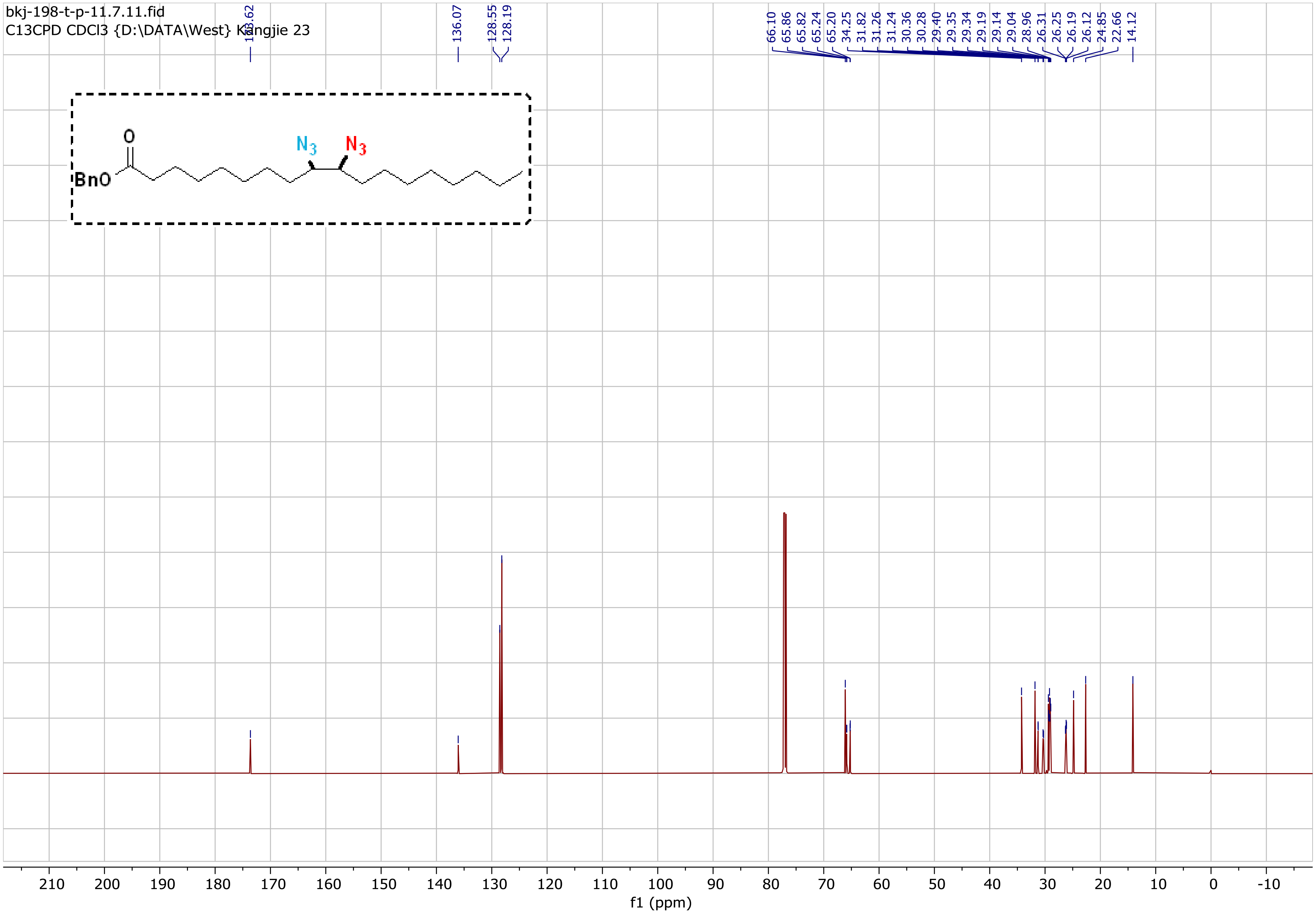

Supplementary Figure 108. ^{13}C NMR Spectrum of **51** (151 MHz, $CDCl_3$)

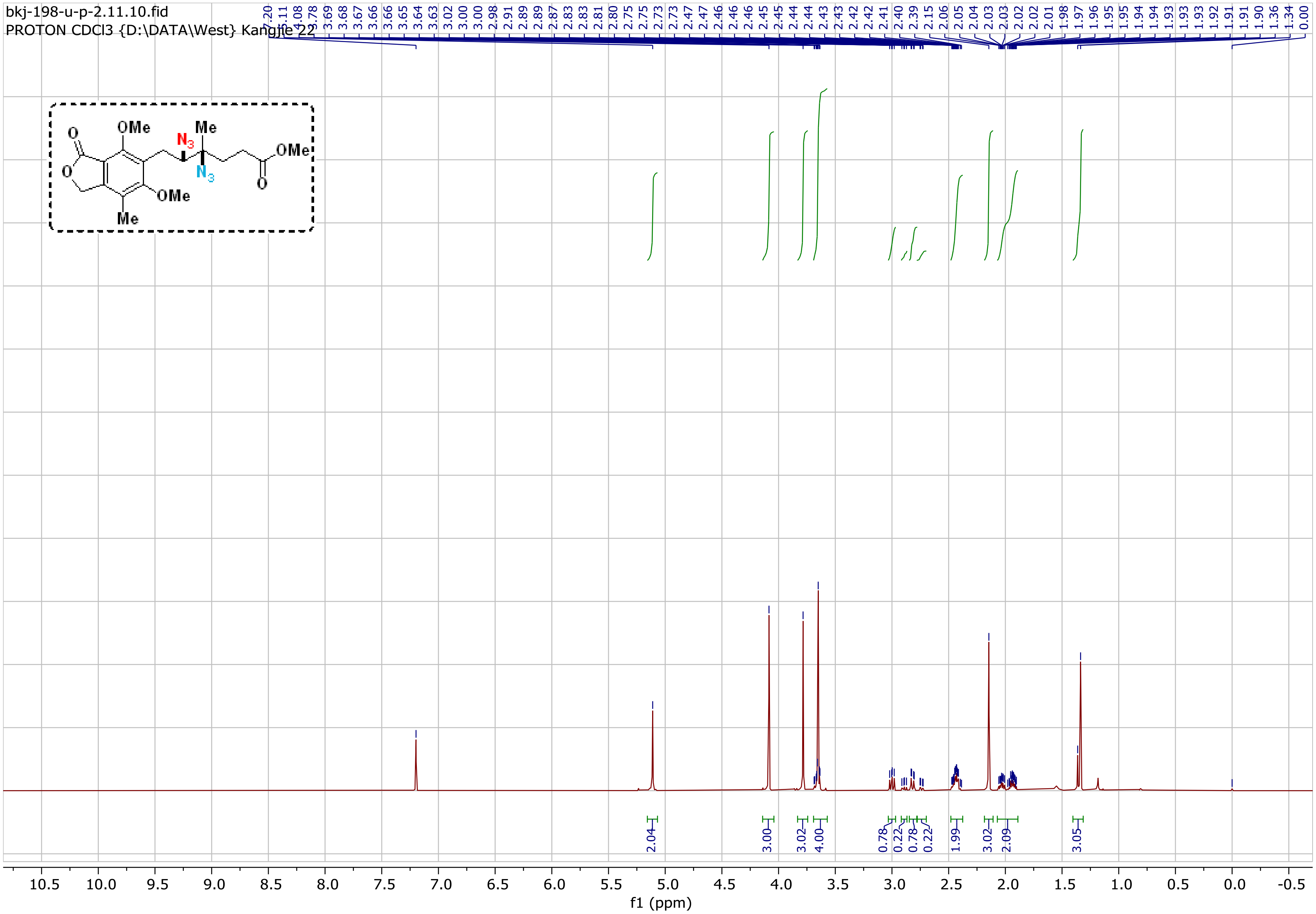

Supplementary Figure 109. ^{1}H NMR Spectrum of **52** (600 MHz, $CDCl_3$)

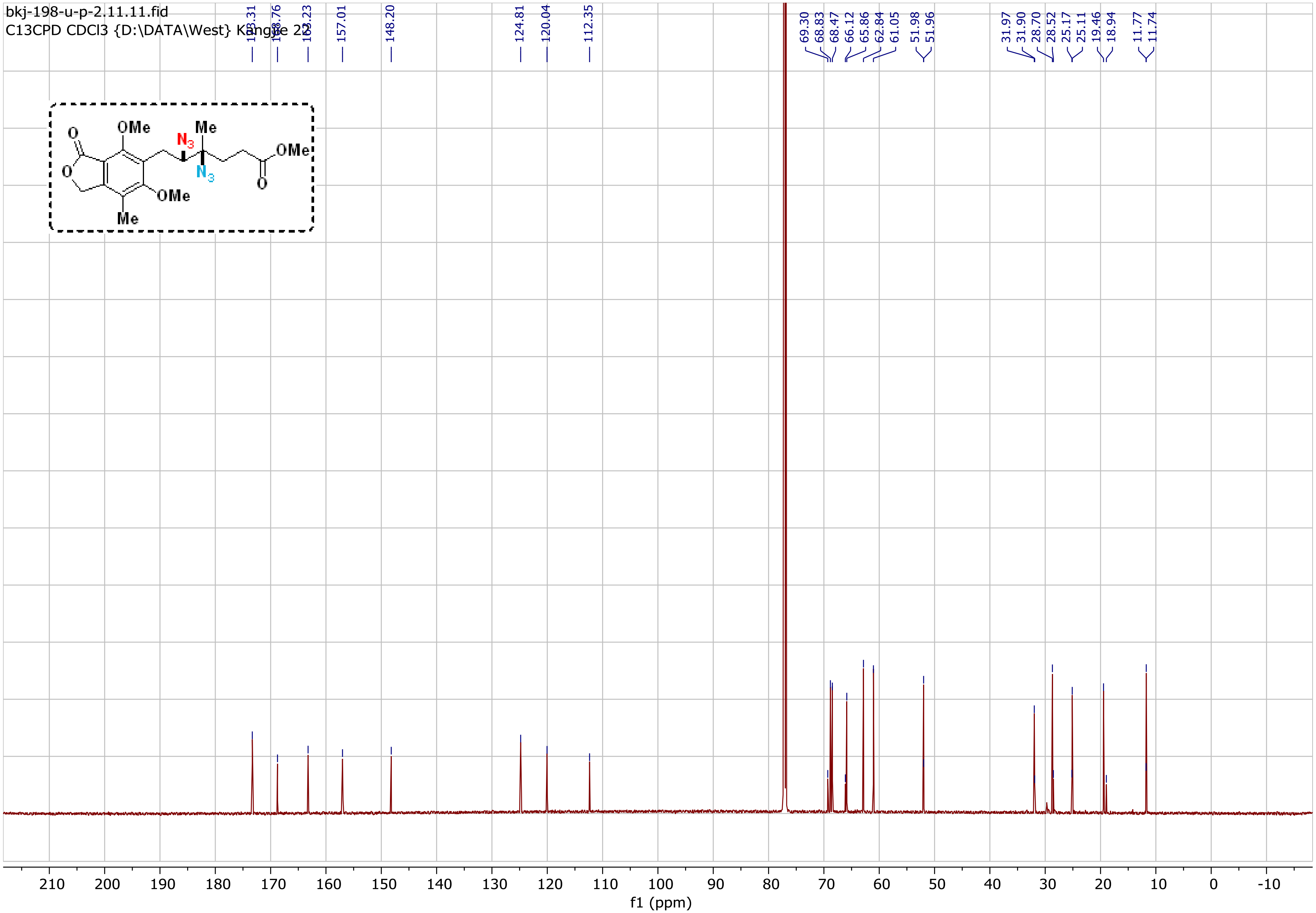

Supplementary Figure 110. ^{13}C NMR Spectrum of **52** (151 MHz, $CDCl_3$)

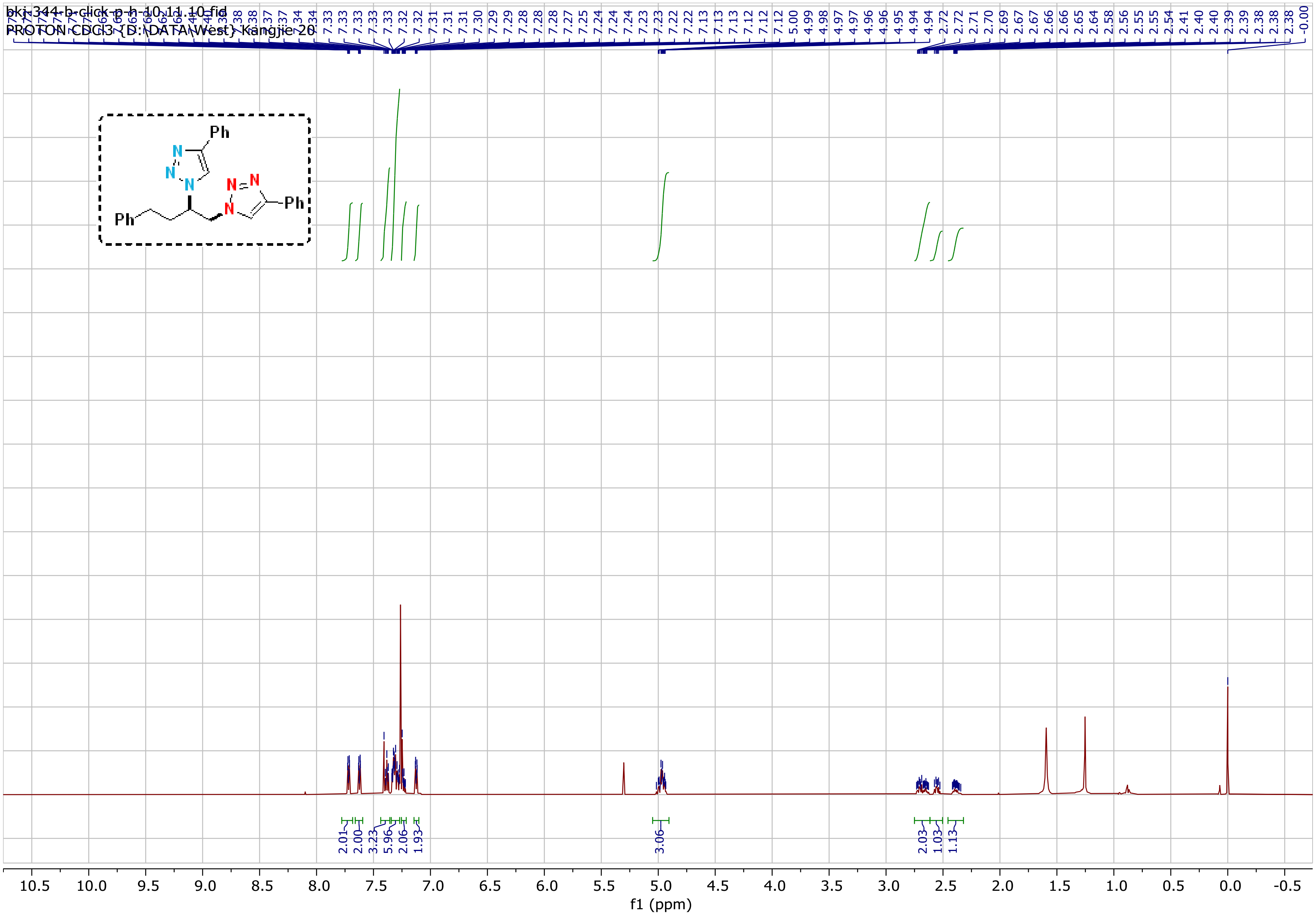

Supplementary Figure 111. ^{1}H NMR Spectrum of **53** (600 MHz, $CDCl_3$)

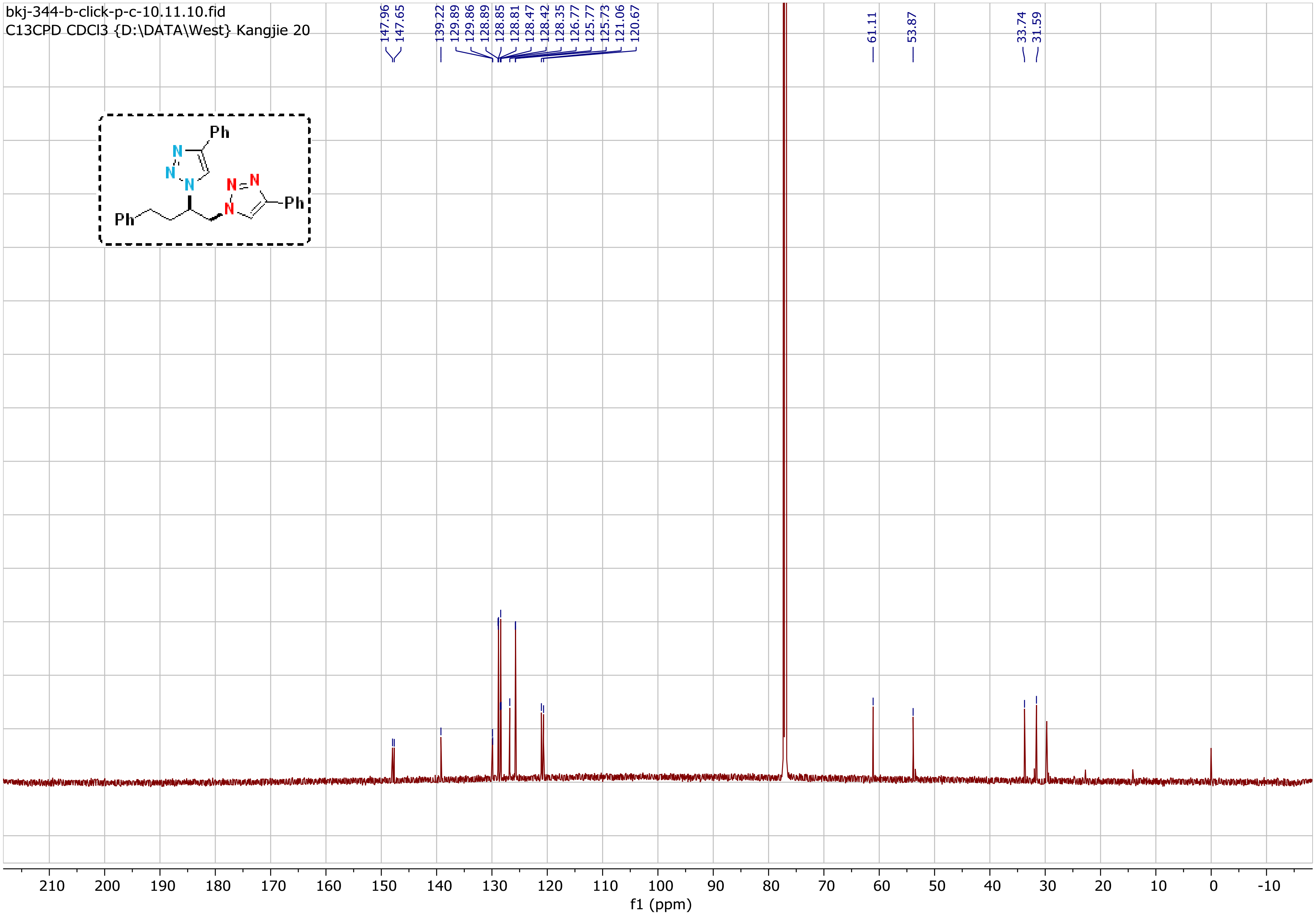

Supplementary Figure 112. ^{13}C NMR Spectrum of **53** (151 MHz, $CDCl_3$)

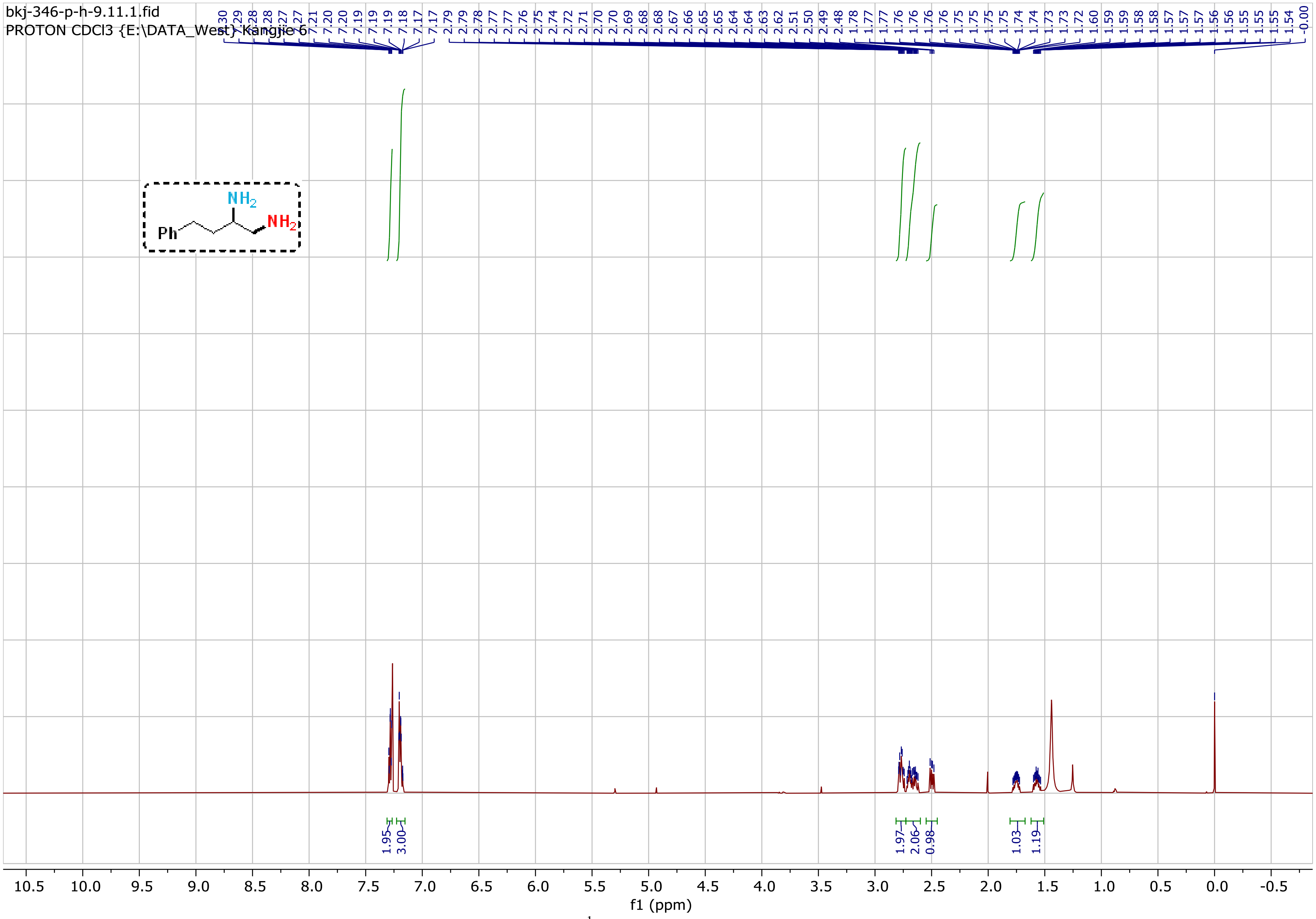

Supplementary Figure 113. ^{1}H NMR Spectrum of **54** (600 MHz, $CDCl_3$)

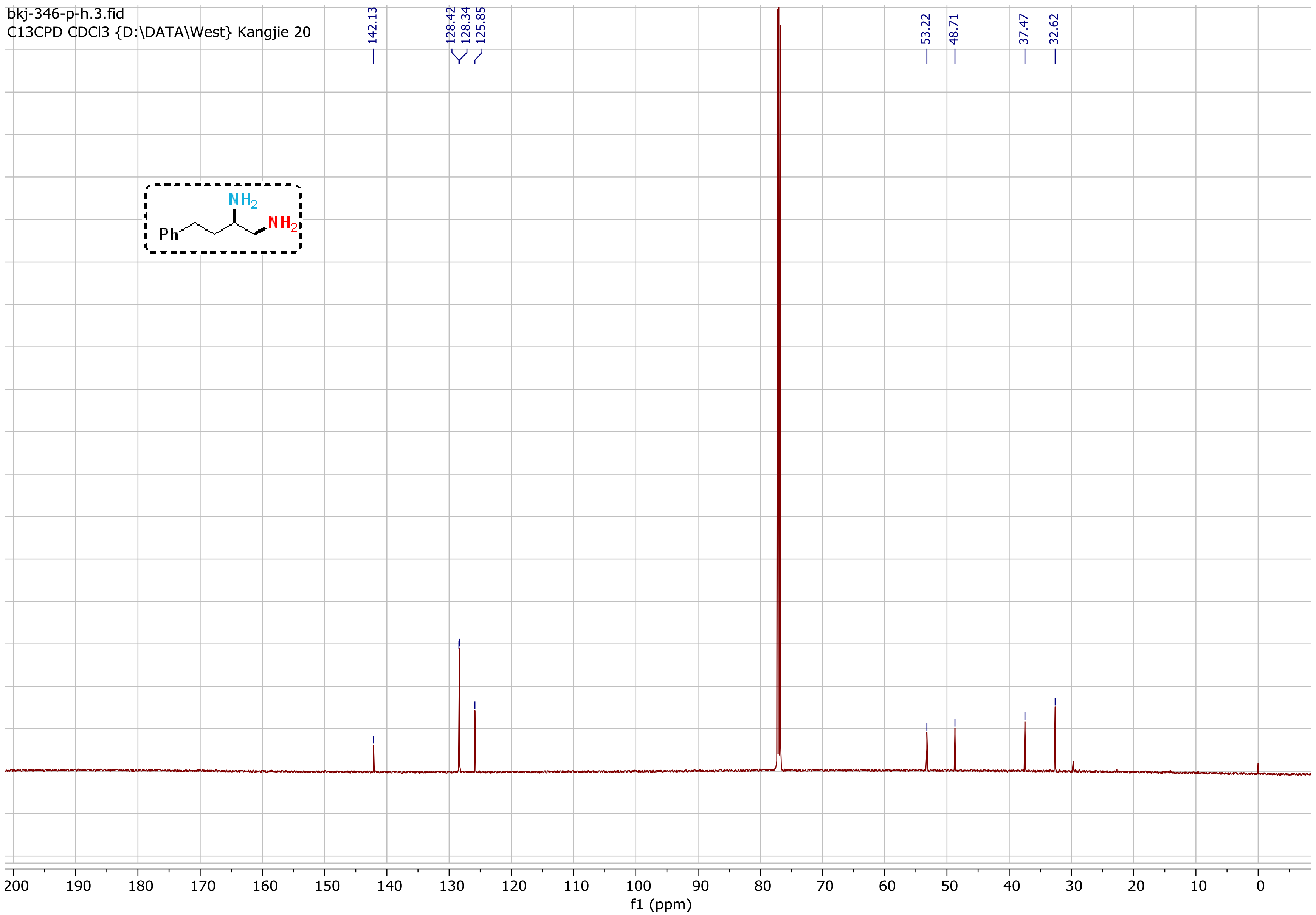

Supplementary Figure 114. ^{13}C NMR Spectrum of **54** (151 MHz, $CDCl_3$)

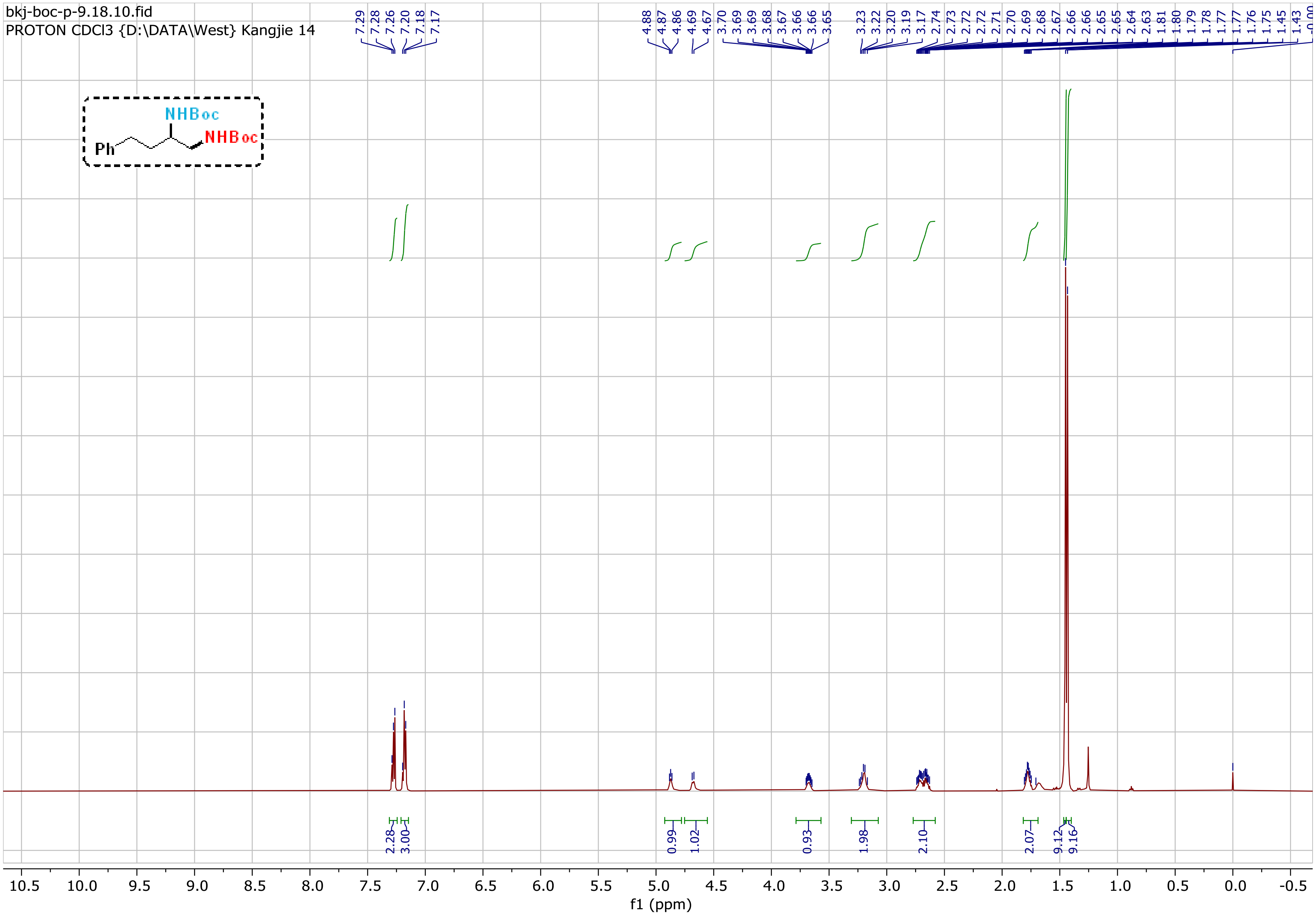

Supplementary Figure 115. ^{1}H NMR Spectrum of **55** (600 MHz, $CDCl_3$)

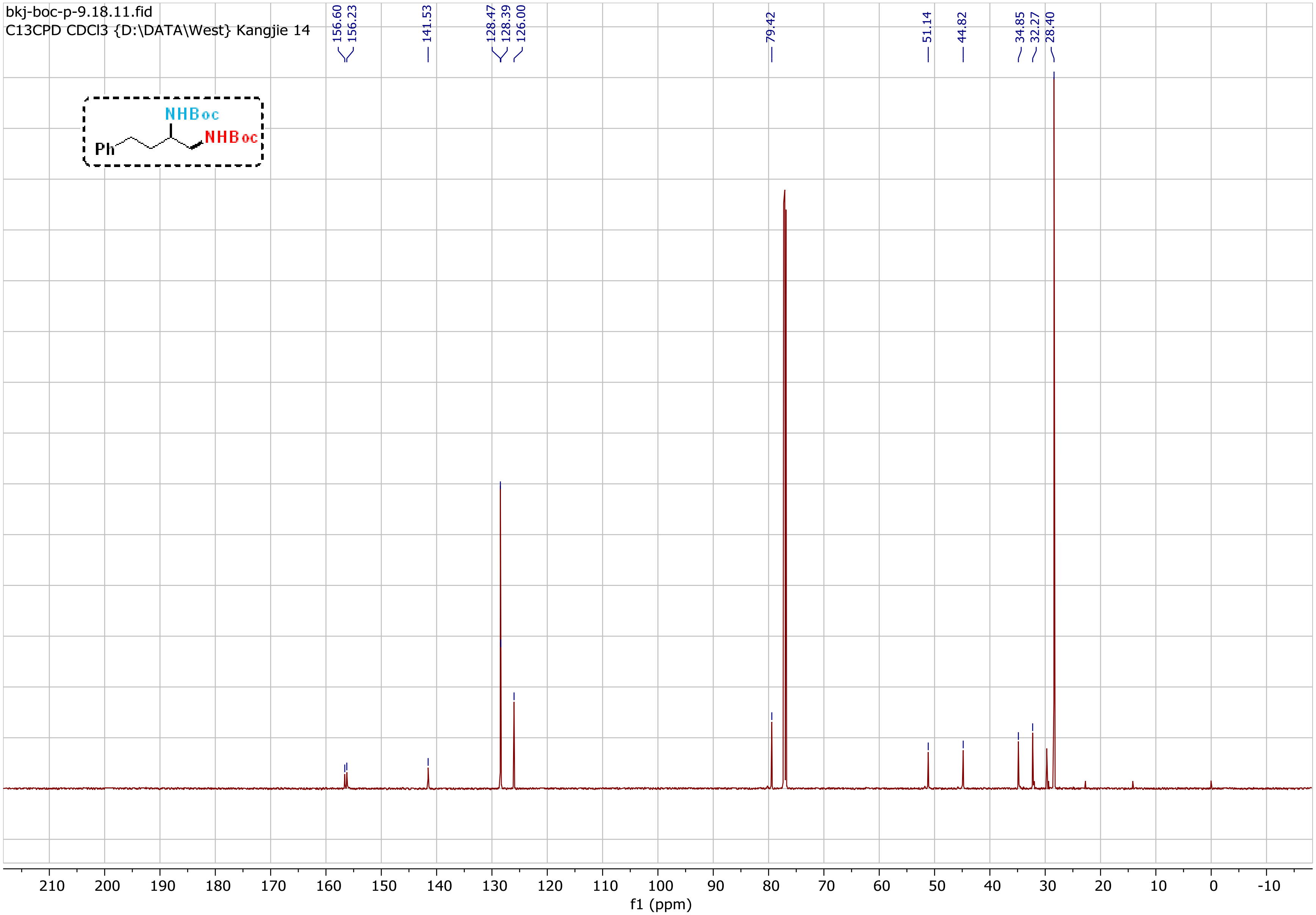

Supplementary Figure 116. ^{13}C NMR Spectrum of **55** (151 MHz, $CDCl_3$)

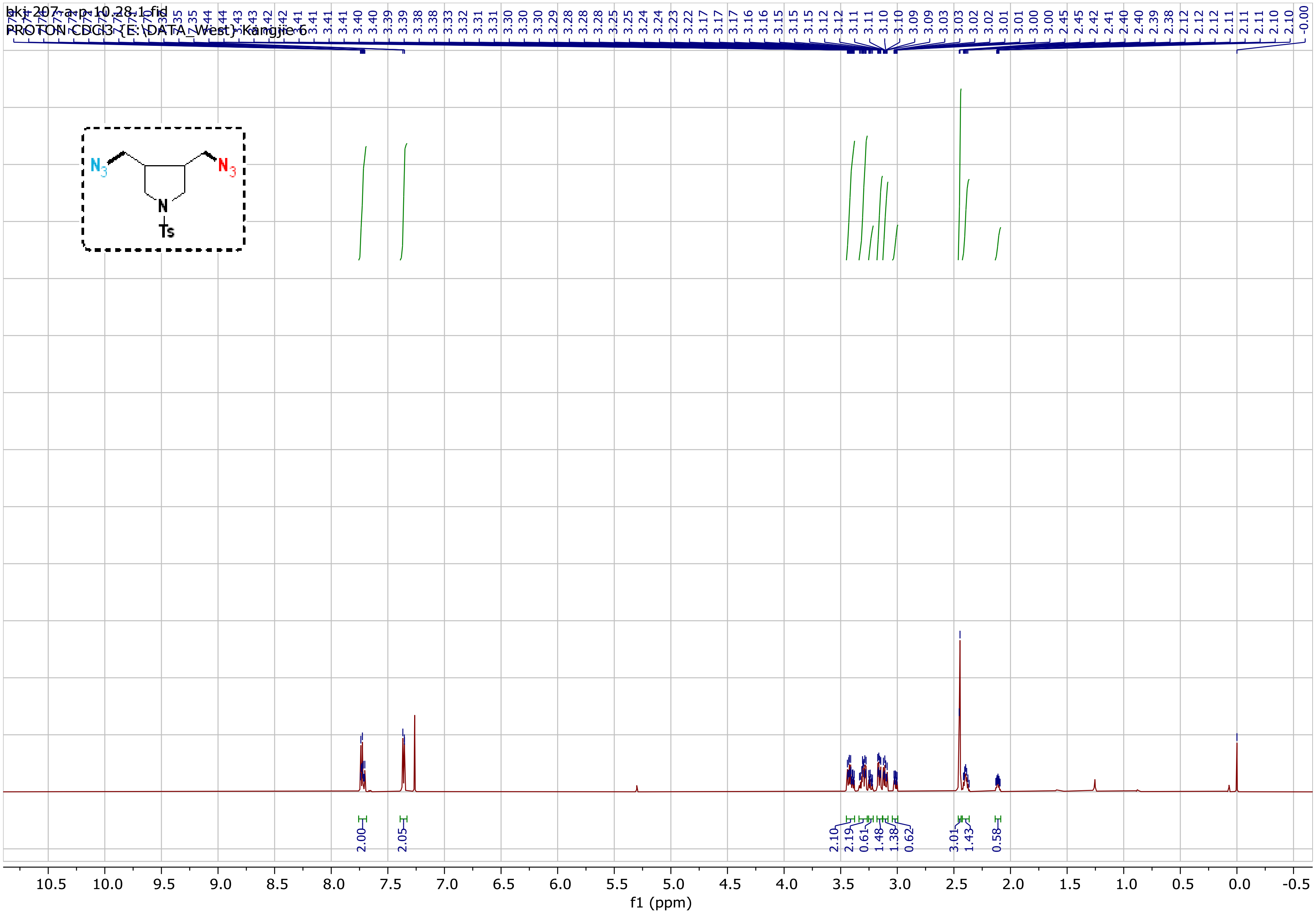

Supplementary Figure 117. ^{1}H NMR Spectrum of **56** (600 MHz, $CDCl_3$)

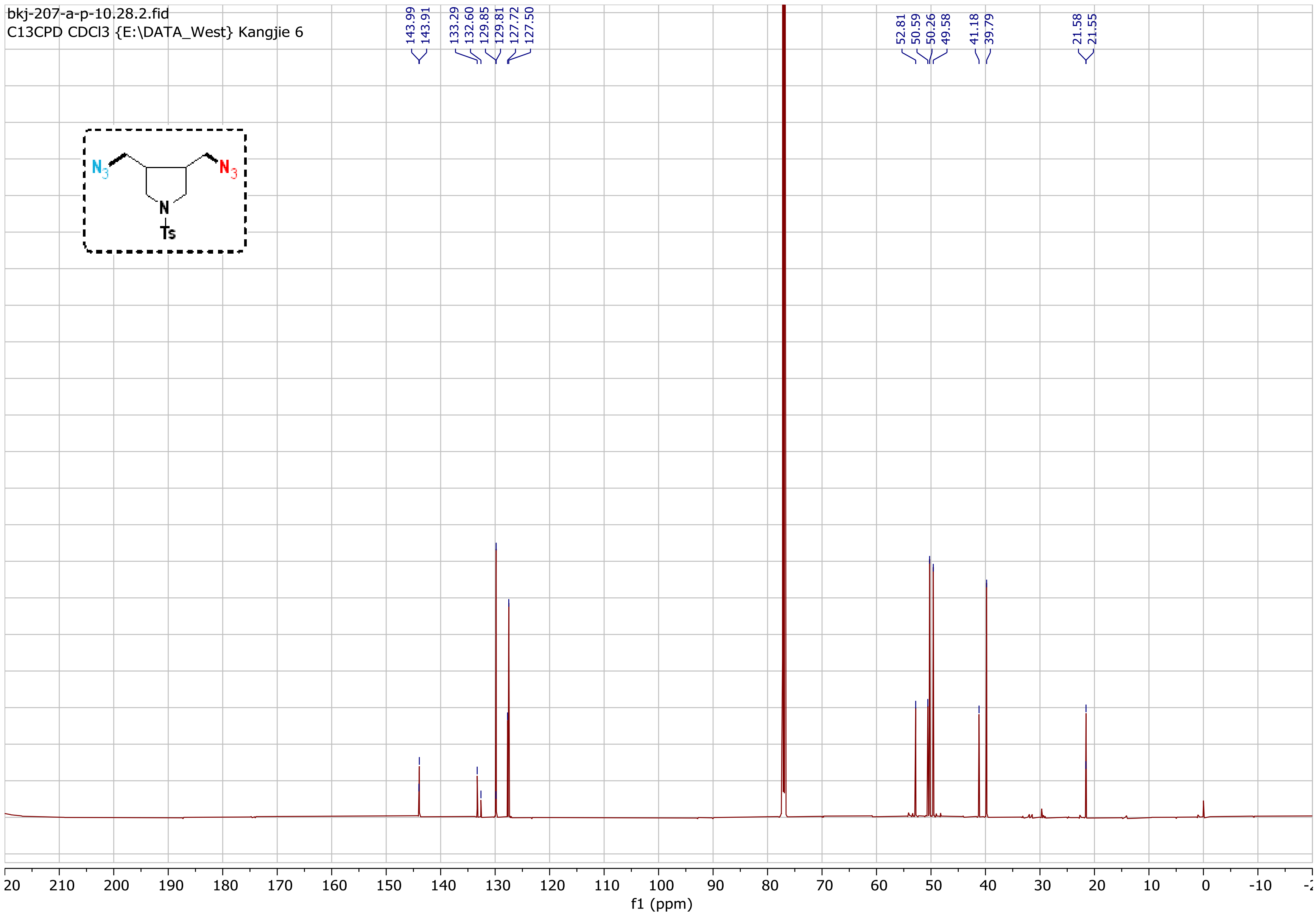

Supplementary Figure 118. ^{13}C NMR Spectrum of **56** (151 MHz, $CDCl_3$)

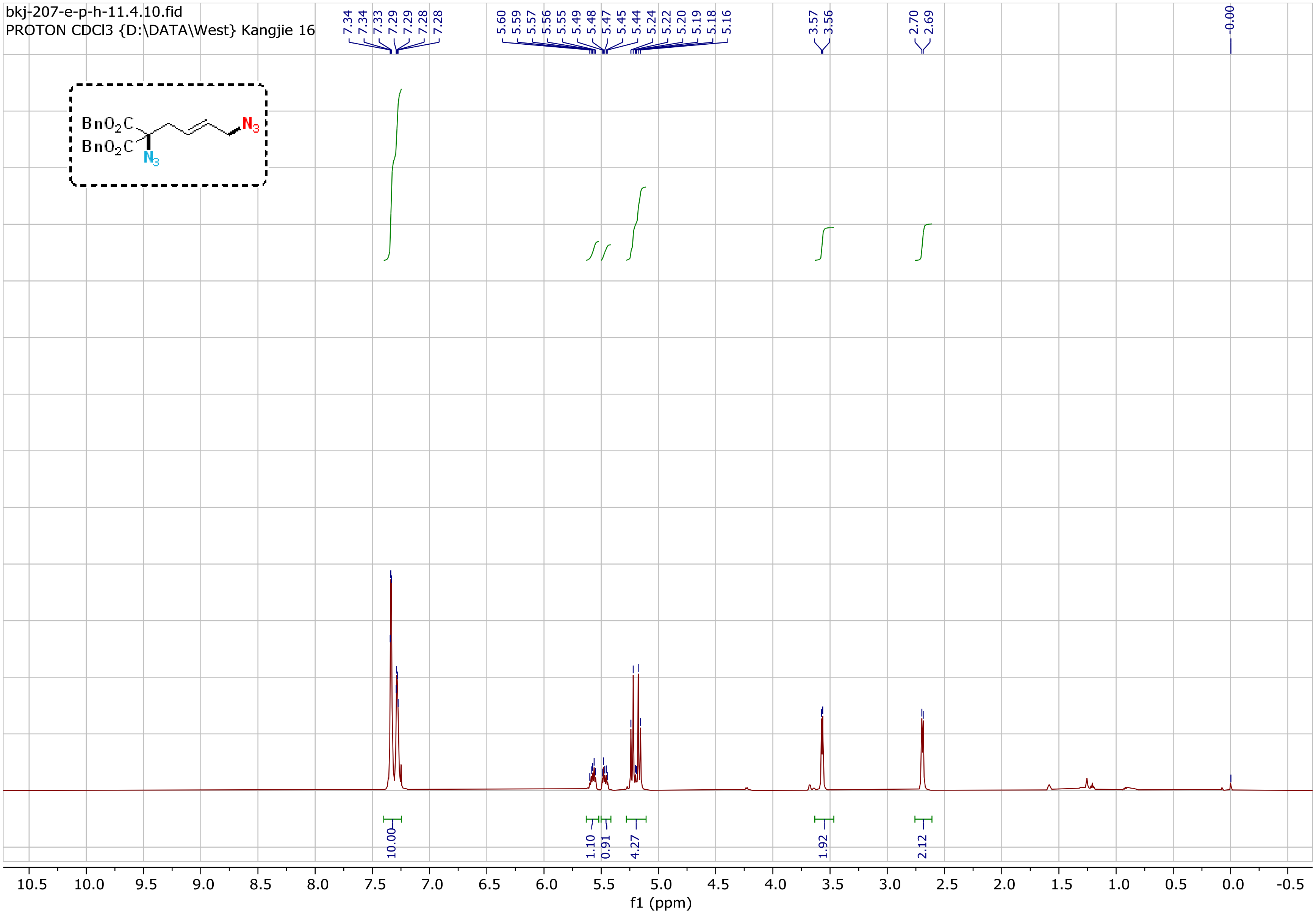

Supplementary Figure 119. ^{1}H NMR Spectrum of **57** (600 MHz, $CDCl_3$)

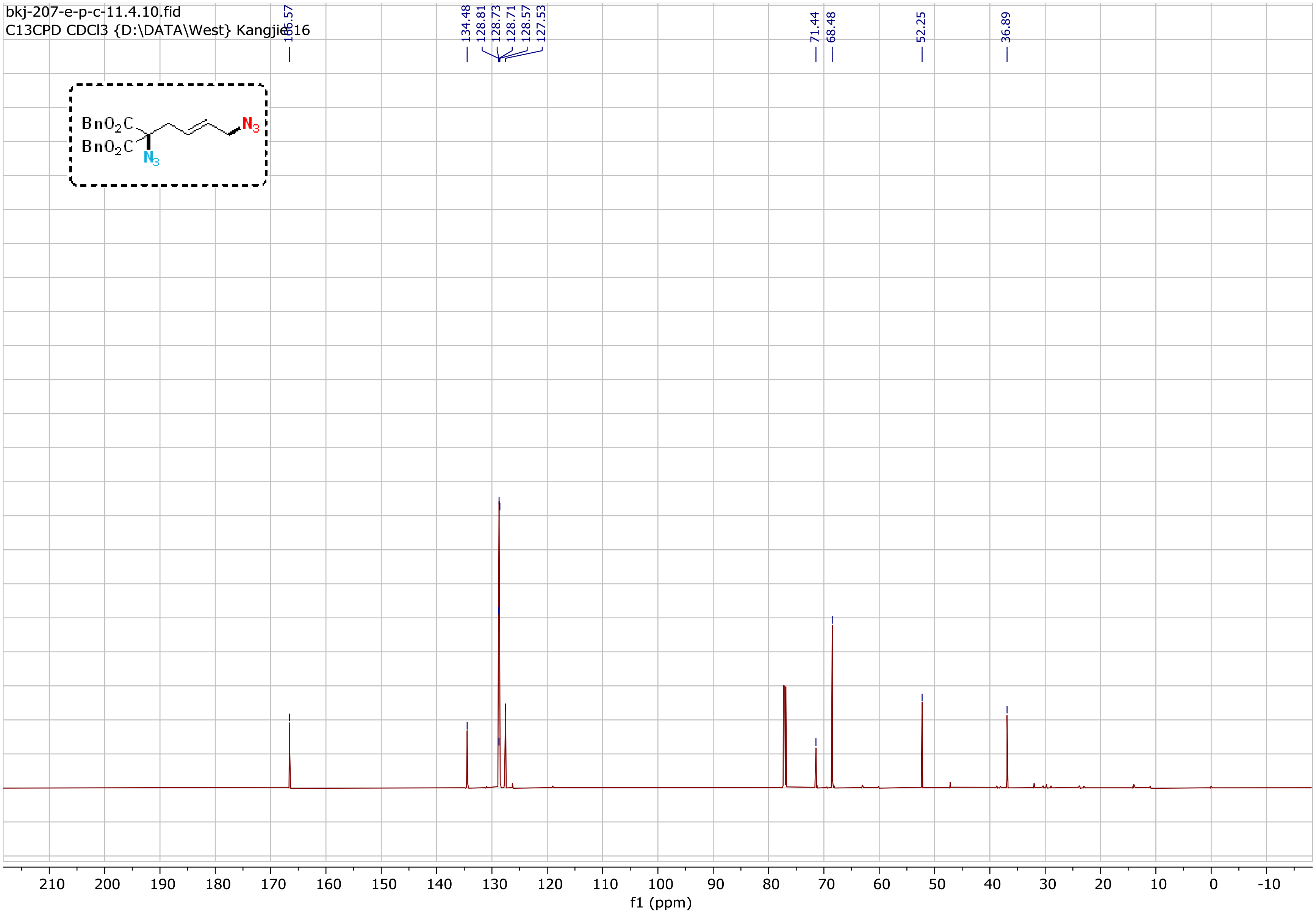

Supplementary Figure 120. ^{13}C NMR Spectrum of **57** (151 MHz, $CDCl_3$)

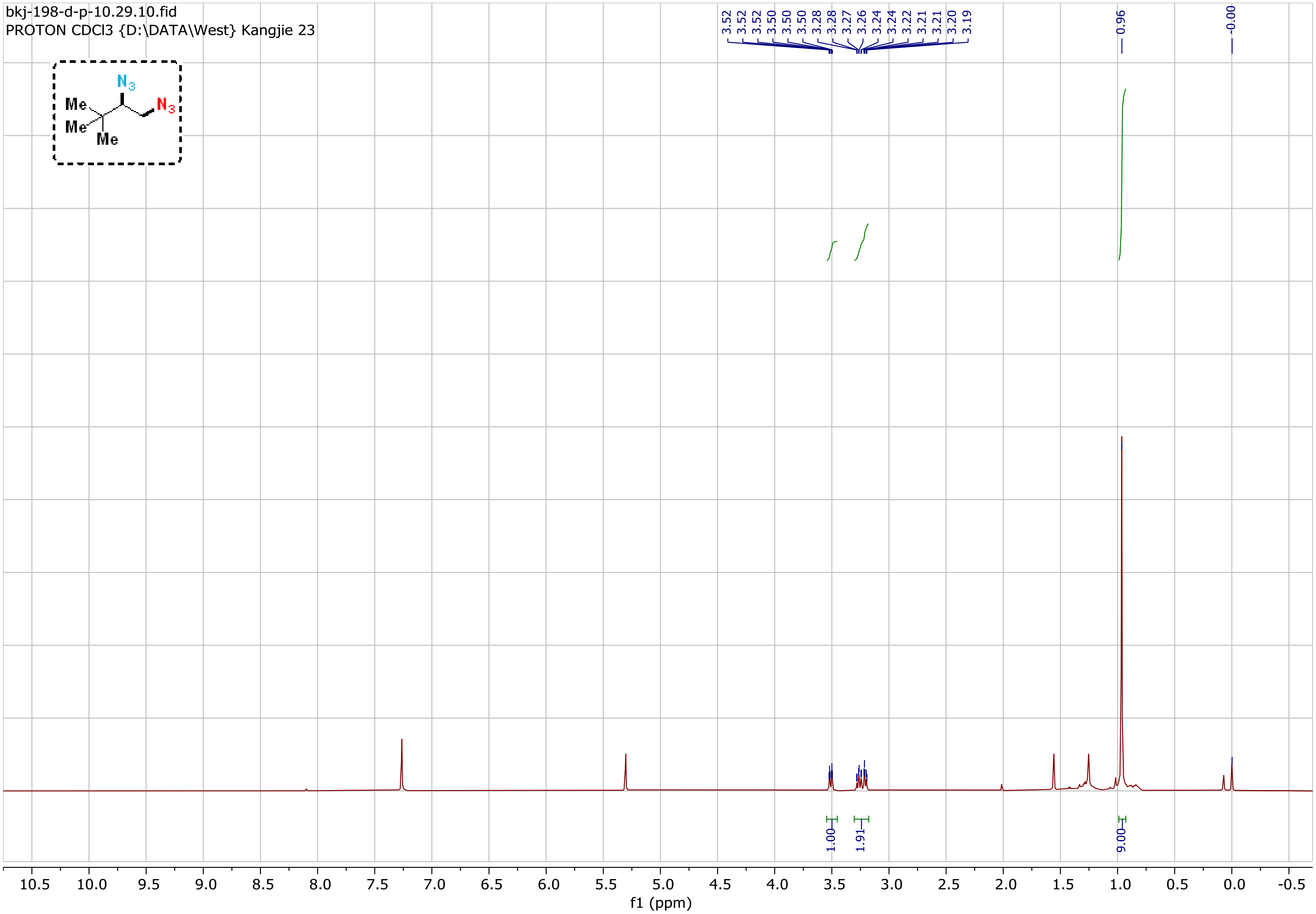

Supplementary Figure 121. ^{1}H NMR Spectrum of **58** (600 MHz, $CDCl_3$)

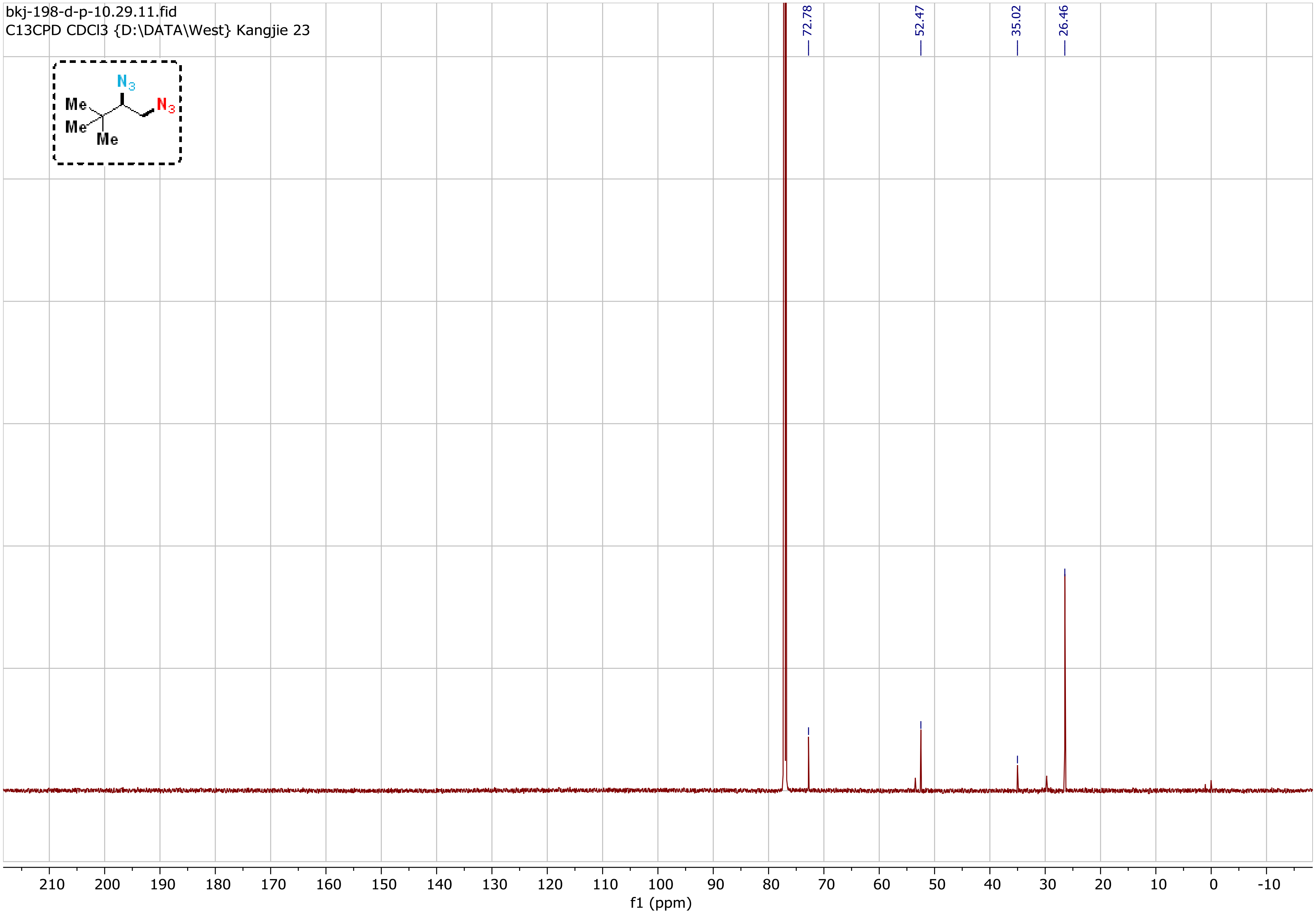

Supplementary Figure 122. ^{13}C NMR Spectrum of **58** (151 MHz, $CDCl_3$)

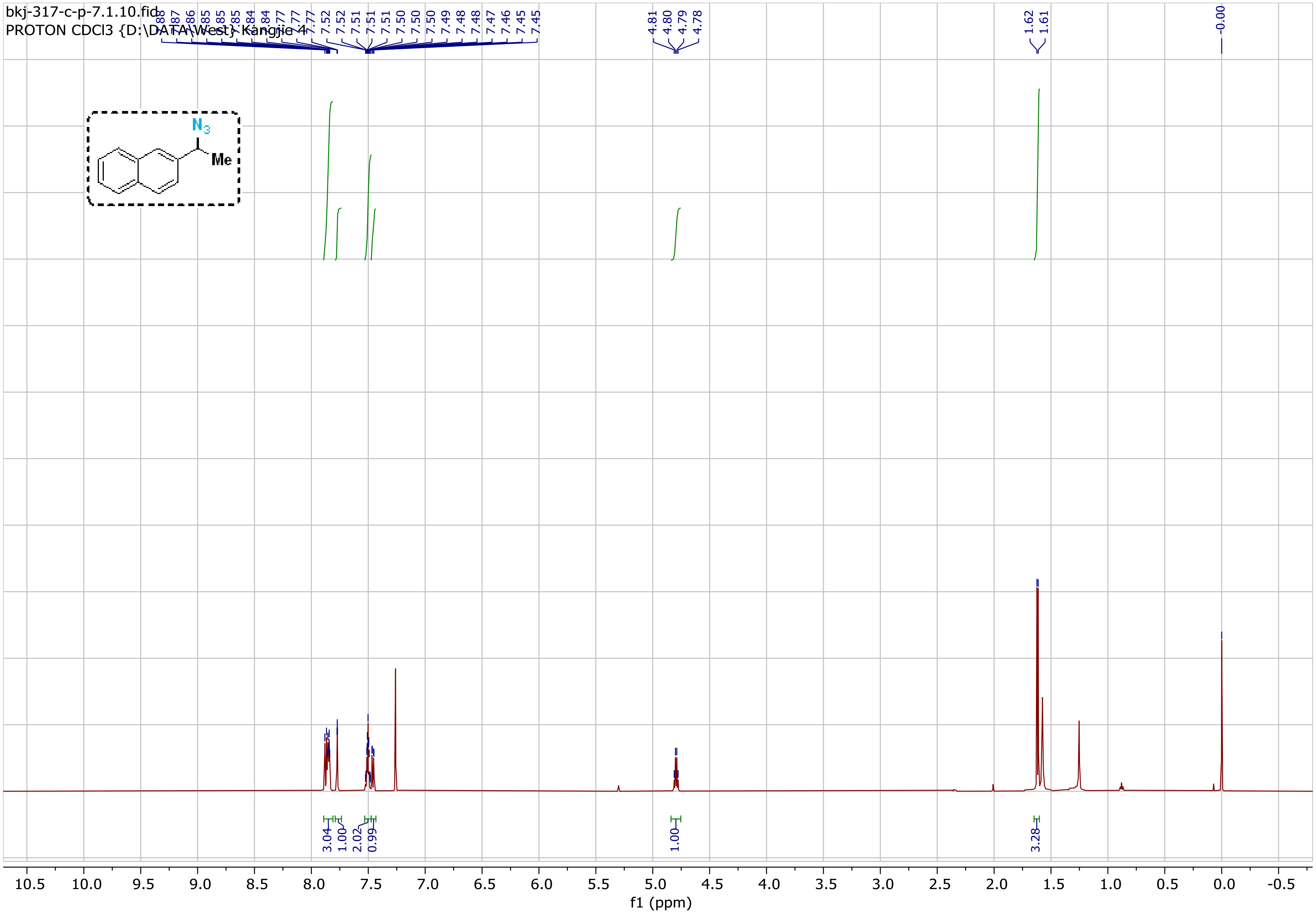

Supplementary Figure 123. ^{1}H NMR Spectrum of **59** (600 MHz, $CDCl_3$)

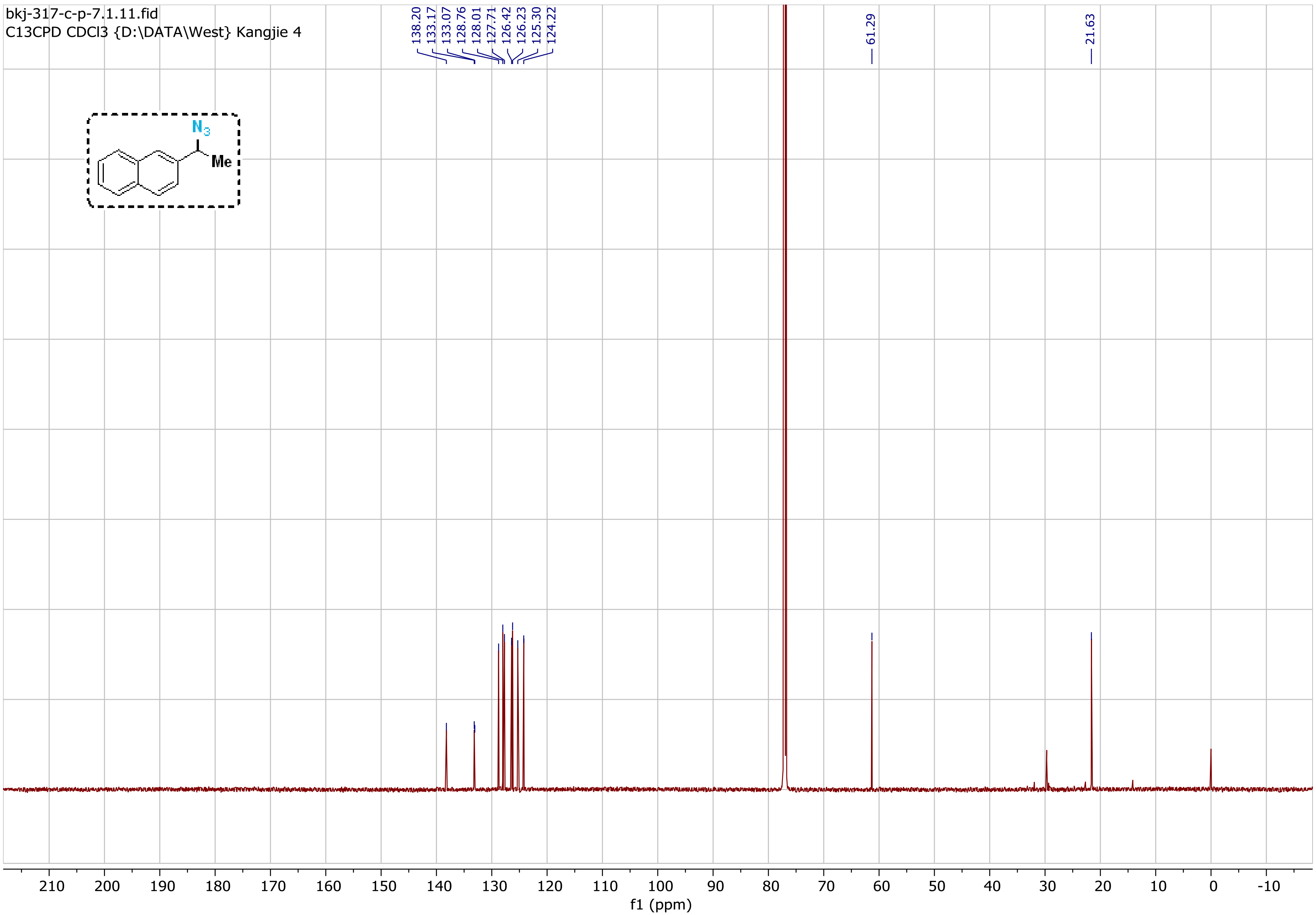

Supplementary Figure 124. ^{13}C NMR Spectrum of **59** (151 MHz, $CDCl_3$)

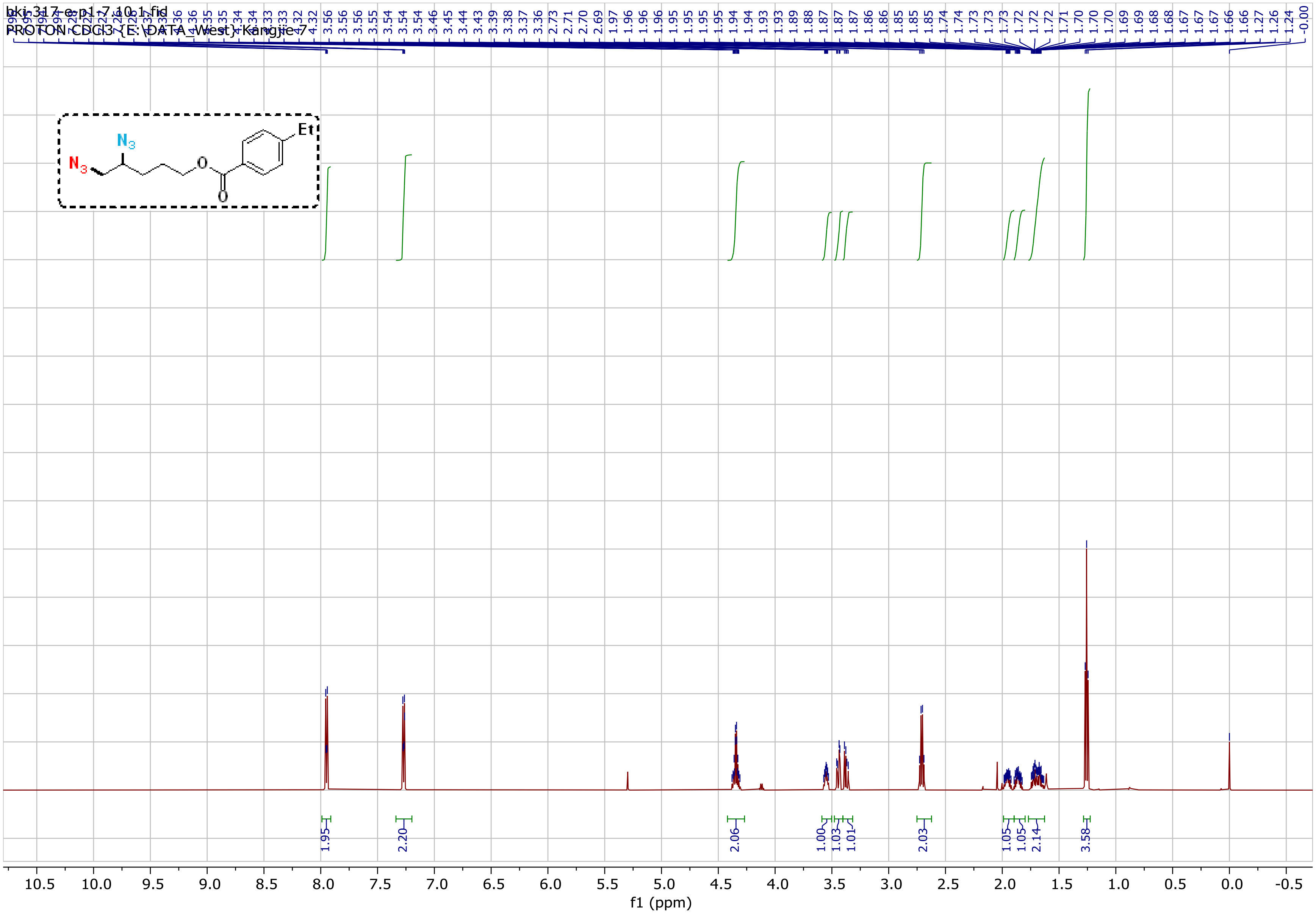

Supplementary Figure 125. ^{1}H NMR Spectrum of **60** (600 MHz, $CDCl_3$)

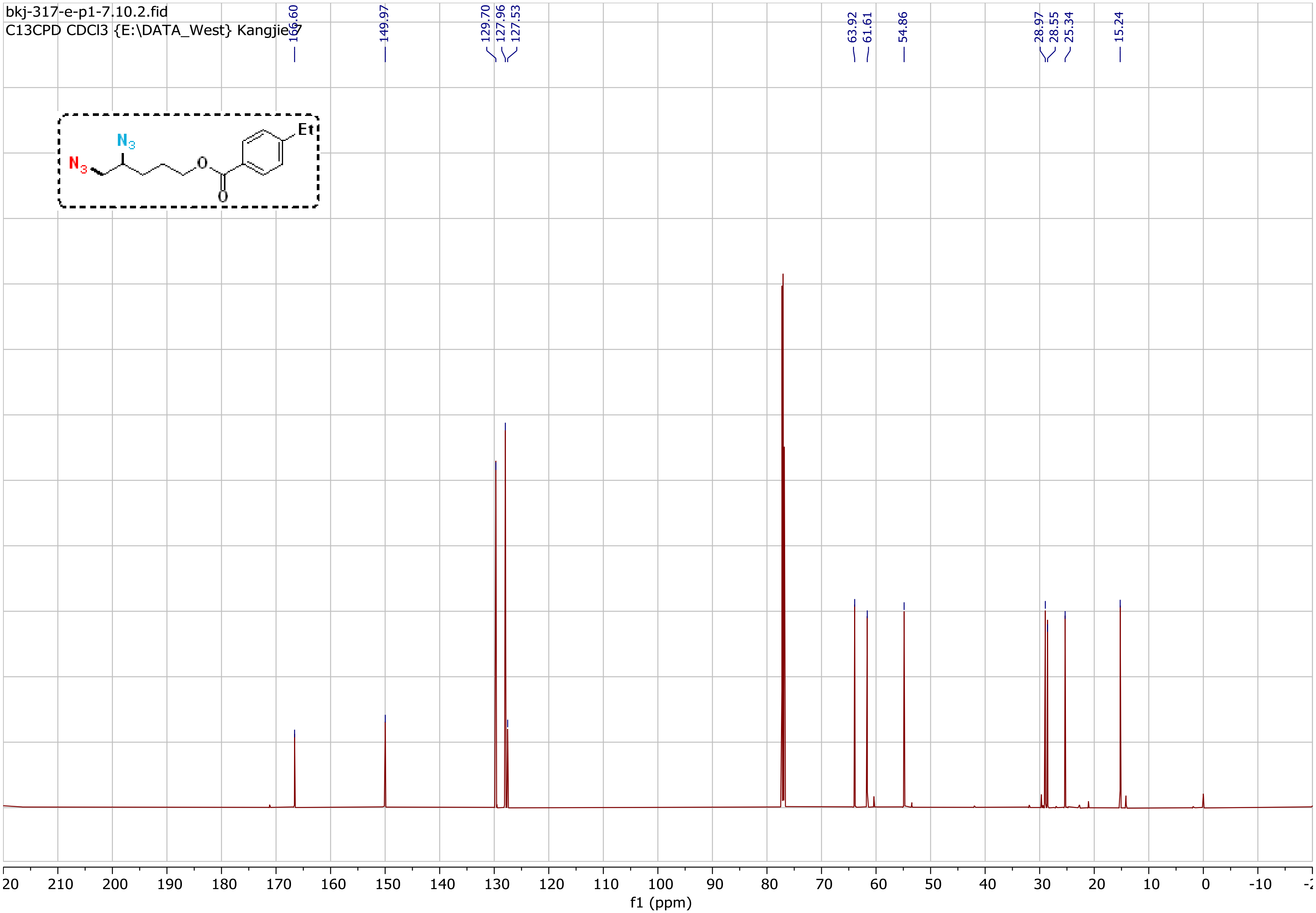

Supplementary Figure 126. ^{13}C NMR Spectrum of **60** (151 MHz, $CDCl_3$)

IV. Supplementary Reference

[1] Nikitas, N. F.; Voutyritsa, E.; Gkizis, P. L.; Kokotos, C. G. *Eur. J. Org. Chem.* **2021**, *2021*, 96.

[2] Liu, J.; Lu, C.; Wu, S.; Wu, X.; Wu, Z.; Xue, X.-S.; Yu, J.; Zhu, C. *Angew. Chem. Int. Ed.* **2020**, *59*, 8195.

[3] Jiang, H.-J.; Liu, K.; Yu, J.; Zhang, L.; Gong, L.-Z. *Angew. Chem. Int. Ed.* **2017**, *56*, 11931.

[4] C. Almansa, J. Alfón, A. F. de Arriba, F. L. Cavalcanti, I. Escamilla, L. A. Gómez, et al. *J. Med. Chem.* **2003**, *46*, 3463.

[5] Aldrich, C.; Brody, S.; Cushman, M.; Fan, B.-Z.; Hiasa, H.; Liang, J.-H.; Lv, W.; Yang, Z.-Y. *Eur. J. Med. Chem.* **2020**, *193*, 112222.

[6] Siu, J. C.; Parry, J. B.; Lin, S. *J. Am. Chem. Soc.* **2019**, *141*, 2825.

[7] Peng, H.; Yuan, Z.; Chen, P.; Liu, G. *Chin. J. Chem.* **2017**, *35*, 876.

[8] Shen, S.-J.; Zhu, C.-L.; Lu, D.-F.; Xu, H. *ACS Catal.* **2018**, *8*, 4473.

[9] Lee, B. K.; Min, S. K.; Hahm, H. S.; Dong, S. K.; Lee, W. K.; Ha, H. J. *Tetrahedron* **2006**, *62*, 8393.
